# The rising burden of male genitourinary cancers in middle- and high-income countries

**DOI:** 10.3389/fonc.2026.1762356

**Published:** 2026-05-20

**Authors:** Wenfu Song, Yingying Li, Zhilei Wang, Yaodong You, Zhou Yexin, Xinhui Wu, Jingyi Zhang, Jianyuan Tang, Diang Chen, Degui Chang, Ju Huang

**Affiliations:** 1 Hospital of Chengdu University of Traditional Chinese Medicine, Chengdu, China; 2 Sichuan Academy of Chinese Medicine Sciences, Chengdu, China; 3 Department of Respiratory Medicine, Hospital of Chengdu University of Traditional Chinese Medicine, Chengdu, China

**Keywords:** disease trends, epidemiology, GBD, male genitourinary cancers, middle- and high-income countries

## Abstract

**Introduction:**

Male genitourinary cancers (prostate, bladder, kidney, and testicular) represent a major cancer burden in middle- and high-income countries (MHIC). We aimed to characterize their distribution and trends across 174 countries using the 2023 Global Burden of Disease (GBD) database.

**Methods:**

This study presented the incidence, mortality, disability-adjusted life years (DALYs), and age-standardized rates for four male genitourinary cancers by age and location from 1990 to 2023. Average annual percent changes (AAPCs) and Bayesian Age-Period-Cohort (BAPC) models were utilized to quantify temporal dynamics and project trends to 2050.

**Results:**

In 2023, an estimated 2.16 million new cases and 0.73 million deaths were attributed to male genitourinary cancers in MHIC. From 1990 to 2023, age-standardized incidence rates (ASIRs) increased for PCa, KCa, and TCa across all income groups. However, age-standardized mortality rates (ASMRs) exhibited divergent trends: declining significantly in high-income countries (HC) while remaining stable or increasing in lower-middle-income countries (LMC). Smoking remained the predominant risk factor, though metabolic factors (high BMI, hyperglycemia) demonstrated increasingly prominent population-attributable fractions in wealthier nations. Projections to 2050 suggest a growing burden in LMC and upper-middle-income countries (UMC).

**Conclusion:**

The marked increase in ASIRs in wealthier nations partially reflects superior diagnostic capacity (e.g., PSA screening) rather than exclusively a true increase in disease burden. The divergence between rising incidence and persistently high mortality in LMC highlights the urgent need for tailored, resource-stratified public health strategies.

## Introduction

Cancer as the second foremost cause of death worldwide, and the burden of cancer is anticipated to increase significantly in the years ahead (1). In 2022, there were approximately 20 million new cancer cases and 9.7 million cancer deaths (2). Among these, Cancers of the male genitourinary system, including those of the prostate, bladder, kidney, and testis, constitute a substantial and growing share of the global cancer burden. Cancers not only impact personal health but also impose a significant strain on healthcare systems. The rising incidence and complex etiology of these cancers, particularly in middle- and high-income countries (MHIC), underscore the need for comprehensive surveillance and evidence-based prevention strategies. Male genitourinary cancers have varying epidemiological trends and risk factor attributions across regions. Characterizing the burden of these cancers and their attributable risk factors is essential for prioritizing prevention strategies in different settings.

MHIC account for a large and growing proportion of the global male genitourinary cancer burden (3). Despite generally stronger health systems and greater capacity for cancer control, many MHIC are experiencing increasing incidence of prostate cancer (PCa), bladder cancer (BCa) and Kidney cancer (KCa). This trend is likely influenced by population aging, shifts toward obesogenic lifestyles (characterized by high-calorie diets and physical inactivity), the widespread use of cancer screening (such as PSA testing, contributing to overdiagnosis), and incomplete control of key risk factors-including tobacco use in some upper-middle-income countries (UMC) (4–6). Moreover, substantial variations in cancer burden and risk factor exposure exist across MHIC, but comprehensive, comparable data on these patterns remain limited.

Characterizing the burden of male genitourinary cancers and their attributable risk factors is essential for effective prevention. Although disparities in incidence and mortality across countries and age groups have been documented (7), few studies have used comparable, population-level data to quantify how modifiable risk factors contribute to these differences. While previous studies have analyzed the global burden of genitourinary cancers utilizing earlier GBD iterations (e.g., GBD 2019 and 2021), the present study offers critical incremental contributions. First, we extend the temporal analysis to 2023, capturing recent epidemiological shifts. Second, we integrate Bayesian Age-Period-Cohort (BAPC) models to project disease burden out to 2050, explicitly incorporating demographic structural changes. Finally, we provide a refined attribution analysis detailing the transition from traditional behavioral risks (e.g., smoking) to metabolic factors specifically within the context of MHIC, exploring the “prevention paradox” wherein increased risk exposure in high-income settings paradoxically accompanies declining mortality.

## Methods

### Study overview

This study complies with the Guidelines for Accurate and Transparent Health Estimates Reporting (GATHER) recommendations (**Appendix 1 Supplementary Table 1**). Briefly, the GBD 2023 study consolidated global data on incidence, prevalence, and mortality to provide systematic and up-to-date assessments of the fatal and nonfatal health loss for 375 diseases and injuries in 204 countries and territories from 1990 to 2023. We extracted GBD 1990 to 2023 estimates for four male genitourinary cancers—prostate cancer (PCa; ICD-10 codes C61-C61.9), bladder cancer (BCa; C67-C67.9), kidney cancer (KCa; C64-C65.9), and testicular cancer (TCa; C62-C62.9), across 174 countries classified by the World Bank into High-Income (HC), Upper-Middle-Income (UMC), and Lower-Middle-Income (LMC) groups. Age groupings were categorized into 5-year intervals from 0–4 up to 95+ years. We evaluated incidence, mortality, and disability-adjusted life years (DALYs), which comprise years of life lost (YLLs) due to premature death and years lived with disability (YLDs), along with their 95% uncertainty intervals (UIs) (8).

### Data sources and processing

Data sources used to produce GBD 2023 estimates are listed in the GBD 2023 Sources Tool (https://ghdx.healthdata.org/gbd-2023/sources). Temporal trends in age-standardized rates from 1990 to 2023 were assessed using joinpoint regression to identify significant changes in trend direction. Analyses were conducted using Joinpoint software (version 5.4.0; National Cancer Institute, https://surveillance.cancer.gov/joinpoint/). A log-linear model (ln(rate) = β × year + ϵ) was fitted to estimate the APC and corresponding 95% CI for each identified segment. The average annual percent changes (AAPC) was calculated to summarize overall trends across the entire study period. Additionally, we employed a Bayesian Age-Period-Cohort (BAPC) model to assess the burden of genitourinary cancers. This methodology not only enhances our understanding of the dynamics of genitourinary cancer burden but also provides valuable insights into how these variables interact and impact disease outcomes across various demographics (9). The Bayesian Age-Period-Cohort (BAPC) model was implemented using the Integrated Nested Laplace Approximations (INLA) package in R. We specified a second-order random walk (RW (2)) for age, period, and cohort effects to ensure smooth transitions in trends. To ensure model transparency and reproducibility, non-informative log-gamma priors were assigned to the precision parameters ($log \text{-{{-}}-} gamma(1, 0.00005)). Model fit was rigorously evaluated using the Deviance Information Criterion (DIC) and the Watanabe-Akaike Information Criterion (WAIC), where lower values indicated superior parsimony and predictive accuracy. *Post-hoc* diagnostics, including the inspection of posterior distributions and effective sample sizes, confirmed model convergence.To account for demographic structural changes, projections incorporated the United Nations’ medium-fertility population growth variant. Regarding risk-attributable burden, the GBD comparative risk assessment framework was utilized, which estimates the joint population-attributable fraction (PAF) for coexisting risk factors by assuming a multiplicative effect and accounting for mediation between interrelated risks.

For visual representation of the results, figures were generated using R software (version 4.5.1). The statistical code used in GBD 2023 is publicly available online (http://ghdx.healthdata.org/gbd-2023/code).

### Role of the funding source

The funder of the study had no role in study design, data collection, data analysis, data interpretation, or writing of the report.

## Results

### Male genitourinary cancers burden in 2023

There were 2.16 million (95%UI 1.98-2.33) new male genitourinary cancers cases and 0.73 million (95%UI 0.69-0.78) male genitourinary cancers deaths estimated to MHIC. Premature mortality comprised the majority of the 148.9 million (140.0-157.9) MHIC male genitourinary cancers DALY in 2023 (**Table 1**), with 91.6% (90.3-93.0; 136.5 million [128.2-144.7]) of DALYs due to YLLs and 8.4% (7.0-9.8; 1.25 million [1.04-1.46]) due to YLDs. The male genitourinary cancer types contributing the greatest MHIC burden in terms of incident cases in 2023 were PCa (1.38 million [95%UI 1.22-1.55]), BCa (424–362 [385 927-462 798]), KCa(252–877 [225 003-280 751]), and TCa (97–447 [79 500-115 394]). The greatest contributors to MHIC male genitourinary cancers deaths in 2023 were PCa (448–469 [407 957-488 981]), BCa (167–428 [154732-180124]), KCa(105–519 [95 626-115 413]), and TCa (11–049 [9387-12 710]) (**Table 1**).

**Table 1:** Male genitourinary cancers incidence, death, DALY, YLL, YLD counts in 2023 by world band middle- and high-income group.

Cancer type	World Bank High Income	World Bank Upper Middle Income	World Bank Lower Middle Income	MHIC
Incidence, Cases in 2023, in thousands (95%UI)				
Prostate cancer	886 (752 to 1027)	352 (280 to 429)	143 (99 to 196)	1382 (1218 to 1546)
Male bladder cancer	237 (211 to 264)	141 (121 to 162)	47 (32 to 67)	424 (386 to 463)
Male kidney cancer	150 (132 to 167)	82 (67 to 107)	21 (14 to 30)	253 (225 to 281)
Testicular cancer	50 (36 to 63)	34 (25 to 43)	14.3 (7.9 to 22.2)	97 (80 to 115)
Mortality, Cases in 2023, in thousands (95%UI)				
Prostate cancer	215 (232 to 192)	143 (159 to 121)	90 (64 to 123)	448 (408 to 489)
Male bladder cancer	84 (77 to 89)	60 (53 to 67)	24 (16 to 34)	167 (155 to 180)
Male kidney cancer	59 (54 to 63)	35 (28 to 43)	12.0 (8.0 to 17.0)	106 (96 to 115)
Testicular cancer	2.7 (2.4 to 3.0)	4.9 (4.3 to 5.6)	3.4 (2.1 to 5.1)	11.0 (9.3 to 12.7)
DALYs, Cases in 2023, in thousands (95%UI)				
Prostate cancer	3861 (3485 to 4176)	2713 (2314 to 3038)	1820 (1277 to 2484)	8394 (7768 to 9020)
Male bladder cancer	1494 (1392 to 1608)	1272 (1155 to 1415)	576 (389 to 799)	3342 (3077 to 3608)
Male kidney cancer	1267 (1188 to 1353)	975 (791 to 1210)	376 (255 to 533)	2618 (2423 to 2813)
Testicular cancer	129 (114 to 150)	233 (206 to 264)	177 (107 to 266)	539 (451 to 628)
YLLs, Cases in 2023, in thousands (95%UI)				
Prostate cancer	3262 (2966 to 3509)	2508 (2126 to 2777)	1744 (1226 to 2388)	7514 (6919 to 8110)
Male bladder cancer	1371 (1285 to 1463)	1199 (1086 to 1337)	554 (371 to 763)	3123 (2874 to 3372)
Male kidney cancer	1206 (1131 to 1287)	941 (763 to 1175)	368 (248 to 522)	2516 (2330 to 2700)
Testicular cancer	104 (95 to 115)	217 (192 to 245)	171 (103 to 256)	492 (411 to 574)
YLDs, Cases in 2023, in thousands (95%UI)				
Prostate cancer	599 (435 to 809)	205 (136 to 290)	76 (48 to 116)	880 (721 to 1039)
Male bladder cancer	123 (89 to 163)	73 (51 to 99)	23 (14 to 34)	219 (174 to 264)
Male kidney cancer	61 (43 to 82)	34 (23 to 53)	7.9 (4.7 to 12.5)	104 (80 to 125)
Testicular cancer	26 (15 to 40)	15.7 (9.4 to 24.8)	5.6 (2.7 to 10.8)	47 (36 to 58)

**Figure 1:**
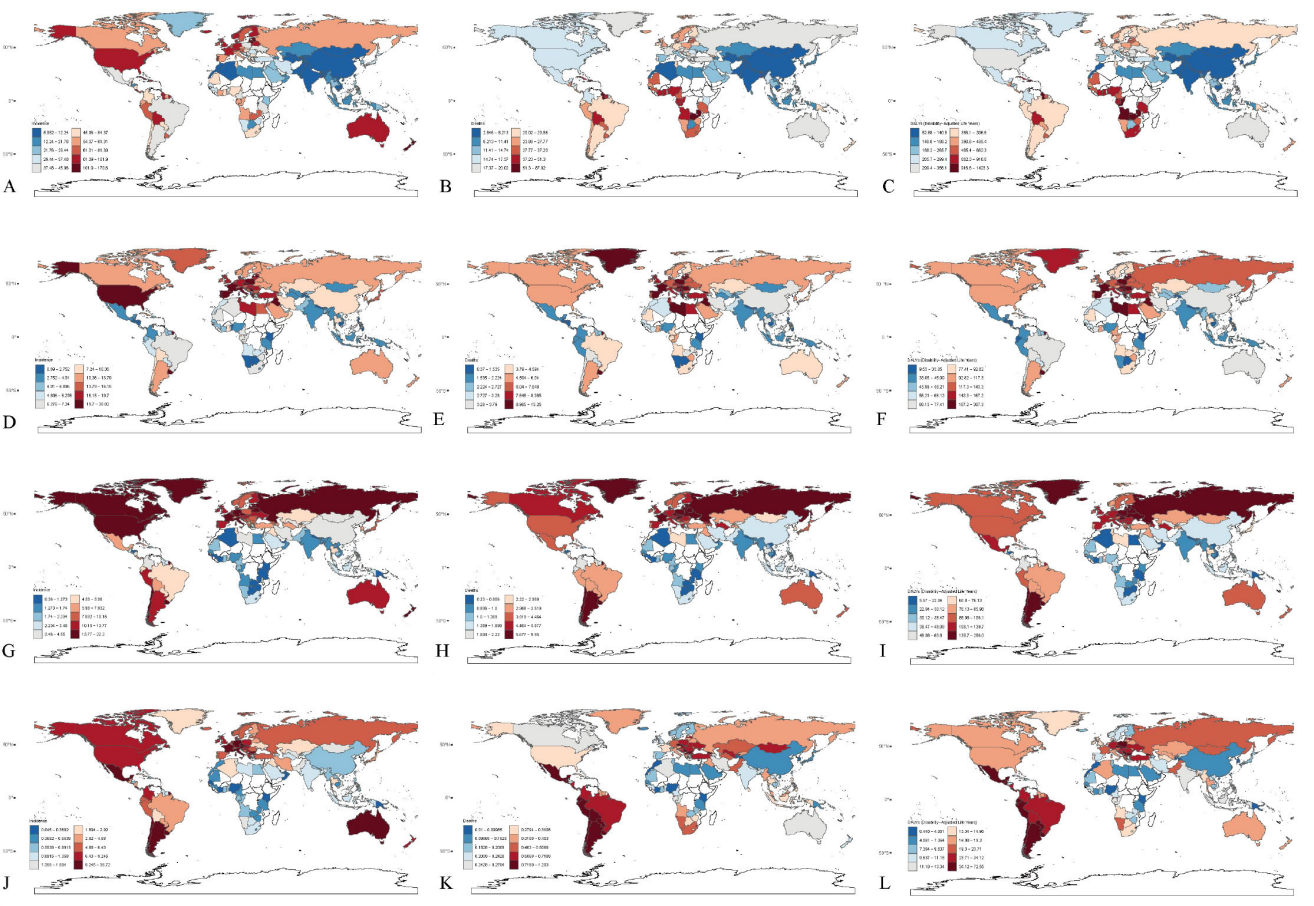
Male genitourinary cancers in 174 Middle- and high-income countries. Age-standardized incidence **(A)**, mortality **(B)** and DALYs rate **(C)** of prostate cancer. Age-standardized incidence **(D)**, mortality **(E)** and DALYs rate **(F)** of male bladder cancer. Age-standardized incidence **(G)**, mortality **(H)** and DALYs rate **(I)** of male kidney cancer. Age-standardized incidence **(J)**, mortality **(K)** and DALYs rate **(L)** of testicular cancer.

With the exception of TCa, the incidence, mortality, and DALY number for the other three male genitourinary cancers increased with higher national income levels. In contrast, while TCa exhibited the highest incidence in HC, both its mortality and DALY rates were highest in UMC (**Appendix 1 Table S4**). Similarly, in terms of age-standardized incidence, mortality, and DALY rates, all male genitourinary cancers showed the highest rates in HC, except for TCa mortality, which was slightly higher in UMC (**Appendix 1 Table S5**). The incidence rates, mortality rates and DALY rates of the 174 countries are shown in **Figure 1** and **Appendix 1 Table S5**.

In 2023, across MHIC, the incidence of male genitourinary cancers, except for TCa increased with age, showing a clear aging trend. PCa and BCa peaked in the 90–94 age group, while KCa reached its highest incidence in the 85–89 age group. In contrast, TCa predominantly affected young adults, with the highest incidence observed between the ages of 25 and 34. Mortality rates for all male genitourinary cancers consistently increased with advancing age. For DALYs rates, the age pattern mirrored that of incidence: they rose with age for all cancers except TCa, which followed the same age distribution as its incidence. Within the MHIC region, the incidence, mortality, and DALY rates of PCa, BCa, and KCa all increased with higher national income levels. In contrast, TCa burden was highest in UMC, followed by HC and LMC. The age-specific trends in YLLs and YLDs rates for PCa, TCa, BCa, and KCa closely mirror those of the overall DALYs rates. (**Appendix 1 Supplementary Figure 1–S4**).

### Trend in male genitourinary cancers burden, 1990-2023

Between 1990 and 2023, the age-standardized incidence rates (ASIR) of male genitourinary cancers increased across MHIC. PCa exhibited the most rapid rise, particularly in LMC (AAPC, 1.26 [95% CI, 1.20-1.32]) and UMC (AAPC, 1.17 [1.13–1.21]). In contrast, BCa and TCa exhibited divergent patterns: incidence more than doubled in both UMC and LMC, while increases in high-income countries were substantially smaller, with BCa rising by 78% and TCa by 58%. The burden of KCa increased across all regions. Notably, mortality and disability-adjusted life years (DALYs) for TCa declined by 10.5% and 15.4%, respectively, in HC (**Table 2**).

**Table 2:** Percentage change in incidence, mortality, and DALYs for male genitourinary cancers by MHIC, 1990-2023.

Cancer type	World Bank High Income	World Bank Upper Middle Income	World Bank Lower Middle Income
Incidence change (%)[95% UI]			
Prostate cancer	119.3 (76.4 to 182.0)	362.9 (265.2 to 507.0)	338.0 (179.1 to 591.6)
Male bladder cancer	78.2 (53.8 to 105.1)	169.1 (111.6 to 248.7)	189.0 (92.9 to 386.7)
Male kidney cancer	113.9 (79.3 to 156.8)	238.0 (182.2 to 323.5)	257.6 (139.7 to 441.5)
Testicular cancer	57.6 (9.8 to 137.6)	304.6 (174.2 to 511.3)	336.2 (92.1 to 806.3)
Mortality change (%)[95% UI]			
Prostate cancer	62.8 (50.5 to 73.7)	204.3 (160.1 to 248.1)	226.3 (108.7 to 415.0)
Male bladder cancer	66.1 ((56.8 to 77.2)	119.4 (81.2 to 161.5)	156.9 (72.5 to 322.7)
Male kidney cancer	80.8 (68.22 to 96.2)	176.4 (138.6 to 221.7)	221.7 (121.7 to 373.9)
Testicular cancer	-10.5 (-19.1 to -0.8)	74.1 (39.9 to 110.0)	130.7 (4.0 to 333.6)
DALYs change (%)[95% UI]			
Prostate cancer	52.2 (42.2 to 63.9)	181.2 (144.3 to 219.6)	213.2 (98.4 to 383.6)
Male bladder cancer	39.5 (31.4 to 48.6)	87.6 (52.7 to 123.4)	134.5 (56.9 to 123.4)
Male kidney cancer	47.8 (37.8 to 60.1)	119.1 (88.2 to 152.1)	179.7 (83.6 to 309.4)
Testicular cancer	-15.4 (-25.0 to -2.5)	66.1 (34.5 to 101.1)	122.0 (-1.9 to 317.6)

From 1990 to 2023, the ASIR of PCa, KCa and TCa increased across all World Bank income groups, while trends in BCa varied significantly by income level. PCa incidence rose most rapidly in LMC (AAPC, 1.26, [95% CI, 1.20-1.32]), followed by UMC (AAPC, 1.17 [1.13–1.21), with a modest increase in HC (AAPC, 0.17 [0.13–0.21]). In contrast, ASMRs declined sharply in HC (AAPC, −1.19 [−1.22 to −1.16]), remained stable or slightly decreased in UMC (AAPC, −0.35 [−0.40 to −0.30]), but increased in LMC (AAPC, 0.42 [0.35–0.48]) (**Appendix 1 Supplementary Table 6**).

BCa incidence decreased in HC (AAPC, −0.42 [95% CI, −0.48 to −0.38), showed no significant change in UMC (AAPC, −0.09 [−0.18 to 0.01]), and increased in LMC (AAPC, 0.38 [0.32-0.45). The ASMRs decreased in HC (AAPC, −0.89 [−0.94 to −0.85]) and UMC (AAPC, −0.79 [−0.90 to −0.64]), while it increased in LMC (AAPC, −0.08 [−0.15 to −0.02]). (**Appendix 1 Supplementary Table 6**).

KCa incidence increased in all income groups, with the fastest growth in LMC (AAPC, 1.40 [95% CI, 1.34–1.45]), followed by UMC (AAPC, 1.38 [1.34–1.42]), and HC (AAPC, 0.49 [0.45–0.52]). Mortality from male KCa declined in HC (AAPC, −0.27 [−0.32 to −0.23]), but increased in both UMC (AAPC, 0.28 [0.23–0.32]) and LMC (AAPC, 0.86 [0.80–0.93]).

TCa incidence rose dramatically across all income levels, with the steepest increases in UMC (AAPC, 3.42 [95% CI, 3.35–3.49]) and LMC (AAPC, 2.34 [2.23–2.48]), compared to a more moderate rise in HC (AAPC, 1.04 [0.99–1.11]). Notably, despite rising incidence, TCa mortality declined in HC (AAPC, −1.30 [−1.38 to −1.21]), whereas it increased in both UMC (AAPC, 0.09 [0.02–0.17]) and LMC (AAPC, 0.35 [0.20–0.51]), indicating worsening outcomes in resource-limited settings (**Appendix 1 Supplementary Table 6**).

### Disability-adjusted life years from male genitourinary cancers attributable to risk factors

In 2023, male genitourinary cancers were estimated to cause 2.8 million (95%UI, 2.5-3.1) DALYs attributable to risk factors exposure. **Table 3** illustrates the contribution of 5 factors across HC, UMC, and LMC in2023 to DALYs caused by male genitourinary cancers. In 2023, smoking was the leading attributable risk factor for PCa, BCa, and KCa among males across all World Bank income groups, with higher population-attributable fractions observed in HC and UMC. The proportion of PCa burden attributable to smoking ranged from 4.2% in LMC to 12.4% in high-income countries, with alcohol use contributing an additional 6.9% in high-income settings. For BCa, smoking accounted for 36.0%, 29.0%, and 14.0% of the disease burden in HC, UMC, and LMC, respectively, while high fasting plasma glucose contributed 13.6%, 7.1%, and 4.7% across the same groups. In KCa, high body mass index (BMI) emerged as the single largest risk factor in high-income countries, accounting for 26.4% of DALYs, surpassing smoking (14.9%); occupational exposure to trichloroethylene had a minimal contribution (<0.1%). Across all three cancer types, the attributable fractions of metabolic risk factors (high BMI, high glucose) and smoking were consistently higher in more affluent populations (**Appendix 1 Supplementary Table 7**).

**Table 3:** MHIC proportion of DALYs attributable to risk factors by male genitourinary cancers cause for all ages.

Locations	Risk factor of prostate cancer		Risk factor of male bladder cancer		Risk factor of male kidney cancer		
	Smoking	Alcohol use	Smoking	High fasting plasma glucose	Smoking	Occupational exposure to trichloroethylene	High body-mass index
World Bank High Income	12.4 (5.6 to 20.8)	6.9 (0.3 to 16.6)	36.0 (29.9 to 43.0)	13.6 (8.3 to 19.9)	14.9 (8.7 to 21.7)	0.03 (0.01 to 0.06)	26.4 (11.5 to 39.4)
World Bank Upper Middle Income	6.2 (2.8 to 10.9)	2.0 (0.1 to 4.6)	29.0 (24.6 to 34.3)	7.1 (4.4 to 10.6)	7.0 (4.3 to 10.0)	0.05 (0.01 to 0.10)	9.4 (4.2 to 14.6)
World Bank Lower Middle Income	4.2 (1.9 to 8.3)	1.2 (0.0 to 2.9)	14.0 (8.8 to 20.0)	4.7 (2.5 to 7.9)	2.0 (1.1 to 3.1)	0.03 (0.01 to 0.05)	2.9 (1.1 to 5.4)

### Risk-attributable male genitourinary cancers burden in 1990-2023

The total number of DALYs attributable to risk factors for male genitourinary cancers in MHIC increased from 1.5 million (95% UI: 1.3–1.8) in 1990 to 2.3 million (1.9–2.7) in 2023. From 1990 to 2023, the annual percent change in DALY rates attributable to risk factors for male genitourinary cancers varied across World Bank income groups. The ASDR for PCa attributable to alcohol use decreased in HC (AAPC, -2.3 [95%CI, -3.1 to -1.5]), UMC and LMC showed a stable trend. Moreover, the ASDR for BCa attributable to smoking decreased for HC (AAPC, -2.1 [-2.6 to -1.7]), as well as for UMC (AAPC, -1.7 [-2.2 to -1.1]) and LMC(AAPC, -1.4 [-2.2 to -0.5]). However, the ASDR for high fasting plasma glucose displayed a stable trend in MHIC. The ASDR for KCa attributable to smoking decreased in HC (AAPC, -1.6(-2.3 to -0.8). In addition, the ASDR for KCa attributable to high BMI increased trend in UMC (AAPC, 3.3 [0.8-5.9]), as well as for LMC (AAPC, 1.5 [0.3-2.8]). Apart from these, all the risk factors are showing a stable trend (**Appendix 1 Supplementary Table 8**).

### Male genitourinary cancers burden in the future up to 2050

**Figure 2:**
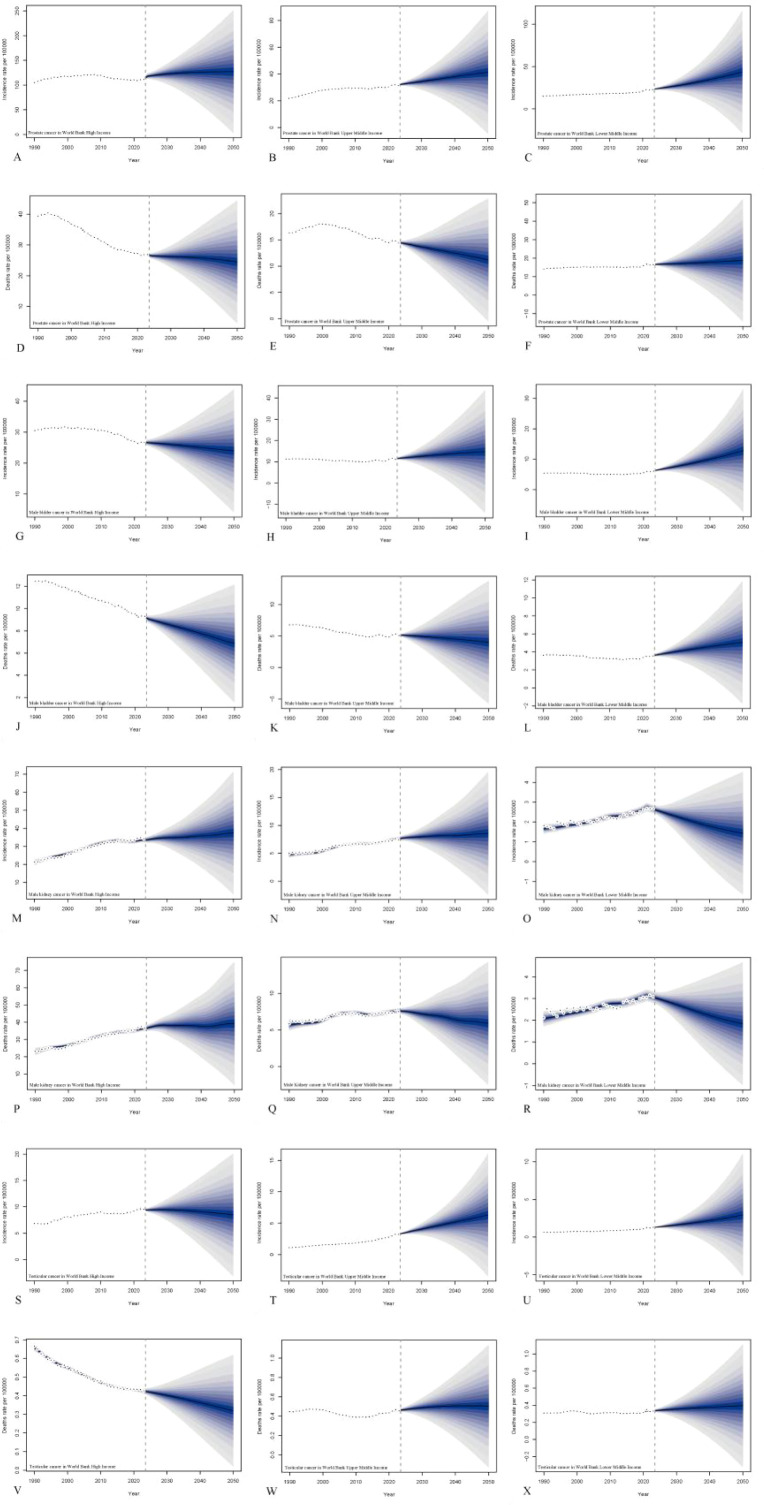
Prediction of male genitourinary cancers. **(A–F)** Predictions for ASIR and ASMR of prostate cancer in MHIC from 2023 to 2050, including HC **(A, D)**, UMC **(B, E)**, and LMC **(C, F)**. **(G–L)** Predictions for ASIR and ASMR for male bladder cancer in MHIC from 2023 to 2050, including HC **(G, J)**, UMC **(H, K)**, and LMC **(I, L)**. **(M–R)** Predictions for ASIR and ASMR for male kidney cancer in MHIC from 2023 to 2050, including HC **(M, P)**, UMC **(N, Q)**, and LMC **(O, R)**. **(S–X)** Predictions for ASIR and ASMR for testicular cancer in MHIC from 2023 to 2050, including HC **(S, V)**, UMC **(T, W)**, and LMC **(U, X)**. ASIRs, age-standardized incidence rates; ASMRs, age-standardized mortality rates.

By separately predicting the burden of genitourinary cancers across different World Bank Income regions, we identified significant disparities in the projected trends. The projections for PCa, BCa, KCa, and TCa from 2023 to 2050 show distinct trends in incidence and mortality across World Bank HC, UMC, and LMC. In HC, the incidence of PCa is projected to remain relatively stable, while BCa incidence shows a slight decline and KCa incidence continues to rise gradually. TCa incidence remains stable with minor fluctuations. Death rates for all four cancers in HC are projected to decrease or stabilize over time. In UMC, PCa incidence is expected to increase steadily, as are KCa and TCa incidence rates, while BCa incidence remains nearly constant. Mortality rates for all cancers in this group are projected to either stabilize or decline slightly. In LMC, incidence rates for PCa, BCa and KCa are projected to increase, with TCa incidence showing a moderate upward trend. Mortality rates for all four cancers in lower-middle-income areas are expected to remain relatively stable or show minor increases. Overall, the predicted trends indicate a growing burden of PCa, BCa and KCa in lower- and UMC, whereas HC may see stabilization or decline in both incidence and mortality (**Figure 2**).

## Discussion

As global economic disparities intensify, the burden of male genitourinary cancers exhibits a pronounced income-related gradient. Drawing on data from the GBD 2023, this study focuses on MHICs to elucidate how levels of economic development shape cancer epidemiology through differential access to healthcare, patterns of risk exposure, and the implementation of public health policies.

In the last thirty years, the ASIRs for all male genitourinary cancers have generally increased, except for a decrease noted in BCa. PCa, in particular, has emerged as one of the most prevalent and deadly cancers, representing the largest share of genitourinary cancer. This could be linked to the slow-growing characteristics of PCa and the extended survival periods seen in its patients in contrast to individuals with other types of genitourinary cancers (10). The advancement and widespread adoption of cross-sectional imaging technology have notably enhanced the incidental detection of KCa. While our findings show a correlation between higher income and increased prostate cancer incidence, these results should be interpreted as hypothesis-generating rather than definitive evidence of causality. Specifically, the observed trends may be partially attributed to intensified diagnostic practices, such as the widespread adoption of prostate-specific antigen (PSA) screening in high-income regions, which enhances the detection of early-stage or indolent tumors that might otherwise remain undiagnosed. Conversely, while UMC possess developing diagnostic infrastructure, LMC face severe deficits in basic healthcare access, contributing to delayed diagnoses and persistently high mortality (11). Furthermore, the diversification of cancer treatment, such as surgical intervention, biological therapy, and the emergence of novel therapeutic approaches, contributed to the decline in ASMRs and ASDRs of all male genitourinary cancer in economically advanced countries. It also indicates the insufficient treatment capacity for genitourinary cancers in UMC and LMC, highlighting the urgent need for these countries to enhance the ability for early detection, diagnosis, and treatment, increase investment in basic healthcare infrastructure, and learn from the healthcare policies of high-income countries (12). These measures are crucial for alleviating the socio-economic burden associated with these cancers.

Age is recognized as a significant risk factor for cancers of the prostate, bladder, and kidneys, and global population aging has contributed to their rising burden (13, 14). In this study, kidney cancer was the only genitourinary malignancy with notable incidence, mortality, and DALY rates in children under 14, driven by Wilms tumor (15). This indicates that while adult screening is a primary public health focus, pediatric oncology infrastructure remains a critical necessity across all income tiers to manage early-onset embryonal malignancies. HC bear a substantially higher disease burden across all age groups, a gap that widens with advancing age due to greater life expectancy and more complete case ascertainment. Additionally, TCa which primarily affects young men, also shows higher burden in high-income settings, partly reflecting its elevated incidence in populations of European ancestry and possibly higher rates of cryptorchidism (16). Interestingly, elderly men with TCa exhibit a higher mortality rate, which is similar to other genitourinary cancers. This may be because young patients often have more favorable prognoses and survival outcomes after early surgical intervention or radiotherapy (17).

In this study, it was found that smoking was a common risk factor for PCa, BCa and KCa. Elevated fasting plasma glucose levels were linked to a higher risk of BCa, whereas increased BMI and occupational exposure to trichloroethylene were correlated with an elevated risk of KCa. Notably, the population-attributable burden of smoking has declined globally, particularly in HC, reflecting stronger tobacco control policies and greater capacity to mitigate behavioral risk factors (6). Conversely, metabolic risks are rising. The impact of elevated BMI on KCa now exceeds that of smoking, especially in UMC, where rapid urbanization and dietary shifts have driven obesity rates upward in the absence of effective public health interventions. Similarly, the association between elevated fasting plasma glucose and BCa has strengthened, consistent with prior evidence (18). In the context of PCa, recent studies indicate that, alongside alcohol consumption and smoking, dietary factors such as high fat intake and obesity play a crucial role as significant risk elements (19). Regions with advanced economic growth tend to have greater exposure to these risk factors, which contributes to a higher prevalence of PCa. This pattern helps explain why HC exhibit higher ASIR despite better diagnostics: economic growth not only improves case detection but also increases exposure to obesogenic and carcinogenic lifestyles. To summarize, in addition to the enhancements in diagnostic and treatment advancements resulting from economic growth, the increased exposure to various risk factors associated with genitourinary cancers may help explain why individuals in wealthier nations present higher age-standardized rates of these cancers.

A notable paradox emerges when comparing risk exposure to mortality outcomes. Despite HC demonstrating a higher population-attributable fraction of DALYs driven by metabolic factors (e.g., high BMI, hyperglycemia), ASMRs for most genitourinary cancers in these regions are steadily declining. This suggests that the detrimental impacts of obesogenic lifestyles are currently being effectively buffered by robust healthcare systems. Comprehensive early detection programs and advancements in multi-modal therapeutics (such as targeted therapies and immunotherapies) have successfully offset the increased risk exposure. In contrast, LMC are undergoing a rapid epidemiological transition, facing increased exposure to Westernized diets without the corresponding therapeutic safety nets, resulting in a rising dual burden of incidence and mortality.

The projected trends in male genitourinary cancers from 2023 to 2050 highlight a growing divergence in cancer burden between differ-income countries. In HC, incidence rates for PCa and TCa remain stable, while KCa and BCa incidence continue to rise, likely reflecting increased detection through advanced imaging and shifting lifestyle risk factors such as obesity and smoking. While PCa mortality is declining in UMC, it remains stable or even increases in LMC, suggesting disparities in access to timely diagnosis and life-saving interventions. For TCa, an often curable disease with high survival when treated early, the stable or rising mortality in lower-resource settings is especially concerning and may reflect underdiagnosis or inadequate treatment capacity. Together, these findings suggest that while high-income countries are managing to reduce the fatal burden of genitourinary cancers through effective care delivery, middle-income countries are facing a dual challenge: rising incidence due to epidemiological transitions and insufficient health system capacity to mitigate mortality. This underscores the urgent need for scalable prevention strategies, strengthened early detection programs, and equitable investment in oncology services in resource-limited settings.

To ensure the reliability of our GBD-derived findings, we performed a qualitative cross-validation by comparing our results with independent national and international cancer registries. Our observed upward trajectories in the incidence of prostate and kidney cancers, particularly in high-SDI regions, are highly consistent with the latest reports from the Surveillance, Epidemiology, and End Results (SEER) program and the International Agency for Research on Cancer (IARC). For instance, the SEER 2024 update highlights a similar rebound in prostate cancer incidence and a steady rise in kidney cancer linked to metabolic factors. This alignment across multiple high-quality data sources reinforces the epidemiological plausibility of our global analysis (2, 20).

This study is subject to several limitations inherent to the GBD ecological framework. First, our analysis relies on the GBD 2023 modeled estimates. While the GBD framework employs a sophisticated ensemble modeling approach, its accuracy is inherently constrained by the quality of primary data, particularly in regions with less robust cancer surveillance. To address this, we cross-referenced our results with independent national registries like the SEER program and IARC’s GLOBOCAN reports, finding that our observed trajectories, such as the divergent trends in prostate cancer incidence and mortality are highly consistent with these authoritative sources. Second, the ecological design of this study constrains our ability to draw direct causal inferences; the associations identified between metabolic risk factors and cancer outcomes should be viewed as providing an epidemiological foundation for future mechanistic research. Third, while our models project the burden of male genitourinary cancers to 2050 based on historical trajectories, these estimates carry inherent uncertainties. They cannot fully account for future paradigm shifts, such as the implementation of novel screening policies (e.g., changes in PSA testing guidelines), the widespread availability of new targeted therapies, or unpredicted shifts in metabolic and lifestyle risk factors. Finally, the database lacks granular indicators such as specific screening protocols or genomic data, which limits a deeper exploration of the biological drivers behind regional disparities.

## Conclusions

Male genitourinary cancers constitute a growing public health challenge in MHIC with distinct income-related disparities. While HC are successfully mitigating mortality through advanced therapeutics despite shifting metabolic risk exposures, LMC face a dual threat of rising incidence and persistently high mortality. Tailored public health interventions are urgently required. Policymakers in HC and UMC should integrate metabolic risk surveillance (e.g., weight management, glycemic control) into urological survivorship programs. Simultaneously, international health initiatives must prioritize capacity-building in LMC, focusing on affordable early detection strategies and expanding access to basic surgical interventions, particularly for highly curable malignancies like TCa.

## Data Availability

The original contributions presented in the study are included in the article/**Supplementary Material**. Further inquiries can be directed to the corresponding authors.
