# Supplementary material for: The rising burden of male genitourinary cancers in middle- and high-income countries
Source: Front Oncol. 2026 May 20;16:1762356. doi: 10.3389/fonc.2026.1762356 (PMC13229648; doi:10.3389/fonc.2026.1762356)
Supplement: Supplementary file 1 [file DataSheet1.docx]

Appendix 1 for “The Rising Burden of Male Genitourinary Cancers in Middle- and High-Income Countries”

Table of Contents

[Section 1 List of acronyms and abbreviations 3](#_Toc23114)

[Section 2 Guidelines for Accurate and Transparent Health Estimates Reporting (GATHER) compliance 4](#_Toc109)

[Table S1 GATHER checklist.GATHER checklist of information that should be included in reports of global health estimates, with description of compliance and location of information for "The Evolving Burden and Future Projections of Male Genitourinary Cancers and Associated Risk Factors in Middle- and High-Income Countries, 1990–2050: A systematic analysis for the Global Burden of Disease Study 2023” 4](#_Toc9389)

[Section 3 Supplementary figures and table 6](#_Toc19172)

[Figure S1 The burden of prostate cancer in different age groups 6](#_Toc23183)

[Figure S2 The burden of male bladder cancer in different age groups 7](#_Toc13067)

[Figure S3 The burden of male kidney cancer in different age groups 8](#_Toc10190)

[Figure S4 The burden of testicular cancer in different age groups 9](#_Toc20686)

[Table S2 Gross national income (GNI) per capita in 2023 10](#_Toc31267)

[Table S3 List of International Classification of Diseases (ICD) codes for genitourinary cancers. 11](#_Toc8853)

[Table S4 Incidence, deaths, DALYs, YLDs, and YLDs number of Prostate, Male bladder, Male kidney, and Testicular Cancer in 2023 12](#_Toc18335)

[Table S5 Age-standardized incidence, deaths, DALYs, YLDs, and YLDs rate of Prostate, Male bladder, Male kidney, and Testicular Cancer in 2023 28](#_Toc6730)

[Table S6 Average annual percent change of age-standardized incidence, mortality, DALYs, YLLs, and YLDs rate from 1990 to 2023 41](#_Toc17952)

[Table S7 Age-standardized DALYs rates for prostate, male bladder, and male kidney cancers attributable to risk factors in 2023. 51](#_Toc23436)

[Table S8 Age-standardized DALYs rate of average annual percent change attributable to risk factors for male genitourinary cancers from 1990 to 2023 55](#_Toc817)

# Section 1 List of acronyms and abbreviations

| **Abbreviation/acronym** | **Full phrase** |
| --- | --- |
| APCs | annual percent changes |
| AAPC | average annual percent changes |
| ASDR | age-standardized disability-adjusted life year rate |
| ASIR | age-standardized incidence rate |
| ASR | age-standardized rate |
| ASMR | age-standardized mortality rate |
| ASYLDRate | age-standardized years lived with disability rate |
| ASYLLRate | age-standardized years of life lost rate |
| BAPC | Bayesian Age-Period-Cohort |
| BMI | body-mass index |
| CI | confidence interval |
| DALYs | disability-adjusted life-years |
| GATHER | Guidelines for Accurate and Transparent Health Estimates Reporting |
| GBD | Global Burden of Disease |
| HC | high-income countries |
| IARC | International Agency for Research on Cancer |
| ICD | International Classification of Diseases |
| LMC | lower-middle-income countries |
| MHIC | middle- and high-income countries |
| MCMC | Markov Chain Monte Carlo |
| PAF | population attributable fraction |
| PSA | prostate specific antigen |
| UIs | uncertainty intervals |
| UMC | upper-middle-income countries |
| YLDs | years lived with disability |
| YLLs | years of life lost |

# Section 2 Guidelines for Accurate and Transparent Health Estimates Reporting (GATHER) compliance

This study complies with GATHER recommendations.3 See table S1 below for the GATHER checklist. The GATHER recommendations can be found on the[GATHER website.](https://www.who.int/data/gather)

## Table S1 GATHER checklist.GATHER checklist of information that should be included in reports of global health estimates, with description of compliance and location of information for "The Evolving Burden and Future Projections of Male Genitourinary Cancers and Associated Risk Factors in Middle- and High-Income Countries, 1990–2050: A systematic analysis for the Global Burden of Disease Study 2023”

| **Item #** | **Checklist item** | **Reported location** |
| --- | --- | --- |
| Objectives and funding | | |
| 1 | Define the indicator(s), populations (including age, sex, and geographic entities), and time period(s) for which estimates were made. | Manuscript (Methods) |
| 2 | List the funding sources for the work. | Manuscript (Funding) |
| Data Inputs | | |
| For all data inputs from multiple sources that are synthesized as part of the study: | | |
| 3 | Describe how the data were identified and how the data were accessed. | Methods: "Our analysis used publicly available secondary data from the GBD 2023 study." Data Sources Tool:https://ghdx.healthdata.org/gbd-2023/sources Results. https://vuzhub.healthdata.org/gbd-results |
| 4 | Specify the inclusion and exclusion criteria. Identify all ad-hoc exclusions. | Methods: "Since GBD 2023, except for kidney cancer, does not include data on the genitourinary cancers burden for individuals under 15 years old, our focus was primarily on those aged 15 years and over."No other ad hoc exclusions reported. |
| 5 | Provide information on all included data sources and their main characteristics. For each data source used, report reference information or contact  name/institution, population represented, data collection method, year(s) of data collection, sex and age range, diagnostic criteria or measurement method, and  sample size, as relevant. | Methods: "Data sources used to produce GBD 2023 estimates are listed in the GBD 2023 Sources Tool (https://ghdx.healthdata.org/gbd-2023/sources)." Appendix 1 Table S2: World Bank income classification based on 2023 GNI per capita. Appendix 1 Table S3: ICD codes for genitourinary cancers. |
| 6 | Identify and describe any categories of input data that have potentially important biases (e.g., based on characteristics listed in item 5). | Discussion: "Due to limited access to imaging technologies and healthcare infrastructure in UMC and LMC, there is a delay in diagnosis, which in turn increases the disease burden." Limitations: "Availability and quality of raw data pose a limiting factor..." |
| For data inputs that contribute to the analysis but were not synthesized as part of the study: | | |
| 7 | Describe and give sources for any other data inputs. | Not applicable; all data used were directly from GBD 2023. No additional non-synthesized data were used. |
| For all data inputs: | | |
| 8 | Provide all data inputs in a file format from which data can be efficiently  extracted (e.g., a spreadsheet rather than a PDF), including all relevant meta-data listed in item 5. For any data inputs that cannot be shared because of ethical or  legal reasons, such as third-party ownership, provide a contact name or the name of the institution that retains the right to the data. | GBD results are publicly accessible via: https://vuzhub.healthdata.org/gbd-results  GBD code is available at: http://ghdx.healthdata.org/gbd-2023/code  All data are open-access and downloadable in machine-readable formats (CSV, Excel). |
| Data analysis | | |
| 9 | Provide a conceptual overview of the data analysis method. A diagram maybe helpful. | Methods: "We employed the APC model to analyze trends in male genitourinary cancers incidence across different age groups, time periods, and birth cohorts." "Bayesian Age-Period-Cohort (BAPC) model" was also used. Joinpoint regression for trend analysis. |
| 10 | Provide a detailed description of all steps of the analysis, including mathematical formulae. This description should cover, as relevant, data cleaning, data pre-  processing, data adjustments and weighting of data sources, and mathematical or statistical model(s). | Methods: - Incidence, mortality, DALYs sourced from GBD 2023 with 95% UIs. - AAPC calculated using joinpoint regression: ln(rate) = β × year + ε. - BAPC model using Bayesian methods and MCMC simulation. - PAF calculation: Population Attributable Fraction formula provided. |
| 11 | Describe how candidate models were evaluated and how the final model(s) were selected. | Methods: "Simulations confirmed that estimates and uncertainty were not impacted by reducing draws from 500 to 250." Model selection based on standard GBD protocols and validation procedures described in GBD methods literature. |
| 12 | Provide the results of an evaluation of model performance, if done, as well as the results of any relevant sensitivity analysis. | Not explicitly reported in manuscript. However, GBD uses extensive validation and uncertainty quantification across multiple iterations. Reference to GBD methodology: "Analytical methods... are described in detail in the GBD methods literature." |
| 13 | Describe methods for calculating uncertainty of the estimates. State which sources of uncertainty were, and were not, accounted for in the uncertainty analysis. | Methods: "Uncertainty was introduced throughout the estimation process." "Mean estimates... represent the mean value across 250 draws... with 95% UIs calculated as the 2.5 and 97.5 percentile values." "Statistical significance determined by whether the 95% CI of the AAPC excludes zero." Uncertainty includes variability in data inputs, modeling assumptions, and parameter estimation. |
| 14 | State how analytic or statistical source code used to generate estimates can be accessed. | Methods: "The statistical code used in GBD 2023 is publicly available online (http://ghdx.healthdata.org/gbd-2023/code)." |
| Results and Discussion | | |
| 15 | Provide published estimates in a file format from which data can be efficiently extracted. | GBD 2023 results are available through online data visualization tools, the Global Health Data Exchange, and the online data query tool |
| 16 | Report a quantitative measure of the uncertainty of the estimates (e.g. uncertainty intervals). | Uncertainty intervals are provided with all results.Uncertinty is available at: https://ghdx.healthdata .org/record/ihme data/gbd-2023-cause specific-mortality-1990-2023 |
| 17 | Interpret results in light of existing evidence. If updating a previous set of estimates, describe the reasons for changes in estimates. | Discussion of methodological changes between GBD rounds provided in the narrative of the anuscript and methods appendix. Manuscript (Methods and Discussion) |
| 18 | Discuss limitations of the estimates. Include a discussion of any modelling assumptions or data limitations that affect interpretation of the estimates. | Discussion of limitations provided in the narrative of the manuscript, as well as in the methodological rite  ups in the methods appendix. Manuscript (Limitations) |

# Section 3 Supplementary figures and table

## Figure S1 The burden of prostate cancer in different age groups


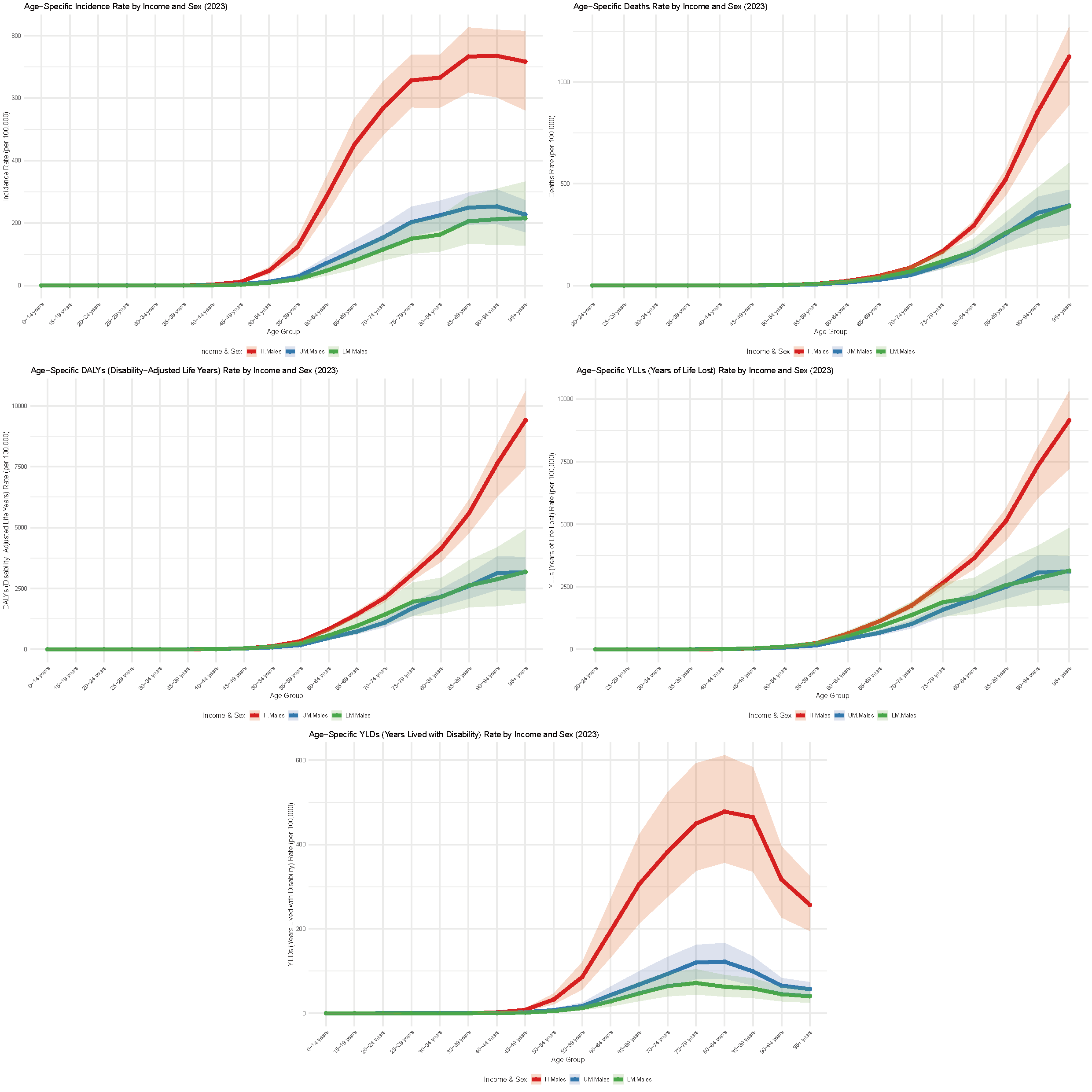


## Figure S2 The burden of male bladder cancer in different age groups


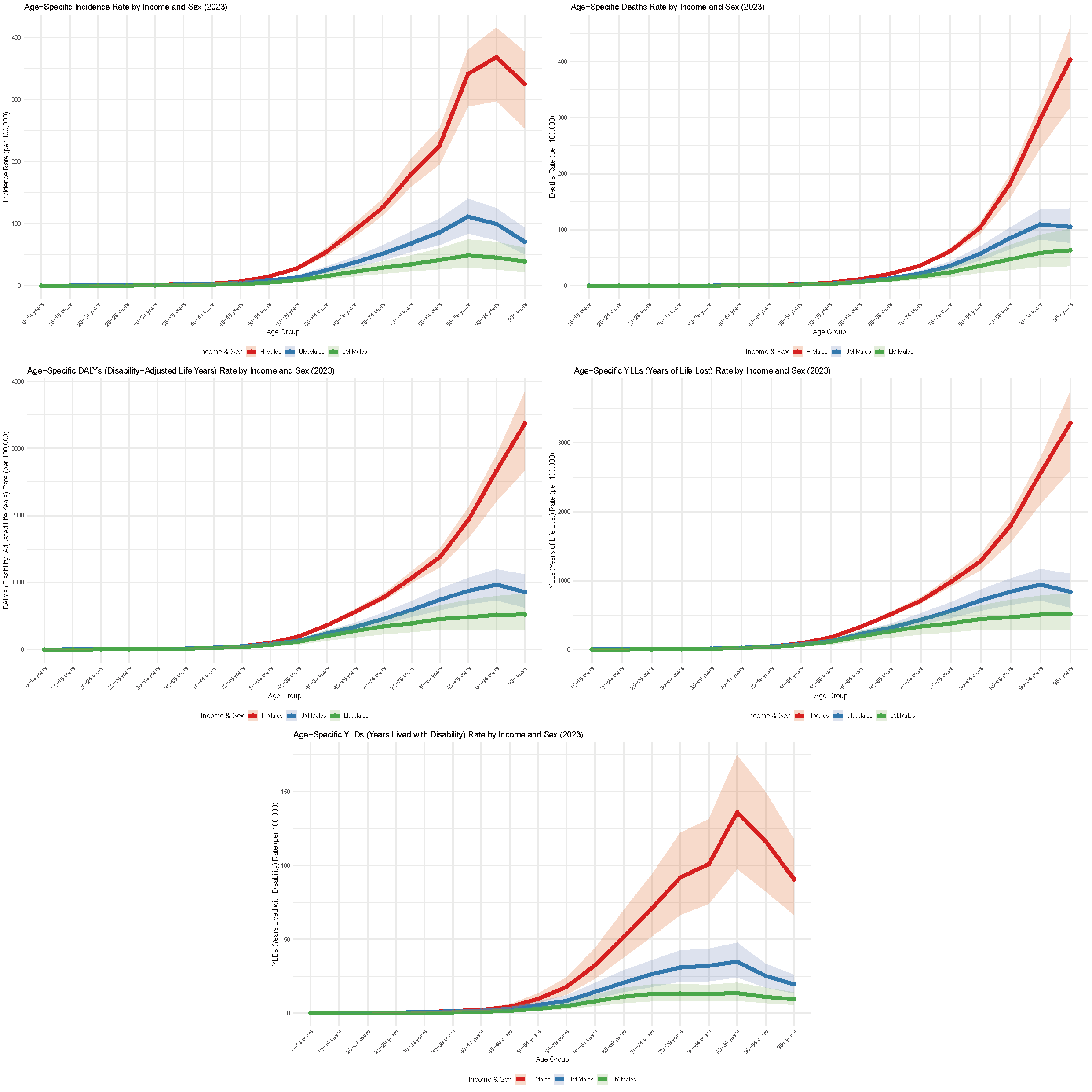


## Figure S3 The burden of male kidney cancer in different age groups


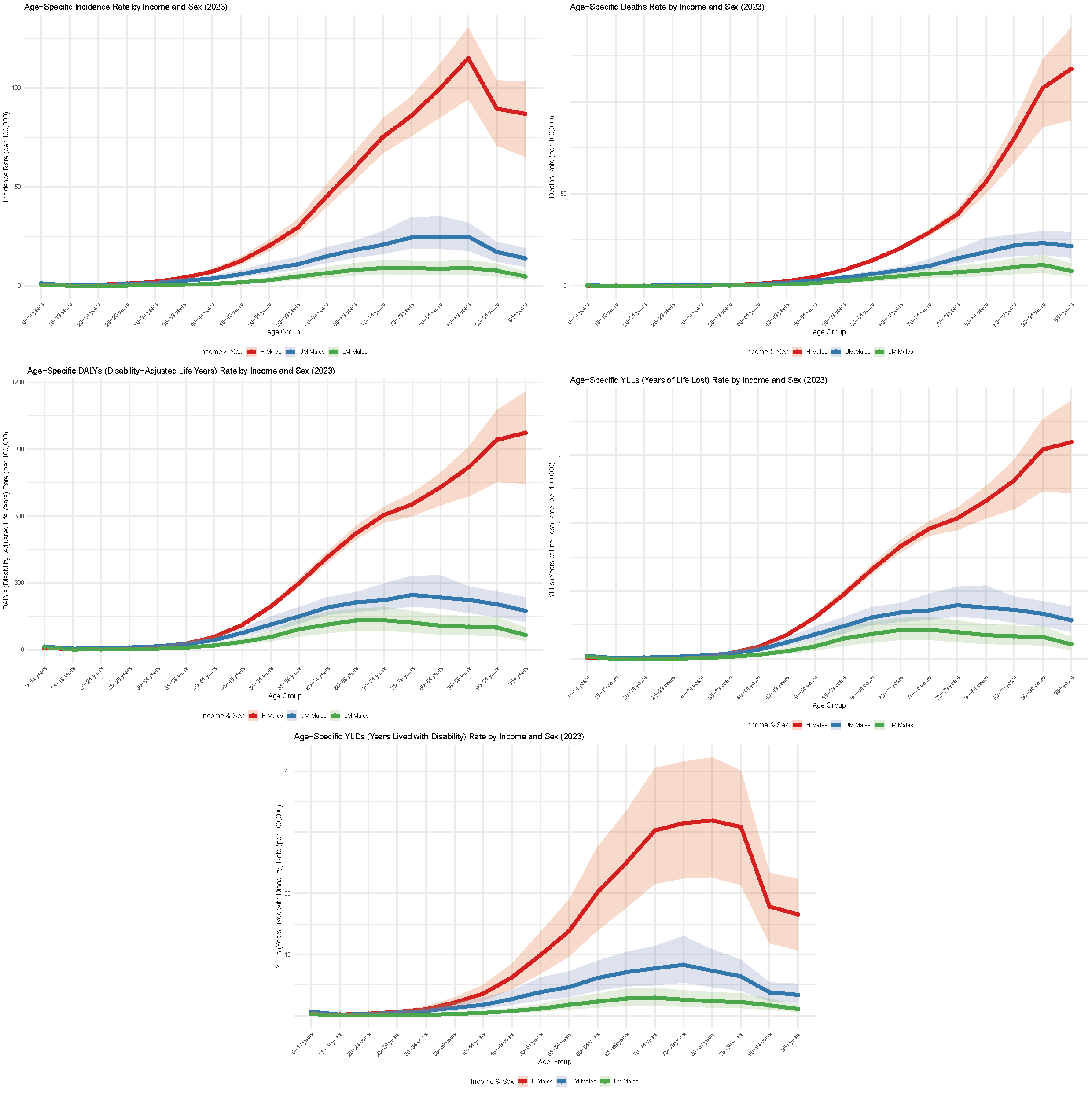


## Figure S4 The burden of testicular cancer in different age groups


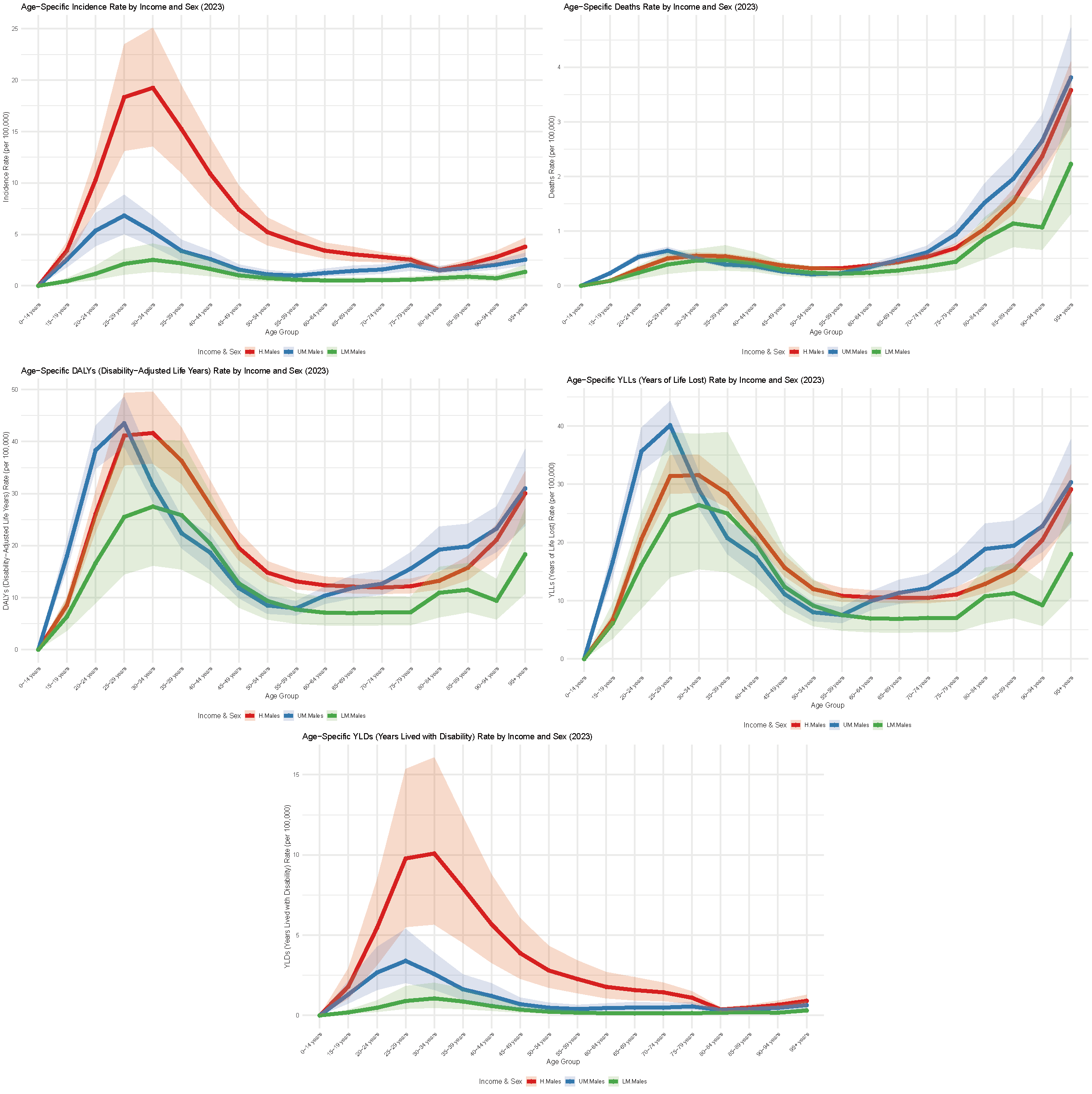


## Table S2 Gross national income (GNI) per capita in 2023

| *Bank's fiscal year:* | **FY25** |
| --- | --- |
| *Data for calendar year :* | **2023** |
| *Low income (L)* | <= 1,145 |
| *Lower middle income (LM)* | 1,146 - 4,515 |
| *Upper middle income (UM)* | 4,516 - 14,005 |
| *High income (H)* | > 14,005 |

## Table S3 List of International Classification of Diseases (ICD) codes for genitourinary cancers.

| **Cause** | | **ICD-10** | **ICD-11** |
| --- | --- | --- | --- |
| **Genitourinary cancers** | Prostate cancer | C61, D07.5, D29.1, D40.0 | 2C82, 2C82.0, 2C82.Y, 2C82.Z, 2E67.5, XH5C49, XH4ZC3, XH4PB1, XH8747 |
|  | Bladder cancer | C67-C67.9, D09.0, D30.3, D41.4 | 2C94-2C94., 2C94.Y, 2C94.Z, 2E68 |
|  | Kidney cancer | C64, C65, D30.0, D30.1, D41.0, D41.1 | 2C90, 2C90.0, 2C90.Y, 2C90.Z, 2C91, 2C91.0, 2C91.Y, 2C91.Z, XH5085, XH8EN1, XH05V6, XH3Z08, XH0RU3, XH7K79, XH4KK0, XH1D07, XH6153, XH9DH7, XH9T60, XH7PR9 |
|  | Testicular cancer | C62, C62.0, C62.1, C62.9, D29.2, D29.3, D29.4, D40.1 | 2C80.0-2C80.7, 2C80.Y, 2C80.Z, XH2855, XH0BG7, XH7E53, XH29E0, XH3BT2, XH4KB9, XH9G68, XH7051, XH9E02, XH0UP7, XH6FQ9, XH8U56, XH6XB6, XH3PN1, XH5BN5, XH4H24, XH7RD2, XH51L7 |

## Table S4 Incidence, deaths, DALYs, YLDs, and YLDs number of Prostate, Male bladder, Male kidney, and Testicular Cancer in 2023

| **Locations** | **Prostate cancer** | | | | | **Male bladder cancer** | | | | | **Male kidney cancer** | | | | | **Testicular cancer** | | | | |
| --- | --- | --- | --- | --- | --- | --- | --- | --- | --- | --- | --- | --- | --- | --- | --- | --- | --- | --- | --- | --- |
|  | **Incidence number, 95%UI** | **Death number, 95%UI** | **DALYs number, 95%UI** | **YLLs number, 95%UI** | **YLDs number, 95%UI** | **Incidence number, 95%UI** | **Death number, 95%UI** | **DALYs number, 95%UI** | **YLLs number, 95%UI** | **YLDs number, 95%UI** | **Incidence number, 95%UI** | **Death number, 95%UI** | **DALYs number, 95%UI** | **YLLs number, 95%UI** | **YLDs number, 95%UI** | **Incidence number, 95%UI** | **Death number, 95%UI** | **DALYs number, 95%UI** | **YLLs number, 95%UI** | **YLDs number, 95%UI** |
| World Bank High Income | 886361.6 (751753.4 to 1027413.3) | 215,332.0 (231,651.5 to 191565.0) | 3860957.0 (4175919.1 to 3484908.2) | 3262351.6 (2965614.6 to 3509323.5) | 598605.4 (434898.7 to 808527.5) | 237068.3 (210767.3 to 264834.0) | 83562.7 (76942.4 to 89274.1) | 1493867.3 (1391612.6 to 1608026.2) | 1370592.4 (1284909.3 to 1463407.3) | 123274.9 (89203.7 to 163015.2) | 149584.3 (132355.9 to 167297.8) | 58501.5 (53568.7 to 62825.2) | 1267309.4 (1188230.0 to 1352557.0) | 1206345.9 (1130569.0 to 1287445.7) | 60963.6 (42907.7 to 82099.2) | 49527.9 (36221.1 to 63384.9) | 2667.5 (2444.4 to 2977.4) | 129465.3 (113751.9 to 149991.2) | 103626.7 (94634.8 to 115377.2) | 25838.6 (15202.6 to 39706.3) |
| World Bank Upper Middle Income | 352254.3 (279962.7 to 428922.1) | 142923.3 (159078.6 to 120804.2) | 2713123.4 (3038354.0 to 2314150.4) | 2508053.8 (2126162.5 to 2776692.1) | 205069.6 (136176.3 to 289648.3) | 140559.0 (120710.7 to 162355.7) | 59644.7 (52758.3 to 66600.5) | 1271978.9 (1154596.4 to 1414918.3) | 1198881.4 (1086209.6 to 1337132.1) | 73097.5 (50673.0 to 98936.9) | 82182.4 (67112.3 to 107397.6) | 35009.4 (28404.7 to 43412.0) | 974627.2 (791494.4 to 1209704.1) | 940847.4 (762963.9 to 1174568.2) | 33779.8 (23195.7 to 52866.7) | 33520.9 (24782.9 to 43377.1) | 4947.1 (4332.3 to 5589.5) | 232981.5 (205573.5 to 264312.7) | 217283.8 (192173.1 to 245234.8) | 15697.6 (9391.5 to 24847.1) |
| World Bank Lower Middle Income | 143460.4 (195568.5 to 99207.5) | 90213.8 (122702.5 to 63599.8) | 1820355.4 (2483989.3 to 1277425.8) | 1743965.7 (1225915.7 to 2388073.9) | 76389.7 (47933.2 to 115880.8) | 46735.2 (31617.2 to 66986.7) | 24220.8 (16154.9 to 33507.9) | 576224.1 (388654.2 to 798520.1) | 553590.5 (371158.7 to 762674.5) | 22633.6 (14049.4 to 33861.8) | 21110.5 (14069.1 to 30327.9) | 12008.5 (8038.4 to 17016.7) | 376223.5 (255185.3 to 533432.8) | 368347.4 (248460.8 to 521833.0) | 7876.0 (4741.1 to 12490.2) | 14397.9 (7924.4 to 22233.8) | 3434.0 (2103.0 to 5132.1) | 177037.1 (106550.7 to 266324.2) | 171407.7 (103022.3 to 256057.9) | 5629.3 (2661.0 to 10779.8) |
| Albania | 664.1 (459.6 to 900.4) | 314.2 (218.2 to 411.5) | 5514.4 (3997.2 to 7159.9) | 5136.2 (3664.6 to 6706.1) | 378.2 (223.2 to 532.6) | 19.9 (12.5 to 28.8) | 10.6 (6.3 to 15.2) | 205.4 (127.5 to 291.5) | 193.3 (119.1 to 275.1) | 12 (7.2 to 18.6) | 129.2 (86.3 to 207.5) | 69.9 (49.1 to 110.2) | 1627.1 (1106.2 to 2559.8) | 1578.6 (1086.7 to 2476.1) | 48.5 (26.7 to 89.5) | 60.1 (34.3 to 95.4) | 6 (3.8 to 9.1) | 268.8 (165.6 to 411.9) | 240.3 (147 to 359.4) | 28.5 (13.3 to 57.1) |
| Algeria | 1103.1 (527.8 to 1799.8) | 485.9 (233.8 to 775) | 9004 (4373.1 to 14379.5) | 8396.3 (4100.5 to 13484.2) | 607.7 (298.3 to 1087.1) | 1228.8 (669.3 to 1917.9) | 551.6 (288.1 to 919.4) | 12185.7 (6483.8 to 19945.1) | 11586.3 (6032.9 to 18978.9) | 599.4 (311.2 to 1026.4) | 260.1 (144.1 to 414.8) | 124.9 (65.1 to 200.7) | 3544.1 (1930.4 to 5608.7) | 3443.2 (1868.5 to 5454.4) | 100.8 (51 to 175.2) | 668.9 (353.2 to 1156.3) | 62.2 (38 to 95.5) | 3460 (2052.6 to 5364.5) | 3132.3 (1874.1 to 4809) | 327.7 (132.1 to 676.6) |
| American Samoa | 13.8 (9.2 to 19.8) | 8.5 (5.7 to 11.9) | 170.6 (112.9 to 238.1) | 163 (108.1 to 227) | 7.6 (4.3 to 11.9) | 1.3 (0.8 to 1.9) | 0.5 (0.3 to 0.8) | 14.5 (8.8 to 21.9) | 13.8 (8.4 to 20.8) | 0.7 (0.4 to 1.1) | 0.6 (0.4 to 0.9) | 0.3 (0.2 to 0.4) | 8.8 (5.7 to 13.3) | 8.6 (5.6 to 13) | 0.2 (0.1 to 0.4) | 0 (0 to 0) | 0 (0 to 0) | 0.2 (0.1 to 0.3) | 0.2 (0.1 to 0.3) | 0 (0 to 0) |
| Andorra | 54.6 (35.1 to 78.9) | 14.6 (10 to 20.2) | 236.2 (161.5 to 329.7) | 200.7 (138.5 to 277.6) | 35.5 (20.5 to 54.9) | 16.6 (10.8 to 24.1) | 6.4 (4 to 9.4) | 114.3 (73.9 to 166.8) | 105.8 (68.4 to 153.7) | 8.5 (4.7 to 13.3) | 6.6 (4.2 to 10.6) | 2.6 (1.7 to 4.1) | 58.5 (38.3 to 90.5) | 55.8 (37 to 86.6) | 2.7 (1.4 to 4.8) | 10.7 (5.6 to 17.4) | 0.3 (0.2 to 0.4) | 16.5 (9.4 to 25.7) | 11.3 (6.6 to 17.2) | 5.2 (2.6 to 9.5) |
| Angola | 2815.5 (1882.5 to 3920.2) | 1999.4 (1361.2 to 2788.9) | 45616.3 (31347.8 to 63319.3) | 44171.4 (30473.3 to 61507.2) | 1444.9 (853.9 to 2175.8) | 330.1 (211 to 456.8) | 192.8 (123.8 to 270.1) | 5042.8 (3190 to 7086.1) | 4898 (3085.8 to 6966.3) | 144.8 (85.8 to 233.2) | 144.3 (82 to 218.6) | 79.2 (45.6 to 122) | 3382.9 (1841.4 to 5275) | 3330 (1807.6 to 5196.2) | 52.9 (27 to 91.7) | 100.3 (50.6 to 162.1) | 37.1 (19.9 to 58.8) | 2084 (1099.5 to 3365.9) | 2056.4 (1085.2 to 3309.7) | 27.6 (12.7 to 48) |
| Antigua and Barbuda | 76.8 (56.3 to 104.3) | 28.7 (23.8 to 34.7) | 556.1 (452.8 to 673.5) | 508.5 (419.6 to 611.8) | 47.7 (30.8 to 67.5) | 2.8 (2.1 to 3.4) | 1.3 (1 to 1.6) | 27.7 (22.5 to 33.9) | 26.3 (21.1 to 32.4) | 1.4 (0.9 to 2.1) | 1.8 (1.4 to 2.2) | 0.8 (0.7 to 1) | 23.7 (19 to 28.5) | 22.9 (18.3 to 27.7) | 0.7 (0.5 to 1) | 0.6 (0.4 to 0.8) | 0.1 (0.1 to 0.1) | 4.1 (3.2 to 5) | 3.8 (3 to 4.6) | 0.3 (0.1 to 0.5) |
| Argentina | 10993.9 (8974.7 to 13428) | 5747.6 (4938 to 6528.7) | 100398.2 (87087.2 to 113124.8) | 93629.4 (80873.1 to 105937.5) | 6768.8 (4595.7 to 9144.1) | 2840.2 (2470.2 to 3332.2) | 1435.8 (1266.9 to 1627.8) | 28298.4 (24954.2 to 32030.6) | 26964 (23695.3 to 30563.8) | 1334.4 (878.9 to 1844.2) | 3571.1 (3160.4 to 4006.1) | 2013 (1841.4 to 2185.5) | 51380.2 (47115.4 to 55973.1) | 50091.8 (45850.7 to 54626.9) | 1288.4 (904.4 to 1709.9) | 2836.8 (1979.1 to 3831.9) | 301.3 (268.2 to 340) | 16452.9 (14323.5 to 18814.8) | 15010.5 (13327.2 to 17006.2) | 1442.3 (798.9 to 2340.5) |
| Armenia | 484.4 (382.5 to 614) | 227.2 (196.2 to 263.8) | 4386 (3780.6 to 5069.1) | 4081.2 (3530.3 to 4713.4) | 304.8 (208.7 to 417.2) | 266.5 (215.2 to 326.8) | 141.3 (118.6 to 165.8) | 2999.8 (2512.8 to 3543.8) | 2863.1 (2406.3 to 3388.7) | 136.7 (90.1 to 187.2) | 125.3 (103 to 151.4) | 69.4 (58.2 to 80.7) | 1772.4 (1474.6 to 2068.7) | 1724 (1435.7 to 2010.2) | 48.4 (32.1 to 68.2) | 41 (27.7 to 57.3) | 8.5 (6.9 to 10.4) | 282.7 (226.3 to 343.1) | 264 (214.9 to 322) | 18.8 (9.9 to 30.2) |
| Australia | 23212.4 (19855.5 to 26839.3) | 4868.1 (4305.3 to 5375.4) | 83853.2 (74036.2 to 93058.1) | 68094.3 (60988.1 to 74985.8) | 15758.9 (11561.2 to 21100.1) | 2742.2 (2421.3 to 3062) | 1085.4 (980.1 to 1174.6) | 17395.3 (15843.8 to 18822.8) | 16017.9 (14750.5 to 17295) | 1377.5 (981.7 to 1872.8) | 2784.4 (2387.2 to 3166.6) | 988.8 (894.5 to 1086.8) | 20457.2 (18444.6 to 22537.3) | 19282.6 (17477.8 to 21141) | 1174.6 (829.7 to 1632.8) | 1272.1 (933.2 to 1668.7) | 42.3 (35.3 to 51.6) | 2288 (1779.8 to 2905.6) | 1613.7 (1327.7 to 1980.2) | 674.3 (395.3 to 1101.4) |
| Austria | 5959.8 (4654.8 to 7539.3) | 1726 (1505.3 to 1909.8) | 28971.2 (24954.2 to 32402.8) | 24963.1 (21797.7 to 27716.2) | 4008.1 (2678.2 to 5504.9) | 1331.5 (1123.3 to 1569.1) | 557.5 (492.4 to 619.5) | 9420.5 (8320.9 to 10546.4) | 8758.2 (7742.4 to 9759.4) | 662.3 (470.9 to 890.6) | 867.2 (727.4 to 1026.9) | 404.6 (356.6 to 461.9) | 8035.5 (7138.3 to 9195) | 7699.5 (6844 to 8820.7) | 336 (227.7 to 467.7) | 504 (343.3 to 664.5) | 20.1 (16.8 to 24.3) | 981.4 (786.3 to 1243.6) | 710.6 (584.7 to 877.6) | 270.8 (152.5 to 430) |
| Azerbaijan | 772.6 (546.7 to 1033.8) | 372.6 (275.3 to 492.5) | 8699.8 (6460.8 to 11483) | 8221.8 (6186.9 to 10841.2) | 478 (312 to 715.6) | 325.7 (214.8 to 464.6) | 164.8 (107.7 to 235.8) | 4268.6 (2789.8 to 6033.5) | 4104.9 (2682.8 to 5840.2) | 163.7 (99.3 to 250.9) | 366.9 (252.9 to 531.7) | 205.8 (135.5 to 303) | 6299.3 (4143.7 to 8993.7) | 6162.2 (4034 to 8827) | 137.1 (79.8 to 238.7) | 50.6 (27.7 to 86.7) | 12.5 (7.4 to 19) | 511.4 (296.5 to 805.2) | 490.9 (281.6 to 767.5) | 20.5 (8.1 to 38) |
| Bahamas | 273.8 (214.7 to 354.5) | 117.1 (98.8 to 136.1) | 2275.8 (1913.1 to 2674.1) | 2117.9 (1786.5 to 2471.2) | 157.9 (108.8 to 228.2) | 10.7 (8.6 to 13.3) | 5 (4.1 to 6.1) | 115.1 (93.8 to 141.7) | 109.7 (90.1 to 135) | 5.4 (3.5 to 7.7) | 11.6 (9.6 to 14.4) | 5.4 (4.6 to 6.5) | 174.3 (147.1 to 207.1) | 169.6 (143.2 to 202.7) | 4.7 (3.2 to 6.7) | 0.8 (0.6 to 1.1) | 0.1 (0.1 to 0.1) | 6.4 (5 to 7.8) | 6 (4.7 to 7.5) | 0.4 (0.2 to 0.6) |
| Bahrain | 163.4 (104.3 to 235.1) | 45.4 (31.1 to 60) | 1043.5 (711.5 to 1396.3) | 948.3 (644.9 to 1253) | 95.2 (54.9 to 152.4) | 88.9 (59.6 to 138.4) | 29.7 (19.4 to 44.1) | 822.3 (534.2 to 1193.1) | 773.7 (501.1 to 1138) | 48.5 (28.9 to 76.4) | 26.5 (17.2 to 40.1) | 10.5 (6.4 to 15.2) | 337 (214.9 to 487.9) | 326 (206.9 to 467.8) | 11 (6 to 20) | 7.5 (4.1 to 12.2) | 0.5 (0.3 to 0.8) | 30.4 (18.1 to 44.6) | 26.6 (15.9 to 39.2) | 3.9 (1.7 to 7.2) |
| Bangladesh | 7535.5 (4485.8 to 13167.2) | 4531.3 (2764.5 to 7915.5) | 90951.9 (54886.8 to 158237.7) | 86987 (52675.4 to 150490.8) | 3964.9 (1940.7 to 6849.9) | 2993.6 (1669.4 to 5473.7) | 1578.8 (809.3 to 2722.1) | 38102.6 (20163.7 to 66426.6) | 36685.7 (19430.3 to 63946.8) | 1416.9 (765.6 to 2427.9) | 1205.2 (665.8 to 2195.2) | 736.4 (416 to 1328.3) | 21489.3 (12023.8 to 39318.9) | 21050.7 (11851.4 to 38313.4) | 438.6 (206.8 to 825.1) | 1024.8 (527.4 to 1814.4) | 222.3 (123 to 360.9) | 11616 (6498.9 to 18707) | 11179.1 (6136.5 to 18050.5) | 437 (173.3 to 958) |
| Barbados | 365.2 (276.9 to 480.6) | 141.3 (116.7 to 171.3) | 2528.5 (2119.7 to 3051.4) | 2317.1 (1942.7 to 2830.1) | 211.4 (141.5 to 295.5) | 19.1 (15.2 to 23.2) | 9.3 (7.7 to 11.3) | 183.3 (149.5 to 222.2) | 174.2 (143.2 to 212.7) | 9.1 (5.8 to 12.8) | 17.4 (14 to 22) | 8.7 (7.2 to 10.7) | 220.2 (183 to 267.5) | 213.4 (178.3 to 262) | 6.7 (4.5 to 9.5) | 1.4 (1 to 1.9) | 0.2 (0.2 to 0.3) | 8.6 (6.9 to 10.5) | 7.9 (6.5 to 9.7) | 0.7 (0.4 to 1.1) |
| Belarus | 5705.9 (4420.4 to 7220.4) | 1190.7 (1095.6 to 1298.4) | 27531.5 (25050.3 to 30546) | 23644.9 (21705.6 to 26172) | 3886.6 (2629.5 to 5581.4) | 834.4 (706.8 to 971.5) | 360.2 (317.6 to 410.4) | 8252.4 (7207.3 to 9341.6) | 7831.7 (6921.2 to 8950.4) | 420.7 (283.4 to 560.4) | 970.1 (825.4 to 1165.1) | 382.7 (335.6 to 439.3) | 10552.6 (9297 to 12097.6) | 10160.5 (8940.5 to 11671.7) | 392.1 (269.2 to 560.1) | 187.7 (123.4 to 256.3) | 21.1 (17.3 to 25.2) | 836 (677.2 to 1004.7) | 740.5 (604.3 to 883.7) | 95.5 (52.5 to 150.9) |
| Belgium | 6545.1 (4973.7 to 8185.9) | 2129.7 (1860.2 to 2361.8) | 34218.2 (29600.7 to 38270.4) | 29853.7 (26240 to 33189.6) | 4364.5 (2867.4 to 6031.1) | 1945.8 (1648.4 to 2329.8) | 836.4 (743.2 to 956.5) | 14142.7 (12514 to 16170.1) | 13163.5 (11747.6 to 15023.7) | 979.1 (690.5 to 1374.6) | 1059.6 (920.4 to 1200.2) | 502.9 (461.9 to 533.5) | 10177.6 (9465.2 to 10781.8) | 9767.7 (9081.3 to 10289.6) | 409.8 (283.4 to 573.7) | 320.6 (216.6 to 433.2) | 15 (12.6 to 17.8) | 675.2 (530.4 to 845.1) | 504.9 (424.7 to 606.5) | 170.3 (91.2 to 289.4) |
| Belize | 96.1 (76 to 124.5) | 45.5 (38.8 to 54.4) | 848.5 (718.6 to 1006.3) | 795.3 (678.4 to 943.6) | 53.2 (35.4 to 77.2) | 6.5 (5.1 to 8.1) | 3.2 (2.6 to 3.9) | 72.1 (58.4 to 88.3) | 68.8 (55.8 to 84.2) | 3.3 (2.2 to 4.7) | 6.8 (5.4 to 8.3) | 3.2 (2.6 to 3.9) | 105.4 (85.2 to 130.2) | 102.6 (83.3 to 126.8) | 2.8 (1.9 to 4.1) | 3.1 (2.2 to 4.2) | 0.5 (0.4 to 0.6) | 26.5 (21.1 to 33.8) | 25.1 (20.1 to 31.8) | 1.4 (0.7 to 2.6) |
| Benin | 1031.4 (687.5 to 1511.5) | 786.3 (514.1 to 1163.6) | 15824 (10539.7 to 23321.6) | 15326.9 (10177.8 to 22648.3) | 497.1 (300.3 to 802.4) | 70.7 (45.4 to 105.9) | 42.2 (27.2 to 64.6) | 1048.3 (674.2 to 1585.5) | 1015.4 (652.5 to 1529.8) | 32.9 (18.6 to 53.3) | 144.8 (86.8 to 220.3) | 70.4 (43.6 to 106.2) | 3296.5 (2021.2 to 4910.3) | 3239.7 (1990.5 to 4817) | 56.8 (28.3 to 96.5) | 35.1 (19 to 56.4) | 11.9 (6.9 to 18.5) | 658.4 (375.6 to 1016.6) | 647.8 (369.4 to 1005) | 10.6 (4.7 to 18.3) |
| Bermuda | 111 (82.2 to 147.3) | 26.2 (22.4 to 30.7) | 462.6 (385.3 to 555.8) | 394.4 (333.5 to 463.5) | 68.2 (45.3 to 98.9) | 12.7 (9.8 to 15.5) | 4.9 (4 to 6) | 93.1 (75.4 to 112.2) | 86.6 (70 to 104.3) | 6.5 (4.3 to 9.2) | 4.8 (3.8 to 5.9) | 1.8 (1.4 to 2.1) | 43.5 (34.6 to 52) | 41.4 (33.1 to 50) | 2.1 (1.4 to 3) | 0.9 (0.6 to 1.2) | 0.1 (0 to 0.1) | 2.3 (1.8 to 2.8) | 1.9 (1.6 to 2.2) | 0.4 (0.2 to 0.7) |
| Bhutan | 20 (11.4 to 31.8) | 14.2 (7.9 to 22.5) | 260.7 (146.4 to 405.6) | 250.9 (139.9 to 389.9) | 9.8 (4.6 to 17.1) | 7.2 (3.7 to 12.3) | 4.2 (2.1 to 7.5) | 94.4 (46.7 to 161.2) | 91.2 (44.5 to 156.1) | 3.3 (1.8 to 5.6) | 4.5 (2.4 to 8) | 2.9 (1.6 to 5) | 82.7 (43.8 to 143.7) | 81.2 (43.2 to 139.9) | 1.5 (0.7 to 3.1) | 4.2 (2.2 to 8.2) | 1 (0.5 to 1.8) | 54.3 (29.2 to 97.7) | 52.8 (28.1 to 94.5) | 1.5 (0.5 to 3.2) |
| Bolivia (Plurinational State of) | 3632.1 (2446.3 to 5187.7) | 1409.2 (991.6 to 1905.2) | 28465.9 (19891.6 to 39081.2) | 26493.9 (18532.9 to 36202.2) | 1972 (1141.7 to 3064.9) | 318.8 (197.6 to 508.7) | 129.4 (80.3 to 200.2) | 2936.6 (1794.5 to 4528.4) | 2783 (1707.5 to 4275) | 153.6 (83.9 to 244.1) | 308.8 (197.2 to 469.8) | 134.5 (87.3 to 201.2) | 4056.9 (2638.3 to 6056.9) | 3936.8 (2583.6 to 5915.3) | 120.2 (65.7 to 191.7) | 166.3 (88 to 264.3) | 43.2 (24.9 to 66.2) | 2013.2 (1153.2 to 3084.5) | 1947.6 (1123 to 2982.3) | 65.6 (29.4 to 123.2) |
| Bosnia and Herzegovina | 926.3 (621.2 to 1253.1) | 423.1 (274.4 to 558.2) | 7853.8 (5165.4 to 10286.3) | 7323.9 (4784.8 to 9563.6) | 529.9 (317 to 824.4) | 414.9 (310.4 to 540.9) | 214.6 (165.5 to 272) | 4372.4 (3396.1 to 5568.6) | 4185.7 (3252.8 to 5279.6) | 186.7 (117.4 to 266.1) | 219 (134.3 to 296.7) | 119.9 (69.9 to 164) | 2889.2 (1726.3 to 3890.7) | 2810.2 (1676.8 to 3762.8) | 79 (43.6 to 125.5) | 91.6 (54 to 145.2) | 11.1 (7.6 to 15.1) | 469.2 (316.9 to 669.2) | 423 (288.7 to 592.2) | 46.3 (23 to 81.3) |
| Botswana | 101.1 (67.9 to 149.3) | 71.9 (49.4 to 105.8) | 1379.2 (940.4 to 2041.7) | 1333.9 (903.2 to 1978.1) | 45.3 (27.6 to 70.3) | 8.2 (5 to 12.9) | 4.7 (2.9 to 7.3) | 111.5 (68.9 to 176.7) | 108.2 (66.8 to 171.5) | 3.3 (1.9 to 5.5) | 7.7 (4.6 to 11.4) | 4.7 (2.8 to 6.9) | 152.4 (90.7 to 225.7) | 150.2 (89.6 to 222.4) | 2.2 (1.2 to 3.6) | 12.8 (6.2 to 21.7) | 3.5 (1.8 to 5.4) | 186.8 (96.4 to 289.9) | 181.9 (94.2 to 284.6) | 4.9 (1.9 to 9.4) |
| Brazil | 52602.7 (42765.2 to 65145.6) | 22024.9 (19651.7 to 24061.7) | 408779 (371271.8 to 445107.3) | 380942.4 (345487.7 to 413568.7) | 27836.6 (18937.3 to 38034.3) | 7919.6 (6952.7 to 8877.3) | 4166.1 (3817.8 to 4465.4) | 82556.7 (77194.2 to 88120.6) | 79083.9 (73855.7 to 83988.7) | 3472.8 (2461.5 to 4608.5) | 6461.3 (5767.8 to 7256.2) | 3437.2 (3210.1 to 3670) | 91118.1 (85774.7 to 96640.1) | 88810 (83694.1 to 94245) | 2308.1 (1629.6 to 3069.2) | 4037.7 (3009.3 to 5380.8) | 590.7 (526.8 to 665.1) | 30348.8 (27080.8 to 33979.5) | 28538.4 (25412.3 to 31791.5) | 1810.4 (1075.6 to 2803.3) |
| Brunei Darussalam | 29.5 (20.7 to 40.4) | 15.8 (11.5 to 20.8) | 307.1 (225.2 to 405.4) | 288.7 (212.9 to 381.2) | 18.5 (10.7 to 27.4) | 12.6 (8.6 to 17.8) | 4.8 (3.3 to 6.8) | 113 (76.4 to 157.4) | 106.1 (72.6 to 147.9) | 6.9 (4 to 10.5) | 12.4 (8.3 to 18.5) | 6.8 (4.7 to 10.1) | 195 (133.3 to 294.5) | 190.3 (130.9 to 287.8) | 4.7 (2.7 to 7.9) | 5.5 (3 to 9.4) | 0.7 (0.4 to 1) | 37.8 (22.3 to 57.2) | 35 (20.7 to 53.8) | 2.8 (1.2 to 5.1) |
| Bulgaria | 3222.9 (2566.2 to 3943.1) | 1140.2 (1038.4 to 1257.5) | 21591.2 (19419.8 to 24033.9) | 19472.5 (17686.2 to 21539.4) | 2118.6 (1450.7 to 2852.8) | 1067.8 (935 to 1222.6) | 496.3 (454 to 546.6) | 10359.8 (9413.3 to 11423.4) | 9845.3 (8933.6 to 10853.4) | 514.5 (372.4 to 693.6) | 569.6 (468.1 to 673.2) | 269.8 (235.4 to 305.2) | 6807 (5913.5 to 7686.7) | 6586.1 (5732.9 to 7447.5) | 220.9 (149 to 299.9) | 511.8 (366.9 to 684.3) | 47.2 (39.4 to 56.9) | 2116.1 (1749.9 to 2545.1) | 1846.1 (1526.6 to 2215.8) | 270 (144.6 to 436.5) |
| Cabo Verde | 95.2 (61 to 134.5) | 61.6 (37.7 to 92.8) | 1091.4 (699 to 1579) | 1041.6 (667.3 to 1520.4) | 49.8 (29.5 to 80.1) | 14.3 (8.5 to 21.1) | 6.6 (3.9 to 9.8) | 168.3 (100.9 to 246.4) | 162.1 (97.1 to 238.2) | 6.1 (3.7 to 9.8) | 4.4 (1.2 to 8.7) | 2.2 (0.6 to 4.3) | 75.5 (20.3 to 146.6) | 74.1 (19.9 to 144.1) | 1.4 (0.3 to 3) | 0.5 (0.2 to 0.9) | 0.2 (0.1 to 0.2) | 5.2 (2.5 to 9) | 5.1 (2.4 to 8.7) | 0.2 (0.1 to 0.3) |
| Cambodia | 499.4 (305.4 to 766.8) | 358.5 (222.2 to 561.3) | 6950.2 (4384.8 to 10454) | 6679 (4214.8 to 10103.5) | 271.3 (159.2 to 442.7) | 165.1 (94.9 to 266.2) | 79.5 (44.9 to 127.6) | 2012.8 (1132.3 to 3185.2) | 1926 (1075.2 to 3046.1) | 86.7 (44.9 to 145.1) | 121.8 (71.9 to 186.3) | 61.9 (36.4 to 93.1) | 2075.2 (1198.3 to 3137.3) | 2026.7 (1162.9 to 3069.4) | 48.5 (25.3 to 79.7) | 44.1 (24.8 to 73) | 15.3 (8.9 to 23.7) | 746.1 (433.4 to 1160.5) | 732.3 (425.1 to 1139.5) | 13.8 (6.2 to 27.8) |
| Cameroon | 2226.8 (1292.1 to 3418) | 1649.6 (896.2 to 2566.5) | 34061.9 (18987.9 to 52460.3) | 32991.2 (18361 to 50806.2) | 1070.7 (584.1 to 1748.5) | 429.1 (269.2 to 682.8) | 244.4 (149.3 to 385.9) | 6289.8 (3851.8 to 9810.4) | 6103.9 (3712.3 to 9514.1) | 185.9 (102.3 to 311.2) | 220.9 (132.2 to 326.6) | 107 (60.6 to 167.2) | 4991 (2987 to 7475.6) | 4912.2 (2937.2 to 7365.2) | 78.8 (38.9 to 131.2) | 81.6 (42.7 to 129.1) | 27.1 (14.6 to 43.3) | 1523.2 (812.9 to 2431.5) | 1499.1 (804.3 to 2393.7) | 24.1 (10.7 to 43.8) |
| Canada | 21697.6 (17113.7 to 26960.7) | 6437.5 (5614.8 to 7177.3) | 108039.3 (94173.3 to 120385.1) | 93899.6 (82724 to 104138.9) | 14139.8 (9516.4 to 19710.6) | 4888.3 (4182.1 to 5769) | 2218.9 (1998.7 to 2422.8) | 36226.3 (32681.5 to 39840.5) | 33915.2 (30918.9 to 36957) | 2311.2 (1624.3 to 3234) | 5301.8 (4235.7 to 6456.3) | 1597.2 (1375.4 to 1826.1) | 34815.1 (30238.4 to 39991.1) | 32499.5 (27947.7 to 37313.7) | 2315.6 (1516.3 to 3175.2) | 1686.5 (1221.3 to 2275.7) | 56.8 (48.2 to 69) | 3191.2 (2554.9 to 4040.8) | 2282.2 (1927 to 2760.5) | 908.9 (499.3 to 1477.6) |
| Chile | 6257.4 (5184.1 to 7615) | 2764.7 (2528 to 2936.1) | 46126.6 (42704.5 to 48855) | 42138.8 (39213.4 to 44421.5) | 3987.9 (2717.3 to 5529) | 937 (818.1 to 1070.7) | 423.2 (386.5 to 469.1) | 8193.5 (7536.7 to 8985.2) | 7711.4 (7104.9 to 8490.3) | 482.1 (341.6 to 652.7) | 1693.9 (1482.6 to 1904.5) | 839.2 (782.4 to 901.9) | 20826.6 (19267.2 to 22350.4) | 20166.7 (18702.7 to 21696) | 659.9 (459.1 to 898.4) | 1816.5 (1206.7 to 2584) | 127.2 (109.4 to 149.2) | 6892.4 (5754.5 to 8296.5) | 5930.1 (5088.8 to 6948.9) | 962.2 (560.4 to 1578.1) |
| China | 92855.3 (64005.3 to 121349.2) | 36820.4 (25594.7 to 46000.1) | 668679.6 (490032.1 to 809422.4) | 611689.8 (448238.9 to 740213.7) | 56989.8 (35430.7 to 85233.5) | 85985.4 (69594.2 to 104250.1) | 34990.2 (30077.6 to 40939.9) | 719937.3 (620915.1 to 842352.7) | 674094.7 (574095.5 to 785312.2) | 45842.6 (30486.8 to 63861.6) | 41120.4 (29821.1 to 58781.3) | 14994.4 (10584.4 to 21308.6) | 412158.7 (295668.5 to 579618.9) | 394028.8 (279089.6 to 558455.1) | 18129.9 (11068.1 to 30276.5) | 5244.7 (3752.8 to 7330.8) | 1070 (853.8 to 1331) | 34895.6 (27706.9 to 43681.1) | 32518.9 (25783.4 to 40779.1) | 2376.7 (1367.8 to 3824) |
| Colombia | 14240.8 (11231.8 to 17727.3) | 4101.9 (3676.7 to 4514.3) | 75949.4 (68029.1 to 84623.2) | 67689.3 (61236.9 to 74059.6) | 8260.1 (5428.4 to 11853.7) | 963.3 (832.7 to 1101.1) | 448.2 (404.9 to 492.1) | 8903.5 (8117.3 to 9690.1) | 8411.6 (7652.9 to 9170.4) | 492 (351.6 to 661.8) | 1171.2 (1011.1 to 1325.1) | 535.7 (491 to 577.1) | 14278.3 (13229.8 to 15290) | 13793.2 (12782.7 to 14823.5) | 485.1 (335.1 to 673.1) | 1875.4 (1358.2 to 2457) | 167.9 (152.6 to 185.7) | 9558.8 (8444.7 to 10934.3) | 8560.7 (7655 to 9519.2) | 998.1 (571.9 to 1586.3) |
| Comoros | 32.3 (19.9 to 49) | 28.1 (17.7 to 42.8) | 496.9 (316.3 to 736.6) | 481.3 (307.4 to 716.5) | 15.6 (9.1 to 25.5) | 5.5 (3.2 to 8.3) | 3.8 (2.2 to 5.8) | 81.1 (48.4 to 121.1) | 78.5 (46.6 to 117.5) | 2.6 (1.5 to 4) | 3.9 (2.1 to 6) | 2.4 (1.3 to 3.8) | 83.4 (46.7 to 130.4) | 82.1 (45.9 to 128.6) | 1.4 (0.7 to 2.1) | 1 (0.6 to 1.6) | 0.4 (0.2 to 0.6) | 20.3 (11.3 to 31.1) | 20 (11.1 to 30.7) | 0.3 (0.2 to 0.6) |
| Congo | 616.3 (416.3 to 831.8) | 429.5 (294.9 to 611.7) | 9642.4 (6604.3 to 13671.8) | 9336.9 (6401.8 to 13190) | 305.5 (180 to 452.2) | 75 (46.6 to 107.3) | 44.4 (26.8 to 65.6) | 1117.9 (673.7 to 1631.8) | 1087.7 (654.2 to 1596.9) | 30.2 (16.2 to 50.2) | 24.8 (16.2 to 36) | 14.5 (9.7 to 20.9) | 558.1 (363.1 to 802.5) | 550.1 (358.4 to 790.1) | 8 (4.4 to 13.7) | 15.8 (8.3 to 26) | 5.9 (3.1 to 9.5) | 303.7 (157.2 to 500.2) | 299.7 (154.8 to 492.4) | 4 (1.8 to 7.7) |
| Costa Rica | 2647.2 (2099.5 to 3362.2) | 699.7 (618.3 to 779.9) | 12646.3 (11250.7 to 13995.6) | 11055.9 (9946.9 to 12283) | 1590.5 (1040.3 to 2254.3) | 201.8 (162.2 to 249.4) | 87.6 (72.8 to 104.7) | 1755.9 (1456.6 to 2083.3) | 1652 (1367.8 to 1968.2) | 103.9 (74.2 to 145) | 191.8 (153.2 to 236) | 82.7 (66.9 to 98.4) | 2156.4 (1740.5 to 2575.8) | 2076.5 (1674.8 to 2471.4) | 79.8 (53.1 to 110.6) | 304.9 (212.1 to 415.8) | 23.4 (19.3 to 28.4) | 1274.2 (1037.2 to 1581.9) | 1121.1 (923 to 1384.1) | 153 (86 to 257.4) |
| C么te d'Ivoire | 2940.2 (1924.6 to 4055.9) | 2144.6 (1423.3 to 2979.3) | 45704.7 (30396.4 to 63356.8) | 44227 (29311.1 to 61396.1) | 1477.7 (864 to 2191.8) | 323 (197.4 to 488.1) | 178.8 (110.4 to 272.6) | 4803.4 (2926.8 to 7136.6) | 4653.6 (2840.7 to 6902) | 149.8 (82.3 to 249.9) | 124.5 (79.7 to 184.9) | 59.9 (36.7 to 88.9) | 2877.3 (1837.8 to 4188.2) | 2830.4 (1791.2 to 4123.3) | 46.9 (22.9 to 80) | 49.4 (25.5 to 82.9) | 16.2 (9.1 to 24.9) | 938.3 (522.3 to 1450.4) | 924.5 (515.1 to 1424.6) | 13.8 (6.3 to 26.2) |
| Croatia | 2198 (1777.2 to 2804.1) | 875.5 (803 to 949) | 15136.4 (13763.4 to 16804.1) | 13821.8 (12571 to 15121.7) | 1314.6 (881.7 to 1807.4) | 732.2 (615.1 to 852.1) | 357.8 (316.1 to 403.1) | 6710.4 (5893.5 to 7614.3) | 6364.8 (5595.7 to 7223) | 345.6 (233.2 to 466.7) | 482.8 (414.1 to 561.3) | 239.7 (213.5 to 265.2) | 5313.8 (4680.2 to 5969.6) | 5134.4 (4536.4 to 5755.6) | 179.4 (120.5 to 242.8) | 156.3 (100.7 to 214.8) | 12.5 (10 to 15.2) | 517.9 (405.4 to 643) | 436 (350.5 to 528.6) | 82 (45.8 to 136.4) |
| Cuba | 10619.6 (8103.3 to 13488.3) | 3553.7 (3017 to 4177.9) | 63007.8 (53952.1 to 71703.4) | 57053.9 (49177.1 to 66125.5) | 5953.9 (4010.4 to 8486.4) | 1233.5 (1001.1 to 1495.9) | 559 (478.9 to 653.8) | 10959.5 (9466.8 to 12788.9) | 10377 (8976.2 to 12152.7) | 582.5 (403.8 to 814.2) | 619.4 (496.4 to 761.2) | 289.4 (239.4 to 345.5) | 7141.9 (5947.1 to 8481.5) | 6902.5 (5751.4 to 8229) | 239.4 (164 to 338) | 156 (108.2 to 209.4) | 22.6 (18.1 to 27.7) | 796.7 (631.4 to 979.2) | 719.4 (574.4 to 886) | 77.3 (39.7 to 130.1) |
| Cyprus | 718.3 (475.5 to 1030) | 226.5 (168 to 296.8) | 3719.7 (2678.1 to 4971.4) | 3242.9 (2347.6 to 4331.9) | 476.8 (273.8 to 702.9) | 199.8 (134.2 to 289.7) | 82.8 (55.5 to 119.4) | 1441.9 (983.7 to 2082) | 1342.5 (920.7 to 1961.8) | 99.4 (58.4 to 148.1) | 70.7 (47.3 to 107) | 31 (20.1 to 45) | 681.2 (443.1 to 984.5) | 652.5 (424.8 to 947.8) | 28.6 (16.8 to 48.6) | 56.9 (29.7 to 92.6) | 2.1 (1.2 to 3) | 109.3 (62.5 to 164.1) | 80.9 (46.9 to 121.1) | 28.5 (13.5 to 53.4) |
| Czechia | 5807.9 (5049.5 to 6717.4) | 1787.6 (1669.5 to 1922.8) | 32919.5 (30499.4 to 35713) | 29218.6 (27303.6 to 31527.3) | 3700.9 (2609.1 to 5156.3) | 2266.8 (1936.2 to 2634.3) | 738.6 (667.3 to 812.6) | 14292.6 (12990.7 to 15796.2) | 13046.1 (11880.9 to 14290) | 1246.5 (884.3 to 1702.2) | 1760.3 (1534.2 to 2036.6) | 771.3 (702.4 to 849) | 16933.5 (15535.2 to 18477.8) | 16250.4 (15017.6 to 17765.2) | 683.1 (471.9 to 916.3) | 669.9 (442.2 to 901.7) | 48.8 (40.9 to 58.2) | 2142.2 (1735 to 2609.6) | 1779.8 (1497.3 to 2130.9) | 362.3 (200.2 to 582.6) |
| Denmark | 5004.3 (4176.3 to 5860.8) | 1968.1 (1725.7 to 2177.2) | 31503.1 (27753.9 to 35236) | 28276.9 (25002.6 to 31371.4) | 3226.1 (2261.3 to 4400.1) | 1039.6 (852.3 to 1219.5) | 456.7 (392.8 to 522.3) | 7523.4 (6538.1 to 8659.3) | 7014.1 (6092.2 to 8053.1) | 509.2 (361.8 to 701.3) | 538.4 (440.5 to 656) | 271.9 (233.6 to 311.8) | 5558 (4770.8 to 6380.6) | 5354.7 (4599 to 6176) | 203.4 (131.9 to 286.2) | 262 (192.1 to 335.3) | 10.5 (8.7 to 12.7) | 477.3 (375.1 to 589.8) | 339.1 (277.4 to 412.1) | 138.1 (83.4 to 218.6) |
| Djibouti | 73.7 (47.7 to 108.2) | 51.3 (33.5 to 75.6) | 1159.2 (777.8 to 1693.3) | 1118.7 (746.7 to 1647.8) | 40.6 (23.5 to 62.7) | 17.4 (9.9 to 27.9) | 9.4 (5.3 to 14.9) | 267 (151.6 to 429) | 258.4 (146.7 to 416.5) | 8.6 (4.6 to 14.6) | 8.6 (5 to 13.6) | 5.1 (2.9 to 8.3) | 190.5 (107.5 to 301.8) | 187.3 (105.7 to 297.8) | 3.1 (1.7 to 5.2) | 7.2 (3.7 to 12.1) | 2.4 (1.3 to 3.9) | 133.3 (71.7 to 216.3) | 131.2 (70.3 to 213) | 2.1 (0.9 to 3.9) |
| Dominica | 68.7 (47.4 to 92.2) | 40.3 (28.9 to 54.1) | 688.6 (499.2 to 920.6) | 651.1 (471.2 to 873.4) | 37.5 (22.6 to 56.3) | 2.9 (1.9 to 4) | 1.7 (1.1 to 2.3) | 33 (22 to 44.9) | 31.7 (21.2 to 43) | 1.3 (0.8 to 2) | 2.1 (1.4 to 3.1) | 1.2 (0.8 to 1.8) | 34.4 (22.4 to 49.4) | 33.5 (21.8 to 48.1) | 0.8 (0.5 to 1.4) | 0.2 (0.1 to 0.3) | 0 (0 to 0.1) | 1.8 (1.1 to 3) | 1.7 (1 to 2.8) | 0.1 (0 to 0.1) |
| Dominican Republic | 4249.7 (2728.4 to 5779.4) | 2494.9 (1620.3 to 3447.6) | 43074.4 (27970.5 to 58293.6) | 41073.9 (26620.7 to 55890) | 2000.5 (1188.6 to 3159) | 181.4 (120.4 to 256.1) | 102 (69.8 to 145.3) | 2123.2 (1443.5 to 2982.4) | 2043.9 (1394 to 2873.7) | 79.3 (45.8 to 122.5) | 104.4 (64.8 to 166) | 55 (34.8 to 89) | 1731.8 (1081.2 to 2736.9) | 1692.8 (1060.4 to 2664.4) | 39 (20 to 65.9) | 48 (29.2 to 81.4) | 11.2 (7.6 to 17.6) | 529 (342.5 to 853.3) | 508.1 (332.4 to 822.8) | 20.9 (8.3 to 41.6) |
| Ecuador | 5033 (4029.5 to 6359.1) | 1692.5 (1515 to 1896.1) | 29406.6 (26456.1 to 32689.2) | 26515.9 (23999.5 to 29399.3) | 2890.7 (1979.9 to 4109.4) | 339.7 (281.8 to 412) | 130.2 (114.6 to 148.5) | 2561.9 (2242.4 to 2956.9) | 2375.7 (2081.8 to 2729.9) | 186.2 (128.8 to 269.5) | 453.5 (366.8 to 541.8) | 164.1 (138.4 to 190.1) | 4634.3 (3869.8 to 5461.3) | 4436.4 (3707.1 to 5213.6) | 197.9 (133.5 to 271.2) | 478.5 (338.4 to 641.5) | 63.2 (52.4 to 75.7) | 3345.5 (2762.5 to 4062.7) | 3108.8 (2581.9 to 3740.5) | 236.7 (130.4 to 384.5) |
| Egypt | 5012.1 (3119.3 to 7160.2) | 2359 (1422.4 to 3472.2) | 48549.1 (29606.9 to 70458) | 45643.6 (27834 to 66450) | 2905.5 (1740.4 to 4849.2) | 4957 (3139.4 to 7097.8) | 2215.3 (1456.8 to 3129.1) | 55000.7 (35964.5 to 78809.1) | 52644.3 (34445.3 to 75250.6) | 2356.4 (1384.9 to 3649.4) | 580.8 (371.3 to 833.4) | 288.8 (175.6 to 413.9) | 9048 (5772.9 to 12822.5) | 8825.5 (5619.3 to 12510.7) | 222.5 (131.6 to 386.9) | 353.5 (182.2 to 579.8) | 55 (32.7 to 81.4) | 2695 (1583.9 to 4077.4) | 2533.9 (1478.9 to 3847.7) | 161.1 (77.6 to 300.7) |
| El Salvador | 1884.5 (1265.4 to 2797.1) | 731.9 (548.2 to 1034.6) | 12943.2 (9716.5 to 18581.1) | 11887.1 (8943 to 17058) | 1056.1 (651.5 to 1605.9) | 89.2 (62.8 to 136) | 44.2 (32.1 to 63.6) | 922.6 (683 to 1357.2) | 879.3 (650.7 to 1281.4) | 43.3 (26.5 to 69.8) | 94.3 (60.2 to 138.7) | 46.5 (30.5 to 67.2) | 1333.8 (863.8 to 1906) | 1296.8 (835 to 1857.7) | 37.1 (22.3 to 60.7) | 96.6 (56.5 to 155.7) | 13.3 (8.7 to 20.2) | 715.2 (461.3 to 1105) | 664.6 (427.7 to 1024.2) | 50.7 (23.2 to 90.5) |
| Equatorial Guinea | 164.4 (110.4 to 239.2) | 98.6 (68.4 to 145) | 2265.3 (1549.9 to 3329.2) | 2188 (1499.9 to 3210.7) | 77.3 (45.9 to 125.5) | 24.4 (14.9 to 37.8) | 12.1 (7.1 to 18.9) | 332.1 (194 to 517) | 322.9 (189.1 to 505.9) | 9.2 (4.9 to 15.7) | 7.2 (4.1 to 11) | 3.6 (2 to 5.4) | 147.4 (81 to 228.6) | 145.4 (79.8 to 225.7) | 2 (1 to 3.4) | 12.9 (5.8 to 23) | 3.2 (1.6 to 5.5) | 187.9 (91.6 to 323.3) | 184.1 (88.7 to 316.3) | 3.8 (1.4 to 8.2) |
| Estonia | 1275.1 (999.2 to 1610.8) | 311.9 (278.2 to 346.3) | 6006.3 (5411 to 6705.9) | 5172.9 (4626.1 to 5700.2) | 833.5 (568 to 1170.4) | 172.9 (142.7 to 207) | 87.2 (74.1 to 100.8) | 1595.8 (1356.9 to 1842) | 1515.1 (1290.4 to 1760.3) | 80.6 (56.6 to 109.4) | 195.4 (158.2 to 241.8) | 83.6 (69.1 to 98.6) | 1906.7 (1581.6 to 2241.1) | 1830.2 (1528.2 to 2166.8) | 76.5 (50.5 to 105.7) | 23.3 (16.2 to 31.4) | 2.7 (2.2 to 3.3) | 93.2 (75.2 to 115.6) | 81.4 (66.9 to 98.2) | 11.8 (5.9 to 20.4) |
| Eswatini | 73.4 (48.2 to 105.4) | 56.5 (37.5 to 81.8) | 1194.6 (787.4 to 1771.8) | 1150.4 (759.2 to 1702.8) | 44.2 (24.6 to 68.4) | 5.5 (3.5 to 8.3) | 3.2 (2 to 4.9) | 86.6 (54.5 to 136.6) | 83.4 (52.3 to 131.3) | 3.2 (1.8 to 5.3) | 6.3 (3.4 to 9.8) | 4.1 (2.1 to 6.6) | 144.6 (77.7 to 224.4) | 142.5 (76.7 to 220.8) | 2.1 (1 to 3.8) | 9.1 (4.5 to 14.6) | 3.3 (1.7 to 5.3) | 191.6 (98.6 to 309.1) | 188.8 (96.9 to 305.6) | 2.9 (1.2 to 5.2) |
| Fiji | 104.6 (68.5 to 144.3) | 68.6 (44.5 to 95.2) | 1406.5 (925.5 to 1979.4) | 1351 (882.3 to 1902.1) | 55.5 (32 to 90.5) | 20.9 (13.1 to 31.4) | 9.5 (6.2 to 14.1) | 257.2 (163.5 to 379.1) | 246.4 (156.7 to 364.5) | 10.8 (6.4 to 17.1) | 3.2 (2.1 to 4.8) | 1.7 (1.1 to 2.4) | 52.2 (33.8 to 75.8) | 50.9 (33 to 73.7) | 1.3 (0.7 to 2.1) | 8.6 (4.4 to 13.6) | 3.5 (1.9 to 5.2) | 136.1 (73.6 to 205.1) | 133.4 (72.7 to 201.2) | 2.6 (1.2 to 4.7) |
| Finland | 6147.2 (5233.3 to 7059.2) | 1342.8 (1152 to 1498.1) | 23546.6 (20243.1 to 26673.8) | 19291 (16763.2 to 21441.2) | 4255.6 (3083.6 to 5667.8) | 670.9 (550.5 to 793.6) | 282.9 (245.6 to 321.9) | 4738.4 (4075 to 5382.5) | 4407.3 (3807.8 to 4981.9) | 331.1 (225.9 to 468) | 649.7 (522.2 to 771.6) | 280.7 (238.2 to 326.4) | 5626.2 (4819.4 to 6445.5) | 5370.3 (4589.5 to 6202.1) | 255.9 (170.5 to 350.6) | 114.6 (80.6 to 156.9) | 5.6 (4.6 to 6.8) | 259.9 (209.9 to 331.7) | 199.1 (163.8 to 241.9) | 60.8 (35.2 to 100.2) |
| France | 62664.9 (47426 to 77649.9) | 13215.6 (11398.8 to 15027.5) | 228048.7 (193006.2 to 260535.4) | 186298.7 (159885.3 to 210882.7) | 41750 (27938.3 to 56458.8) | 15998 (13556 to 19138) | 6285.3 (5609 to 7204.4) | 109879.3 (97299.4 to 126078.9) | 101881.7 (90971.1 to 116502.2) | 7997.6 (5700.4 to 10984.7) | 12775.6 (10590 to 15500.4) | 3720.2 (3198.8 to 4212.5) | 77353.3 (67735.4 to 87824.3) | 71822.9 (63131.3 to 81570.4) | 5530.4 (3823.1 to 7631.7) | 3761.9 (2704.1 to 4807.6) | 125 (109.6 to 146.7) | 6556.6 (5304.4 to 8066.3) | 4576.5 (3962.4 to 5371.3) | 1980.1 (1119.5 to 3237.8) |
| Gabon | 317.3 (214.3 to 446.6) | 209.9 (141.5 to 293.9) | 4465 (3024.4 to 6232.9) | 4294.4 (2904.6 to 6018.8) | 170.5 (98.8 to 258.2) | 37.8 (22 to 58.8) | 22.5 (12.6 to 34.5) | 521.7 (299.1 to 802.8) | 505.1 (284.8 to 774.8) | 16.6 (8.8 to 27.6) | 13.4 (8.2 to 19.8) | 8.1 (5 to 12) | 271.1 (165.2 to 403) | 266.5 (162.7 to 396.6) | 4.6 (2.6 to 7.4) | 6.2 (3.1 to 10.4) | 2 (1 to 3.2) | 97.4 (49 to 155.7) | 95.2 (48.3 to 151.8) | 2.2 (1 to 4.2) |
| Georgia | 1138 (922.4 to 1407.8) | 582.4 (492.7 to 675.7) | 11728.6 (9868.8 to 13539.1) | 11060.3 (9307.1 to 12839.6) | 668.3 (445.1 to 934.2) | 447.3 (364.6 to 545.7) | 246.1 (205 to 293.6) | 5370.2 (4463.1 to 6313) | 5170.1 (4292 to 6110.1) | 200.1 (135.9 to 269.3) | 222.9 (181.7 to 262.8) | 130.6 (109.2 to 152.4) | 3420.2 (2869.2 to 3978.1) | 3341.7 (2786.1 to 3891) | 78.5 (50.7 to 111.8) | 100.5 (70.6 to 136.7) | 25.3 (20.7 to 30.1) | 858.2 (696.3 to 1044.5) | 815.6 (665.6 to 991.3) | 42.6 (23.6 to 67) |
| Germany | 80207.9 (71101.3 to 88735.8) | 20148.6 (17856 to 21926.1) | 351521 (312366.7 to 384927.4) | 295431.5 (265219.9 to 321067.1) | 56089.5 (42058.1 to 74276) | 16276.3 (14331.6 to 18495.9) | 6332.5 (5683.1 to 7130.4) | 107947.5 (97247.8 to 121090.5) | 99733.4 (90247.1 to 111589.9) | 8214.1 (5959.3 to 11131.3) | 11794.3 (10048.5 to 13835) | 5119.9 (4386 to 5881.4) | 99907.6 (87272.7 to 114928.9) | 95328.4 (83193.1 to 109662.2) | 4579.2 (3048.4 to 6625.1) | 5771.2 (4524.4 to 7096.4) | 256.6 (225.6 to 292) | 11515.2 (9890.1 to 13818.3) | 8869.9 (7785.7 to 10148.6) | 2645.3 (1647.9 to 4311.4) |
| Ghana | 3195.6 (2075.4 to 4582.8) | 2233.8 (1452.9 to 3120.6) | 47373.7 (30975.9 to 65031.3) | 45735.5 (29770.5 to 62724.2) | 1638.3 (940.7 to 2474) | 706.6 (425.7 to 1122.7) | 343.4 (205.3 to 539.5) | 10178.1 (6063.1 to 15793.4) | 9840.9 (5861.1 to 15321.6) | 337.2 (190.8 to 537.1) | 195.7 (109 to 315) | 103.4 (55.7 to 166.9) | 4221.5 (2332.3 to 6657.1) | 4151.9 (2294 to 6569.2) | 69.6 (32.6 to 116.1) | 87.7 (39.8 to 145) | 28 (14.5 to 45.1) | 1507.7 (770.7 to 2416.1) | 1481.1 (761.5 to 2362.7) | 26.6 (11.8 to 49.7) |
| Greece | 6144.3 (4935.4 to 7612) | 2454.3 (2125.8 to 2777.3) | 36918.7 (32107.3 to 41657.2) | 32827.3 (28508.1 to 36844.7) | 4091.4 (2727.6 to 5751.4) | 3162 (2735.6 to 3716.5) | 1388.8 (1246.4 to 1550.6) | 23888.7 (21399.7 to 26871.7) | 22320.8 (20170.7 to 25056.9) | 1568 (1110.8 to 2134) | 1053.5 (888.2 to 1234) | 529 (472.9 to 586.3) | 10731.4 (9532.4 to 11972.5) | 10328.3 (9169.6 to 11488) | 403.1 (280.6 to 560.1) | 445.9 (300.4 to 593.8) | 25.4 (21.3 to 30.1) | 1074 (836.7 to 1334.8) | 837 (692.9 to 1007.2) | 236.9 (128.6 to 403.3) |
| Greenland | 9.2 (5.4 to 13.8) | 4.3 (2.6 to 6.3) | 82.8 (49.5 to 120.4) | 77.1 (45.6 to 111.6) | 5.7 (3.2 to 9.1) | 4.4 (3.1 to 6.4) | 2.3 (1.6 to 3.2) | 47 (32.4 to 65.6) | 45 (30.8 to 62.8) | 2 (1.2 to 3) | 5.3 (3.3 to 7.6) | 2.7 (1.6 to 3.8) | 65.4 (40.6 to 91) | 63.4 (39.2 to 87.6) | 2 (1.1 to 3.1) | 0.8 (0.4 to 1.3) | 0.1 (0.1 to 0.1) | 4.6 (2.8 to 6.8) | 4.3 (2.6 to 6.2) | 0.4 (0.2 to 0.7) |
| Grenada | 88.3 (69.1 to 114.8) | 41.3 (34.6 to 49.7) | 802.2 (667.6 to 978.1) | 753.8 (629.8 to 917.3) | 48.4 (32.4 to 68) | 3.6 (2.8 to 4.4) | 1.8 (1.4 to 2.2) | 40.2 (31.6 to 48.9) | 38.5 (30.3 to 46.8) | 1.7 (1.1 to 2.4) | 2.8 (2.3 to 3.6) | 1.6 (1.3 to 2) | 43.6 (35.2 to 53.3) | 42.6 (34.4 to 51.9) | 1 (0.7 to 1.4) | 0.9 (0.6 to 1.3) | 0.2 (0.2 to 0.2) | 8.8 (6.9 to 10.8) | 8.4 (6.6 to 10.3) | 0.4 (0.2 to 0.7) |
| Guam | 16.3 (12.5 to 21.7) | 7.1 (6 to 8.4) | 139.1 (116.6 to 164.9) | 128.2 (108 to 152.5) | 10.9 (7.2 to 15.6) | 8.3 (6.4 to 10.4) | 2.9 (2.4 to 3.5) | 75.8 (61.4 to 93.1) | 70.7 (57.8 to 86) | 5.1 (3.3 to 7.3) | 7.3 (5.9 to 8.9) | 2.8 (2.3 to 3.3) | 83.8 (68.7 to 98.1) | 80.6 (66.2 to 94.2) | 3.2 (2.1 to 4.6) | 0.4 (0.2 to 0.5) | 0.1 (0.1 to 0.1) | 2.5 (2 to 3.1) | 2.4 (1.9 to 2.9) | 0.2 (0.1 to 0.3) |
| Guatemala | 2180.9 (1702.4 to 2721.2) | 1077 (909.6 to 1265.7) | 18751.4 (15971.7 to 21744) | 17596.9 (14925.6 to 20452.7) | 1154.4 (730.6 to 1620.5) | 82.2 (65.9 to 104.3) | 44.5 (36.7 to 54.5) | 946.3 (778.7 to 1157.8) | 905.8 (749 to 1110.8) | 40.5 (26.6 to 57) | 200.5 (162.8 to 240.6) | 106.8 (86.4 to 128.7) | 3255.6 (2617 to 3918.5) | 3178 (2552.8 to 3837.8) | 77.6 (52.1 to 112.2) | 302.1 (214.1 to 411) | 55.3 (44 to 68) | 3156.9 (2461.6 to 3892.5) | 3015.7 (2353.9 to 3761.6) | 141.2 (71.2 to 233.9) |
| Guinea | 910.3 (590 to 1326.8) | 787.8 (507 to 1154.6) | 14816.2 (9840.2 to 21595.4) | 14403.9 (9540.2 to 21070.1) | 412.3 (248.3 to 653.1) | 125.2 (78.3 to 187.2) | 84.4 (52.8 to 125.7) | 1908.8 (1190.3 to 2801.4) | 1855.3 (1162.4 to 2722.4) | 53.5 (30.3 to 86) | 56.8 (34.9 to 83) | 29.7 (18.2 to 43.5) | 1392.1 (857.4 to 2005.3) | 1369.6 (843.5 to 1978.2) | 22.6 (11.1 to 38.6) | 26.4 (14 to 42) | 10.8 (6.1 to 16.5) | 597.5 (340.6 to 932.6) | 590.2 (336.2 to 919) | 7.3 (3.7 to 13.1) |
| Guyana | 288 (221.5 to 379.5) | 160.9 (133.5 to 198.9) | 3309.4 (2736 to 4051.6) | 3151.7 (2610.8 to 3919.8) | 157.8 (104.6 to 224.3) | 14.9 (11.9 to 19.4) | 7.9 (6.4 to 10) | 194.2 (156.6 to 246) | 187.1 (149.6 to 237.9) | 7 (4.6 to 10.3) | 12.7 (9.9 to 15.8) | 6.9 (5.3 to 8.4) | 239.4 (188.6 to 292.5) | 234.6 (183.9 to 286.6) | 4.8 (3.1 to 6.8) | 5.5 (3.7 to 7.5) | 1.6 (1.2 to 1.9) | 77.7 (61.5 to 96) | 75.7 (59.5 to 94.4) | 2 (1.1 to 3.4) |
| Haiti | 1836.5 (1148 to 2735.4) | 1315.6 (838.8 to 1991.5) | 27054.7 (17280.3 to 39509.3) | 26166.1 (16660.4 to 38325) | 888.6 (519.9 to 1401) | 157.1 (89.8 to 234.7) | 95.1 (55.5 to 148.2) | 2336.9 (1354.4 to 3585.7) | 2270.5 (1322 to 3464.5) | 66.4 (36.9 to 118.4) | 130.2 (83.7 to 206.4) | 73 (45.2 to 108.8) | 3041.2 (1982 to 4590.8) | 2994.6 (1940.1 to 4514.9) | 46.6 (23.9 to 79.2) | 38.5 (19.2 to 66.8) | 15.3 (8 to 26) | 841.9 (433.6 to 1472.5) | 832.5 (426.7 to 1447.4) | 9.3 (4.2 to 18.1) |
| Honduras | 633.2 (423 to 886.5) | 426 (290.3 to 604.8) | 6511.8 (4477.6 to 9152.8) | 6216.9 (4242.6 to 8811.3) | 294.9 (177.4 to 455.9) | 31.6 (19 to 50.2) | 20.6 (12.1 to 32.1) | 380.2 (226 to 597.1) | 366.9 (218.6 to 574.7) | 13.4 (7.4 to 22.2) | 43 (26.3 to 67.7) | 26.4 (16.2 to 40.8) | 741.3 (461.5 to 1140.6) | 725.8 (452.1 to 1113.1) | 15.5 (8.2 to 25.6) | 42.9 (20.7 to 75) | 10.5 (5.6 to 17.2) | 571.9 (297.7 to 948.8) | 557 (289.6 to 922.6) | 14.9 (5.7 to 31.9) |
| Hungary | 3705.8 (3049.5 to 4558.2) | 1534.2 (1381.3 to 1670.3) | 28504.4 (25526.7 to 31364.2) | 26264.7 (23590.8 to 28687.1) | 2239.7 (1538.6 to 3021.3) | 1544.1 (1371.3 to 1775.2) | 751.1 (703.7 to 817.4) | 15465.8 (14450 to 16755.1) | 14733.5 (13819.9 to 15947.2) | 732.3 (518.7 to 990.6) | 959.4 (833.1 to 1101.8) | 495.2 (441.3 to 550.1) | 11840.1 (10501.6 to 13305.3) | 11487.6 (10198.7 to 12879.1) | 352.5 (242.1 to 492.1) | 407.7 (285.7 to 536.5) | 40.7 (35.6 to 46.9) | 1752.7 (1510.2 to 2057.5) | 1541.1 (1336.5 to 1785.8) | 211.6 (115.8 to 349.3) |
| Iceland | 296.5 (229.4 to 385.6) | 84 (69.7 to 99) | 1376.8 (1165.6 to 1619.4) | 1179.6 (987.6 to 1386.8) | 197.2 (133.2 to 277.6) | 44.4 (34.9 to 55.9) | 17.2 (14.1 to 20.8) | 296.2 (242.8 to 357.7) | 272.9 (224.2 to 330.4) | 23.3 (16.2 to 32.6) | 48 (37 to 60.2) | 19.3 (15.7 to 23.6) | 413 (335.2 to 503.5) | 393.4 (320.1 to 478.8) | 19.7 (13.1 to 26.7) | 10.8 (8 to 13.9) | 0.3 (0.2 to 0.4) | 17.4 (13.9 to 21.9) | 11.6 (9.6 to 14.6) | 5.8 (3.4 to 8.6) |
| India | 45449.5 (30368.8 to 64376.7) | 28334 (19484.4 to 40090.6) | 561499.5 (389781.9 to 783992.6) | 536733 (369412.5 to 746749.7) | 24766.5 (14806.3 to 38692.1) | 19889.6 (13017.4 to 28171.5) | 10996.1 (7293.3 to 15415.5) | 254155.9 (168646.2 to 356355.3) | 244760 (162371.8 to 343215.5) | 9395.9 (5670.9 to 14201) | 9077.1 (5814.4 to 13386.6) | 5833.9 (3633.4 to 8461.7) | 163897.3 (104257 to 237421) | 160717.6 (101823.6 to 232439.1) | 3179.8 (1850.8 to 5093.4) | 7335 (3960.1 to 12138.7) | 1736.8 (1038.4 to 2613.8) | 89661.8 (52360.1 to 135606.2) | 86801.8 (51317.5 to 131393.4) | 2860 (1371.9 to 5622.9) |
| Indonesia | 15093 (9783.7 to 22114.5) | 8670.9 (5717.7 to 12600.8) | 197604.5 (129558.3 to 286679.6) | 188865.6 (123601.4 to 276629.6) | 8738.9 (5017.2 to 14167.9) | 4331 (2412.5 to 7391.8) | 1830.5 (990.5 to 3210.3) | 51955.9 (28586.4 to 88119) | 49550.3 (27057.1 to 85112.7) | 2405.6 (1329.1 to 4036) | 3604.5 (2100.5 to 5523.1) | 1775.6 (1105 to 2628.6) | 58122.5 (36224.6 to 87108.2) | 56709.3 (35374.8 to 85003.6) | 1413.2 (776.9 to 2366.3) | 1411.7 (684 to 2261.1) | 449.2 (235.9 to 677.8) | 21084.7 (10717.9 to 32009.3) | 20609.3 (10498.6 to 31448.8) | 475.4 (218.9 to 877.7) |
| Iran (Islamic Republic of) | 11703.3 (8205 to 16059.7) | 3935.3 (2887.4 to 5242.1) | 75522.6 (55383.5 to 98653.5) | 68344.1 (50097.9 to 89405.1) | 7178.6 (4584.5 to 11131.7) | 3291.1 (2194.8 to 4878.4) | 1239.4 (832.8 to 1823.3) | 29003.9 (19494.2 to 42216) | 27160.6 (18266.7 to 39726.4) | 1843.3 (1093.6 to 2855.2) | 1577.1 (1043.6 to 2389.3) | 653.8 (431.1 to 948.2) | 18919.7 (12606.2 to 27192.8) | 18236.6 (12129.6 to 26305.4) | 683.1 (386.7 to 1179.8) | 1487.5 (719.1 to 2381.7) | 105.5 (59.6 to 156.4) | 5180.2 (2864.4 to 7942.1) | 4394.3 (2416.9 to 6712.1) | 785.9 (369 to 1431.1) |
| Iraq | 2687.1 (1791.8 to 3735.1) | 1009 (676.9 to 1390.9) | 21725.3 (14509.1 to 29773.9) | 20083.5 (13359.8 to 27373.8) | 1641.8 (1010.8 to 2510.4) | 3618.8 (2598.7 to 4842.7) | 1403.8 (1003.5 to 1831.2) | 36301.2 (26273.8 to 47081.5) | 34461.7 (24888.6 to 45080.5) | 1839.5 (1189.5 to 2900.6) | 904.1 (622 to 1321.2) | 379.2 (263.5 to 553.8) | 13023.1 (8773.1 to 18253.2) | 12647 (8562.1 to 17715.8) | 376.1 (221.5 to 649.4) | 620 (296 to 1034.5) | 58.2 (32.1 to 89.3) | 3352.9 (1832 to 5154) | 3054.2 (1649.6 to 4732.9) | 298.7 (129.4 to 580) |
| Ireland | 3322.8 (2565.1 to 4248.5) | 859.7 (762 to 947) | 14574.2 (12540.6 to 16219.2) | 12332.6 (10842.7 to 13699) | 2241.7 (1455.4 to 3135.7) | 590.8 (490.2 to 703.8) | 223 (193.7 to 257.1) | 3925.9 (3397.2 to 4505.7) | 3621.6 (3141.6 to 4185.5) | 304.4 (213.8 to 415.8) | 442.1 (349.9 to 546.3) | 179.3 (149.7 to 213.3) | 3857.1 (3243.2 to 4593.1) | 3676.4 (3090.6 to 4392.2) | 180.7 (119.3 to 245.7) | 222.9 (156.3 to 284.4) | 7.6 (6.3 to 9) | 388.6 (299.2 to 498.6) | 268.7 (222.7 to 321.5) | 119.9 (66.8 to 196.3) |
| Israel | 2322.3 (1802.4 to 2908.1) | 745.4 (655.2 to 824) | 12293.6 (10726.7 to 13703.2) | 10753.5 (9532.3 to 11941.4) | 1540.1 (1011.6 to 2122.8) | 997.9 (852.5 to 1158.8) | 430.6 (385.4 to 475.9) | 7396.2 (6667.8 to 8162.2) | 6896.4 (6259.9 to 7651.5) | 499.8 (357.5 to 694.8) | 524.9 (420.2 to 638.2) | 240.1 (200.9 to 283) | 5190.6 (4374.4 to 6150.8) | 4980 (4216.9 to 5868.9) | 210.6 (142.9 to 295.3) | 200.2 (131.3 to 268.9) | 7.7 (6.4 to 9.1) | 415.9 (330.7 to 527.1) | 308.1 (255.1 to 371.4) | 107.7 (62.6 to 187.3) |
| Italy | 36327.1 (29362.8 to 43588.3) | 10538.3 (8916.9 to 11816.5) | 168148.2 (143193 to 190526.1) | 143684.7 (122944.1 to 160630.6) | 24463.5 (16890.6 to 33184.7) | 20837 (17716.1 to 24286.8) | 6591.4 (5858 to 7271.8) | 109105 (96867.2 to 121062.3) | 97669.2 (86763.4 to 108410.7) | 11435.8 (7939.6 to 15414.1) | 8252.2 (6639.6 to 10019.5) | 3208.1 (2743 to 3703.2) | 63670.1 (54581.9 to 73941.8) | 60268.6 (51833.7 to 69858.2) | 3401.5 (2281.1 to 4570) | 2687 (1853.5 to 3597.1) | 139.4 (116.1 to 168.7) | 5900.7 (4652.6 to 7366.6) | 4478.4 (3688 to 5476.6) | 1422.3 (814.1 to 2249) |
| Jamaica | 1954.5 (1510 to 2495.4) | 875.9 (748.6 to 1038.4) | 16001.3 (13451.4 to 19200.3) | 14917.6 (12485.7 to 18082.3) | 1083.7 (749.3 to 1535) | 107 (85.3 to 132.3) | 52.4 (42.9 to 64.1) | 1122.3 (926.7 to 1363.2) | 1071.8 (877.9 to 1298.8) | 50.5 (33.8 to 72) | 57.2 (44.5 to 72.8) | 27.9 (22.6 to 35.2) | 823.4 (672 to 1032.9) | 801.9 (654.8 to 1000.6) | 21.5 (14.6 to 30.4) | 29.4 (20 to 40.8) | 4.4 (3.5 to 5.5) | 221 (170.4 to 278.4) | 210.7 (164 to 267.1) | 10.3 (5.4 to 17.6) |
| Japan | 60978.6 (52825.3 to 68782.1) | 18762.7 (16340.3 to 20575.1) | 283150.9 (247012.4 to 307514.8) | 241874 (214960.2 to 263284.9) | 41277 (30951.9 to 54348.2) | 24194.1 (21573.6 to 26420.2) | 8763.5 (7549 to 9578.9) | 131484.2 (116329.3 to 142322.1) | 119073.3 (105223.8 to 129724.8) | 12410.9 (9154.4 to 16261) | 13012.1 (10982.8 to 14950) | 6526.5 (5621.1 to 7294.7) | 110792.9 (97988.4 to 123813.5) | 105918.5 (93566.1 to 118112.4) | 4874.4 (3375.1 to 6547.8) | 2455.6 (1730.9 to 3263.4) | 109 (90.9 to 133.1) | 4841 (3870.8 to 6148.4) | 3567.7 (2942.1 to 4378.8) | 1273.3 (740.5 to 2040.5) |
| Jordan | 1423.5 (966.8 to 1974.4) | 471.4 (364.3 to 600.2) | 9546.9 (7431.1 to 12188.6) | 8739.5 (6764.4 to 11151.2) | 807.4 (488 to 1208.1) | 669.2 (460.1 to 958.1) | 248.5 (172.9 to 347.1) | 6148.2 (4296.4 to 8564.2) | 5801.9 (4024.8 to 8112.3) | 346.3 (201.3 to 529.1) | 182.4 (117.8 to 275.4) | 76.4 (50.1 to 109.5) | 2295.2 (1492.4 to 3301.2) | 2220.8 (1446.5 to 3202) | 74.4 (41.7 to 123.6) | 194.4 (93.7 to 337.2) | 15.5 (8.7 to 23.4) | 805.2 (443.9 to 1225.9) | 711.3 (394.1 to 1082.5) | 93.9 (41.8 to 179.3) |
| Kazakhstan | 1467.1 (1205.2 to 1822.1) | 636.7 (568.1 to 704.5) | 14170.1 (12548.4 to 15661.9) | 13249.1 (11665.7 to 14739.8) | 921.1 (619.3 to 1288.2) | 607.1 (489.8 to 737.9) | 293.6 (248.3 to 344.7) | 7235 (6111.8 to 8512.3) | 6925.7 (5817.4 to 8127.2) | 309.3 (204.5 to 434.7) | 507.9 (425.4 to 600.8) | 268.7 (228.6 to 305.4) | 7762 (6634.8 to 8805.5) | 7567.9 (6421 to 8582.5) | 194.1 (130.8 to 275.8) | 197.8 (132.2 to 268.8) | 36.5 (29.5 to 43.4) | 1501.3 (1195.1 to 1807.3) | 1411 (1137.6 to 1680.6) | 90.3 (49.6 to 157.9) |
| Kenya | 2363 (1472.6 to 3441.9) | 1648.1 (1009 to 2447) | 33970.9 (21312.7 to 49253.1) | 32827.5 (20525 to 47589.9) | 1143.4 (687.7 to 1724.8) | 260.6 (168.8 to 396.7) | 147.1 (92.4 to 216) | 3672.5 (2357.9 to 5476.6) | 3554.9 (2254.2 to 5300.8) | 117.6 (67.2 to 185.4) | 182.5 (118.6 to 273.5) | 112.7 (68.4 to 167.5) | 3712.1 (2408.1 to 5494.2) | 3650.2 (2368.8 to 5388.1) | 61.9 (35.1 to 97.1) | 65.4 (34.6 to 108.7) | 17.8 (10.5 to 26.8) | 982 (573.1 to 1484.4) | 958.6 (560.1 to 1450.5) | 23.4 (10.5 to 43.3) |
| Kiribati | 2 (1.3 to 3) | 1.5 (1 to 2.2) | 32.7 (20.7 to 49.6) | 31.6 (19.7 to 48) | 1.1 (0.6 to 1.9) | 0.3 (0.1 to 0.4) | 0.1 (0.1 to 0.2) | 3.7 (2.2 to 5.7) | 3.5 (2.1 to 5.5) | 0.2 (0.1 to 0.3) | 0.8 (0.5 to 1.3) | 0.4 (0.2 to 0.7) | 16.3 (9.8 to 24.8) | 15.9 (9.6 to 24.3) | 0.3 (0.2 to 0.6) | 0.1 (0.1 to 0.1) | 0 (0 to 0.1) | 2 (1.1 to 3) | 2 (1.1 to 3) | 0 (0 to 0) |
| Kuwait | 351.7 (252.2 to 460.1) | 60.4 (50.1 to 72.7) | 1454.9 (1194.9 to 1754.6) | 1234.3 (1030.8 to 1481) | 220.6 (139.1 to 333.7) | 164.5 (129 to 204) | 41.2 (33.2 to 51) | 1220.5 (988.4 to 1508.7) | 1114.7 (905.5 to 1367.4) | 105.8 (70.6 to 155.5) | 85.7 (63.7 to 106.4) | 23.9 (18.6 to 28.7) | 834.9 (646.8 to 1005.7) | 794.4 (615.9 to 958.6) | 40.6 (26.9 to 58.1) | 49.3 (33 to 71.7) | 1.7 (1.4 to 2) | 109.4 (85.7 to 137.8) | 82.4 (67.3 to 99) | 27 (13.3 to 45.9) |
| Kyrgyzstan | 231.4 (184.3 to 297.9) | 108.1 (93.7 to 124.7) | 2486.8 (2166.5 to 2838.5) | 2342.5 (2051.9 to 2686.7) | 144.2 (96.8 to 205.8) | 92 (74.1 to 115.2) | 44.4 (36.5 to 54.4) | 1164.2 (954.9 to 1435.4) | 1115.2 (912.5 to 1381.7) | 48.9 (31.7 to 68.4) | 95.9 (77.1 to 118.6) | 49.9 (40.5 to 59.6) | 1583.2 (1289 to 1903.2) | 1545.8 (1260.5 to 1857.3) | 37.4 (24.6 to 51.5) | 43.5 (31 to 56.8) | 8.8 (7.3 to 10.7) | 387.3 (311.5 to 477.1) | 368.7 (300 to 452.5) | 18.6 (9.2 to 30.3) |
| Lao People's Democratic Republic | 290.5 (180.9 to 444.9) | 206.9 (131.4 to 322) | 4208.1 (2681.6 to 6372.8) | 4054.6 (2586.7 to 6163.1) | 153.5 (90.4 to 244) | 91.4 (54.3 to 148.7) | 44.1 (25.6 to 70.4) | 1156.3 (670.1 to 1889.3) | 1110.4 (647.5 to 1801.8) | 45.9 (25.4 to 79.7) | 49.7 (29.3 to 74.7) | 25.6 (14.5 to 38.3) | 878.7 (497.2 to 1335.7) | 859.4 (482.6 to 1310.2) | 19.3 (10.5 to 31.8) | 23.4 (12.6 to 37.1) | 8.8 (5.1 to 13.3) | 422.1 (237 to 644) | 415.5 (234 to 634.6) | 6.6 (3 to 12.2) |
| Latvia | 1541.2 (1210.5 to 1938.9) | 477.9 (428.9 to 530.9) | 9368 (8382.9 to 10390.7) | 8358.9 (7505 to 9226.2) | 1009.1 (660.1 to 1399.4) | 313.9 (266.7 to 368.3) | 162.6 (142.4 to 188.3) | 3294.6 (2833.5 to 3804.8) | 3148.6 (2731 to 3624.9) | 145.9 (99.7 to 202.1) | 263.3 (224.1 to 308.2) | 126.5 (110.1 to 143.2) | 3056.3 (2639.2 to 3493.8) | 2956 (2552.6 to 3381.7) | 100.3 (67.6 to 141.8) | 49.8 (32.6 to 67.2) | 7.7 (6.3 to 9.3) | 284.9 (228.5 to 342.4) | 261 (212.7 to 313.7) | 23.9 (12.8 to 37.2) |
| Lebanon | 824.7 (572.4 to 1160.3) | 348.3 (248.2 to 473.5) | 5834.8 (4215.8 to 7700) | 5323.1 (3787.9 to 7039.8) | 511.7 (312.2 to 784) | 590.5 (402.6 to 804) | 241 (163.8 to 331.5) | 5239.3 (3539.2 to 7220.8) | 4914 (3310 to 6744.8) | 325.2 (197.8 to 495.9) | 156 (102.9 to 227.5) | 68.2 (44.9 to 98.7) | 1859 (1214.8 to 2628.6) | 1790.3 (1165.8 to 2538.3) | 68.7 (39.2 to 114.9) | 137.4 (71 to 229.5) | 10 (6 to 14.9) | 510.4 (302 to 764.1) | 438 (263.2 to 654.9) | 72.4 (30.6 to 135) |
| Lesotho | 141 (85.6 to 219.2) | 118.3 (72.4 to 187.5) | 2394.5 (1464.1 to 3861.5) | 2327.6 (1424.4 to 3759.1) | 66.9 (37.1 to 103.8) | 16.2 (9.9 to 25.2) | 10.6 (6.5 to 16.1) | 256.7 (160.4 to 399.9) | 250.3 (156.3 to 391.5) | 6.4 (3.6 to 10.2) | 10 (5.4 to 15.3) | 7.1 (3.7 to 11.2) | 230.6 (124 to 362.8) | 227.5 (122.3 to 358.4) | 3.1 (1.6 to 5.3) | 3.4 (1.8 to 5.2) | 1.8 (1.1 to 2.8) | 77 (43.7 to 120.3) | 76.2 (43.2 to 119) | 0.8 (0.4 to 1.5) |
| Libya | 469.3 (269.8 to 727.5) | 233.1 (131.4 to 358) | 4333.5 (2463.4 to 6716.3) | 4079.9 (2322.8 to 6369.8) | 253.6 (133.4 to 428.1) | 503.2 (299.5 to 793.1) | 222 (131 to 345.3) | 5386.4 (3158.4 to 8312.7) | 5137.9 (3001.3 to 7928.2) | 248.5 (139.7 to 402.8) | 147.5 (88.6 to 220.5) | 67.1 (38 to 104.5) | 2222.3 (1324.5 to 3464.5) | 2163.3 (1282.5 to 3370.4) | 58.9 (30.7 to 98.5) | 47.4 (22.9 to 78.8) | 5.3 (3 to 8.2) | 302.3 (166.4 to 467.4) | 278.4 (154.9 to 435.6) | 23.9 (10.4 to 46.5) |
| Lithuania | 2585.4 (2108.1 to 3158) | 602.4 (547.1 to 655.1) | 12192.7 (11043 to 13372.9) | 10483.6 (9557.7 to 11398.6) | 1709.1 (1187.1 to 2364.7) | 371.1 (327.4 to 425.3) | 201.3 (188 to 217.1) | 3947.9 (3700.1 to 4227.9) | 3781.8 (3556.6 to 4026.5) | 166 (117.9 to 220.7) | 421.3 (360.1 to 482.5) | 195.2 (176.5 to 213) | 4721.1 (4171.6 to 5198.8) | 4562 (4029 to 5015.3) | 159.1 (108.7 to 223.7) | 44.6 (32.1 to 60.7) | 7.2 (6 to 8.9) | 262.4 (218 to 316.3) | 241.2 (200.7 to 294.8) | 21.2 (11.7 to 34.1) |
| Luxembourg | 279.7 (212.9 to 359.5) | 75.4 (65.3 to 88.4) | 1288.5 (1110.8 to 1502) | 1097.5 (950.1 to 1281.5) | 191 (125.2 to 273.1) | 75.5 (60.5 to 94.9) | 28.6 (24.1 to 34.7) | 526.3 (441.6 to 640.3) | 486.3 (409.5 to 589.9) | 40 (27.3 to 54.2) | 29 (22 to 36) | 12.3 (9.9 to 15.1) | 269.3 (214.8 to 331.5) | 257.2 (206 to 317.1) | 12.1 (8 to 16.9) | 22.1 (15.2 to 29.7) | 0.8 (0.7 to 1) | 41.3 (32.4 to 52.9) | 29.7 (24.8 to 37) | 11.6 (6.6 to 18.9) |
| Malaysia | 2674.2 (1896.6 to 3629.3) | 1384.9 (1058.3 to 1737.1) | 25910.6 (19504.7 to 32612.9) | 24347.1 (18429.7 to 30556.9) | 1563.5 (978.5 to 2261.3) | 819.6 (589.3 to 1170.9) | 360.5 (252 to 503.6) | 8037.6 (5523 to 11124.5) | 7605.2 (5223.9 to 10548.5) | 432.4 (273 to 643) | 558.6 (359.2 to 803.9) | 241.3 (151.1 to 337.8) | 6973.9 (4348.1 to 9737.7) | 6744.3 (4238.1 to 9453.5) | 229.7 (123.7 to 366.9) | 300.4 (171.9 to 487.8) | 59.6 (37 to 87.4) | 2599.7 (1648.1 to 3793.3) | 2470.5 (1560.9 to 3620.7) | 129.2 (55.5 to 243.1) |
| Maldives | 35.4 (23.3 to 49.5) | 15.5 (9.7 to 21.8) | 279.1 (184.3 to 383.6) | 258.9 (169.3 to 356.5) | 20.3 (12 to 32.4) | 9.9 (6.6 to 14.2) | 4.2 (2.8 to 5.8) | 85.6 (56.8 to 119) | 80.2 (53.5 to 112.2) | 5.4 (3.2 to 8.1) | 4.4 (3 to 6.5) | 1.5 (1 to 2.1) | 47 (32.5 to 68) | 45 (31 to 65.2) | 2 (1.1 to 3.7) | 8 (4.7 to 12.9) | 1.1 (0.7 to 1.5) | 47.8 (30.7 to 71.2) | 43.8 (28 to 64) | 4 (1.9 to 7.7) |
| Malta | 210.9 (156 to 272.7) | 51.5 (43.1 to 60.7) | 900.8 (757 to 1061.5) | 752.8 (631.2 to 883.5) | 148.1 (92 to 213.5) | 79.3 (64.2 to 97.2) | 32.9 (27.6 to 39.5) | 582.2 (493.1 to 691.1) | 541.7 (454.9 to 646.1) | 40.6 (28 to 56.6) | 47.3 (38.3 to 59.7) | 21.1 (17.3 to 26.1) | 456.4 (382.4 to 565.9) | 437.3 (363.1 to 541.1) | 19 (12.6 to 27) | 24.3 (16 to 32.8) | 1 (0.8 to 1.2) | 50.1 (39.4 to 63.8) | 36.9 (29.7 to 44.1) | 13.2 (7.1 to 21.7) |
| Marshall Islands | 4.2 (2.8 to 5.7) | 2.7 (1.9 to 3.8) | 61.4 (41.5 to 83.7) | 59.2 (40.2 to 80.9) | 2.2 (1.2 to 3.4) | 0.8 (0.5 to 1.3) | 0.4 (0.2 to 0.6) | 11.2 (6.6 to 17.6) | 10.7 (6.3 to 17) | 0.4 (0.2 to 0.7) | 0.3 (0.2 to 0.4) | 0.1 (0.1 to 0.2) | 4.9 (2.7 to 8) | 4.8 (2.6 to 7.7) | 0.1 (0 to 0.2) | 0.1 (0 to 0.1) | 0 (0 to 0) | 1 (0.6 to 1.5) | 1 (0.6 to 1.5) | 0 (0 to 0) |
| Mauritania | 502.8 (297.2 to 761.8) | 342.3 (199.2 to 530) | 6784.4 (4005.2 to 10277) | 6555.4 (3847.1 to 9959.5) | 229 (121.7 to 383.3) | 70.9 (39.7 to 112.3) | 40.9 (23.4 to 63.9) | 944.6 (546.3 to 1437) | 916.6 (529.9 to 1395.2) | 28 (14.8 to 44.8) | 30.7 (17.1 to 47.1) | 16.7 (9 to 25.9) | 600.2 (339 to 911.8) | 589.4 (331.8 to 896.9) | 10.8 (5.2 to 18.7) | 10.2 (4.6 to 16.9) | 2.7 (1.4 to 4.2) | 148.6 (74.5 to 232) | 145.2 (72.9 to 228.4) | 3.4 (1.5 to 6.4) |
| Mauritius | 297.6 (231.4 to 370.8) | 134.6 (116.7 to 156) | 2679 (2293.5 to 3109.1) | 2499.8 (2150.1 to 2904.2) | 179.2 (116.6 to 247.3) | 65 (51.9 to 81.4) | 27.1 (22.7 to 32.8) | 620.7 (514.8 to 748.6) | 585.7 (486.2 to 709.6) | 34.9 (23.6 to 50.8) | 45.4 (35 to 56.7) | 19.5 (15.6 to 24) | 541.5 (433.4 to 669.3) | 522.5 (418.2 to 643.8) | 19 (12.4 to 26.9) | 21.4 (15.5 to 29.2) | 4.2 (3.5 to 5.1) | 170.3 (139.4 to 208.4) | 160.6 (132.4 to 196.6) | 9.7 (5.5 to 15.3) |
| Mexico | 23085.3 (18663.5 to 28199.1) | 8903.6 (8220.2 to 9502.9) | 162325.5 (151647.2 to 173561.1) | 149285 (139789.9 to 159964.1) | 13040.5 (8727.8 to 17955.8) | 2102.4 (1730.3 to 2600.1) | 1046.1 (881.8 to 1237.5) | 22147.8 (18694.8 to 26106.3) | 21103.5 (17765.7 to 24970) | 1044.4 (730.6 to 1471.2) | 5093.8 (4245.8 to 5961.7) | 2587.9 (2230.9 to 2972.5) | 73774.5 (63435.5 to 84723.1) | 71841.1 (61810.1 to 82630.9) | 1933.4 (1307.8 to 2565.7) | 6320.7 (4694 to 8235) | 866 (748.3 to 1021.7) | 49461.5 (42819.3 to 57902.8) | 46395.1 (40373.2 to 54726.8) | 3066.4 (1765.3 to 4828.8) |
| Micronesia (Federated States of) | 6.5 (4.3 to 9.1) | 4.4 (3 to 5.9) | 94.1 (64.5 to 128.9) | 90.5 (62.2 to 123.8) | 3.6 (2.2 to 5.3) | 1.7 (1.1 to 2.5) | 0.8 (0.5 to 1.1) | 21.8 (13.7 to 32) | 20.9 (13.3 to 30.6) | 0.9 (0.6 to 1.4) | 0.2 (0.1 to 0.3) | 0.1 (0.1 to 0.2) | 3.5 (2.1 to 5.2) | 3.4 (2 to 5.1) | 0.1 (0 to 0.1) | 0.1 (0.1 to 0.2) | 0.1 (0 to 0.1) | 2.4 (1.5 to 3.5) | 2.4 (1.4 to 3.4) | 0 (0 to 0.1) |
| Monaco | 53.8 (33.1 to 86) | 17 (10.5 to 25.5) | 272.5 (172 to 389.1) | 240.1 (150.9 to 343.8) | 32.4 (18 to 51.5) | 12.1 (6.9 to 19.7) | 5.3 (3 to 8.6) | 86.2 (50.3 to 137.5) | 81 (46.6 to 130.1) | 5.3 (3 to 9.2) | 7.4 (4.6 to 11.7) | 3.5 (2.1 to 5.2) | 69.9 (42.1 to 103.9) | 67.3 (40.7 to 99.8) | 2.6 (1.4 to 4.4) | 5.8 (2.9 to 9.8) | 0.3 (0.1 to 0.4) | 11.9 (6.4 to 17.6) | 9.1 (4.8 to 13.1) | 2.9 (1.4 to 5.3) |
| Mongolia | 69.5 (47.3 to 95.9) | 36 (25.4 to 51.2) | 867.9 (616.5 to 1224.5) | 826.4 (588 to 1164.5) | 41.5 (25.4 to 61) | 37 (23.9 to 53.2) | 18.4 (12 to 25.6) | 513.4 (332.3 to 714.1) | 495.5 (321.6 to 690.9) | 17.9 (10.6 to 28) | 58.5 (39.2 to 84.3) | 32.1 (20.9 to 45.4) | 1083.2 (711.2 to 1510) | 1063.5 (695 to 1481.1) | 19.8 (11.1 to 31.6) | 26.5 (15.8 to 45) | 7 (4.5 to 10.8) | 307.3 (197.3 to 471.6) | 298.2 (190.8 to 455.6) | 9.1 (3.8 to 16.4) |
| Montenegro | 296.1 (208.4 to 404.8) | 114.5 (79.9 to 145.5) | 2111.5 (1512.2 to 2709.6) | 1929.6 (1366.2 to 2478.1) | 181.9 (112.8 to 277.4) | 82.6 (57.1 to 114.8) | 38.8 (26.8 to 53.2) | 791.5 (550.1 to 1085.8) | 752 (522.2 to 1028.9) | 39.5 (24.7 to 60) | 43 (29 to 59.5) | 21 (14.1 to 28.3) | 501.9 (343.6 to 678.8) | 485.6 (331.6 to 653.8) | 16.3 (9.9 to 26.5) | 35.2 (19.4 to 55) | 2.7 (1.7 to 3.7) | 126.9 (79.4 to 177.3) | 108.3 (68.8 to 154.4) | 18.5 (9.2 to 34.2) |
| Morocco | 1695.3 (737.3 to 2902) | 981.5 (426.3 to 1639.3) | 17850.4 (7638.9 to 29660.3) | 17046.4 (7215.9 to 28536) | 804 (344.3 to 1485.3) | 945.7 (546.6 to 1556.7) | 505.6 (290 to 834.7) | 10761.5 (6256.3 to 17532.1) | 10374.6 (5994.7 to 16958.1) | 386.9 (214.3 to 638.9) | 66.6 (38.3 to 103.4) | 40.6 (22.7 to 63.9) | 1052 (607.7 to 1631.3) | 1030.5 (594.5 to 1597.3) | 21.5 (11.4 to 35.8) | 94.6 (47.8 to 154.6) | 15.9 (9.3 to 24.7) | 805.3 (462.7 to 1267.6) | 765.2 (439.5 to 1216.7) | 40 (17 to 78.3) |
| Myanmar | 3687.2 (2294 to 5458.8) | 2502.4 (1562.8 to 3779.2) | 51751.9 (31917.9 to 77607.4) | 49776.7 (30604.8 to 74771) | 1975.2 (1209.5 to 3196.3) | 860.1 (500.2 to 1341.3) | 422.2 (241.9 to 679.9) | 10707.5 (6083.6 to 16785.2) | 10276.2 (5811 to 16122.9) | 431.3 (240 to 717.7) | 418.1 (258.7 to 640.3) | 207.2 (123.5 to 305.6) | 7249.1 (4260.3 to 10898.8) | 7083 (4145.2 to 10696.5) | 166.1 (87.2 to 271.7) | 215.3 (116.1 to 354.1) | 80.8 (47.5 to 131.7) | 3682.5 (2102.8 to 5882.3) | 3619 (2071.9 to 5784.4) | 63.5 (29.1 to 113.7) |
| Namibia | 186.7 (121.3 to 246.2) | 131.1 (85.3 to 169.6) | 2767.6 (1772.3 to 3621.3) | 2663.9 (1710.5 to 3484.6) | 103.7 (63.1 to 147.1) | 17.6 (11.2 to 27) | 9.3 (6 to 14.1) | 257.9 (165.4 to 387.6) | 249 (159.2 to 374.6) | 8.8 (5.3 to 13.8) | 20.2 (12.4 to 31.2) | 11.4 (6.9 to 17.2) | 439.9 (269.8 to 667.1) | 433.1 (266.3 to 657) | 6.8 (3.4 to 10.7) | 20.8 (10.7 to 34.5) | 6.2 (3.2 to 9.7) | 345 (176.9 to 544.5) | 338 (174 to 534.9) | 7 (2.8 to 13) |
| Nauru | 0.7 (0.4 to 1) | 0.4 (0.3 to 0.6) | 10.6 (7.1 to 14.9) | 10.2 (6.8 to 14.2) | 0.4 (0.2 to 0.6) | 0.3 (0.2 to 0.4) | 0.1 (0.1 to 0.1) | 3.4 (2 to 5.4) | 3.2 (1.9 to 5.2) | 0.1 (0.1 to 0.2) | 0.1 (0 to 0.1) | 0 (0 to 0.1) | 1.6 (0.8 to 2.5) | 1.6 (0.8 to 2.5) | 0 (0 to 0.1) | 0 (0 to 0) | 0 (0 to 0) | 0.5 (0.3 to 0.8) | 0.5 (0.3 to 0.8) | 0 (0 to 0) |
| Nepal | 692.4 (409.6 to 1115.6) | 511 (310.7 to 810.6) | 9723.7 (5934.3 to 15602.1) | 9371.1 (5726.9 to 15000.1) | 352.7 (175.4 to 619.1) | 274.7 (154.5 to 476.9) | 165.7 (92.4 to 277.7) | 3762.7 (2129.3 to 6436.3) | 3639.4 (2050.9 to 6233.3) | 123.2 (67.3 to 212.1) | 135.8 (73.3 to 244.8) | 93.7 (52.7 to 169.6) | 2595 (1436.5 to 4654.3) | 2548.8 (1410.2 to 4550.3) | 46.2 (20.3 to 99) | 111.3 (57.2 to 186.8) | 31.2 (17 to 50.9) | 1721.5 (925.1 to 2833.1) | 1685.5 (909.3 to 2758.7) | 36.1 (14.7 to 76.5) |
| Netherlands | 13145.2 (10738.9 to 15789.6) | 4263.5 (3716.3 to 4730.7) | 70447 (62080.6 to 78580.9) | 61866.8 (54598.5 to 68469.6) | 8580.2 (5848.2 to 12225.8) | 2832.6 (2453.1 to 3292.2) | 1413.2 (1268.2 to 1582.1) | 23049.4 (20866.8 to 25776.6) | 21716.5 (19754.2 to 24332.8) | 1333 (922.9 to 1809.1) | 2038.4 (1715.1 to 2349.9) | 989.9 (865.8 to 1097.2) | 19901.2 (17579.4 to 22158.5) | 19115.3 (16930.8 to 21217.1) | 786 (562 to 1070.4) | 743 (524.5 to 995.6) | 33 (27.7 to 40.6) | 1513.3 (1217 to 1895.5) | 1121 (935.7 to 1381.7) | 392.2 (214.9 to 634.1) |
| New Zealand | 4616.3 (3472.1 to 5679.3) | 984.6 (850.2 to 1099) | 17332.9 (14793.8 to 19522.4) | 14209.8 (12408.9 to 15838) | 3123.2 (2112.9 to 4275.9) | 568.8 (476.3 to 666.6) | 226.2 (198.6 to 258.4) | 3926.1 (3464.2 to 4464) | 3644.2 (3222.7 to 4146.8) | 281.9 (202.1 to 384) | 729.7 (562 to 894.9) | 169.1 (141.5 to 198.9) | 3978.8 (3342.4 to 4714.4) | 3647.7 (3069.3 to 4342) | 331.2 (224.2 to 469.4) | 239.3 (164.6 to 307.4) | 7 (5.8 to 8.4) | 434.2 (345.7 to 535.8) | 310.1 (260.6 to 375) | 124.2 (73.7 to 193.1) |
| Nicaragua | 998.4 (735.4 to 1351.2) | 306.5 (230.5 to 399) | 6122.8 (4772.2 to 7767.3) | 5564.4 (4224.3 to 7218.4) | 558.4 (348.2 to 809.5) | 45.1 (29.5 to 65.8) | 19.9 (12.8 to 29.1) | 459.3 (296.6 to 653.3) | 435.5 (281.4 to 624.8) | 23.9 (14.6 to 35.5) | 106.2 (65.9 to 160.2) | 47.9 (28.4 to 70.7) | 1471.8 (898.8 to 2097.3) | 1428.9 (875.1 to 2033.7) | 42.9 (23.9 to 66.9) | 135.3 (70.1 to 222.2) | 16 (9.6 to 24.9) | 903.9 (531.8 to 1425.9) | 837.7 (494.8 to 1312.6) | 66.2 (31.2 to 135.1) |
| Nigeria | 18864.5 (11591.3 to 27679.7) | 13458.5 (8481 to 19924.1) | 272133.8 (170259.1 to 398770.8) | 263457.7 (164838 to 390332.1) | 8676.1 (5243.5 to 13061.9) | 1152.7 (662 to 1849.8) | 690.5 (392.3 to 1064.6) | 16182.1 (9062.1 to 24882.1) | 15685.4 (8794.4 to 24061.1) | 496.7 (275.1 to 806.7) | 1192 (689.3 to 1799.3) | 566 (314.4 to 882.7) | 25890.6 (15121.8 to 38276.5) | 25456.7 (14784.5 to 37545.5) | 433.9 (228.2 to 758.2) | 105.8 (55.3 to 177.5) | 29.9 (17.1 to 45.7) | 1662 (941.7 to 2540.3) | 1625.5 (920.2 to 2492.5) | 36.5 (15.7 to 73.6) |
| North Macedonia | 382.3 (247.1 to 493.7) | 246.8 (158.3 to 319.9) | 4453.4 (2855 to 5739.3) | 4246.5 (2695.1 to 5513) | 206.9 (124.2 to 300.4) | 249.5 (189.1 to 326.6) | 130.4 (101.4 to 162.7) | 2642.2 (2057.4 to 3324.8) | 2527.8 (1959.8 to 3181.8) | 114.4 (76.5 to 168.5) | 73.7 (47.1 to 103.7) | 40.5 (25 to 54.6) | 975.9 (601.9 to 1298.5) | 948 (584.3 to 1265) | 27.9 (16.6 to 44.4) | 86.2 (49.4 to 137) | 10.6 (7 to 14.8) | 446.4 (289.1 to 646) | 403.5 (262.9 to 575) | 42.9 (21.1 to 77.4) |
| Northern Mariana Islands | 7.7 (4.3 to 12.7) | 3.2 (1.9 to 5.2) | 70.3 (41.5 to 115.2) | 65.3 (38.8 to 106) | 5 (2.5 to 8.4) | 2.1 (1.3 to 3) | 0.7 (0.5 to 1) | 20.4 (13.4 to 29.3) | 19.1 (12.5 to 27.4) | 1.3 (0.7 to 2) | 1.4 (0.9 to 2) | 0.6 (0.4 to 0.8) | 17 (11.4 to 24.4) | 16.4 (11 to 23.7) | 0.6 (0.3 to 1) | 0.2 (0.1 to 0.3) | 0 (0 to 0) | 1.4 (0.8 to 2) | 1.3 (0.8 to 1.9) | 0.1 (0 to 0.1) |
| Norway | 4322 (3562.7 to 5097.3) | 1266.1 (1093.9 to 1440.8) | 20465.3 (17699.4 to 22958) | 17610.9 (15266.8 to 20041.1) | 2854.4 (2047.6 to 3855.8) | 697.9 (560.9 to 887.6) | 281.7 (234.8 to 334.7) | 4666.6 (3939.4 to 5475.2) | 4310.8 (3628.6 to 5091.1) | 355.9 (253.2 to 491.8) | 466.1 (354.7 to 572.7) | 193.5 (156.6 to 235.2) | 3924.2 (3197 to 4768.4) | 3735 (3048.2 to 4530) | 189.2 (125.8 to 256.8) | 178.9 (141 to 227) | 5.7 (4.7 to 7) | 293.4 (235.3 to 367.2) | 200.7 (166.1 to 249.9) | 92.7 (60 to 140.8) |
| Oman | 188.1 (117.4 to 279.3) | 62.7 (41.6 to 88.6) | 1365 (901.8 to 1942.8) | 1249 (835.4 to 1773.9) | 116 (62.1 to 190.1) | 108.6 (68.4 to 172.2) | 36.6 (22.9 to 55.6) | 1030.8 (647.9 to 1579.6) | 967.2 (613.8 to 1494.9) | 63.5 (36.6 to 102) | 27.6 (16.3 to 43.5) | 10.6 (6 to 16.4) | 364.1 (204.3 to 568.5) | 351.8 (198 to 548.5) | 12.3 (6.4 to 22) | 3.7 (1.7 to 6.7) | 0.3 (0.1 to 0.4) | 16.5 (8.1 to 26.5) | 14.5 (7.2 to 22.9) | 2 (0.9 to 3.9) |
| Pakistan | 5620.9 (3708.1 to 8208.3) | 4152.5 (2794 to 5912.4) | 81779.5 (54513.8 to 117588.8) | 78821.6 (52526.2 to 113660.2) | 2957.9 (1748.5 to 4390.7) | 3394.7 (2025.4 to 5214.5) | 2057.5 (1203.1 to 3173.3) | 48277.5 (28948.3 to 74605.3) | 46713 (27784.8 to 71952.1) | 1564.5 (888.8 to 2653.2) | 1818.4 (1146.1 to 2805.1) | 1097.7 (684.9 to 1710) | 37933.5 (23886.7 to 57624.9) | 37262.2 (23474.2 to 56475.9) | 671.3 (360.8 to 1176.8) | 1613.5 (823.5 to 2748.5) | 492.7 (275.1 to 754.7) | 26817.7 (14950.2 to 41611.5) | 26300.8 (14645.2 to 40422.6) | 516.9 (215.4 to 975.2) |
| Palau | 3.9 (2.5 to 5.7) | 2.4 (1.6 to 3.4) | 47.7 (31.9 to 67.1) | 45.7 (30.8 to 64) | 2 (1.2 to 3.1) | 0.2 (0.2 to 0.4) | 0.1 (0.1 to 0.2) | 2.9 (1.8 to 4.3) | 2.8 (1.7 to 4.1) | 0.1 (0.1 to 0.2) | 0.1 (0.1 to 0.2) | 0.1 (0 to 0.1) | 1.7 (1.1 to 2.5) | 1.7 (1.1 to 2.5) | 0 (0 to 0.1) | 0 (0 to 0.1) | 0 (0 to 0) | 0.5 (0.3 to 0.8) | 0.5 (0.3 to 0.8) | 0 (0 to 0) |
| Palestine | 356.3 (251.8 to 500.2) | 138.5 (103.6 to 177.8) | 2828 (2103.9 to 3712.3) | 2605.9 (1935.4 to 3415) | 222.1 (134.3 to 333.8) | 161.3 (112.5 to 235.7) | 66.3 (46.2 to 94.8) | 1621.4 (1131.5 to 2308.4) | 1533.9 (1077 to 2178.3) | 87.5 (53 to 136.7) | 50.3 (33.3 to 75.1) | 22 (14.4 to 31.6) | 701.2 (470.5 to 1007.5) | 679.3 (455.9 to 977.6) | 22 (12.4 to 36.6) | 18.8 (9.9 to 31.7) | 2 (1.2 to 3) | 102.2 (59.9 to 154.2) | 92.1 (54.2 to 139.9) | 10.1 (4.3 to 19.1) |
| Panama | 1612.4 (1325.2 to 1944.3) | 476.4 (415.2 to 534.6) | 8499.8 (7551.6 to 9518.5) | 7566.7 (6779.5 to 8452.2) | 933.1 (656.1 to 1294.5) | 60.2 (47.4 to 73.7) | 28.1 (22.6 to 34.2) | 552.3 (445.9 to 661.6) | 520.4 (423.8 to 628.5) | 32 (20.7 to 44.5) | 145.8 (114.2 to 176.7) | 64.3 (51.7 to 77) | 1745.7 (1402.5 to 2074) | 1685.8 (1359 to 2004.2) | 59.8 (37.2 to 88.2) | 103.3 (71.5 to 142.1) | 9.4 (7.7 to 11.3) | 504.6 (412.6 to 614) | 451.5 (360.9 to 553.5) | 53.1 (31.1 to 85.8) |
| Papua New Guinea | 599.2 (386 to 877.3) | 395 (258.2 to 570.6) | 8304.5 (5419 to 12167.2) | 7974 (5190.6 to 11664) | 330.5 (188.8 to 518.3) | 98.6 (50.5 to 167.2) | 41.1 (19.4 to 69.2) | 1208 (588.2 to 2032.5) | 1152.1 (553 to 1952.9) | 55.9 (27.9 to 101.5) | 42.4 (18.4 to 76.5) | 20.4 (8.9 to 36.3) | 708.3 (311.1 to 1271.2) | 690.7 (303.6 to 1232.1) | 17.6 (7.4 to 33) | 10.8 (4.7 to 21.1) | 3.6 (1.6 to 6.9) | 178.2 (79.7 to 340.5) | 174.6 (78.4 to 332.5) | 3.6 (1.3 to 7.9) |
| Paraguay | 1194.9 (717 to 1592.9) | 584.6 (348.9 to 720) | 10864.3 (6631.8 to 13368.5) | 10187.4 (6194.6 to 12559.7) | 676.9 (387 to 1026.1) | 124.8 (83.8 to 162.2) | 68.9 (45.9 to 89.5) | 1408.8 (968.2 to 1808.6) | 1350.6 (926.4 to 1739) | 58.2 (32.7 to 81.7) | 143.6 (80.4 to 201) | 78.3 (42.9 to 109.5) | 2274 (1294.8 to 3114.3) | 2216.3 (1260.6 to 3046.1) | 57.7 (31.5 to 89.6) | 123.9 (78 to 187.3) | 24.6 (16.6 to 35.2) | 1245.3 (828 to 1777.7) | 1184.6 (793 to 1689.2) | 60.7 (30.9 to 101.7) |
| Peru | 12884.2 (8999.1 to 18169.8) | 3744.4 (2781.4 to 4912.5) | 62938 (46885 to 81826.6) | 57582.3 (42887.8 to 75533.7) | 5355.7 (3249.3 to 8057.1) | 973.9 (780.5 to 1207) | 343.2 (283.4 to 395) | 6661.7 (5432.6 to 8161.5) | 6280.8 (5153.2 to 7647.2) | 380.8 (255.2 to 546.1) | 1880.9 (1321.5 to 2468.2) | 600.6 (378.5 to 774.1) | 17221.1 (11286.8 to 22202.7) | 16664 (10882.6 to 21592.8) | 557 (331.2 to 884) | 1169.7 (731.7 to 1676.4) | 139.4 (103.5 to 174.9) | 6745.5 (4973 to 8613.3) | 6375.9 (4716.7 to 8107.2) | 369.6 (195.2 to 587) |
| Philippines | 6758.7 (4822.9 to 8647.5) | 4149.7 (2864.9 to 5381) | 85351.8 (60635.8 to 111660.6) | 81567 (57723.8 to 106457.9) | 3784.8 (2367.5 to 5592.9) | 1042.2 (726 to 1420.2) | 441.6 (305.8 to 610.9) | 11944.8 (8208.9 to 16602.1) | 11358.8 (7843.9 to 15724.4) | 586 (373.1 to 850.1) | 1437.9 (984.9 to 2015.5) | 664.6 (446.3 to 932.2) | 22602.2 (14963.6 to 30992.8) | 22000.8 (14549.5 to 30136.6) | 601.4 (364.6 to 943.9) | 496.9 (300 to 735.9) | 146 (95.3 to 203.5) | 6976.4 (4508.7 to 9816.9) | 6797.1 (4421.5 to 9508.4) | 179.2 (87.5 to 306.8) |
| Poland | 13525.9 (12114.7 to 15190.2) | 7084.2 (6581.6 to 7543.3) | 126931.4 (117786.1 to 135289.7) | 119255.3 (110991.8 to 126754.8) | 7676.1 (5658.4 to 10296.4) | 6675.9 (6091.7 to 7317) | 3742.9 (3492.7 to 4042.8) | 72905.2 (68008.6 to 78688.2) | 69946 (65081.8 to 75890) | 2959.2 (2132.6 to 3848.8) | 3058.3 (2668.4 to 3461.2) | 1976.1 (1785.7 to 2184.5) | 44412.1 (40124.5 to 49184) | 43393.9 (39199.4 to 48050.6) | 1018.2 (732.8 to 1360.3) | 1314.8 (962.2 to 1733) | 162.4 (144.5 to 184) | 7171.7 (6396.7 to 8140.7) | 6499.4 (5713.2 to 7377.5) | 672.4 (406.3 to 1063.5) |
| Portugal | 9968.9 (7583.7 to 12811.5) | 2460.2 (2135.1 to 2743.2) | 41585 (35221.7 to 46763.4) | 34743.9 (30382.1 to 38701.8) | 6841.1 (4534.5 to 9678.8) | 3183 (2698.8 to 3763.6) | 831.3 (739.6 to 927.4) | 15211.1 (13485.1 to 17084.3) | 13394.2 (11912.7 to 15005.2) | 1816.9 (1315.6 to 2462.9) | 1129.5 (924.1 to 1358.3) | 371.6 (322.1 to 421.1) | 7979.6 (6927.1 to 9006.6) | 7490.2 (6507.6 to 8483.5) | 489.4 (326.1 to 678.2) | 281.7 (188.2 to 383.5) | 16.4 (13.9 to 19.9) | 711.8 (573.4 to 892.1) | 562.6 (477.1 to 689.9) | 149.1 (83 to 241.7) |
| Puerto Rico | 2210.6 (1733.1 to 2781.2) | 668.6 (583.4 to 746.6) | 11511.1 (10094.4 to 12861.8) | 10104.8 (8904 to 11233.9) | 1406.2 (920.2 to 1980.2) | 281.1 (236 to 340.2) | 125.3 (108 to 144.9) | 2308 (1993.2 to 2635.7) | 2156.4 (1871.3 to 2468.7) | 151.6 (103.5 to 217.9) | 189.8 (148.4 to 234.9) | 81.7 (66 to 98.2) | 1932.9 (1561.5 to 2329.5) | 1850.6 (1497.1 to 2223.2) | 82.3 (53.3 to 113.6) | 90.9 (63.3 to 123.8) | 8 (6.6 to 9.8) | 334.9 (273.4 to 416.2) | 285.8 (233.8 to 358.1) | 49.1 (27.5 to 81.3) |
| Qatar | 116 (63 to 205.8) | 21.5 (12.8 to 33.6) | 543.8 (309.5 to 887.1) | 467.6 (269.8 to 757.2) | 76.2 (35.3 to 134.7) | 33.8 (21.1 to 51.4) | 8.1 (5 to 11.8) | 260.4 (159.4 to 373.9) | 237 (145.4 to 344.9) | 23.4 (13.4 to 36.4) | 21.3 (13.7 to 33.7) | 5.6 (3.8 to 8.3) | 217.7 (143.7 to 335.2) | 207.4 (137.7 to 316.4) | 10.3 (5.4 to 18.9) | 9.1 (4.8 to 15.7) | 0.3 (0.2 to 0.5) | 22.7 (12.7 to 35.2) | 17.8 (10.2 to 27.2) | 4.9 (2.2 to 9.9) |
| Republic of Korea | 11459.4 (7956.4 to 15609.4) | 3057.9 (2139.3 to 3804.5) | 53569 (40112.4 to 66659.8) | 45863.2 (33622.6 to 57059) | 7705.8 (4806.1 to 11318.1) | 5661.6 (4689.8 to 7059) | 1788 (1503.1 to 2122.1) | 31558.2 (27239.9 to 37868.5) | 28538.7 (24541 to 33990.3) | 3019.5 (2118.2 to 4296.3) | 2706.3 (1808.6 to 3693.6) | 1094.4 (698.1 to 1377.1) | 24889.6 (16163.6 to 32443.1) | 23755.2 (15534 to 30912.5) | 1134.4 (665.7 to 1814.7) | 368.8 (220.2 to 552.1) | 13.2 (9.4 to 16.9) | 728.7 (500.1 to 985.6) | 540.7 (383 to 690.4) | 188 (89.5 to 341.1) |
| Republic of Moldova | 1134.6 (889.7 to 1442.4) | 340.2 (295.3 to 393.8) | 7816.8 (6709.6 to 9036.8) | 7072.2 (6079.4 to 8288.1) | 744.6 (511.1 to 1079.8) | 307.4 (250 to 384.4) | 153.9 (129.2 to 185.5) | 3581.1 (3020.9 to 4288.6) | 3434.6 (2882.1 to 4082.8) | 146.4 (96.9 to 202) | 205.4 (165 to 246.2) | 100.5 (82.2 to 120.3) | 2743.8 (2237.3 to 3266.4) | 2665.9 (2175.8 to 3178.6) | 77.9 (49.2 to 114.1) | 47.7 (32 to 64.6) | 9.5 (7.7 to 11.3) | 357.1 (285.2 to 432.5) | 335.3 (272.9 to 402.8) | 21.8 (12.3 to 36.1) |
| Romania | 5635.4 (4554.3 to 6864.7) | 2641.4 (2330.6 to 2989.7) | 49224.8 (43430.1 to 55783.4) | 45822.3 (40480.9 to 51908.6) | 3402.5 (2347.2 to 4656.6) | 2762.5 (2389.8 to 3256) | 1419.5 (1264.7 to 1601.7) | 29581.6 (26112 to 33747.8) | 28279.7 (24860.6 to 31920.2) | 1301.9 (903.1 to 1735.8) | 1336.1 (1108.5 to 1604.2) | 707 (608.1 to 836.2) | 18075.7 (15426 to 21756.1) | 17577.4 (15001.4 to 21189.5) | 498.3 (327.2 to 716.5) | 546.3 (385.6 to 749.3) | 68.9 (57.4 to 84.7) | 2931.2 (2419.6 to 3612.8) | 2658 (2176.5 to 3272.9) | 273.2 (156.1 to 440.7) |
| Russian Federation | 62553.5 (52275.7 to 77470.1) | 16357.2 (14580.7 to 18414.6) | 358074 (315930.7 to 405205.5) | 316392.4 (283236 to 357058.7) | 41681.6 (29385.1 to 57008.7) | 13253.7 (11060.8 to 15832.2) | 5435.3 (4654.5 to 6259.2) | 123894.7 (105804.3 to 143029.8) | 116976.1 (100154.4 to 134737.5) | 6918.6 (4844.4 to 9225.8) | 13848.8 (11830.3 to 16516.7) | 5999.9 (5208.5 to 6826.3) | 158732.2 (137871.7 to 180845.7) | 153221.9 (132764.9 to 175236.6) | 5510.4 (3930.1 to 7645.8) | 3865.7 (2555.6 to 5315.8) | 338.1 (275.9 to 404.9) | 15645.7 (12416.2 to 18956.3) | 13575.1 (11088.9 to 16277.7) | 2070.6 (1158.6 to 3297.7) |
| Saint Kitts and Nevis | 33.6 (26.4 to 43.8) | 16.5 (13.7 to 19.4) | 308.1 (256.1 to 366.5) | 287.6 (239.4 to 340.7) | 20.6 (14 to 30.2) | 1.5 (1.2 to 1.8) | 0.8 (0.7 to 0.9) | 17.1 (14.3 to 20.6) | 16.3 (13.7 to 19.7) | 0.8 (0.5 to 1.1) | 1.1 (0.9 to 1.3) | 0.6 (0.5 to 0.7) | 16.6 (13.8 to 19.9) | 16.1 (13.3 to 19.3) | 0.5 (0.3 to 0.7) | 0.6 (0.4 to 0.7) | 0.1 (0.1 to 0.1) | 5.3 (4.3 to 6.5) | 5 (4.1 to 6.1) | 0.3 (0.1 to 0.5) |
| Saint Lucia | 139.6 (109.2 to 178.7) | 62.6 (50.8 to 75.5) | 1163.4 (962.6 to 1403.5) | 1083.9 (882.1 to 1315.3) | 79.6 (54.4 to 111.5) | 7.1 (5.6 to 9.2) | 3.5 (2.9 to 4.4) | 76.2 (62.2 to 94.3) | 72.7 (58.7 to 90.5) | 3.5 (2.3 to 5) | 3.5 (2.9 to 4.4) | 1.7 (1.4 to 2.2) | 51.9 (42.3 to 65.2) | 50.4 (40.8 to 63.4) | 1.4 (1 to 2.1) | 2.4 (1.7 to 3.2) | 0.4 (0.3 to 0.5) | 19.1 (15.7 to 23.7) | 17.9 (14.7 to 22.2) | 1.2 (0.7 to 2) |
| Saint Vincent and the Grenadines | 97.6 (76.4 to 123.4) | 52.3 (43.4 to 61.6) | 936.3 (783 to 1117.7) | 883.4 (737.3 to 1048.3) | 52.9 (34.9 to 75.6) | 4 (3.2 to 5) | 2.2 (1.7 to 2.7) | 46.3 (37 to 56.9) | 44.3 (35.2 to 54) | 1.9 (1.2 to 2.7) | 1.8 (1.4 to 2.2) | 1 (0.8 to 1.2) | 28.6 (22.8 to 35.8) | 28 (22.3 to 35) | 0.7 (0.5 to 1) | 0.8 (0.6 to 1.2) | 0.2 (0.2 to 0.2) | 8.2 (6.6 to 10.3) | 7.8 (6.3 to 10) | 0.4 (0.2 to 0.6) |
| Samoa | 9.1 (4 to 15) | 5.6 (2.5 to 9.4) | 110.7 (47 to 185.1) | 105.6 (44.9 to 176.6) | 5.2 (2.1 to 9.4) | 2.7 (1.6 to 4.4) | 1.3 (0.8 to 2.1) | 31.7 (19 to 49.6) | 30.2 (18 to 47.5) | 1.5 (0.9 to 2.3) | 0.6 (0.3 to 0.9) | 0.2 (0.1 to 0.4) | 8.2 (5.1 to 13.2) | 8 (4.9 to 12.8) | 0.2 (0.1 to 0.4) | 1.6 (0.8 to 2.5) | 0.4 (0.3 to 0.6) | 20.8 (12.2 to 30) | 20.1 (11.8 to 29.1) | 0.6 (0.3 to 1.2) |
| San Marino | 24.1 (14.9 to 34.8) | 6.7 (4.4 to 9.5) | 107 (69.1 to 147.8) | 90.6 (59.4 to 127.2) | 16.4 (9.1 to 25.3) | 9 (5.6 to 13.8) | 3.6 (2.2 to 5.5) | 59.4 (37.8 to 88.7) | 54.9 (34.6 to 80.6) | 4.6 (2.7 to 7.2) | 2.5 (1.5 to 3.9) | 1 (0.7 to 1.6) | 21 (13.3 to 32.3) | 19.9 (12.7 to 30.6) | 1 (0.5 to 1.9) | 1.9 (1.1 to 2.9) | 0.1 (0 to 0.1) | 2.9 (1.8 to 4.6) | 2 (1.2 to 3.1) | 0.9 (0.4 to 1.6) |
| Sao Tome and Principe | 14.3 (9.2 to 19.8) | 9.6 (6.1 to 13.7) | 191.5 (129.1 to 263.3) | 184.3 (124.1 to 255.5) | 7.1 (4.2 to 10.7) | 9.3 (5.6 to 14.9) | 4.7 (2.7 to 7.1) | 124.5 (71.4 to 188.3) | 120.3 (68.6 to 182.1) | 4.2 (2.4 to 6.7) | 1.3 (0.8 to 2) | 0.6 (0.3 to 0.9) | 26.5 (15.8 to 39.2) | 26 (15.5 to 38.6) | 0.5 (0.2 to 0.8) | 0.6 (0.3 to 1) | 0.2 (0.1 to 0.2) | 8.2 (4.4 to 13.1) | 8 (4.3 to 12.9) | 0.2 (0.1 to 0.4) |
| Saudi Arabia | 1528.6 (971.1 to 2634.6) | 520.4 (354.7 to 803.4) | 11090.6 (7707.1 to 17561) | 10231.6 (6996.4 to 16185.2) | 859 (484.7 to 1504.1) | 836.2 (585.8 to 1281.3) | 285.6 (201.8 to 427.7) | 7776.1 (5556.3 to 11463.5) | 7319.4 (5169.2 to 10893.9) | 456.6 (288.1 to 714.8) | 373.1 (241.3 to 568.6) | 140.8 (93.7 to 214) | 4923.2 (3261 to 7366.8) | 4769.4 (3158.2 to 7094.3) | 153.8 (84.6 to 275.3) | 252.2 (123.9 to 427.6) | 18.7 (10.5 to 29) | 1126.1 (630.7 to 1718.4) | 993.7 (550.1 to 1538.7) | 132.4 (53.3 to 259.4) |
| Senegal | 1200.6 (700.6 to 1835.6) | 940.1 (548.4 to 1416.4) | 18119.3 (10753.3 to 27918.1) | 17538.9 (10379.5 to 26982.1) | 580.5 (323.4 to 987.1) | 177.4 (105.5 to 268.8) | 109.1 (65 to 161.2) | 2563.8 (1531.3 to 3771) | 2484.2 (1481.5 to 3637.8) | 79.6 (44.6 to 128.2) | 97.4 (58.7 to 146.2) | 50.4 (29 to 76.9) | 2168.6 (1278.6 to 3237) | 2130.4 (1256.2 to 3182.8) | 38.2 (19.6 to 67.7) | 29.2 (15.3 to 49.9) | 9.8 (5.5 to 15.7) | 545.5 (302.9 to 880.3) | 536.5 (297.3 to 866.6) | 9 (4.1 to 17.1) |
| Serbia | 3180.4 (2275.8 to 4103.3) | 1413.7 (1050.4 to 1750.1) | 26048.9 (19328.3 to 32065.5) | 24159.3 (17997.7 to 29876.6) | 1889.6 (1180.7 to 2747.2) | 1660.7 (1312.2 to 2097.3) | 733.4 (603.1 to 902) | 15132.6 (12363.9 to 18760) | 14325.2 (11573.8 to 17775.6) | 807.4 (532 to 1140.2) | 678.6 (441.1 to 952) | 358.6 (233.4 to 473.4) | 8641.2 (5575.8 to 11607.8) | 8390.2 (5427.4 to 11232.1) | 251.1 (147.9 to 392.8) | 337.5 (199 to 526.9) | 45 (29.6 to 62.7) | 1857.4 (1230.5 to 2563.6) | 1691.2 (1126.7 to 2313.2) | 166.2 (84.6 to 295.3) |
| Seychelles | 48.8 (33.1 to 67.9) | 23 (15.6 to 31.7) | 476.7 (330.1 to 651.9) | 446.1 (304.1 to 616) | 30.6 (18.2 to 46.5) | 7.6 (5.5 to 11) | 3.1 (2.2 to 4.3) | 75.7 (54.4 to 103.2) | 71.5 (50.3 to 97) | 4.2 (2.6 to 6.7) | 1.4 (0.9 to 2) | 0.6 (0.4 to 0.8) | 18.4 (11.5 to 25.6) | 17.7 (11.1 to 24.6) | 0.6 (0.3 to 1) | 2.2 (1.1 to 3.7) | 0.4 (0.2 to 0.6) | 18.9 (10.9 to 29.1) | 17.9 (10.3 to 27.2) | 1 (0.4 to 1.9) |
| Singapore | 1252.2 (951.3 to 1585) | 318.1 (279.8 to 353.7) | 5836.7 (5135.4 to 6557.3) | 5017.3 (4501.9 to 5600.4) | 819.5 (547.9 to 1161.8) | 344.9 (270.6 to 447.2) | 102.9 (85.7 to 122.6) | 1965.6 (1653.6 to 2360.6) | 1772.5 (1482.2 to 2115.7) | 193.1 (131.9 to 270.5) | 309.4 (236.4 to 378.9) | 125 (100.6 to 149.9) | 2912.2 (2389.1 to 3497.1) | 2783.2 (2296.8 to 3349) | 129.1 (83.8 to 180.6) | 84.3 (57.7 to 118) | 2.9 (2.4 to 3.5) | 173.3 (138.4 to 224.3) | 126.7 (103.7 to 155.6) | 46.6 (25.7 to 78.6) |
| Slovakia | 1978.5 (1431.6 to 2541.6) | 824.3 (609.6 to 946.1) | 15604.7 (11711 to 18169.8) | 14404.7 (10787.9 to 16613.4) | 1200.1 (794.5 to 1759.7) | 681 (581.6 to 798) | 341.6 (285.7 to 389.2) | 6918.4 (5967.3 to 7742.7) | 6599.6 (5675.7 to 7341.6) | 318.8 (216.3 to 437.2) | 549.5 (348.9 to 711.1) | 281.1 (170.9 to 354.6) | 6924 (4272.9 to 8759.8) | 6718.5 (4121.2 to 8527.9) | 205.5 (118.8 to 307.6) | 279.2 (155.9 to 440.4) | 25.8 (16.2 to 37.3) | 1215.2 (761 to 1788.2) | 1072.6 (670.6 to 1554.4) | 142.6 (70 to 251.7) |
| Slovenia | 1592.4 (1333.3 to 1909.7) | 529.9 (481.1 to 576.4) | 8989 (8229.2 to 9746.6) | 8047.9 (7386.3 to 8660.4) | 941.1 (675.5 to 1314.3) | 319.2 (268.6 to 373.5) | 174.2 (150.6 to 195.2) | 3160.1 (2761 to 3564.9) | 3015.1 (2632.8 to 3393) | 145 (102.1 to 200.2) | 267.1 (220.3 to 316.3) | 111.7 (94.6 to 126.6) | 2376.7 (2035.6 to 2720.5) | 2270.6 (1934.7 to 2591.4) | 106.1 (70.9 to 148) | 96.4 (66 to 127.5) | 5.1 (4.1 to 5.9) | 225.3 (175.8 to 284.1) | 174.3 (142.1 to 206.4) | 51 (28.3 to 82.7) |
| Solomon Islands | 85.3 (54.8 to 127.5) | 58.9 (37.9 to 87.4) | 1248.8 (805.5 to 1869.9) | 1204.1 (776 to 1798.7) | 44.7 (26.4 to 72.4) | 15.9 (9.1 to 25.2) | 7.1 (3.9 to 11.3) | 207.5 (114.8 to 332.3) | 199.4 (109.7 to 319.1) | 8.1 (3.9 to 13.9) | 4 (1.8 to 6.8) | 2.1 (0.9 to 3.7) | 69.9 (30.9 to 118.7) | 68.4 (30.1 to 116.3) | 1.5 (0.6 to 2.9) | 0.8 (0.4 to 1.4) | 0.4 (0.2 to 0.6) | 14 (7.1 to 25) | 13.8 (6.9 to 24.5) | 0.2 (0.1 to 0.5) |
| South Africa | 9672.8 (7220.1 to 12217.4) | 6212.7 (4748.5 to 7632.7) | 130486.1 (102104.7 to 159666.9) | 125040.4 (96714.8 to 153143.1) | 5445.7 (3579.6 to 7553.1) | 1356.7 (1006.8 to 1941.8) | 733 (547.2 to 1002) | 18221.3 (13530.5 to 25086.8) | 17534.2 (12952.7 to 24145.7) | 687.1 (458.8 to 1009.7) | 624.2 (418 to 888.7) | 380.7 (247.6 to 525.2) | 12066.1 (8117.2 to 16814.6) | 11829.6 (7959.1 to 16486.7) | 236.5 (141.6 to 359) | 275 (152.7 to 439.6) | 101.2 (59.7 to 147.5) | 3996.6 (2311.2 to 5914.5) | 3895.7 (2249.6 to 5763.7) | 100.8 (49.6 to 179.7) |
| Spain | 26993.5 (20921.8 to 33786.8) | 7369.4 (6493.2 to 8167) | 121185.1 (106374.4 to 133872.8) | 102752.7 (91589.6 to 112900) | 18432.3 (12362.8 to 25539.9) | 12730 (10925.1 to 14918.5) | 4798.1 (4340.2 to 5284) | 81984.7 (74193 to 90726.9) | 75324.6 (68917.1 to 83236.4) | 6660.1 (4754.7 to 8940) | 5123.9 (4371.4 to 5910.6) | 2218 (1977.3 to 2433.4) | 46174.2 (41585 to 50586.8) | 44095.9 (39888.4 to 48613.2) | 2078.3 (1453.6 to 2755.3) | 1454.8 (1024.8 to 1837.3) | 54.3 (46.9 to 64) | 2658.1 (2172.7 to 3250.9) | 1876.9 (1620.8 to 2201.8) | 781.2 (438.1 to 1212.1) |
| Sri Lanka | 3037.2 (1952.2 to 4205.4) | 1187.9 (786.6 to 1679.1) | 23816.9 (16366.6 to 33268.9) | 21742.9 (14818.4 to 30805.4) | 2074 (1229.2 to 3207.9) | 923.8 (662.2 to 1210.8) | 373.5 (261.4 to 478.9) | 8191.6 (5971.9 to 10461.5) | 7642.5 (5505 to 9743.1) | 549.1 (348.4 to 818.9) | 463.6 (331.7 to 704.8) | 189.5 (129.8 to 270) | 4964.9 (3443.4 to 7235.4) | 4742 (3316.8 to 6816.2) | 222.8 (136.8 to 380.8) | 218.9 (114.6 to 365.7) | 36.5 (21.2 to 54.2) | 1402.2 (817.3 to 2116.6) | 1292.6 (735.2 to 1926.8) | 109.6 (49.8 to 216) |
| Suriname | 162.3 (112.9 to 205.9) | 97.3 (68.9 to 117.3) | 1774.9 (1238.1 to 2140.4) | 1682.2 (1175.4 to 2037.4) | 92.7 (58.1 to 134.7) | 13 (8.5 to 18.9) | 7.3 (4.9 to 10.6) | 159.9 (106.7 to 226.8) | 153.4 (103.2 to 218.4) | 6.5 (3.8 to 10.3) | 10.3 (7 to 14.8) | 5.8 (3.9 to 8.2) | 181 (119.4 to 256.8) | 176.8 (116 to 251.4) | 4.2 (2.4 to 6.7) | 3.9 (2.3 to 6.1) | 1 (0.6 to 1.6) | 49.9 (30.4 to 77.1) | 48.3 (29.8 to 74.7) | 1.6 (0.7 to 3.5) |
| Sweden | 8818.1 (7552.7 to 10255.4) | 2870.2 (2509.7 to 3212.9) | 44894.7 (39041.9 to 49786.6) | 39085.9 (34415.2 to 43451.9) | 5808.8 (4148.5 to 7850.9) | 1418.1 (1202.7 to 1647.5) | 590.4 (534.6 to 646.6) | 9451 (8628.1 to 10276.6) | 8735.6 (7939.6 to 9594) | 715.4 (511.4 to 983.5) | 880.5 (752.3 to 1009.8) | 483.5 (427.8 to 538.8) | 9148.4 (8215.9 to 10146.1) | 8827 (7940.6 to 9803.1) | 321.4 (218.3 to 429.9) | 310.9 (220.8 to 406.2) | 11.7 (9.8 to 14.5) | 556.2 (438.5 to 715.7) | 392.7 (321.9 to 492.2) | 163.5 (89.5 to 258) |
| Switzerland | 6019.1 (4705 to 7620.5) | 1714.4 (1493.1 to 1906.1) | 27156 (23551.6 to 30516.5) | 23105.1 (20299.6 to 25679.4) | 4050.9 (2677.1 to 5785.8) | 1242.4 (1028.6 to 1443) | 502.9 (441.7 to 564.4) | 8237.2 (7254.2 to 9325.8) | 7603 (6681.4 to 8583) | 634.2 (454 to 878.8) | 635.9 (510.8 to 783.9) | 280.4 (238.6 to 323.8) | 5420.1 (4566.2 to 6241.6) | 5164.9 (4370.8 to 5946) | 255.1 (158.6 to 346.3) | 286.3 (211.2 to 360.3) | 10.8 (9.1 to 13) | 498.1 (388.1 to 628.6) | 345.1 (285.5 to 417) | 152.9 (90 to 239) |
| Taiwan (Province of China) | 9489.3 (7811.1 to 11259.2) | 2465 (2231.1 to 2686.1) | 44643.9 (39973.5 to 48824.1) | 38729 (35275.1 to 42247.8) | 5914.9 (4063 to 8312.8) | 3501.8 (2903 to 4214.1) | 1158.3 (1021.8 to 1303.8) | 23087.2 (20154.6 to 26546.4) | 21133.1 (18664 to 24115.5) | 1954.1 (1401.8 to 2675.4) | 3313.7 (2639.6 to 4183.1) | 934.5 (779.1 to 1120.8) | 22915.7 (19069.4 to 27471.3) | 21476.3 (17872 to 25745.6) | 1439.3 (950.7 to 2111.3) | 462.3 (324.8 to 616.6) | 32 (26.3 to 39) | 1323.3 (1054.8 to 1665.8) | 1082.3 (880.4 to 1326) | 241 (125.9 to 407.9) |
| Tajikistan | 239.8 (158.3 to 360.7) | 133.8 (92.8 to 193.8) | 3060.2 (2154.6 to 4461.1) | 2921.1 (2063.1 to 4286.4) | 139 (82.2 to 220.4) | 64.8 (40.5 to 97.7) | 36.3 (22.8 to 55.7) | 892 (566.2 to 1367.7) | 859.3 (544.6 to 1315.6) | 32.6 (18.8 to 52.1) | 156.2 (98.6 to 247.6) | 89.9 (56.8 to 141.2) | 2869.1 (1866.2 to 4399.5) | 2810.2 (1824.6 to 4310.1) | 58.9 (30.8 to 106.1) | 1.9 (1 to 3.1) | 0.6 (0.3 to 0.9) | 23.4 (14 to 35.5) | 22.7 (13.5 to 34.4) | 0.7 (0.3 to 1.4) |
| Thailand | 15747.3 (10073.8 to 26390.2) | 6240.7 (4194 to 9896.1) | 124311.3 (84152.5 to 188148.2) | 114515.1 (78027.1 to 172814.2) | 9796.2 (5294.6 to 16029.9) | 4634.6 (3591.8 to 6314.7) | 1783.7 (1412.1 to 2343.4) | 40819.5 (32929.6 to 54226.3) | 38219.4 (30786.3 to 50535) | 2600.1 (1701 to 3722.1) | 2709.6 (1928.3 to 4332.6) | 1048 (754.4 to 1693) | 29549.4 (21342.8 to 45669.9) | 28363.4 (20411.8 to 43959.4) | 1186.1 (719.5 to 2175.3) | 805.9 (502.9 to 1297.9) | 113.5 (79.1 to 163.7) | 5047.4 (3642.7 to 7374.9) | 4651 (3329.5 to 6858.4) | 396.5 (202 to 669.5) |
| Timor-Leste | 52.9 (32.1 to 82.2) | 37.8 (22.8 to 58.6) | 716.4 (441.8 to 1083.5) | 688.2 (423.7 to 1042.1) | 28.2 (15.8 to 44.5) | 11.9 (6.9 to 19.5) | 5.9 (3.2 to 9.6) | 141.8 (79 to 232.5) | 135.5 (75.2 to 225) | 6.3 (3.6 to 10.8) | 9.7 (5.6 to 14.8) | 4.6 (2.7 to 7.4) | 162.2 (92.2 to 248.9) | 158.3 (89.9 to 243.5) | 3.9 (2 to 6.7) | 3.4 (1.8 to 5.7) | 1.2 (0.7 to 1.9) | 56.5 (32.9 to 90.8) | 55.3 (31.9 to 89.5) | 1.1 (0.5 to 2.2) |
| Tonga | 16 (10.1 to 23.1) | 10.2 (6.3 to 14.4) | 182.2 (113.7 to 257.6) | 173.8 (108.7 to 247) | 8.4 (4.8 to 13.2) | 1.8 (1 to 2.8) | 0.8 (0.5 to 1.2) | 19.3 (11.6 to 29.2) | 18.4 (10.9 to 27.8) | 0.9 (0.5 to 1.5) | 0.7 (0.3 to 1.1) | 0.3 (0.2 to 0.5) | 9.2 (4.5 to 14.8) | 8.9 (4.4 to 14.4) | 0.3 (0.1 to 0.4) | 0.1 (0.1 to 0.2) | 0 (0 to 0.1) | 1.4 (0.8 to 2.2) | 1.3 (0.8 to 2.1) | 0 (0 to 0.1) |
| Trinidad and Tobago | 1177.9 (939.3 to 1500.3) | 525.7 (441.3 to 609.7) | 9922.6 (8291 to 11529.7) | 9241.3 (7788.6 to 10781.4) | 681.3 (480.1 to 949.1) | 57.4 (45.8 to 70.6) | 27.9 (22.8 to 34.6) | 616.7 (502.3 to 762.6) | 588.3 (480.1 to 728) | 28.4 (18.9 to 41.8) | 41.7 (33.5 to 51.6) | 21.4 (17.4 to 26) | 610.2 (496.3 to 742) | 593.6 (481.9 to 725.5) | 16.6 (11 to 23.2) | 11.4 (8 to 15.7) | 2.1 (1.7 to 2.6) | 94.4 (75.2 to 116.6) | 88.9 (70.4 to 109.5) | 5.5 (3 to 9.2) |
| Tunisia | 3271.9 (1999.4 to 5452.8) | 1107.4 (723.1 to 1759.6) | 22679.2 (14721.4 to 35793.1) | 20302.3 (13245.8 to 32299.6) | 2376.9 (1244.9 to 4017.5) | 1252.8 (830.5 to 1724) | 505.8 (354.9 to 707.9) | 11639.9 (8069.7 to 15987) | 10880.1 (7557.7 to 15005.6) | 759.9 (453.6 to 1154.3) | 342 (225.9 to 508.5) | 158.4 (104.6 to 228.4) | 4261.3 (2818.8 to 6219.1) | 4102.6 (2729 to 5971.7) | 158.7 (85.8 to 266.2) | 110.2 (59.1 to 185.8) | 11 (6.9 to 16.4) | 492.8 (300.1 to 763.4) | 432.8 (269.8 to 652.6) | 60 (27 to 121.7) |
| Türkiye | 16186 (11160.1 to 22827.8) | 6368.4 (4480.9 to 8409.1) | 116694.7 (83910.8 to 151753.1) | 106954.4 (76937.7 to 139508.8) | 9740.3 (5970.9 to 15112.9) | 7904.1 (5565.2 to 10434.6) | 3318.5 (2360.6 to 4312.3) | 72500.5 (51917.2 to 93813.4) | 68482.5 (49131.2 to 89767) | 4018 (2496.5 to 6009.9) | 2937.7 (1897 to 4229.4) | 1353.6 (884.3 to 1943.9) | 36890.2 (23958.9 to 51941.4) | 35709.9 (23137.9 to 50491) | 1180.3 (688 to 1883.9) | 2667.8 (1793.3 to 3830) | 228.5 (183.6 to 284.3) | 11394.6 (8782.8 to 14286.1) | 10040.7 (7810 to 12439.6) | 1354 (773.9 to 2209.5) |
| Turkmenistan | 143.9 (111.7 to 187.6) | 75.4 (60.8 to 89.6) | 1889.3 (1499.9 to 2297.2) | 1797.3 (1435 to 2164.5) | 92 (60.1 to 135) | 71.7 (55.6 to 87.4) | 37.1 (28.8 to 45.1) | 1019.7 (795.1 to 1248.9) | 982.1 (769.1 to 1197.2) | 37.5 (23.1 to 53.6) | 166.9 (130.1 to 215.8) | 95.1 (75.7 to 119.7) | 3156.1 (2514 to 3964) | 3093.8 (2470.3 to 3890.5) | 62.4 (41.5 to 91.9) | 30.4 (20.8 to 43.3) | 9.3 (6.9 to 12.1) | 387.3 (287.3 to 513) | 375.6 (279.7 to 496.9) | 11.7 (5.8 to 19.9) |
| Tuvalu | 1.8 (1.1 to 2.5) | 1.2 (0.8 to 1.7) | 24.9 (16.4 to 35.4) | 23.9 (15.7 to 34.1) | 0.9 (0.5 to 1.4) | 0.3 (0.2 to 0.5) | 0.1 (0.1 to 0.2) | 4 (2.4 to 6.2) | 3.9 (2.3 to 6) | 0.2 (0.1 to 0.3) | 0.1 (0.1 to 0.2) | 0.1 (0 to 0.1) | 1.9 (1 to 3.1) | 1.9 (1 to 3) | 0 (0 to 0.1) | 0 (0 to 0) | 0 (0 to 0) | 0.4 (0.2 to 0.6) | 0.4 (0.2 to 0.6) | 0 (0 to 0) |
| Ukraine | 11577.7 (9252.1 to 14013.5) | 4166.9 (3747.3 to 4699.2) | 94147.3 (84938.2 to 105525.2) | 86479.3 (78537.6 to 97005.3) | 7668 (5377.2 to 10509) | 3745 (3116.2 to 4535.4) | 1817.2 (1546 to 2136.8) | 42570 (36058.3 to 50137.1) | 40757.1 (34637 to 48227.8) | 1813 (1247.2 to 2468.2) | 3305.4 (2837.8 to 3970.9) | 1739.9 (1495.1 to 2106.6) | 47950.9 (41441.3 to 57723.1) | 46755.3 (40322.5 to 56409.3) | 1195.6 (845.6 to 1676.3) | 565.3 (386.4 to 763.2) | 131.2 (106.7 to 159.3) | 5784.3 (4646.6 to 7065.1) | 5556 (4505 to 6749.4) | 228.3 (120.4 to 378) |
| United Arab Emirates | 306.7 (175.3 to 513) | 109 (66.2 to 171.1) | 2526.8 (1552.9 to 3956.5) | 2327.6 (1441.2 to 3663) | 199.3 (107 to 343.5) | 127.7 (80.9 to 189.4) | 46.2 (29.2 to 67.2) | 1362.8 (856.9 to 1971.6) | 1276.6 (808.7 to 1849.1) | 86.2 (50.4 to 132.9) | 56.2 (34.6 to 90.7) | 24.5 (15.4 to 37.9) | 839.4 (523 to 1347.9) | 814.2 (509.4 to 1307) | 25.2 (12.9 to 43.8) | 20.9 (11 to 38.1) | 2.4 (1.4 to 3.8) | 125.8 (74.8 to 198.9) | 113.4 (66.8 to 178.6) | 12.4 (5.8 to 24.9) |
| United Kingdom | 53892.5 (46162.5 to 61977.5) | 16800.6 (15121.2 to 18465.5) | 272047 (246572.1 to 296230.4) | 235951.9 (214890 to 257737.9) | 36095.2 (25451.7 to 48462.1) | 11873.2 (10300.5 to 13465.5) | 5317.5 (4753.9 to 5801.8) | 86051.9 (78074.8 to 93137.7) | 80364.8 (73262.3 to 87722.4) | 5687.1 (4016.2 to 7720.4) | 7057.4 (6080.5 to 8063.6) | 3290.1 (2975.4 to 3626.7) | 68739.3 (62861.3 to 75517) | 66007.7 (60370.4 to 72298.4) | 2731.6 (1946.6 to 3752) | 2556 (1863.5 to 3429.5) | 99.9 (84.3 to 119.7) | 4973.3 (4050.2 to 6224.9) | 3612.4 (3050.2 to 4358.3) | 1360.9 (812.2 to 2117.8) |
| United Republic of Tanzania | 5665.7 (3851.2 to 8480.8) | 4416.1 (2919.8 to 6609.2) | 86798.5 (60008.8 to 128820.9) | 84085.7 (58025.4 to 125251.8) | 2712.8 (1605.6 to 4234.8) | 432.4 (275.9 to 634) | 271.7 (168.5 to 411.9) | 6369 (4044 to 9566.8) | 6176.9 (3898.9 to 9251.6) | 192.1 (108.7 to 302.1) | 127.1 (78.7 to 185.7) | 76.4 (44.3 to 113) | 2775.4 (1678.4 to 4009.8) | 2730.4 (1651.9 to 3950.6) | 45 (25.2 to 73.7) | 103 (52 to 173.5) | 35.2 (18.7 to 55.7) | 1964 (1040.5 to 3099.7) | 1934 (1031.4 to 3051.2) | 30 (13.4 to 53.9) |
| United States of America | 290194.6 (235815 to 347463) | 44824.3 (38495.9 to 50728) | 917787.8 (799662.2 to 1034277.1) | 716852.3 (625449 to 808104.7) | 200935.5 (139050.8 to 274249.9) | 64058.2 (55889.8 to 71962.3) | 15466.8 (13880.4 to 16899.4) | 293124.3 (268031.9 to 320440) | 258152.1 (235838.2 to 281378.1) | 34972.2 (25922.8 to 46003.6) | 38817.2 (33067.9 to 44903.5) | 11829.5 (10613.4 to 13068.3) | 270099 (244862.2 to 298993) | 253443.4 (231018 to 280228.5) | 16655.6 (11374.7 to 22234.5) | 12125.4 (8403.7 to 15987.7) | 590.8 (494.1 to 731.7) | 31866.6 (25575.9 to 40353.8) | 25377.3 (21204.3 to 31535.1) | 6489.3 (3667.3 to 10479.4) |
| United States Virgin Islands | 97.6 (71.7 to 130.9) | 36.7 (28.8 to 45.5) | 641.7 (511 to 794.8) | 588.5 (465.4 to 725.1) | 53.3 (35.9 to 76.4) | 4.2 (3.1 to 5.5) | 2 (1.6 to 2.5) | 39.5 (31.6 to 49.1) | 37.5 (29.7 to 46.5) | 2 (1.3 to 2.9) | 6.6 (5.2 to 8.5) | 3.1 (2.5 to 3.8) | 81 (66.6 to 98.3) | 78.5 (64.3 to 95) | 2.6 (1.7 to 3.7) | 0.6 (0.4 to 0.9) | 0.1 (0.1 to 0.1) | 3.5 (2.8 to 4.5) | 3.2 (2.5 to 4.1) | 0.3 (0.2 to 0.5) |
| Uruguay | 1668.7 (1375.4 to 2030.1) | 806 (725.6 to 881.6) | 13911.6 (12687.3 to 15133.1) | 12854.1 (11727.9 to 13976) | 1057.6 (710.1 to 1408.9) | 467.4 (398.9 to 546) | 231.2 (202 to 265.1) | 4385.3 (3830.9 to 4946.1) | 4168.8 (3648.8 to 4762.5) | 216.5 (147.2 to 298.5) | 421.2 (359.7 to 487.9) | 229.5 (206.8 to 256) | 5629.2 (4998.8 to 6302.9) | 5476 (4864.6 to 6120.8) | 153.2 (104.8 to 212.1) | 209.1 (135.1 to 293.2) | 18.9 (15.4 to 22.5) | 1006.7 (804.7 to 1219.7) | 901.7 (730.3 to 1070.5) | 105 (58.4 to 162.2) |
| Uzbekistan | 947.7 (774.4 to 1178.7) | 457.8 (395.3 to 528.1) | 10589.7 (9103.5 to 12295.4) | 10002.2 (8614.9 to 11578) | 587.5 (386.5 to 838) | 478.8 (386.6 to 588) | 244.7 (204.1 to 289.6) | 6227.3 (5181.5 to 7382.5) | 5978.4 (4945.2 to 7076.4) | 248.9 (161.1 to 342.6) | 512.4 (424.8 to 608.3) | 271.3 (230.7 to 313.2) | 8759.6 (7446.2 to 10088.5) | 8557.9 (7258 to 9851.8) | 201.7 (131.3 to 295.4) | 275.8 (183.3 to 372.6) | 67.8 (54.7 to 82.2) | 2749.9 (2198.1 to 3356.3) | 2632.7 (2127.2 to 3217.2) | 117.2 (61.3 to 190.2) |
| Vanuatu | 14.8 (9.4 to 22.1) | 11.1 (7.2 to 16.7) | 225.7 (143.9 to 344.9) | 217.7 (139.3 to 334) | 8 (4.5 to 12.5) | 3.1 (1.8 to 5) | 1.4 (0.8 to 2.2) | 41.7 (24.7 to 66.9) | 39.9 (23.5 to 64) | 1.8 (1 to 3) | 1.4 (0.7 to 2.4) | 0.8 (0.4 to 1.3) | 25.5 (11.7 to 43.3) | 24.9 (11.5 to 42.2) | 0.6 (0.2 to 1) | 0.2 (0.1 to 0.4) | 0.1 (0 to 0.2) | 3.7 (2 to 6.4) | 3.7 (2 to 6.3) | 0.1 (0 to 0.1) |
| Viet Nam | 2672.7 (1110.3 to 4904.9) | 1074.8 (441 to 1862.9) | 22668.9 (9795.9 to 39107.3) | 20915.4 (8877.7 to 36225.2) | 1753.4 (711.1 to 3468) | 2545.8 (1571.7 to 4009.3) | 945.9 (564.5 to 1456.6) | 23598.5 (14247.8 to 35993) | 22161.3 (13308.6 to 34352.6) | 1437.2 (777.2 to 2365.8) | 899 (479 to 1464.5) | 347.8 (184.9 to 555.4) | 10415.5 (5553.9 to 16663.5) | 10023.8 (5320 to 16097.3) | 391.6 (193.9 to 700.5) | 882.7 (461.6 to 1512.3) | 116.4 (70.8 to 176.2) | 6049.7 (3558.7 to 8991.3) | 5622.4 (3325.2 to 8582.5) | 427.4 (184.4 to 818.7) |
| Zambia | 1598.9 (1041.3 to 2310.9) | 1165.1 (743 to 1687.6) | 25809.6 (16772.8 to 37374.9) | 25044.8 (16231.8 to 36269.7) | 764.8 (449.4 to 1156.2) | 185.1 (114.5 to 274.1) | 100.8 (62.6 to 147.4) | 2899.1 (1777.3 to 4303.5) | 2819.5 (1726 to 4182.8) | 79.7 (44.4 to 130.8) | 67.2 (41.6 to 99) | 34.5 (20.5 to 51.2) | 1583.3 (981.9 to 2283.3) | 1563.1 (966.9 to 2250) | 20.2 (10.5 to 33.1) | 43 (22.3 to 71.2) | 15.3 (8.3 to 23.5) | 856.3 (461.4 to 1330.1) | 843.7 (452.5 to 1307) | 12.6 (5.9 to 23.2) |
| Zimbabwe | 962.4 (641.9 to 1427.7) | 786.4 (501.4 to 1194.2) | 15895 (10428 to 23654.9) | 15380.3 (10069.6 to 22854.6) | 514.7 (317.1 to 811.8) | 241.1 (145.5 to 354.2) | 152.2 (90.4 to 227) | 3751.6 (2216.9 to 5598.1) | 3641.7 (2134.7 to 5433.6) | 110 (62.8 to 177.7) | 46.5 (29.6 to 69.5) | 28.9 (18.4 to 43.3) | 1105.4 (708.4 to 1613.5) | 1088.8 (699.1 to 1590.7) | 16.6 (9.3 to 27.2) | 23 (12.4 to 36) | 12.7 (7.6 to 20) | 499.8 (283.8 to 764.8) | 493.3 (280 to 756) | 6.6 (3.1 to 12.3) |

## Table S5 Age-standardized incidence, deaths, DALYs, YLDs, and YLDs rate of Prostate, Male bladder, Male kidney, and Testicular Cancer in 2023

| **Locations** | **Prostate cancer** | | | | | **Male bladder cancer** | | | | | **Male kidney cancer** | | | | | **Testicular cancer** | | | | |
| --- | --- | --- | --- | --- | --- | --- | --- | --- | --- | --- | --- | --- | --- | --- | --- | --- | --- | --- | --- | --- |
|  | ASIR and 95%UI | ASMR and 95%UI | ASDR and 95%UI | Age-Standardized YLL Rates and 95%UI | Age-Standardized YLD Rates and 95%UI | ASIR and 95%UI | ASMR and 95%UI | ASDR and 95%UI | Age-Standardized YLL Rates and 95%UI | Age-Standardized YLD Rates and 95%UI | ASIR and 95%UI | ASMR and 95%UI | ASDR and 95%UI | Age-Standardized YLL Rates and 95%UI | Age-Standardized YLD Rates and 95%UI | ASIR and 95%UI | ASMR and 95%UI | ASDR and 95%UI | Age-Standardized YLL Rates and 95%UI | Age-Standardized YLD Rates and 95%UI |
| World Bank High Income | 70.0 (59.3 to 81.4) | 16.7 (14.9 to 18.0) | 301.7 (272.4 to 326.6) | 254.5 (231.5 to 273.7) | 47.2 (34.2 to 63.8) | 18.9 (16.8 to 21.1) | 6.6 (6.0 to 7.0) | 119.8 (111.6 to 128.7) | 109.8 (103.0 to 117.4) | 9.9 (7.2 to 13.1) | 12.9 (11.3 to 14.5) | 4.8 (4.4 to 5.1) | 109.5 (102.8 to 116.9) | 104.2 (97.8 to 111.3) | 5.4 (3.8 to 7.3) | 6.8 (5.0 to 8.7) | 0.30 (0.28 to 0.34) | 17.1 (20.0 to 15.0) | 13.5 (12.3 to 15.0) | 3.6 (2.1 to 5.5) |
| World Bank Upper Middle Income | 19.7 (15.5 to 23.9) | 9.0 (7.6 to 10.0) | 157.0 (132.9 to 176.0) | 145.8 (123.2 to 161.8) | 11.2 (7.5 to 15.8) | 7.9 (6.8 to 9.1) | 3.6 (3.2 to 4.0) | 71.3 (64.6 to 79.3) | 67.3 (60.8 to 75.3) | 4.0 (2.8 to 5.4) | 4.7 (3.8 to 6.2) | 2.0 (1.6 to 2.5) | 54.9 (44.9 to 68.1) | 52.9 (43.2 to 66.0) | 1.9 (1.3 to 3.0) | 2.3 (1.7 to 3.0) | 0.32 (0.28 to 0.36) | 15.9 (14.1 to 18.0) | 14.8 (13.1 to 16.7) | 1.1 (0.7 to 1.7) |
| World Bank Lower Middle Income | 14.3 (10.0 to 19.6) | 10.3 (7.1 to 14.1) | 182.8 (128.6 to 250.0) | 175.6 (123.7 to 239.1) | 7.2 (4.6 to 10.8) | 4.3 (2.9 to 6.2) | 2.5 (1.7 to 3.5) | 52.4 (35.3 to 72.4) | 50.4 (33.6 to 69.6) | 2.0 (1.2 to 2.9) | 1.7 (1.2 to 2.5) | 1.1 (0.7 to 1.5) | 29.8 (20.1 to 42.2) | 29.2 (19.6 to 41.4) | 0.6 (0.4 to 1.0) | 0.9 (0.5 to 1.4) | 0.24 (0.15 to 0.35) | 11.3 (6.8 to 16.9) | 10.9 (6.6 to 16.3) | 0.4 (0.2 to 0.7) |
| Albania | 31.1 (21.5 to 42.2) | 16.1 (11.2 to 20.8) | 267.4 (192.1 to 347.1) | 250.1 (178.2 to 326) | 17.3 (10.3 to 24.3) | 1 (0.6 to 1.4) | 0.5 (0.3 to 0.8) | 9.9 (6.2 to 14) | 9.4 (5.8 to 13.3) | 0.6 (0.3 to 0.9) | 6.5 (4.3 to 10.2) | 3.4 (2.4 to 5.4) | 81.8 (55.4 to 126) | 79.3 (54.4 to 122.1) | 2.5 (1.4 to 4.5) | 5.3 (2.9 to 8.4) | 0.4 (0.3 to 0.7) | 22.6 (13.7 to 34.2) | 20.1 (12.1 to 30) | 2.5 (1.1 to 5) |
| Algeria | 6.2 (3 to 10) | 3.1 (1.5 to 5) | 52.6 (25.4 to 84.3) | 49.3 (23.9 to 79.1) | 3.3 (1.6 to 5.8) | 6.6 (3.6 to 10.3) | 3.3 (1.7 to 5.5) | 64.8 (34.5 to 107.7) | 61.8 (32.1 to 102.4) | 3.1 (1.6 to 5.2) | 1.3 (0.7 to 2) | 0.7 (0.3 to 1.1) | 17.1 (9.2 to 27) | 16.6 (8.9 to 26.3) | 0.5 (0.2 to 0.8) | 2.9 (1.5 to 4.9) | 0.3 (0.2 to 0.4) | 15 (8.9 to 23.3) | 13.6 (8.1 to 20.9) | 1.4 (0.6 to 2.9) |
| American Samoa | 80.1 (52.8 to 113.4) | 62.3 (41.3 to 87.7) | 995.2 (659.4 to 1385.9) | 955.8 (631.7 to 1338.6) | 39.4 (23.2 to 60) | 5.6 (3.5 to 8.4) | 2.9 (1.7 to 4.3) | 63.7 (38.7 to 95.3) | 60.9 (36.3 to 91.8) | 2.9 (1.7 to 4.5) | 2.5 (1.6 to 3.8) | 1.4 (0.9 to 2.2) | 36.6 (23.7 to 54.8) | 35.6 (23.1 to 53.4) | 0.9 (0.5 to 1.5) | 0.1 (0 to 0.1) | 0 (0 to 0.1) | 1.1 (0.6 to 1.7) | 1.1 (0.6 to 1.6) | 0 (0 to 0) |
| Andorra | 65.6 (41.8 to 95.3) | 14.8 (10.3 to 20.1) | 266.2 (184 to 361.3) | 222.8 (156.3 to 302.4) | 43.4 (24.9 to 67.4) | 19.9 (12.9 to 28.8) | 6.8 (4.3 to 10) | 132.9 (86.3 to 192) | 122.4 (79.6 to 176.4) | 10.4 (5.8 to 16.4) | 8.7 (5.5 to 13.6) | 3 (2 to 4.8) | 74.5 (49.3 to 113.4) | 70.8 (47.2 to 108.3) | 3.7 (2 to 6.4) | 22 (11.4 to 35.6) | 0.4 (0.3 to 0.7) | 33 (18.5 to 51.7) | 22.2 (12.7 to 33.8) | 10.7 (5.3 to 19.3) |
| Angola | 59.6 (40.1 to 83.2) | 48.6 (33.6 to 68.4) | 937.9 (642.3 to 1309.5) | 909.8 (621 to 1272) | 28 (16.9 to 42.3) | 6.1 (3.9 to 8.6) | 4.4 (2.8 to 6.1) | 88.4 (56.8 to 123.7) | 86.1 (55.2 to 120.6) | 2.3 (1.4 to 3.7) | 1.5 (0.9 to 2.3) | 1.1 (0.6 to 1.7) | 32.2 (18.6 to 49.3) | 31.7 (18.3 to 48.5) | 0.5 (0.3 to 0.8) | 0.7 (0.4 to 1.1) | 0.3 (0.2 to 0.5) | 14.6 (7.9 to 22.8) | 14.4 (7.8 to 22.6) | 0.2 (0.1 to 0.3) |
| Antigua and Barbuda | 145.1 (107.9 to 194) | 63 (52.3 to 76.8) | 1095.5 (905.6 to 1310.6) | 1007.2 (833.3 to 1205.9) | 88.3 (58.3 to 124.5) | 5.2 (4 to 6.4) | 2.6 (2.1 to 3.2) | 51.6 (42 to 63.3) | 49 (39.7 to 60.4) | 2.6 (1.7 to 3.8) | 3.3 (2.6 to 4.1) | 1.6 (1.3 to 1.9) | 44 (35.3 to 52.8) | 42.7 (34.4 to 51.4) | 1.4 (0.9 to 1.9) | 1.3 (0.8 to 1.7) | 0.2 (0.1 to 0.2) | 8.2 (6.5 to 10.2) | 7.6 (6 to 9.4) | 0.6 (0.3 to 1.1) |
| Argentina | 41.5 (34 to 50.7) | 22.4 (19.2 to 25.5) | 382.1 (331.3 to 431.2) | 356.8 (308.2 to 404.6) | 25.3 (17.1 to 34.2) | 10.8 (9.3 to 12.6) | 5.5 (4.9 to 6.3) | 107.2 (94.5 to 121) | 102.1 (89.8 to 115.6) | 5 (3.3 to 6.9) | 13.6 (12.1 to 15.3) | 7.7 (7 to 8.3) | 196.4 (180.1 to 213.9) | 191.4 (175.3 to 209.1) | 4.9 (3.5 to 6.6) | 11.6 (8.1 to 15.7) | 1.2 (1.1 to 1.4) | 67 (58.3 to 76.8) | 61.1 (54.2 to 69.3) | 5.9 (3.3 to 9.6) |
| Armenia | 21.5 (17.2 to 27) | 10.7 (9.2 to 12.4) | 200.3 (173.9 to 228.3) | 186.9 (162.4 to 215) | 13.4 (9.2 to 18.3) | 12.3 (9.9 to 15.1) | 6.6 (5.6 to 7.7) | 137.9 (115.6 to 162.8) | 131.6 (111 to 155.6) | 6.3 (4.2 to 8.7) | 6 (4.9 to 7.2) | 3.2 (2.7 to 3.8) | 85.2 (70.3 to 99.8) | 82.8 (68.4 to 97.1) | 2.4 (1.6 to 3.3) | 2.6 (1.8 to 3.7) | 0.5 (0.4 to 0.6) | 17.3 (13.8 to 21) | 16.1 (13.1 to 19.7) | 1.2 (0.7 to 2.1) |
| Australia | 98.9 (84.3 to 115) | 19.4 (17.1 to 21.4) | 343.7 (303.5 to 382) | 276.6 (247.6 to 304.4) | 67.1 (48.8 to 91.3) | 11.5 (10.3 to 12.9) | 4.4 (4 to 4.8) | 73.6 (67.2 to 79.8) | 67.7 (62.5 to 73.1) | 5.9 (4.2 to 8.1) | 13 (11.1 to 14.9) | 4.2 (3.8 to 4.6) | 96.1 (86.5 to 105.5) | 90.4 (81.6 to 98.5) | 5.7 (4 to 7.9) | 9.3 (6.8 to 12.2) | 0.3 (0.2 to 0.3) | 16 (12.4 to 20.4) | 11.1 (9.1 to 13.5) | 5 (2.9 to 8.1) |
| Austria | 68.3 (53.3 to 86.5) | 19.2 (16.8 to 21.2) | 325.2 (281.4 to 362.5) | 279.5 (244.3 to 309.9) | 45.7 (30.5 to 63) | 15.3 (12.9 to 18) | 6.3 (5.6 to 7) | 109 (96.5 to 121.8) | 101.3 (89.4 to 112.5) | 7.8 (5.5 to 10.4) | 10.8 (9 to 12.8) | 4.7 (4.2 to 5.4) | 99.7 (88.2 to 114.8) | 95.3 (84.4 to 110) | 4.3 (3 to 6) | 11.3 (7.7 to 14.8) | 0.3 (0.3 to 0.4) | 20.5 (16.4 to 26.1) | 14.4 (11.8 to 17.8) | 6.1 (3.4 to 9.8) |
| Azerbaijan | 15.4 (10.7 to 20.4) | 9.1 (6.9 to 12.3) | 180.8 (135.2 to 235.1) | 171.7 (128.3 to 226.5) | 9 (5.9 to 13.3) | 6.2 (4.1 to 8.9) | 3.6 (2.3 to 5.1) | 80.2 (52.8 to 113.6) | 77.2 (50.7 to 110) | 3 (1.8 to 4.5) | 6.7 (4.7 to 9.9) | 4 (2.6 to 5.8) | 112.9 (73.9 to 160) | 110.4 (72.1 to 157.1) | 2.5 (1.4 to 4.2) | 0.9 (0.5 to 1.6) | 0.3 (0.1 to 0.4) | 9.4 (5.5 to 14.8) | 9.1 (5.2 to 14.1) | 0.4 (0.1 to 0.7) |
| Bahamas | 156.1 (124.5 to 198) | 82.3 (69.3 to 95.7) | 1366.3 (1162.7 to 1593) | 1280.2 (1080.3 to 1487.5) | 86.1 (59.4 to 123.4) | 5.7 (4.6 to 7) | 3 (2.5 to 3.7) | 60.3 (49.5 to 74.1) | 57.7 (47.7 to 71) | 2.7 (1.8 to 3.7) | 5.6 (4.6 to 6.9) | 2.7 (2.3 to 3.3) | 82 (69.6 to 97.2) | 79.8 (67.6 to 95.1) | 2.2 (1.5 to 3.1) | 0.4 (0.3 to 0.5) | 0.1 (0 to 0.1) | 2.9 (2.3 to 3.6) | 2.8 (2.1 to 3.4) | 0.2 (0.1 to 0.3) |
| Bahrain | 37.2 (24.2 to 51.5) | 19.7 (13.5 to 25.8) | 295.6 (205.2 to 393.5) | 276.9 (191 to 370) | 18.7 (11.2 to 29.4) | 16.6 (11.1 to 24.8) | 8.5 (5.6 to 12.4) | 151.3 (100.5 to 218.2) | 144.2 (94.8 to 207.9) | 7.1 (4.2 to 10.8) | 4 (2.5 to 5.9) | 2.2 (1.3 to 3.1) | 48.1 (30.1 to 68.2) | 46.7 (29.2 to 65.9) | 1.4 (0.8 to 2.3) | 0.6 (0.3 to 0.9) | 0.1 (0 to 0.1) | 2.6 (1.6 to 3.8) | 2.3 (1.4 to 3.4) | 0.3 (0.1 to 0.6) |
| Bangladesh | 11.8 (7 to 20.5) | 8.2 (5 to 14.2) | 144.9 (89.1 to 253.8) | 139.1 (85.4 to 243.1) | 5.8 (2.9 to 10) | 4.4 (2.4 to 7.8) | 2.6 (1.3 to 4.5) | 55 (28.9 to 94.7) | 53 (27.6 to 91.7) | 2 (1.1 to 3.3) | 1.6 (0.9 to 3) | 1.1 (0.6 to 1.9) | 28.5 (15.9 to 52.3) | 28 (15.7 to 51) | 0.6 (0.3 to 1.1) | 1.2 (0.6 to 2.1) | 0.3 (0.1 to 0.4) | 13.5 (7.6 to 21.8) | 13 (7.2 to 21) | 0.5 (0.2 to 1.1) |
| Barbados | 146.5 (111.4 to 191.3) | 60.5 (49.9 to 73.5) | 1035.9 (865.3 to 1251.4) | 952.1 (796.1 to 1157.9) | 83.8 (56 to 116.8) | 7.9 (6.3 to 9.6) | 4 (3.3 to 4.8) | 75.9 (61.7 to 92) | 72.2 (59.5 to 87.7) | 3.7 (2.4 to 5.2) | 7.9 (6.4 to 10.1) | 3.7 (3.1 to 4.6) | 101.1 (84.1 to 123.4) | 97.9 (81.8 to 120.6) | 3.1 (2.1 to 4.4) | 0.9 (0.6 to 1.3) | 0.1 (0.1 to 0.1) | 5.4 (4.4 to 6.7) | 5 (4 to 6.1) | 0.5 (0.2 to 0.8) |
| Belarus | 87.8 (68.7 to 110.2) | 21.8 (20.1 to 23.6) | 448.9 (409.9 to 494.7) | 389.5 (358.7 to 426.3) | 59.5 (40.2 to 84) | 13.4 (11.4 to 15.7) | 6.3 (5.6 to 7.2) | 131.5 (115.6 to 149) | 125 (110.6 to 142.5) | 6.5 (4.4 to 8.6) | 15 (12.9 to 17.8) | 6.1 (5.4 to 6.8) | 162.2 (144.9 to 183) | 156.2 (138.9 to 175.3) | 6 (4.2 to 8.6) | 3.7 (2.4 to 5) | 0.4 (0.3 to 0.5) | 15.8 (12.8 to 19) | 13.9 (11.3 to 16.7) | 1.9 (1 to 3) |
| Belgium | 58.6 (44.5 to 73.4) | 18.3 (16 to 20.3) | 299.8 (259.5 to 334.5) | 260.7 (229.6 to 289.2) | 39.1 (25.5 to 54.2) | 17.5 (14.8 to 20.9) | 7.3 (6.5 to 8.3) | 128.2 (113.6 to 145.8) | 119.2 (106.4 to 135.7) | 9 (6.3 to 12.6) | 10.4 (9.1 to 11.8) | 4.6 (4.2 to 4.8) | 100.3 (93 to 106) | 96.2 (89.7 to 101.4) | 4.2 (2.9 to 5.9) | 5.6 (3.7 to 7.6) | 0.2 (0.2 to 0.2) | 10.9 (8.5 to 13.8) | 7.9 (6.6 to 9.6) | 3 (1.6 to 5.1) |
| Belize | 68.7 (54.4 to 87.2) | 38.3 (32.5 to 46) | 629.5 (535.5 to 748.1) | 592.9 (506.4 to 705) | 36.5 (24.6 to 52.3) | 4.4 (3.5 to 5.4) | 2.4 (2 to 3) | 48 (38.9 to 58.9) | 46 (37.1 to 56.3) | 2.1 (1.4 to 3) | 3.9 (3.1 to 4.8) | 2 (1.6 to 2.4) | 59.7 (48.5 to 73.6) | 58.2 (47.3 to 71.7) | 1.6 (1 to 2.3) | 1.5 (1 to 2) | 0.3 (0.2 to 0.3) | 12.8 (10.3 to 16.3) | 12.2 (9.7 to 15.4) | 0.7 (0.3 to 1.2) |
| Benin | 45.9 (30.4 to 67.1) | 38.6 (24.9 to 57.1) | 694 (459.3 to 1024.6) | 673.2 (444 to 995.7) | 20.8 (12.7 to 33.1) | 2.7 (1.7 to 4.2) | 1.8 (1.2 to 2.9) | 39.3 (25.4 to 60) | 38.1 (24.7 to 58) | 1.2 (0.7 to 1.9) | 3.1 (1.9 to 4.8) | 2 (1.1 to 3.2) | 64.2 (39 to 97.7) | 63.2 (38.3 to 95.7) | 1 (0.6 to 1.7) | 0.6 (0.3 to 1) | 0.2 (0.1 to 0.4) | 11.5 (6.6 to 17.8) | 11.3 (6.5 to 17.6) | 0.2 (0.1 to 0.3) |
| Bermuda | 170.6 (127 to 226.5) | 43.4 (36.9 to 51.1) | 730.6 (606.3 to 872.8) | 627.3 (530.2 to 736.4) | 103.4 (68.6 to 150.1) | 20.3 (15.7 to 24.8) | 8 (6.5 to 9.6) | 149.8 (121.7 to 180.1) | 139.4 (113 to 166.8) | 10.4 (7 to 14.7) | 9.3 (7.5 to 11.3) | 3 (2.4 to 3.6) | 83.8 (67.3 to 100) | 79.7 (64.7 to 95.9) | 4.1 (2.8 to 5.8) | 3.3 (2.3 to 4.6) | 0.1 (0.1 to 0.2) | 8.4 (6.8 to 10.3) | 6.7 (5.7 to 7.7) | 1.8 (1 to 2.9) |
| Bhutan | 6.4 (3.7 to 10.2) | 4.8 (2.6 to 7.5) | 83.5 (46.7 to 130.6) | 80.4 (44.8 to 125.7) | 3.1 (1.4 to 5.4) | 2.2 (1.1 to 3.8) | 1.4 (0.7 to 2.4) | 28.3 (13.9 to 49) | 27.3 (13.3 to 47.5) | 1 (0.5 to 1.6) | 1.3 (0.7 to 2.4) | 0.9 (0.5 to 1.6) | 24.2 (12.8 to 41.9) | 23.7 (12.7 to 40.8) | 0.4 (0.2 to 0.9) | 0.9 (0.5 to 1.7) | 0.2 (0.1 to 0.4) | 11.7 (6.3 to 21) | 11.4 (6.1 to 20.3) | 0.3 (0.1 to 0.7) |
| Bolivia (Plurinational State of) | 89.2 (60.4 to 127.4) | 41.7 (29.3 to 56.7) | 735.5 (515.1 to 1009) | 687.4 (482.5 to 933.5) | 48 (27.9 to 73.5) | 7.5 (4.6 to 12) | 3.5 (2.1 to 5.4) | 69.4 (42.4 to 106.6) | 65.9 (40.6 to 101.4) | 3.5 (1.9 to 5.7) | 6.3 (4 to 9.6) | 3 (1.9 to 4.5) | 81.8 (53.5 to 121.8) | 79.4 (52.2 to 119) | 2.4 (1.3 to 3.8) | 2.8 (1.5 to 4.4) | 0.8 (0.5 to 1.3) | 33.6 (19.2 to 51.5) | 32.6 (18.7 to 49.9) | 1.1 (0.5 to 2) |
| Bosnia and Herzegovina | 32.3 (21.8 to 43.7) | 15.9 (10.3 to 21.1) | 281.7 (185.5 to 368.3) | 263.5 (172.5 to 344.6) | 18.1 (10.9 to 27.9) | 14.8 (11.2 to 19.4) | 7.9 (6.1 to 10.1) | 156 (121.9 to 199.3) | 149.4 (116.1 to 189) | 6.6 (4.2 to 9.5) | 8.1 (5 to 10.9) | 4.3 (2.6 to 5.9) | 106.2 (63.9 to 141.8) | 103.3 (62 to 138.1) | 3 (1.6 to 4.6) | 5.8 (3.4 to 9.2) | 0.6 (0.4 to 0.8) | 28.4 (18.9 to 41.2) | 25.3 (17.1 to 35.8) | 3 (1.5 to 5.3) |
| Botswana | 15.3 (10.3 to 22.4) | 11.2 (7.8 to 16.4) | 208.8 (142.4 to 309) | 202 (136.9 to 299.6) | 6.8 (4.1 to 10.4) | 1.1 (0.7 to 1.8) | 0.7 (0.4 to 1.1) | 15 (9.3 to 23.8) | 14.6 (9 to 23.1) | 0.4 (0.3 to 0.7) | 0.9 (0.5 to 1.3) | 0.6 (0.4 to 0.9) | 17.1 (10.2 to 25.2) | 16.8 (10.1 to 24.8) | 0.3 (0.1 to 0.4) | 1 (0.5 to 1.7) | 0.3 (0.2 to 0.4) | 14.7 (7.8 to 22.8) | 14.3 (7.6 to 22.3) | 0.4 (0.1 to 0.7) |
| Brazil | 45.4 (37.1 to 56) | 20.8 (18.4 to 22.8) | 362.4 (326.7 to 394.3) | 338.8 (305.5 to 368) | 23.7 (16.1 to 32.2) | 6.9 (6.1 to 7.7) | 3.8 (3.5 to 4.1) | 71.6 (66.5 to 76.7) | 68.7 (63.8 to 73.1) | 2.9 (2.1 to 3.9) | 5.5 (4.9 to 6.2) | 3 (2.8 to 3.2) | 77 (72.3 to 81.7) | 75.1 (70.6 to 79.6) | 2 (1.4 to 2.6) | 3.7 (2.8 to 4.9) | 0.5 (0.5 to 0.6) | 27.8 (24.8 to 31.1) | 26.1 (23.2 to 29) | 1.7 (1 to 2.6) |
| Brunei Darussalam | 18.1 (12.8 to 24.5) | 11.6 (8.4 to 15.4) | 189.2 (138.4 to 248.1) | 178.5 (130.7 to 235.8) | 10.7 (6.2 to 15.7) | 6.7 (4.6 to 9.4) | 3.2 (2.2 to 4.5) | 59.6 (40.3 to 83.6) | 56.3 (38.3 to 79) | 3.3 (2 to 5) | 5.7 (3.9 to 8.5) | 3.5 (2.4 to 5.2) | 85.8 (58.6 to 126.8) | 83.7 (57.5 to 124.2) | 2 (1.1 to 3.5) | 1.8 (1 to 3.1) | 0.2 (0.1 to 0.4) | 12.5 (7.4 to 18.9) | 11.6 (6.8 to 17.8) | 0.9 (0.4 to 1.7) |
| Bulgaria | 46.7 (37.4 to 57) | 18 (16.5 to 19.7) | 321.5 (291.2 to 355.4) | 291.4 (266.1 to 320.7) | 30.1 (20.6 to 40.1) | 16 (14 to 18.2) | 7.6 (6.9 to 8.3) | 155.6 (141.9 to 172) | 147.8 (134.6 to 162.8) | 7.7 (5.5 to 10.4) | 9.1 (7.5 to 10.8) | 4.2 (3.6 to 4.7) | 109.2 (94.9 to 123.9) | 105.6 (92.1 to 119.5) | 3.6 (2.4 to 5) | 15.8 (11.1 to 21.4) | 1.2 (1 to 1.4) | 64.1 (52.8 to 77.6) | 55.7 (46.2 to 67.2) | 8.4 (4.5 to 13.5) |
| Cabo Verde | 57.2 (36.7 to 81.5) | 39.9 (24.9 to 59.9) | 667 (430 to 974.3) | 637.2 (411.4 to 939) | 29.8 (17.9 to 47.2) | 7.1 (4.2 to 10.6) | 3.8 (2.2 to 5.7) | 82.7 (49.2 to 124.1) | 79.7 (47.2 to 118.6) | 3 (1.8 to 4.6) | 2.2 (0.6 to 4.4) | 1.2 (0.3 to 2.4) | 37.4 (9.8 to 73.7) | 36.7 (9.6 to 72.4) | 0.7 (0.2 to 1.5) | 0.2 (0.1 to 0.4) | 0.1 (0 to 0.1) | 2.2 (1.1 to 3.5) | 2.2 (1 to 3.4) | 0.1 (0 to 0.1) |
| Cambodia | 9.8 (6 to 15.2) | 7.8 (4.9 to 12.5) | 134.8 (84.3 to 206.8) | 129.8 (80.6 to 200.4) | 5 (3 to 8.3) | 2.7 (1.5 to 4.4) | 1.5 (0.9 to 2.5) | 33 (18.5 to 52.3) | 31.7 (17.8 to 49.9) | 1.3 (0.7 to 2.2) | 1.8 (1 to 2.7) | 1 (0.6 to 1.5) | 29.4 (17.2 to 44.3) | 28.8 (16.7 to 43.4) | 0.7 (0.4 to 1.1) | 0.5 (0.3 to 0.9) | 0.2 (0.1 to 0.3) | 8.7 (5.1 to 13.5) | 8.6 (5 to 13.3) | 0.2 (0.1 to 0.3) |
| Cameroon | 41.9 (24 to 64.2) | 35.1 (19 to 54.7) | 628.9 (343.6 to 984.5) | 610.5 (331.8 to 948.7) | 18.4 (10.1 to 29.4) | 6.7 (4.2 to 10.5) | 4.5 (2.7 to 7.2) | 94 (57.5 to 148.3) | 91.4 (55.8 to 144.3) | 2.5 (1.4 to 4.2) | 1.9 (1.1 to 3) | 1.3 (0.7 to 2) | 40 (22.4 to 62.7) | 39.4 (22.1 to 61.9) | 0.6 (0.3 to 1) | 0.5 (0.3 to 0.9) | 0.2 (0.1 to 0.3) | 10.1 (5.4 to 16.2) | 10 (5.4 to 16) | 0.2 (0.1 to 0.3) |
| Canada | 59.2 (46.6 to 73.9) | 17.2 (15 to 19.2) | 289.6 (252 to 322.9) | 251 (221.1 to 278.3) | 38.5 (25.8 to 53.9) | 13.4 (11.4 to 15.7) | 6 (5.4 to 6.5) | 99.5 (90.4 to 109.2) | 93.1 (85.5 to 101.5) | 6.4 (4.5 to 8.9) | 16.4 (13.1 to 20) | 4.5 (3.9 to 5.1) | 106 (92.4 to 121.7) | 98.6 (85.2 to 113.9) | 7.4 (4.8 to 10.2) | 9 (6.5 to 12.2) | 0.2 (0.2 to 0.3) | 16.2 (13 to 20.7) | 11.4 (9.6 to 13.9) | 4.9 (2.6 to 8) |
| Chile | 51.3 (42.8 to 62.1) | 24.1 (22 to 25.6) | 387.1 (357.2 to 409.7) | 354.8 (328.6 to 374.5) | 32.2 (22 to 44.6) | 7.7 (6.8 to 8.8) | 3.6 (3.3 to 4) | 67.4 (62.1 to 73.7) | 63.5 (58.5 to 69.7) | 3.9 (2.8 to 5.3) | 14 (12.3 to 15.8) | 6.9 (6.5 to 7.4) | 172 (159.8 to 183.9) | 166.5 (154.9 to 178.7) | 5.5 (3.8 to 7.4) | 17.6 (11.7 to 25) | 1.2 (1 to 1.4) | 66.2 (55.3 to 79.8) | 56.9 (48.7 to 66.7) | 9.3 (5.4 to 15.3) |
| China | 8.4 (5.8 to 10.9) | 3.7 (2.5 to 4.7) | 63.2 (45.5 to 76.6) | 58.1 (41.7 to 71.2) | 5.1 (3.2 to 7.5) | 8 (6.5 to 9.6) | 3.4 (2.9 to 4) | 66.6 (57.4 to 76.9) | 62.5 (53 to 72.3) | 4.2 (2.8 to 5.8) | 4.2 (3.2 to 6) | 1.4 (1 to 2) | 41.2 (30.7 to 58) | 39.3 (29 to 55.5) | 1.9 (1.2 to 3.2) | 0.6 (0.4 to 0.9) | 0.1 (0.1 to 0.1) | 4.1 (3.2 to 5.1) | 3.8 (3 to 4.8) | 0.3 (0.2 to 0.5) |
| Colombia | 54.3 (42.9 to 67.5) | 16.7 (14.9 to 18.4) | 296.9 (265.4 to 330.4) | 265.5 (239.4 to 290.9) | 31.4 (20.6 to 44.8) | 3.7 (3.2 to 4.2) | 1.8 (1.6 to 2) | 34.2 (31.1 to 37.3) | 32.3 (29.4 to 35.3) | 1.9 (1.3 to 2.5) | 4.4 (3.8 to 5) | 2 (1.9 to 2.2) | 53.8 (49.8 to 57.6) | 51.9 (48.3 to 55.7) | 1.8 (1.3 to 2.5) | 6.5 (4.7 to 8.5) | 0.6 (0.5 to 0.7) | 33.3 (29.4 to 37.9) | 29.8 (26.8 to 33.1) | 3.5 (2 to 5.5) |
| Comoros | 12 (7.3 to 18.1) | 10 (6.3 to 15.2) | 185 (117.8 to 274) | 179.1 (113.9 to 266.6) | 5.9 (3.4 to 9.8) | 2 (1.2 to 3) | 1.4 (0.8 to 2.1) | 29 (17.5 to 43.2) | 28.1 (16.9 to 42) | 0.9 (0.5 to 1.4) | 1.2 (0.6 to 1.8) | 0.8 (0.4 to 1.3) | 24.4 (13.5 to 38.2) | 24 (13.3 to 37.6) | 0.4 (0.2 to 0.6) | 0.3 (0.1 to 0.4) | 0.1 (0.1 to 0.1) | 5 (2.8 to 7.5) | 4.9 (2.7 to 7.4) | 0.1 (0 to 0.1) |
| Congo | 60.1 (40.6 to 82.5) | 47.2 (32.4 to 66.6) | 919.7 (633.4 to 1306.3) | 891.7 (612.3 to 1270.9) | 28 (16.6 to 40.1) | 6.6 (4.1 to 9.5) | 4.6 (2.8 to 6.8) | 92.8 (56 to 136.7) | 90.4 (54.6 to 133.9) | 2.4 (1.3 to 3.9) | 1.4 (0.9 to 2) | 1 (0.7 to 1.4) | 29.1 (19.5 to 41.8) | 28.7 (19.3 to 41.2) | 0.4 (0.2 to 0.7) | 0.6 (0.3 to 1) | 0.3 (0.1 to 0.4) | 11.4 (6 to 18.5) | 11.2 (5.9 to 18.2) | 0.1 (0.1 to 0.3) |
| Costa Rica | 92 (73.6 to 116.4) | 27.6 (24.4 to 30.7) | 463.2 (411.7 to 513.1) | 408.3 (365.5 to 451.4) | 55 (36 to 77.4) | 7.1 (5.7 to 8.7) | 3.3 (2.7 to 3.9) | 61.8 (51.3 to 73.5) | 58.2 (48.2 to 69.5) | 3.6 (2.5 to 4.9) | 6.6 (5.2 to 8.1) | 2.9 (2.3 to 3.4) | 73.4 (59.2 to 87.5) | 70.7 (57 to 84.2) | 2.7 (1.8 to 3.8) | 10.6 (7.4 to 14.4) | 0.8 (0.7 to 1) | 44.2 (36.1 to 54.9) | 38.9 (32.1 to 48.1) | 5.3 (3 to 8.9) |
| C么te d'Ivoire | 54.4 (35.1 to 76.1) | 45 (29.6 to 62.5) | 825.9 (546.2 to 1154.5) | 800.8 (528.9 to 1116.4) | 25.1 (15.4 to 37) | 4.7 (3 to 7.2) | 3.2 (2 to 5) | 67.2 (41.4 to 102) | 65.3 (40.2 to 99.6) | 1.9 (1.1 to 3) | 1 (0.6 to 1.5) | 0.7 (0.4 to 1) | 22 (13.4 to 32.7) | 21.6 (13.2 to 32.2) | 0.4 (0.2 to 0.6) | 0.3 (0.1 to 0.5) | 0.1 (0.1 to 0.2) | 5.3 (3 to 8.1) | 5.2 (2.9 to 8) | 0.1 (0 to 0.1) |
| Croatia | 55.3 (45 to 70.3) | 23.1 (21.2 to 25.2) | 386.4 (350.3 to 427.7) | 353.7 (321.2 to 388.8) | 32.7 (21.9 to 44.9) | 19.2 (16.1 to 22.4) | 9.4 (8.3 to 10.7) | 175.9 (154.4 to 199.5) | 166.8 (147.2 to 189.8) | 9.1 (6.2 to 12.3) | 13.4 (11.4 to 15.6) | 6.4 (5.7 to 7) | 148.5 (130.7 to 166.9) | 143.4 (126.1 to 161.1) | 5.1 (3.5 to 6.9) | 8.4 (5.4 to 11.5) | 0.5 (0.4 to 0.6) | 25.8 (20.2 to 32.3) | 21.4 (17.2 to 26) | 4.5 (2.5 to 7.5) |
| Cuba | 112.8 (85.9 to 143.8) | 37.4 (31.7 to 44) | 668.3 (572.9 to 762.7) | 604.9 (520.4 to 703.2) | 63.4 (42.6 to 89.9) | 13.2 (10.7 to 16.1) | 5.9 (5.1 to 6.9) | 117.2 (100.8 to 136.3) | 110.9 (96 to 130) | 6.3 (4.3 to 8.7) | 7.1 (5.7 to 8.8) | 3.2 (2.6 to 3.8) | 81.8 (68.3 to 97.4) | 79 (66.1 to 94.6) | 2.8 (1.9 to 3.9) | 2.5 (1.8 to 3.5) | 0.3 (0.2 to 0.4) | 12.2 (9.6 to 15.1) | 10.9 (8.7 to 13.6) | 1.3 (0.7 to 2.2) |
| Cyprus | 77 (51.2 to 108.7) | 27.7 (20.7 to 36.3) | 420.5 (303 to 551.9) | 370.6 (270.5 to 492.2) | 49.8 (28.9 to 73.4) | 21.9 (14.7 to 32) | 9.8 (6.5 to 14.1) | 160.6 (109.3 to 233.1) | 150 (102.6 to 218.8) | 10.6 (6.2 to 15.7) | 7.7 (5.2 to 11.6) | 3.4 (2.2 to 4.9) | 74.2 (48.4 to 107.7) | 71.1 (46.4 to 103.7) | 3.1 (1.8 to 5.3) | 7.5 (3.9 to 12.4) | 0.3 (0.2 to 0.4) | 14.3 (8.2 to 21.6) | 10.5 (6.1 to 15.9) | 3.8 (1.8 to 7.1) |
| Czechia | 57.7 (50.4 to 66.8) | 18.8 (17.5 to 20.4) | 332.6 (306.7 to 360) | 296.2 (276.2 to 318.3) | 36.4 (26 to 51.1) | 23.3 (19.9 to 27) | 7.8 (7 to 8.5) | 147 (134.7 to 161.3) | 134.2 (123.3 to 146.1) | 12.8 (9.1 to 17.5) | 19 (16.5 to 21.8) | 8.1 (7.4 to 8.8) | 183.7 (170.3 to 198.9) | 176.2 (163.8 to 190.6) | 7.5 (5.2 to 10.1) | 12.1 (8 to 16.5) | 0.7 (0.6 to 0.9) | 37.7 (30.3 to 46) | 31.1 (26 to 37) | 6.7 (3.7 to 10.7) |
| Denmark | 82.3 (67.8 to 97.7) | 32.4 (28.4 to 35.8) | 513.5 (452.2 to 573.6) | 460.9 (406.8 to 512.1) | 52.6 (36.3 to 71.7) | 17.2 (14.1 to 20.2) | 7.5 (6.5 to 8.6) | 125.8 (109.5 to 144.2) | 117.2 (101.2 to 134.3) | 8.5 (6 to 11.7) | 9.8 (8 to 11.9) | 4.7 (4 to 5.3) | 103.2 (88.4 to 118.4) | 99.3 (85.1 to 114.1) | 3.9 (2.5 to 5.4) | 8.9 (6.5 to 11.4) | 0.3 (0.2 to 0.3) | 14.8 (11.6 to 18.5) | 10.1 (8.2 to 12.2) | 4.7 (2.8 to 7.4) |
| Djibouti | 22.1 (14.1 to 31.7) | 18 (11.6 to 26.7) | 338.4 (221.3 to 495.2) | 327.3 (213.7 to 482.6) | 11.2 (6.6 to 17.2) | 4.2 (2.4 to 6.8) | 2.7 (1.5 to 4.2) | 62 (35.2 to 97.6) | 60.1 (33.9 to 94.7) | 1.9 (1 to 3.2) | 1.7 (1 to 2.7) | 1.2 (0.7 to 1.9) | 34.5 (19.7 to 55.5) | 33.9 (19.3 to 54.6) | 0.6 (0.3 to 1) | 0.9 (0.5 to 1.6) | 0.3 (0.2 to 0.5) | 17.4 (9.4 to 28.4) | 17.1 (9.2 to 28) | 0.3 (0.1 to 0.5) |
| Dominica | 140 (96.3 to 187.7) | 87 (62 to 117.1) | 1423.3 (1027.7 to 1909.3) | 1348.2 (971.6 to 1808) | 75.1 (45.6 to 112.6) | 5.9 (3.9 to 8.1) | 3.6 (2.4 to 4.8) | 67.7 (45.1 to 92) | 65 (43.4 to 88.1) | 2.6 (1.6 to 4.1) | 4.7 (3.2 to 7) | 2.6 (1.7 to 3.8) | 76.5 (50.5 to 110) | 74.7 (49.3 to 106.8) | 1.9 (1.1 to 3.2) | 0.4 (0.2 to 0.7) | 0.1 (0.1 to 0.2) | 4.7 (2.9 to 7.9) | 4.5 (2.8 to 7.4) | 0.2 (0.1 to 0.4) |
| Dominican Republic | 99 (64.3 to 134.9) | 63.6 (41.3 to 88.1) | 1017.9 (665.5 to 1392.5) | 973.2 (636.1 to 1338.5) | 44.7 (26.6 to 69.9) | 4 (2.7 to 5.7) | 2.4 (1.7 to 3.5) | 46.6 (31.7 to 65.7) | 45 (30.6 to 63.4) | 1.7 (1 to 2.6) | 2.1 (1.3 to 3.3) | 1.2 (0.7 to 1.9) | 34 (21.2 to 54.2) | 33.2 (20.8 to 52.8) | 0.8 (0.4 to 1.3) | 0.8 (0.5 to 1.4) | 0.2 (0.1 to 0.3) | 9.2 (6 to 14.7) | 8.8 (5.8 to 14.2) | 0.3 (0.1 to 0.7) |
| Ecuador | 60.4 (48.5 to 76.2) | 21.5 (19.3 to 24) | 360.5 (325.2 to 399.9) | 326 (295.3 to 361.6) | 34.5 (23.7 to 49) | 4.1 (3.4 to 4.9) | 1.6 (1.4 to 1.9) | 30.9 (27 to 35.7) | 28.7 (25.1 to 33) | 2.2 (1.5 to 3.2) | 5.3 (4.3 to 6.4) | 2 (1.6 to 2.3) | 54.4 (45.4 to 64.1) | 52.1 (43.6 to 61.2) | 2.3 (1.6 to 3.2) | 5.4 (3.8 to 7.2) | 0.7 (0.6 to 0.9) | 37.6 (31 to 45.6) | 34.9 (29 to 42) | 2.7 (1.5 to 4.3) |
| Egypt | 17.2 (10.6 to 24.5) | 10.8 (6.3 to 16.2) | 177.9 (106.4 to 263) | 168.8 (100.6 to 248.7) | 9.1 (5.5 to 15) | 14.6 (9.2 to 20.8) | 7.8 (5 to 11.3) | 159.4 (104.7 to 228.4) | 153 (100.6 to 217.9) | 6.4 (3.8 to 9.8) | 1.5 (0.9 to 2.1) | 0.9 (0.5 to 1.2) | 21.7 (13.5 to 30.9) | 21.2 (13.2 to 30.1) | 0.5 (0.3 to 0.9) | 0.7 (0.3 to 1.1) | 0.1 (0.1 to 0.2) | 5.2 (3.1 to 7.9) | 4.9 (2.9 to 7.4) | 0.3 (0.1 to 0.5) |
| El Salvador | 76.6 (51.6 to 113.6) | 31.8 (23.6 to 44.3) | 536.7 (404 to 764.5) | 494.4 (373 to 704.8) | 42.3 (26.1 to 64.4) | 3.6 (2.5 to 5.5) | 1.9 (1.4 to 2.7) | 37.3 (27.6 to 54.9) | 35.6 (26.3 to 52) | 1.7 (1 to 2.8) | 3.7 (2.4 to 5.4) | 1.9 (1.2 to 2.7) | 52.2 (33.9 to 74.6) | 50.8 (32.9 to 72.5) | 1.4 (0.9 to 2.4) | 3 (1.8 to 4.9) | 0.5 (0.3 to 0.7) | 22.9 (14.9 to 35.1) | 21.3 (13.8 to 32.5) | 1.6 (0.7 to 2.8) |
| Equatorial Guinea | 96.3 (64.4 to 138.6) | 66.3 (45.8 to 95.9) | 1312.9 (906.6 to 1922.6) | 1268.9 (878 to 1857.9) | 44 (26.5 to 69.3) | 11.3 (6.7 to 17.5) | 7.1 (4.1 to 11.1) | 150.6 (87.8 to 234.4) | 146.7 (85.3 to 228.2) | 3.9 (2.2 to 6.6) | 2.1 (1.2 to 3.1) | 1.4 (0.8 to 2) | 40 (23.7 to 60.3) | 39.5 (23.3 to 59.5) | 0.6 (0.3 to 0.9) | 1.5 (0.7 to 2.6) | 0.5 (0.2 to 0.8) | 22.3 (11.1 to 37.3) | 21.9 (10.9 to 36.4) | 0.4 (0.2 to 0.8) |
| Estonia | 119.3 (93.6 to 150.5) | 30.2 (26.9 to 33.7) | 570 (512.8 to 636.5) | 492.3 (439.6 to 544.7) | 77.7 (52.9 to 108.8) | 16.4 (13.5 to 19.6) | 8.3 (7.1 to 9.7) | 151.5 (128.7 to 175) | 143.8 (122.2 to 167.1) | 7.7 (5.3 to 10.4) | 19.1 (15.5 to 23.7) | 8.1 (6.7 to 9.5) | 187.8 (155.8 to 221.6) | 180.2 (150.5 to 214) | 7.6 (5 to 10.5) | 3.1 (2.2 to 4.2) | 0.3 (0.2 to 0.4) | 11.8 (9.5 to 14.8) | 10.2 (8.4 to 12.4) | 1.6 (0.8 to 2.8) |
| Eswatini | 28 (18.6 to 40) | 23.5 (15.7 to 33.9) | 446 (291.4 to 653.8) | 430.3 (281.2 to 632.3) | 15.7 (8.9 to 23.7) | 1.8 (1.1 to 2.7) | 1.2 (0.7 to 1.7) | 27.3 (17.1 to 42.2) | 26.4 (16.5 to 40.5) | 1 (0.6 to 1.6) | 1.7 (0.9 to 2.8) | 1.3 (0.7 to 2.1) | 36.8 (19.3 to 58.4) | 36.2 (19 to 57.4) | 0.6 (0.3 to 1) | 1.4 (0.7 to 2.3) | 0.5 (0.3 to 0.9) | 30 (15.8 to 48.3) | 29.5 (15.6 to 47.7) | 0.4 (0.2 to 0.8) |
| Fiji | 36.7 (23.9 to 51.1) | 29.3 (19.2 to 40.2) | 487 (320.2 to 672.7) | 469.8 (305.6 to 650) | 17.3 (10.3 to 27.9) | 5.6 (3.5 to 8.2) | 3 (2 to 4.6) | 67.2 (43.3 to 99.4) | 64.6 (41.8 to 95.8) | 2.6 (1.6 to 4.2) | 0.8 (0.5 to 1.2) | 0.5 (0.3 to 0.7) | 12.7 (8.3 to 18.3) | 12.4 (8.1 to 17.8) | 0.3 (0.2 to 0.5) | 2.1 (1.1 to 3.3) | 1.1 (0.6 to 1.7) | 33.3 (17.9 to 49.7) | 32.6 (17.6 to 48.8) | 0.6 (0.3 to 1.1) |
| Finland | 100.3 (85.7 to 115.3) | 21.4 (18.4 to 23.9) | 378.1 (325.1 to 427) | 308.9 (269.5 to 342.8) | 69.2 (50.1 to 92.5) | 11.2 (9.2 to 13.3) | 4.6 (4 to 5.2) | 79.6 (68.9 to 89.8) | 74 (64 to 84) | 5.6 (3.9 to 8) | 12 (9.6 to 14.3) | 4.8 (4 to 5.5) | 105.7 (90.7 to 122.7) | 100.8 (86.2 to 116.7) | 4.9 (3.2 to 6.7) | 4.1 (2.9 to 5.7) | 0.2 (0.1 to 0.2) | 8.9 (7.1 to 11.4) | 6.7 (5.5 to 8.2) | 2.2 (1.3 to 3.6) |
| France | 96 (72.2 to 119.5) | 18.5 (15.9 to 21) | 332.8 (281.4 to 381.1) | 268.7 (231 to 303.8) | 64.2 (42.6 to 87.1) | 24 (20.3 to 28.7) | 9.1 (8.1 to 10.3) | 167.3 (148.5 to 191.4) | 155.1 (138.2 to 177) | 12.2 (8.8 to 16.8) | 22.2 (18.4 to 26.9) | 5.7 (4.9 to 6.4) | 131 (115.1 to 148.7) | 121 (107.2 to 137.6) | 9.9 (6.9 to 13.8) | 11.8 (8.4 to 15.2) | 0.3 (0.3 to 0.3) | 19.4 (15.6 to 24.3) | 13.1 (11.2 to 15.6) | 6.3 (3.5 to 10.2) |
| Gabon | 56.1 (37.4 to 78.5) | 40.4 (26.9 to 56.4) | 787.9 (534.8 to 1098.3) | 759 (513.8 to 1062.5) | 29 (16.8 to 43.9) | 6.5 (3.8 to 10.2) | 4.3 (2.4 to 6.6) | 88 (50.2 to 135.1) | 85.3 (47.9 to 130.7) | 2.7 (1.4 to 4.5) | 1.9 (1.2 to 2.8) | 1.2 (0.8 to 1.9) | 37.3 (23.1 to 55.4) | 36.7 (22.8 to 54.5) | 0.6 (0.4 to 1) | 0.7 (0.4 to 1.2) | 0.3 (0.1 to 0.4) | 11.6 (5.9 to 18.5) | 11.4 (5.8 to 18) | 0.2 (0.1 to 0.5) |
| Georgia | 45.9 (37.4 to 56.6) | 25.5 (21.6 to 29.7) | 484.8 (408.2 to 560.8) | 458.4 (386.4 to 533.5) | 26.4 (17.6 to 36.8) | 18.5 (15.1 to 22.7) | 10.6 (8.7 to 12.6) | 221.4 (183.3 to 260.6) | 213.2 (176.2 to 252.8) | 8.2 (5.6 to 11.2) | 9.3 (7.6 to 10.9) | 5.4 (4.5 to 6.3) | 142.3 (119.2 to 165.9) | 139.1 (116 to 162.3) | 3.3 (2.1 to 4.7) | 5.1 (3.6 to 7) | 1.2 (1 to 1.4) | 42.9 (34.8 to 52.5) | 40.6 (33 to 49.2) | 2.3 (1.2 to 3.6) |
| Germany | 87.9 (77.6 to 97.5) | 20.1 (17.8 to 21.8) | 368.9 (329.4 to 405.5) | 307.9 (278.5 to 333.6) | 61.1 (45.5 to 81.3) | 17.6 (15.6 to 20) | 6.5 (5.9 to 7.2) | 118.4 (106.9 to 131.8) | 109.3 (98.9 to 121) | 9.1 (6.6 to 12.5) | 14.2 (12.3 to 16.6) | 5.6 (4.8 to 6.4) | 121.2 (106.3 to 139.7) | 115.4 (100.8 to 132.2) | 5.7 (3.8 to 8.2) | 13.3 (10.3 to 16.7) | 0.4 (0.4 to 0.5) | 24.4 (20.6 to 29.9) | 18.4 (16.2 to 21.2) | 6 (3.6 to 9.7) |
| Ghana | 48.9 (31.8 to 70.1) | 39 (25.1 to 55.5) | 716 (470 to 995.7) | 692.8 (450.9 to 964.3) | 23.3 (13.2 to 34.5) | 8.1 (5 to 12.7) | 4.9 (3 to 7.7) | 112.6 (67 to 175.4) | 109.1 (64.9 to 171.4) | 3.4 (1.9 to 5.6) | 1.8 (0.9 to 2.9) | 1.2 (0.6 to 1.9) | 35.7 (19.4 to 57.8) | 35.1 (19 to 56.8) | 0.6 (0.3 to 1) | 0.6 (0.3 to 0.9) | 0.2 (0.1 to 0.3) | 9.6 (4.9 to 15.4) | 9.4 (4.9 to 15.1) | 0.2 (0.1 to 0.3) |
| Greece | 51.1 (41 to 63.9) | 18 (15.7 to 20.3) | 288.5 (252.3 to 324.8) | 254.2 (220.9 to 284.1) | 34.3 (22.7 to 48.5) | 26.8 (23.1 to 31.5) | 10.8 (9.8 to 12.1) | 203.4 (182.7 to 228) | 189.7 (170.9 to 212.9) | 13.8 (9.7 to 18.8) | 10.1 (8.4 to 11.9) | 4.5 (4 to 5) | 104.2 (92.2 to 115.8) | 100.1 (88.7 to 111.1) | 4.1 (2.8 to 5.7) | 9 (6 to 12) | 0.3 (0.3 to 0.4) | 19.4 (14.9 to 24.2) | 14.6 (12 to 17.7) | 4.8 (2.6 to 8.2) |
| Greenland | 29.2 (17 to 43) | 19.5 (11.6 to 28) | 295.8 (178.5 to 424.2) | 279.8 (167.6 to 403.5) | 16 (8.9 to 25.5) | 14.7 (10.2 to 20.9) | 9 (6.3 to 12.4) | 149.9 (103.1 to 207.2) | 144.1 (99.3 to 198.5) | 5.8 (3.5 to 8.7) | 14.8 (8.8 to 21.3) | 8.4 (4.7 to 12.3) | 177.2 (106.2 to 248) | 172 (102.8 to 242.3) | 5.2 (2.9 to 7.8) | 2.5 (1.4 to 4.2) | 0.3 (0.2 to 0.5) | 14.9 (9 to 22.2) | 13.7 (8.3 to 20) | 1.2 (0.5 to 2.2) |
| Grenada | 111.7 (88.1 to 144.4) | 56.8 (47.9 to 68) | 1035.7 (870.5 to 1255.4) | 975.2 (816.6 to 1179.9) | 60.5 (40.8 to 84.6) | 4.6 (3.6 to 5.7) | 2.5 (1.9 to 3) | 51.5 (40.6 to 62.4) | 49.4 (38.8 to 59.9) | 2.2 (1.5 to 3.1) | 3.8 (3 to 4.8) | 2 (1.6 to 2.5) | 58.9 (47.6 to 72.2) | 57.5 (46.4 to 70.3) | 1.4 (1 to 2) | 1.5 (1 to 2.1) | 0.3 (0.2 to 0.4) | 14.2 (11.1 to 17.5) | 13.5 (10.6 to 16.5) | 0.7 (0.4 to 1.1) |
| Guam | 16.2 (12.5 to 21.4) | 8.2 (6.9 to 9.7) | 144.1 (121.4 to 168.8) | 133.4 (112.9 to 157.4) | 10.7 (7.2 to 15.1) | 8.3 (6.4 to 10.4) | 3 (2.5 to 3.6) | 75.2 (60.9 to 92.2) | 70.2 (57 to 85.2) | 5.1 (3.3 to 7.3) | 7.7 (6.2 to 9.5) | 3 (2.5 to 3.5) | 88 (72 to 103.4) | 84.7 (69.4 to 99) | 3.4 (2.2 to 4.9) | 0.4 (0.3 to 0.6) | 0.1 (0.1 to 0.1) | 3.1 (2.5 to 3.8) | 2.9 (2.3 to 3.5) | 0.2 (0.1 to 0.4) |
| Guatemala | 45.3 (35.9 to 55.9) | 26.3 (22.4 to 31.1) | 407 (348.2 to 473.1) | 384.1 (327.4 to 444.5) | 22.9 (14.6 to 31.8) | 1.6 (1.3 to 2) | 1 (0.8 to 1.2) | 18.6 (15.3 to 22.7) | 17.8 (14.7 to 21.8) | 0.8 (0.5 to 1.1) | 3.4 (2.8 to 4.1) | 2 (1.6 to 2.4) | 54.7 (44 to 65.8) | 53.4 (42.9 to 64.4) | 1.3 (0.9 to 1.9) | 3.6 (2.6 to 4.8) | 0.7 (0.6 to 0.9) | 38 (30 to 46.7) | 36.3 (28.6 to 44.8) | 1.6 (0.8 to 2.7) |
| Guinea | 32.5 (21.2 to 47.7) | 29.3 (18.7 to 42.9) | 521.8 (342.4 to 761) | 507.7 (330.9 to 743.1) | 14.1 (8.5 to 22.5) | 4.1 (2.6 to 6.2) | 3 (1.9 to 4.5) | 61.7 (38.6 to 90.8) | 60 (37.5 to 88.5) | 1.7 (0.9 to 2.7) | 1.1 (0.7 to 1.6) | 0.7 (0.4 to 1.1) | 24.6 (14.9 to 36) | 24.2 (14.7 to 35.4) | 0.4 (0.2 to 0.6) | 0.5 (0.3 to 0.8) | 0.2 (0.1 to 0.3) | 10.8 (6.1 to 16.3) | 10.6 (6 to 16.2) | 0.1 (0.1 to 0.2) |
| Guyana | 91.7 (71.8 to 120.2) | 59.3 (48.8 to 73.1) | 1066.2 (883.7 to 1306.8) | 1018.4 (845.5 to 1256.4) | 47.7 (32.2 to 67.1) | 4.4 (3.5 to 5.6) | 2.6 (2 to 3.3) | 56 (45.3 to 70.5) | 54 (43.5 to 68.4) | 2 (1.3 to 2.9) | 3.4 (2.6 to 4.2) | 1.9 (1.5 to 2.4) | 62 (49 to 75.5) | 60.8 (47.9 to 74) | 1.2 (0.8 to 1.8) | 1.3 (0.9 to 1.8) | 0.4 (0.3 to 0.5) | 18.4 (14.6 to 22.8) | 18 (14.2 to 22.2) | 0.5 (0.3 to 0.8) |
| Haiti | 66 (41.4 to 97.3) | 54.6 (34.1 to 84.1) | 948.8 (612 to 1430.6) | 919.5 (588.3 to 1392.1) | 29.4 (17.4 to 45.2) | 4.8 (2.8 to 7.4) | 3.4 (1.9 to 5.3) | 69.2 (40.3 to 107.7) | 67.3 (39.2 to 104.9) | 1.9 (1.1 to 3.3) | 2.7 (1.7 to 4.2) | 1.8 (1.1 to 2.7) | 58.3 (36.7 to 86.6) | 57.4 (35.9 to 85.5) | 0.9 (0.5 to 1.5) | 0.6 (0.3 to 1) | 0.3 (0.1 to 0.4) | 12.7 (6.7 to 21.9) | 12.6 (6.6 to 21.5) | 0.1 (0.1 to 0.3) |
| Honduras | 21.7 (14.5 to 30.8) | 16.4 (11 to 24) | 228.9 (155.6 to 322.8) | 219.6 (149.6 to 310) | 9.3 (5.6 to 14.2) | 1 (0.6 to 1.6) | 0.7 (0.4 to 1.2) | 12.2 (7.2 to 19.1) | 11.8 (7 to 18.5) | 0.4 (0.2 to 0.7) | 1.2 (0.7 to 1.9) | 0.8 (0.5 to 1.2) | 20 (12.3 to 31.2) | 19.6 (12 to 30.4) | 0.4 (0.2 to 0.7) | 0.8 (0.4 to 1.4) | 0.2 (0.1 to 0.4) | 10.8 (5.7 to 18) | 10.6 (5.5 to 17.5) | 0.3 (0.1 to 0.6) |
| Hungary | 46 (37.9 to 56.2) | 20 (18 to 21.7) | 357.7 (320.5 to 392.3) | 330.4 (297.5 to 360.2) | 27.4 (18.9 to 36.9) | 19.6 (17.4 to 22.4) | 9.6 (9 to 10.5) | 195.7 (183 to 211.7) | 186.4 (174.5 to 202.1) | 9.3 (6.7 to 12.5) | 12.6 (10.9 to 14.5) | 6.4 (5.7 to 7) | 156.1 (138.4 to 175.1) | 151.4 (134.5 to 169.5) | 4.7 (3.2 to 6.6) | 8.1 (5.7 to 10.8) | 0.7 (0.6 to 0.8) | 33.6 (28.7 to 39.9) | 29.3 (25.2 to 33.8) | 4.3 (2.3 to 7.3) |
| Iceland | 96.5 (74.6 to 125.6) | 27.5 (22.8 to 32.4) | 449.4 (381.2 to 529.3) | 385.2 (322.7 to 453.8) | 64.3 (43.6 to 90.3) | 14.8 (11.6 to 18.6) | 5.7 (4.6 to 6.8) | 98.8 (81 to 119.4) | 91 (74.8 to 110.1) | 7.8 (5.4 to 10.9) | 17.1 (13.2 to 21.4) | 6.5 (5.3 to 8) | 146.2 (118.9 to 178.7) | 139.1 (113.3 to 169.2) | 7.1 (4.8 to 9.8) | 4.9 (3.6 to 6.3) | 0.1 (0.1 to 0.1) | 7.8 (6.2 to 9.8) | 5.2 (4.2 to 6.4) | 2.6 (1.6 to 4) |
| India | 8.3 (5.6 to 11.9) | 6 (4.1 to 8.3) | 104.1 (71.7 to 146.3) | 99.8 (68.4 to 140) | 4.3 (2.6 to 6.5) | 3.5 (2.2 to 4.9) | 2.1 (1.4 to 3) | 43.2 (28.7 to 60.3) | 41.7 (27.6 to 58.2) | 1.5 (0.9 to 2.3) | 1.4 (0.9 to 2.1) | 1 (0.6 to 1.4) | 25.5 (16.2 to 36.9) | 25 (15.9 to 36.1) | 0.5 (0.3 to 0.8) | 0.9 (0.5 to 1.5) | 0.2 (0.1 to 0.3) | 11.2 (6.6 to 16.9) | 10.8 (6.4 to 16.4) | 0.3 (0.2 to 0.7) |
| Indonesia | 13.3 (8.8 to 19.5) | 9.1 (5.9 to 13.1) | 172.7 (115.4 to 251.2) | 165.5 (110.2 to 239.4) | 7.2 (4.2 to 11.6) | 3.2 (1.8 to 5.4) | 1.6 (0.8 to 2.8) | 38 (20.8 to 65.8) | 36.3 (19.6 to 63.6) | 1.7 (0.9 to 2.9) | 2.6 (1.5 to 4) | 1.4 (0.8 to 2.1) | 41 (25.3 to 61.1) | 40 (24.7 to 60) | 1 (0.6 to 1.7) | 0.9 (0.4 to 1.4) | 0.3 (0.2 to 0.5) | 13.3 (6.8 to 20.3) | 13 (6.7 to 19.9) | 0.3 (0.1 to 0.6) |
| Iran (Islamic Republic of) | 31.9 (22.6 to 43.7) | 13.5 (9.8 to 18.1) | 223.7 (163.8 to 294.6) | 204.6 (150.2 to 272.1) | 19.1 (12.2 to 29.4) | 8.5 (5.6 to 12.5) | 3.7 (2.5 to 5.5) | 74.4 (50.7 to 108.8) | 69.9 (46.9 to 102.6) | 4.5 (2.7 to 6.9) | 3.8 (2.5 to 5.7) | 1.7 (1.2 to 2.5) | 44.8 (29.7 to 64.7) | 43.2 (28.7 to 62.5) | 1.6 (0.9 to 2.7) | 3.1 (1.5 to 5) | 0.3 (0.1 to 0.4) | 11.1 (6.1 to 17.1) | 9.5 (5.3 to 14.5) | 1.6 (0.8 to 3) |
| Iraq | 26.3 (17.1 to 38) | 13.8 (9.3 to 20.1) | 232.8 (157 to 322.1) | 217.8 (146.8 to 302.6) | 15 (9.1 to 23.5) | 30.8 (21.7 to 41.4) | 15.2 (10.8 to 19.9) | 307.3 (220.7 to 396.9) | 293.2 (210 to 382.6) | 14.1 (9.2 to 22.2) | 5.9 (4.1 to 8.8) | 3 (2.1 to 4.6) | 82.6 (57.3 to 119.8) | 80.3 (55.7 to 116.5) | 2.3 (1.4 to 3.7) | 2.7 (1.3 to 4.4) | 0.3 (0.2 to 0.5) | 14.9 (8.2 to 22.8) | 13.6 (7.4 to 20.9) | 1.3 (0.6 to 2.4) |
| Ireland | 79.6 (61.6 to 101.4) | 21.6 (19.1 to 23.8) | 354.4 (306.6 to 393.1) | 301 (264.5 to 333.9) | 53.4 (34.7 to 74.5) | 14.3 (11.8 to 17) | 5.5 (4.8 to 6.3) | 95.8 (83.1 to 110.2) | 88.4 (76.6 to 102) | 7.4 (5.2 to 10) | 11.3 (8.9 to 13.9) | 4.4 (3.7 to 5.3) | 98.3 (82.1 to 117.7) | 93.7 (78.6 to 112.3) | 4.7 (3.1 to 6.3) | 8.5 (6 to 10.9) | 0.2 (0.2 to 0.3) | 14.1 (10.8 to 18.2) | 9.5 (7.9 to 11.4) | 4.6 (2.6 to 7.5) |
| Israel | 38.8 (30.2 to 48.8) | 12 (10.6 to 13.3) | 201.8 (176.5 to 224.6) | 176 (156.4 to 194.8) | 25.8 (17 to 35.6) | 16.7 (14.3 to 19.5) | 7 (6.3 to 7.8) | 124 (112 to 136.9) | 115.5 (105 to 128) | 8.5 (6 to 11.8) | 9.3 (7.5 to 11.3) | 4.1 (3.4 to 4.8) | 92.3 (77.6 to 109.6) | 88.5 (75 to 104.5) | 3.8 (2.5 to 5.3) | 4.2 (2.7 to 5.6) | 0.1 (0.1 to 0.2) | 8.5 (6.7 to 10.8) | 6.3 (5.2 to 7.6) | 2.2 (1.3 to 3.9) |
| Italy | 54.7 (44 to 66.8) | 13.9 (11.8 to 15.5) | 234.4 (199.7 to 265.6) | 197.4 (170 to 220.5) | 37 (24.9 to 50.2) | 30.3 (25.6 to 35.4) | 8.9 (7.8 to 9.8) | 157.2 (139.5 to 175.2) | 140.1 (124.9 to 156.3) | 17 (11.8 to 23.1) | 13.9 (11 to 17) | 4.7 (4 to 5.4) | 106.8 (91.1 to 124.6) | 100.8 (86.3 to 117.8) | 6 (4 to 8.1) | 9.8 (6.7 to 13.2) | 0.3 (0.3 to 0.4) | 19.3 (14.9 to 24.4) | 14 (11.5 to 17.4) | 5.3 (3 to 8.4) |
| Jamaica | 130.7 (102.3 to 165.5) | 60.9 (52.5 to 72.3) | 1081.5 (915.4 to 1300.7) | 1009.4 (849 to 1215.1) | 72 (50 to 101.5) | 7.1 (5.7 to 8.8) | 3.6 (2.9 to 4.3) | 74.1 (61 to 90.3) | 70.8 (58 to 86.3) | 3.3 (2.2 to 4.7) | 3.9 (3 to 4.9) | 1.9 (1.5 to 2.4) | 55.4 (44.9 to 69.3) | 54 (43.8 to 67.1) | 1.5 (1 to 2.1) | 1.8 (1.3 to 2.6) | 0.3 (0.2 to 0.4) | 13.9 (10.8 to 17.5) | 13.3 (10.4 to 16.9) | 0.7 (0.3 to 1.1) |
| Japan | 33.3 (28.5 to 38.5) | 8.9 (7.9 to 9.8) | 147.6 (129.6 to 161.9) | 125.1 (111.8 to 136.3) | 22.5 (16.9 to 29.7) | 13.9 (12.3 to 15.3) | 4.3 (3.8 to 4.8) | 74.4 (67 to 81.1) | 66.9 (60.2 to 73.2) | 7.6 (5.6 to 10) | 8.5 (7.2 to 9.8) | 3.6 (3.1 to 4) | 73.3 (65.3 to 82.7) | 69.9 (61.9 to 78.7) | 3.5 (2.3 to 4.6) | 4.4 (3 to 5.8) | 0.1 (0.1 to 0.2) | 7.7 (6.1 to 9.9) | 5.4 (4.5 to 6.7) | 2.3 (1.3 to 3.7) |
| Jordan | 36.7 (24.9 to 50.4) | 15.3 (11.7 to 19.7) | 266.1 (206 to 340.1) | 245.8 (189.6 to 312.3) | 20.3 (12.4 to 29.8) | 15.4 (10.8 to 22.2) | 6.8 (4.7 to 9.6) | 140.8 (99.1 to 197.3) | 133.3 (92.6 to 186.4) | 7.5 (4.4 to 11.3) | 3.7 (2.4 to 5.7) | 1.8 (1.2 to 2.6) | 45.4 (29.9 to 65.5) | 44 (29 to 63.4) | 1.4 (0.8 to 2.4) | 2.6 (1.3 to 4.4) | 0.3 (0.1 to 0.4) | 11.2 (6.2 to 16.9) | 10 (5.6 to 15.1) | 1.2 (0.5 to 2.3) |
| Kazakhstan | 18.1 (15.1 to 21.9) | 10.1 (9.1 to 11.3) | 186.8 (167.4 to 205.8) | 176.1 (157.9 to 195.1) | 10.7 (7.3 to 14.6) | 7.4 (6 to 9) | 4.2 (3.6 to 5) | 86.7 (73 to 102.1) | 83.2 (70.4 to 97.7) | 3.5 (2.3 to 4.9) | 5.5 (4.6 to 6.5) | 3.2 (2.7 to 3.6) | 82.1 (70.1 to 93.2) | 80.1 (68 to 90.7) | 2 (1.4 to 2.9) | 2.1 (1.4 to 2.8) | 0.4 (0.4 to 0.5) | 15.9 (12.7 to 19.1) | 15 (12.2 to 17.9) | 0.9 (0.5 to 1.6) |
| Kenya | 21.9 (13.5 to 32.1) | 17 (10.5 to 25.5) | 312.5 (194.2 to 460.4) | 302.5 (187 to 445.6) | 9.9 (6 to 14.9) | 2.1 (1.4 to 3.2) | 1.4 (0.8 to 2) | 29.2 (18.6 to 43.1) | 28.3 (17.9 to 41.7) | 0.9 (0.5 to 1.4) | 1.2 (0.8 to 1.8) | 0.9 (0.5 to 1.3) | 23.9 (14.7 to 35.5) | 23.5 (14.4 to 35) | 0.4 (0.2 to 0.6) | 0.3 (0.1 to 0.4) | 0.1 (0 to 0.1) | 4 (2.4 to 6.1) | 3.9 (2.3 to 6) | 0.1 (0 to 0.2) |
| Kiribati | 8.7 (5.7 to 12.6) | 7.9 (5.2 to 11.1) | 133 (88.7 to 192.3) | 128.6 (85.7 to 186.7) | 4.4 (2.6 to 6.8) | 0.7 (0.4 to 1.1) | 0.4 (0.2 to 0.6) | 9.5 (5.8 to 14.7) | 9.1 (5.4 to 14.1) | 0.4 (0.2 to 0.6) | 2 (1.1 to 3.4) | 1.2 (0.6 to 2.2) | 35.9 (20.7 to 60.7) | 35.1 (20.4 to 59.4) | 0.7 (0.3 to 1.4) | 0.2 (0.1 to 0.3) | 0.1 (0.1 to 0.2) | 3.5 (2.1 to 5.3) | 3.5 (2.1 to 5.2) | 0 (0 to 0.1) |
| Kuwait | 27.4 (20.6 to 35.7) | 9.5 (7.8 to 11.8) | 151.7 (124.1 to 184.9) | 134.8 (111.2 to 163.2) | 16.9 (11.2 to 24.3) | 10.3 (8 to 13.1) | 4.2 (3.3 to 5.3) | 78 (63.1 to 97) | 72.3 (58 to 89.5) | 5.7 (3.8 to 8.1) | 3.8 (2.8 to 4.7) | 1.5 (1.1 to 1.8) | 36.4 (28 to 43.7) | 34.7 (27 to 41.9) | 1.7 (1.1 to 2.4) | 1.3 (0.9 to 1.9) | 0.1 (0.1 to 0.1) | 3.1 (2.4 to 3.8) | 2.4 (1.9 to 2.8) | 0.7 (0.3 to 1.2) |
| Kyrgyzstan | 11.4 (9.2 to 14.3) | 7 (6 to 8.2) | 129.6 (112.9 to 149.8) | 123.1 (106.4 to 142.6) | 6.5 (4.4 to 9) | 4.1 (3.3 to 5.2) | 2.4 (1.9 to 2.9) | 50.7 (41.6 to 61.8) | 48.7 (40 to 59.7) | 2 (1.3 to 2.8) | 3.6 (2.9 to 4.4) | 2.1 (1.7 to 2.5) | 56.7 (45.8 to 68.2) | 55.3 (44.7 to 66.6) | 1.3 (0.9 to 1.8) | 1.3 (1 to 1.7) | 0.3 (0.3 to 0.4) | 12.3 (9.9 to 15.1) | 11.7 (9.6 to 14.3) | 0.5 (0.3 to 0.9) |
| Lao People's Democratic Republic | 14 (8.7 to 22.1) | 10.9 (7 to 17.4) | 198.8 (126.4 to 305.6) | 191.7 (122.1 to 296.5) | 7.1 (4.1 to 11.3) | 3.6 (2.1 to 5.9) | 2 (1.2 to 3.3) | 45.9 (27 to 74) | 44.1 (25.9 to 70.7) | 1.7 (1 to 3) | 1.7 (1 to 2.6) | 1 (0.6 to 1.5) | 29.5 (16.7 to 44.9) | 28.9 (16.3 to 44) | 0.7 (0.3 to 1) | 0.6 (0.3 to 1) | 0.3 (0.2 to 0.4) | 11.2 (6.3 to 16.8) | 11 (6.2 to 16.6) | 0.2 (0.1 to 0.3) |
| Latvia | 102.1 (80.3 to 127.9) | 32.7 (29.4 to 36.2) | 628.4 (562.6 to 696.8) | 561.9 (506.1 to 619.2) | 66.5 (43.4 to 91.9) | 21.1 (17.8 to 24.7) | 11 (9.6 to 12.7) | 221.4 (190.8 to 255.7) | 211.6 (183.5 to 243.1) | 9.8 (6.7 to 13.6) | 18.4 (15.6 to 21.6) | 8.7 (7.6 to 9.8) | 214.2 (185.6 to 244.3) | 207.1 (179.1 to 235.9) | 7.1 (4.8 to 10.1) | 5.2 (3.4 to 7) | 0.7 (0.5 to 0.8) | 28.1 (22.7 to 33.9) | 25.6 (20.9 to 30.5) | 2.6 (1.4 to 4.1) |
| Lebanon | 30.2 (20.7 to 42.5) | 11.7 (8.3 to 15.9) | 206.6 (148.7 to 272.3) | 187.7 (133.9 to 246.5) | 18.9 (11.6 to 29.1) | 21.7 (14.8 to 29.8) | 8.6 (5.8 to 11.8) | 194.1 (130.9 to 268.6) | 182 (122.4 to 250.3) | 12.1 (7.3 to 18.4) | 5.9 (3.9 to 8.5) | 2.5 (1.7 to 3.6) | 70.5 (45.6 to 100) | 67.9 (43.8 to 96.4) | 2.6 (1.5 to 4.3) | 4.2 (2.2 to 7) | 0.3 (0.2 to 0.5) | 16.1 (9.5 to 23.9) | 13.9 (8.4 to 20.5) | 2.2 (1 to 4.2) |
| Lesotho | 32.3 (19.8 to 50.6) | 29.7 (17.9 to 46.8) | 533.7 (332.3 to 850.9) | 519.4 (323.5 to 828.9) | 14.3 (7.9 to 22) | 3.3 (2 to 5.1) | 2.4 (1.5 to 3.7) | 50.7 (31.6 to 78.3) | 49.5 (30.8 to 76.7) | 1.2 (0.7 to 1.9) | 1.7 (0.9 to 2.7) | 1.3 (0.7 to 2.2) | 37.6 (19.8 to 59.6) | 37.1 (19.6 to 58.9) | 0.5 (0.3 to 0.8) | 0.4 (0.2 to 0.7) | 0.3 (0.2 to 0.4) | 9.7 (5.6 to 15.1) | 9.6 (5.5 to 14.9) | 0.1 (0 to 0.2) |
| Libya | 19 (11 to 29.3) | 11.1 (6.4 to 16.9) | 183 (103.5 to 282.8) | 173.2 (98 to 269.4) | 9.8 (5.2 to 16.3) | 17.7 (10.6 to 27.8) | 9.1 (5.4 to 14.2) | 186.3 (110.2 to 288.6) | 178.2 (104.7 to 276.5) | 8 (4.5 to 12.9) | 4.4 (2.6 to 6.5) | 2.2 (1.3 to 3.4) | 64.3 (38.2 to 99.6) | 62.6 (37 to 96.9) | 1.7 (0.9 to 2.8) | 1.1 (0.5 to 1.9) | 0.1 (0.1 to 0.2) | 7.1 (3.9 to 11) | 6.6 (3.6 to 10.3) | 0.6 (0.2 to 1.1) |
| Lithuania | 113.2 (92.7 to 137.8) | 27.9 (25.3 to 30.4) | 545.7 (493.6 to 596.9) | 471.3 (429.2 to 511.9) | 74.5 (51.8 to 102.3) | 16.6 (14.7 to 19) | 9.2 (8.6 to 9.9) | 176.9 (165.4 to 188.8) | 169.5 (159.3 to 180.7) | 7.4 (5.3 to 9.8) | 19.2 (16.4 to 22.1) | 8.8 (8 to 9.6) | 215.4 (189.3 to 237.6) | 208 (182.6 to 229) | 7.3 (5.1 to 10.3) | 2.8 (2 to 3.9) | 0.4 (0.3 to 0.5) | 16.3 (13.5 to 19.6) | 14.9 (12.4 to 18.2) | 1.4 (0.8 to 2.2) |
| Luxembourg | 55.7 (42.5 to 71.2) | 16.4 (14.2 to 19.3) | 266 (229.5 to 309.5) | 228.3 (198.1 to 266.7) | 37.7 (24.7 to 53.7) | 15.2 (12.2 to 19.1) | 6 (5.1 to 7.3) | 106.9 (90 to 129.8) | 99 (83.3 to 119.7) | 7.9 (5.4 to 10.8) | 5.9 (4.5 to 7.3) | 2.5 (2 to 3.1) | 54.5 (43.5 to 67) | 52 (41.7 to 64) | 2.5 (1.6 to 3.4) | 6.1 (4.2 to 8.2) | 0.2 (0.2 to 0.2) | 10.9 (8.5 to 14) | 7.7 (6.4 to 9.6) | 3.2 (1.8 to 5.1) |
| Malaysia | 18 (12.9 to 23.7) | 10.5 (8 to 13.2) | 178.1 (137 to 221.8) | 168 (129.6 to 210) | 10.2 (6.4 to 14.5) | 5.2 (3.7 to 7.4) | 2.5 (1.8 to 3.6) | 51 (35.3 to 71.1) | 48.4 (33.4 to 67.5) | 2.6 (1.7 to 3.9) | 3.3 (2.1 to 4.8) | 1.5 (0.9 to 2.1) | 40.9 (25.5 to 57.1) | 39.5 (24.9 to 55.4) | 1.3 (0.7 to 2.1) | 1.5 (0.9 to 2.5) | 0.3 (0.2 to 0.5) | 13.3 (8.3 to 19.5) | 12.7 (8 to 18.5) | 0.6 (0.3 to 1.2) |
| Maldives | 27.7 (17.3 to 39.5) | 14.2 (8.5 to 20.3) | 227.6 (143.6 to 318.6) | 212.3 (133.8 to 296.7) | 15.3 (8.8 to 24.1) | 6.9 (4.6 to 9.8) | 3.6 (2.4 to 5) | 60.5 (40.7 to 83.3) | 57.2 (38.5 to 79.3) | 3.2 (1.9 to 4.8) | 2.1 (1.5 to 3) | 0.9 (0.6 to 1.2) | 22.1 (14.6 to 30.6) | 21.2 (14.1 to 29.2) | 0.9 (0.5 to 1.6) | 2.3 (1.3 to 3.5) | 0.5 (0.4 to 0.7) | 15 (9.8 to 21.6) | 13.9 (9.1 to 19.9) | 1 (0.5 to 1.8) |
| Malta | 41.1 (30.3 to 53.1) | 10.9 (9 to 12.8) | 180.9 (151.2 to 213.8) | 152.3 (127.3 to 178.8) | 28.7 (17.9 to 41.2) | 15.9 (12.9 to 19.5) | 6.8 (5.7 to 8.1) | 118.2 (99.6 to 140.3) | 110.1 (92.1 to 130.8) | 8.1 (5.6 to 11.2) | 10.2 (8.2 to 12.8) | 4.4 (3.6 to 5.4) | 99.1 (83.3 to 123.2) | 94.9 (79.3 to 117.8) | 4.2 (2.8 to 5.9) | 7 (4.6 to 9.3) | 0.3 (0.2 to 0.3) | 14.1 (10.9 to 17.9) | 10.3 (8.4 to 12.3) | 3.9 (2.1 to 6.4) |
| Marshall Islands | 50 (32.9 to 67.6) | 43.5 (29.3 to 59.4) | 706.3 (485.3 to 962.3) | 684.5 (466.3 to 933.7) | 21.8 (12.8 to 33.4) | 6.8 (4.1 to 10.3) | 3.9 (2.3 to 6.2) | 85.8 (50.2 to 135.7) | 82.8 (48.3 to 131.9) | 3 (1.8 to 4.8) | 1.9 (1.1 to 3.1) | 1.2 (0.7 to 2) | 31.4 (17.4 to 51.6) | 30.8 (17 to 50.5) | 0.6 (0.3 to 1.1) | 0.4 (0.2 to 0.6) | 0.2 (0.1 to 0.4) | 6.4 (3.7 to 9.4) | 6.3 (3.6 to 9.3) | 0.1 (0 to 0.2) |
| Mauritania | 48.8 (29.1 to 74.4) | 36.2 (20.8 to 56.1) | 659.2 (384.2 to 1008) | 637.9 (370.2 to 976.6) | 21.3 (11.4 to 35.4) | 6.4 (3.6 to 10.2) | 4.1 (2.3 to 6.3) | 84.6 (49.1 to 129.9) | 82.2 (47.3 to 126.9) | 2.4 (1.3 to 3.9) | 2.1 (1.1 to 3.2) | 1.4 (0.7 to 2.2) | 38.2 (20.6 to 60) | 37.5 (20.3 to 58.8) | 0.6 (0.3 to 1.1) | 0.5 (0.2 to 0.9) | 0.2 (0.1 to 0.2) | 7.7 (3.8 to 12) | 7.5 (3.7 to 11.8) | 0.2 (0.1 to 0.3) |
| Mauritius | 36.3 (28.9 to 44.6) | 19 (16.5 to 22) | 337.2 (289.9 to 391.3) | 316.1 (274.3 to 366.6) | 21.1 (14 to 28.8) | 7.8 (6.2 to 9.8) | 3.5 (3 to 4.3) | 74.2 (61.8 to 89.6) | 70.2 (58.6 to 85.1) | 4.1 (2.8 to 5.9) | 5.4 (4.1 to 6.7) | 2.3 (1.9 to 2.9) | 63.5 (50.8 to 78.6) | 61.2 (49 to 75.7) | 2.2 (1.5 to 3.2) | 2.9 (2.1 to 4) | 0.6 (0.5 to 0.7) | 23.3 (19.2 to 28.3) | 21.9 (18 to 26.9) | 1.4 (0.8 to 2.1) |
| Mexico | 37.6 (30.5 to 45.8) | 15.6 (14.4 to 16.7) | 271 (252.9 to 289.6) | 250 (233.1 to 267.8) | 21 (14.1 to 28.8) | 3.4 (2.8 to 4.2) | 1.8 (1.5 to 2.1) | 35.4 (29.9 to 41.6) | 33.7 (28.4 to 39.9) | 1.6 (1.1 to 2.3) | 7.8 (6.5 to 9.1) | 4 (3.5 to 4.7) | 111.3 (95.6 to 127.9) | 108.4 (93.2 to 124.7) | 2.9 (2 to 3.9) | 9.3 (6.9 to 12.1) | 1.3 (1.1 to 1.5) | 72.5 (62.8 to 84.9) | 68.1 (59.2 to 80.3) | 4.5 (2.6 to 7.1) |
| Micronesia (Federated States of) | 27.8 (18.5 to 38.1) | 24.4 (16.5 to 33.3) | 387.3 (264.1 to 524.5) | 374.8 (256 to 510.8) | 12.5 (7.8 to 18.3) | 4.9 (3.1 to 7.2) | 2.8 (1.8 to 4.3) | 60.6 (38.2 to 90.5) | 58.2 (36.9 to 87) | 2.3 (1.4 to 3.5) | 0.5 (0.3 to 0.8) | 0.3 (0.2 to 0.5) | 8.8 (5.3 to 13.1) | 8.6 (5.1 to 12.8) | 0.2 (0.1 to 0.3) | 0.3 (0.2 to 0.5) | 0.2 (0.1 to 0.3) | 5.4 (3.3 to 7.8) | 5.3 (3.2 to 7.7) | 0.1 (0 to 0.2) |
| Monaco | 107.1 (65.5 to 169.7) | 34.7 (21.3 to 51.9) | 541.3 (344.3 to 773.6) | 477.8 (301.2 to 700.9) | 63.5 (34.9 to 101.7) | 24.9 (14.2 to 40.5) | 10.9 (6.2 to 17.9) | 179.1 (103.5 to 289.5) | 168.2 (97 to 270.9) | 10.9 (6 to 18.9) | 17.4 (10.9 to 27.6) | 7.3 (4.5 to 10.9) | 164.8 (100.6 to 244.7) | 158.3 (96.8 to 235.5) | 6.4 (3.7 to 11) | 36.7 (17.7 to 63.5) | 1.1 (0.6 to 1.6) | 66.8 (35.9 to 101.4) | 48.3 (25.7 to 72.7) | 18.5 (8.9 to 34.6) |
| Mongolia | 7.4 (5 to 10.1) | 4.8 (3.3 to 6.8) | 94.1 (66.3 to 131.9) | 90 (63.3 to 126.5) | 4.2 (2.6 to 6.1) | 3.4 (2.2 to 4.8) | 2 (1.3 to 2.9) | 46.3 (30.1 to 64.7) | 44.8 (29.1 to 63) | 1.6 (0.9 to 2.4) | 4.5 (3 to 6.4) | 2.8 (1.8 to 3.9) | 79.3 (52 to 112.2) | 77.8 (51 to 110.3) | 1.5 (0.8 to 2.4) | 1.7 (1 to 2.8) | 0.5 (0.3 to 0.8) | 19.8 (12.7 to 30.4) | 19.3 (12.3 to 29.4) | 0.6 (0.2 to 1) |
| Montenegro | 55.7 (39.1 to 75.7) | 23.9 (16.6 to 30.4) | 412.9 (293.6 to 526.5) | 379.4 (266.5 to 484.5) | 33.5 (20.9 to 50.9) | 16 (11 to 22.3) | 7.8 (5.4 to 10.8) | 153.2 (106 to 210.8) | 145.6 (100.5 to 199.9) | 7.6 (4.7 to 11.5) | 8.6 (5.8 to 12) | 4.1 (2.8 to 5.6) | 100.3 (69.7 to 136.4) | 97 (67.2 to 131.1) | 3.3 (2 to 5.5) | 10.7 (5.9 to 16.8) | 0.7 (0.5 to 1) | 37.4 (23.4 to 52.4) | 31.7 (19.9 to 45.3) | 5.7 (2.8 to 10.6) |
| Morocco | 9.8 (4.4 to 16.6) | 6.6 (3 to 11.2) | 106.8 (46.9 to 176.7) | 102.4 (44.9 to 170.1) | 4.4 (1.9 to 8) | 5.3 (3 to 8.7) | 3.1 (1.8 to 5.3) | 59.6 (34.6 to 97.8) | 57.5 (33.3 to 94.5) | 2 (1.1 to 3.4) | 0.4 (0.2 to 0.6) | 0.2 (0.1 to 0.4) | 5.6 (3.2 to 8.6) | 5.4 (3.2 to 8.4) | 0.1 (0.1 to 0.2) | 0.5 (0.2 to 0.8) | 0.1 (0 to 0.1) | 4.1 (2.3 to 6.4) | 3.9 (2.2 to 6.1) | 0.2 (0.1 to 0.4) |
| Myanmar | 19.5 (12 to 29.2) | 14.7 (9.1 to 22.5) | 269.9 (167.8 to 403.2) | 260 (162.3 to 391.1) | 9.8 (6.1 to 16) | 4 (2.3 to 6.3) | 2.2 (1.2 to 3.5) | 48.9 (28.2 to 78.1) | 47 (27 to 75.3) | 1.9 (1 to 3.1) | 1.7 (1.1 to 2.6) | 0.9 (0.6 to 1.4) | 28.9 (17.2 to 43) | 28.2 (16.7 to 42.2) | 0.7 (0.3 to 1.1) | 0.8 (0.4 to 1.3) | 0.3 (0.2 to 0.5) | 13.5 (7.7 to 21.8) | 13.3 (7.6 to 21.4) | 0.2 (0.1 to 0.4) |
| Namibia | 30.6 (20.4 to 40) | 24.2 (16.4 to 31.1) | 447.3 (291.3 to 576.6) | 431 (279.2 to 556.9) | 16.3 (10 to 23.2) | 2.3 (1.5 to 3.6) | 1.4 (0.9 to 2.2) | 33.1 (21.4 to 50.1) | 32 (20.7 to 48.5) | 1.1 (0.7 to 1.7) | 2.1 (1.3 to 3.2) | 1.4 (0.8 to 2) | 42.8 (25.7 to 64.5) | 42.1 (25.3 to 63.4) | 0.7 (0.4 to 1.1) | 1.4 (0.7 to 2.3) | 0.4 (0.2 to 0.7) | 23.3 (12.1 to 36.4) | 22.8 (11.9 to 35.7) | 0.5 (0.2 to 0.8) |
| Nauru | 46.7 (29.7 to 64.3) | 38.1 (24.9 to 52.5) | 671.2 (443.7 to 933.5) | 649.2 (428.4 to 895.8) | 22 (13.3 to 33.7) | 8.7 (5.1 to 13.8) | 4.4 (2.6 to 7.1) | 111 (65.3 to 176.7) | 107 (63.5 to 170.3) | 4 (2.2 to 6.8) | 2.5 (1.4 to 3.9) | 1.5 (0.8 to 2.4) | 42.8 (22.8 to 67.7) | 41.9 (22.4 to 66.1) | 0.9 (0.4 to 1.5) | 0.5 (0.3 to 0.8) | 0.3 (0.1 to 0.4) | 9.3 (5 to 14.2) | 9.1 (5 to 14) | 0.1 (0.1 to 0.2) |
| Nepal | 6 (3.6 to 9.7) | 4.8 (2.9 to 7.6) | 83.4 (50.8 to 134.5) | 80.5 (49.1 to 129.7) | 2.9 (1.5 to 5.1) | 2.2 (1.3 to 3.9) | 1.5 (0.8 to 2.5) | 30.2 (17 to 52.2) | 29.3 (16.3 to 50.2) | 1 (0.5 to 1.7) | 1 (0.6 to 1.9) | 0.8 (0.4 to 1.4) | 19.6 (10.9 to 35.2) | 19.2 (10.7 to 34.4) | 0.3 (0.1 to 0.8) | 0.7 (0.4 to 1.2) | 0.2 (0.1 to 0.3) | 11 (5.9 to 18.2) | 10.8 (5.8 to 17.6) | 0.2 (0.1 to 0.5) |
| Netherlands | 72.4 (59.1 to 87.4) | 23.7 (20.6 to 26.3) | 385.6 (339.8 to 430.4) | 338.6 (298.4 to 374.4) | 47 (31.9 to 67.2) | 15.8 (13.7 to 18.3) | 7.9 (7.1 to 8.9) | 130.1 (117.8 to 145.1) | 122.6 (111.3 to 137) | 7.5 (5.2 to 10.1) | 12.4 (10.5 to 14.5) | 5.7 (5 to 6.3) | 121.9 (108.5 to 135.5) | 117 (103.7 to 129.7) | 5 (3.6 to 6.7) | 8.6 (6.1 to 11.5) | 0.3 (0.2 to 0.3) | 16.1 (12.9 to 20.1) | 11.5 (9.6 to 14.2) | 4.6 (2.5 to 7.4) |
| New Zealand | 107.6 (81.1 to 132.7) | 22.7 (19.6 to 25.4) | 401.1 (342.2 to 450.8) | 328.3 (286.3 to 366.4) | 72.8 (49.1 to 99.4) | 13.6 (11.4 to 15.9) | 5.3 (4.6 to 6) | 94.1 (83.3 to 107) | 87.3 (77 to 99.2) | 6.8 (4.9 to 9.4) | 19.1 (14.8 to 23.5) | 4.2 (3.5 to 4.9) | 105 (88.3 to 124.3) | 96.2 (81.3 to 114.5) | 8.8 (6 to 12.3) | 9.2 (6.3 to 11.9) | 0.2 (0.2 to 0.3) | 16.5 (13.1 to 20.3) | 11.7 (9.8 to 14.2) | 4.8 (2.8 to 7.5) |
| Nicaragua | 48.7 (36.1 to 65.2) | 19.2 (13.9 to 24.1) | 323.5 (245.2 to 408) | 297.1 (223.1 to 376.2) | 26.4 (16.6 to 37.9) | 2.1 (1.4 to 3.1) | 1.1 (0.7 to 1.6) | 21.5 (13.8 to 30.9) | 20.4 (13.2 to 29.6) | 1 (0.7 to 1.6) | 4.2 (2.6 to 6.4) | 2.1 (1.2 to 3.1) | 57.7 (34.6 to 83.2) | 56 (33.7 to 81) | 1.6 (0.9 to 2.6) | 3.7 (1.9 to 6) | 0.5 (0.3 to 0.8) | 25.4 (15.1 to 39.5) | 23.6 (14.1 to 36.8) | 1.8 (0.8 to 3.6) |
| Nigeria | 49.9 (30.6 to 73.5) | 38.8 (24 to 57.6) | 715.5 (449.9 to 1050.7) | 693.6 (436.8 to 1029.3) | 21.9 (13.4 to 32.9) | 2.8 (1.6 to 4.4) | 1.9 (1 to 2.9) | 38 (21.6 to 58.4) | 36.9 (20.8 to 56.7) | 1.1 (0.6 to 1.8) | 1.6 (0.9 to 2.5) | 1 (0.5 to 1.6) | 31 (16.9 to 48.6) | 30.5 (16.6 to 48.1) | 0.5 (0.3 to 0.8) | 0.1 (0.1 to 0.2) | 0 (0 to 0.1) | 1.7 (1 to 2.7) | 1.7 (1 to 2.6) | 0 (0 to 0.1) |
| North Macedonia | 23.8 (15.2 to 30.5) | 16.6 (10.7 to 21.3) | 279.7 (178.7 to 358.5) | 267.3 (169.9 to 344) | 12.4 (7.4 to 17.9) | 15.7 (11.9 to 20.4) | 8.6 (6.6 to 10.7) | 166.2 (128.7 to 210.6) | 159.1 (122.8 to 201.8) | 7.1 (4.8 to 10.5) | 4.8 (3.1 to 6.8) | 2.6 (1.6 to 3.5) | 64 (39.7 to 84.8) | 62.2 (38.5 to 82.6) | 1.9 (1.1 to 3) | 9.3 (5.2 to 14.9) | 1 (0.6 to 1.3) | 46.3 (29.8 to 67.2) | 41.6 (26.5 to 59.9) | 4.8 (2.3 to 8.7) |
| Northern Mariana Islands | 42.4 (24.2 to 68.5) | 25.5 (14.9 to 42.1) | 416.3 (245.6 to 679.1) | 391.3 (230.8 to 639.8) | 25.1 (13 to 42.2) | 8.4 (5.4 to 12) | 3.8 (2.5 to 5.4) | 81.9 (53.1 to 115) | 77.2 (50.3 to 108.6) | 4.7 (2.8 to 7.3) | 5.5 (3.7 to 8) | 2.6 (1.7 to 3.8) | 65.4 (43.2 to 94.4) | 63.2 (41.8 to 91.2) | 2.2 (1.2 to 3.5) | 0.7 (0.4 to 1.1) | 0.2 (0.1 to 0.3) | 6.2 (3.6 to 8.9) | 5.9 (3.5 to 8.4) | 0.3 (0.1 to 0.6) |
| Norway | 84.1 (69.2 to 99.7) | 24.9 (21.4 to 28.4) | 396.8 (342.6 to 444.8) | 341.5 (295.2 to 388.4) | 55.4 (39.6 to 74.5) | 13.8 (11.1 to 17.5) | 5.6 (4.6 to 6.6) | 92.8 (78.3 to 109) | 85.7 (72.1 to 101.3) | 7.1 (5 to 9.8) | 9.9 (7.5 to 12.3) | 3.9 (3.2 to 4.7) | 83.6 (68 to 102) | 79.5 (64.8 to 96.6) | 4.1 (2.7 to 5.6) | 6.2 (4.9 to 7.9) | 0.2 (0.1 to 0.2) | 9.6 (7.7 to 12.2) | 6.4 (5.3 to 8.1) | 3.2 (2.1 to 4.9) |
| Oman | 21.6 (13.6 to 31.9) | 11.5 (7.3 to 16.5) | 179.5 (118.3 to 255.4) | 167.7 (109.7 to 238) | 11.8 (6.7 to 19) | 9 (5.6 to 14) | 4.6 (2.8 to 7) | 85.7 (52.5 to 129.7) | 81.4 (50.3 to 123.3) | 4.3 (2.4 to 6.8) | 1.7 (1 to 2.6) | 0.9 (0.5 to 1.4) | 21.6 (12.5 to 33.2) | 21 (12 to 32.1) | 0.7 (0.3 to 1.1) | 0.1 (0 to 0.2) | 0 (0 to 0) | 0.4 (0.2 to 0.7) | 0.4 (0.2 to 0.6) | 0 (0 to 0.1) |
| Pakistan | 8.4 (5.5 to 12.2) | 6.7 (4.5 to 9.6) | 121.1 (81.1 to 172.1) | 116.9 (78.2 to 166.2) | 4.2 (2.5 to 6.2) | 4.7 (2.9 to 7.2) | 3.1 (1.8 to 4.9) | 66 (39.6 to 102.6) | 63.9 (37.9 to 98.8) | 2.1 (1.2 to 3.5) | 2.1 (1.3 to 3.2) | 1.4 (0.9 to 2.2) | 41.4 (26.1 to 63.6) | 40.7 (25.7 to 62.3) | 0.7 (0.4 to 1.3) | 1.4 (0.7 to 2.4) | 0.5 (0.3 to 0.7) | 23.6 (13.1 to 36.4) | 23.1 (12.9 to 35.4) | 0.4 (0.2 to 0.8) |
| Palau | 42.7 (26.8 to 61.8) | 33.6 (21.2 to 47.3) | 528.5 (340.4 to 740.5) | 509.2 (330.2 to 708.5) | 19.3 (11.1 to 29.5) | 2.2 (1.4 to 3.2) | 1.1 (0.7 to 1.6) | 24.8 (15.3 to 36.4) | 23.6 (14.5 to 34.6) | 1.1 (0.7 to 1.8) | 1.1 (0.7 to 1.6) | 0.7 (0.4 to 1) | 15.3 (9.9 to 22.5) | 14.9 (9.6 to 21.9) | 0.4 (0.2 to 0.6) | 0.4 (0.2 to 0.6) | 0.2 (0.1 to 0.3) | 5.3 (2.9 to 8.3) | 5.2 (2.8 to 8.2) | 0.1 (0.1 to 0.2) |
| Palestine | 31.3 (22.7 to 43.2) | 16.1 (12.1 to 20.8) | 270 (201.8 to 346.8) | 251.6 (188.3 to 320.2) | 18.4 (11.3 to 27.4) | 12.7 (8.9 to 18.6) | 6.3 (4.4 to 9.1) | 126.8 (88.5 to 181.3) | 120.5 (83.7 to 172.4) | 6.3 (3.9 to 9.7) | 3.1 (2.1 to 4.7) | 1.7 (1.1 to 2.4) | 42.4 (27.7 to 60.8) | 41.1 (26.9 to 58.9) | 1.3 (0.7 to 2.1) | 0.8 (0.4 to 1.2) | 0.1 (0.1 to 0.2) | 4.4 (2.6 to 6.5) | 4 (2.4 to 6) | 0.4 (0.2 to 0.7) |
| Panama | 74.8 (61.4 to 90.1) | 23.2 (20.2 to 26) | 401.7 (356.6 to 450) | 358.6 (320.4 to 401.3) | 43.1 (30.4 to 59.6) | 2.8 (2.2 to 3.4) | 1.4 (1.1 to 1.6) | 25.8 (20.8 to 30.8) | 24.3 (19.7 to 29.3) | 1.5 (0.9 to 2) | 6.7 (5.3 to 8.2) | 3 (2.4 to 3.6) | 80.2 (64.5 to 95.4) | 77.5 (62.5 to 92.1) | 2.8 (1.7 to 4.1) | 4.9 (3.4 to 6.7) | 0.4 (0.4 to 0.5) | 23.8 (19.5 to 29) | 21.3 (17 to 26.1) | 2.5 (1.5 to 4) |
| Papua New Guinea | 28.2 (17.8 to 40.7) | 22.7 (14.4 to 33.6) | 381.8 (245.7 to 550.4) | 367.9 (237.9 to 533.5) | 13.9 (8.1 to 21.8) | 3.2 (1.6 to 5.1) | 1.7 (0.8 to 2.8) | 38.7 (18.3 to 64.8) | 37 (17.5 to 62.4) | 1.6 (0.8 to 2.9) | 1.2 (0.5 to 2.2) | 0.7 (0.3 to 1.3) | 18.9 (8.3 to 33.7) | 18.5 (8.1 to 32.8) | 0.4 (0.2 to 0.8) | 0.2 (0.1 to 0.4) | 0.1 (0 to 0.2) | 3.6 (1.7 to 7) | 3.6 (1.6 to 6.9) | 0.1 (0 to 0.1) |
| Paraguay | 51.2 (30.7 to 67.6) | 29.8 (17.4 to 37.6) | 485.3 (291.3 to 604.1) | 457.9 (273.9 to 566.6) | 27.3 (16 to 41.4) | 5.3 (3.5 to 6.9) | 3.3 (2.2 to 4.2) | 58.8 (39.7 to 75.5) | 56.5 (38.1 to 72.7) | 2.3 (1.3 to 3.1) | 5.3 (2.9 to 7.4) | 3.1 (1.7 to 4.3) | 81.7 (46.1 to 112.3) | 79.7 (44.9 to 109.9) | 2 (1.1 to 3.2) | 3.7 (2.3 to 5.6) | 0.8 (0.6 to 1.2) | 37.7 (25.2 to 54.1) | 36 (24.2 to 51.3) | 1.8 (0.9 to 3) |
| Peru | 75.3 (52.6 to 106) | 24.1 (17.8 to 31.6) | 382.3 (285.1 to 498) | 351.2 (261.8 to 459.6) | 31.1 (18.9 to 46.5) | 5.7 (4.6 to 7) | 2.1 (1.8 to 2.4) | 38.9 (31.7 to 47.1) | 36.7 (30 to 44.1) | 2.2 (1.4 to 3.1) | 10.5 (7.4 to 13.8) | 3.4 (2.2 to 4.4) | 95.4 (62.5 to 122.5) | 92.4 (60.3 to 119.2) | 3.1 (1.8 to 4.9) | 6.2 (3.9 to 8.8) | 0.8 (0.6 to 1) | 35.8 (26.5 to 45.7) | 33.9 (25.1 to 43.1) | 1.9 (1 to 3.1) |
| Philippines | 21.8 (15.1 to 28.2) | 16.4 (11 to 21.4) | 274.7 (189.9 to 354.2) | 263.7 (182.2 to 342) | 11 (6.7 to 16.2) | 2.6 (1.8 to 3.5) | 1.4 (0.9 to 1.9) | 29.9 (20.6 to 41.5) | 28.5 (19.7 to 39.5) | 1.4 (0.9 to 2) | 3.1 (2.2 to 4.5) | 1.6 (1.1 to 2.3) | 47.9 (32.1 to 66.8) | 46.6 (31.2 to 64.9) | 1.3 (0.8 to 2) | 0.9 (0.5 to 1.3) | 0.3 (0.2 to 0.4) | 12.2 (7.9 to 17.1) | 11.9 (7.8 to 16.7) | 0.3 (0.1 to 0.5) |
| Poland | 42.2 (37.7 to 47.4) | 24 (22.1 to 25.7) | 406.3 (375.8 to 434.2) | 383 (356 to 408) | 23.3 (17.2 to 31.2) | 21.2 (19.5 to 23.2) | 12.3 (11.4 to 13.2) | 231 (215.4 to 248.4) | 221.7 (206.7 to 239.9) | 9.3 (6.7 to 12) | 10 (8.7 to 11.3) | 6.4 (5.8 to 7.1) | 145 (130.9 to 160.8) | 141.7 (127.7 to 157.1) | 3.4 (2.4 to 4.5) | 6.6 (4.8 to 8.7) | 0.7 (0.6 to 0.8) | 34.3 (30.5 to 39) | 30.9 (27 to 35) | 3.5 (2.1 to 5.5) |
| Portugal | 89.4 (67.9 to 114.9) | 19.6 (17.1 to 21.9) | 351.1 (298.1 to 397.2) | 289.7 (253.2 to 322.6) | 61.4 (40.9 to 87.5) | 28.6 (24.1 to 33.7) | 6.9 (6.2 to 7.7) | 136.4 (120.5 to 153.1) | 119.7 (106.8 to 134.2) | 16.7 (11.9 to 22.8) | 11.6 (9.4 to 13.9) | 3.4 (2.9 to 3.8) | 81.6 (70.8 to 92) | 76.4 (66.4 to 87) | 5.2 (3.4 to 7.2) | 5.8 (3.8 to 7.9) | 0.2 (0.2 to 0.3) | 13.3 (10.6 to 16.7) | 10.3 (8.7 to 12.7) | 3.1 (1.7 to 5) |
| Puerto Rico | 55.1 (43.1 to 69) | 15.6 (13.6 to 17.3) | 279.8 (246.1 to 311) | 244.8 (217.6 to 271.6) | 34.9 (23 to 48.8) | 7.6 (6.3 to 9.2) | 3.1 (2.6 to 3.5) | 61.7 (53.2 to 70.1) | 57.5 (49.8 to 65.8) | 4.2 (2.9 to 6) | 7 (5.4 to 8.7) | 2.4 (1.9 to 2.9) | 71.2 (57.3 to 86.6) | 68 (54.7 to 82.2) | 3.2 (2.1 to 4.4) | 7 (4.9 to 9.7) | 0.4 (0.3 to 0.5) | 23.6 (19.1 to 29.6) | 19.7 (16 to 25) | 3.9 (2.2 to 6.5) |
| Qatar | 29.2 (16.7 to 47.9) | 12 (7.4 to 17.7) | 182.4 (112 to 279.3) | 164.4 (102 to 247.8) | 18 (9.1 to 30.2) | 5.7 (3.6 to 8.4) | 2.6 (1.6 to 3.9) | 45.3 (29 to 66.7) | 42.2 (26.9 to 63) | 3.1 (1.8 to 4.7) | 2.1 (1.4 to 3.1) | 0.9 (0.6 to 1.3) | 21.2 (14 to 29.7) | 20.3 (13.5 to 28.6) | 0.9 (0.5 to 1.5) | 0.3 (0.2 to 0.5) | 0 (0 to 0) | 0.9 (0.5 to 1.3) | 0.7 (0.4 to 1.1) | 0.2 (0.1 to 0.3) |
| Republic of Korea | 26.1 (17.9 to 35.5) | 8.3 (5.6 to 10.4) | 130.2 (93.3 to 162.5) | 112.8 (80.2 to 139.2) | 17.4 (10.9 to 25.5) | 13.6 (11.2 to 16.8) | 4.8 (4 to 5.7) | 76.8 (65.7 to 90.8) | 69.8 (59.8 to 82.6) | 6.9 (4.9 to 9.7) | 6.1 (4.2 to 8.3) | 2.5 (1.6 to 3.1) | 55.8 (36.3 to 72.4) | 53.2 (34.7 to 68.9) | 2.5 (1.5 to 4) | 1.4 (0.8 to 2.1) | 0 (0 to 0.1) | 2.6 (1.8 to 3.5) | 1.9 (1.3 to 2.5) | 0.7 (0.3 to 1.3) |
| Republic of Moldova | 43.5 (33.9 to 55) | 13.9 (12 to 15.9) | 305.6 (264.6 to 351.7) | 277.1 (238.7 to 321.2) | 28.5 (20 to 41.2) | 11.9 (9.7 to 14.8) | 6.1 (5.1 to 7.4) | 138.1 (115.8 to 165.4) | 132.4 (111 to 157.7) | 5.7 (3.8 to 7.8) | 8 (6.5 to 9.7) | 3.9 (3.2 to 4.7) | 107.4 (87.3 to 127.7) | 104.3 (85 to 124.2) | 3.1 (2 to 4.5) | 2.3 (1.6 to 3.2) | 0.4 (0.3 to 0.5) | 17 (13.5 to 20.5) | 15.9 (12.8 to 19.2) | 1.1 (0.6 to 1.9) |
| Romania | 34.5 (28 to 41.8) | 17.1 (15.1 to 19.3) | 305.5 (269.5 to 345.9) | 285 (251.1 to 322.3) | 20.5 (14.2 to 27.8) | 17.3 (15 to 20.3) | 9 (8.1 to 10.2) | 184.7 (163.4 to 210.1) | 176.5 (155.8 to 199.3) | 8.1 (5.7 to 10.9) | 8.7 (7.2 to 10.4) | 4.5 (3.9 to 5.3) | 117.9 (100.6 to 141.3) | 114.6 (97.8 to 137.6) | 3.3 (2.1 to 4.7) | 5.7 (4 to 7.9) | 0.6 (0.5 to 0.7) | 29.3 (24.1 to 36.2) | 26.4 (21.4 to 32.7) | 2.9 (1.6 to 4.7) |
| Russian Federation | 61 (51.2 to 75) | 19 (17 to 21.4) | 371.5 (328.8 to 420.4) | 331.6 (296.1 to 373.3) | 40 (28.3 to 54.2) | 13.4 (11.2 to 16) | 6 (5.1 to 6.9) | 125 (106.8 to 144.7) | 118.2 (101.3 to 136.1) | 6.8 (4.8 to 9.1) | 13.8 (11.8 to 16.5) | 6.1 (5.3 to 7) | 157.1 (136.6 to 179) | 151.6 (131.4 to 173.5) | 5.5 (3.9 to 7.6) | 5 (3.3 to 6.9) | 0.4 (0.3 to 0.5) | 19.5 (15.5 to 23.6) | 16.8 (13.7 to 20.2) | 2.7 (1.5 to 4.3) |
| Saint Kitts and Nevis | 133.8 (106.5 to 172.6) | 78.4 (65.7 to 91.7) | 1279.2 (1065.7 to 1512.7) | 1200.7 (998.2 to 1414.8) | 78.5 (53.5 to 113.1) | 5.8 (4.7 to 7) | 3.4 (2.8 to 4.1) | 64.6 (53.5 to 77.9) | 61.7 (51.3 to 74.2) | 2.8 (1.9 to 4) | 3.9 (3.2 to 4.6) | 2.2 (1.8 to 2.6) | 59.8 (49.4 to 71.1) | 58.1 (47.9 to 69.1) | 1.7 (1.1 to 2.4) | 2.3 (1.7 to 3) | 0.5 (0.4 to 0.6) | 21.6 (17.5 to 26.4) | 20.4 (16.6 to 24.9) | 1.2 (0.6 to 2.1) |
| Saint Lucia | 117.7 (92.2 to 150.2) | 55.6 (45.1 to 66.8) | 994.4 (823.6 to 1195.7) | 927.8 (756.3 to 1124) | 66.6 (45.9 to 93.6) | 6 (4.7 to 7.7) | 3 (2.5 to 3.8) | 63.9 (52.2 to 78.8) | 60.9 (49.3 to 76) | 2.9 (1.9 to 4.1) | 3.1 (2.6 to 3.9) | 1.5 (1.2 to 1.9) | 46.5 (38.1 to 58.4) | 45.3 (36.7 to 56.8) | 1.3 (0.9 to 1.9) | 2.5 (1.7 to 3.4) | 0.4 (0.3 to 0.5) | 19.8 (16.2 to 24.6) | 18.5 (15 to 23) | 1.2 (0.7 to 2.1) |
| Saint Vincent and the Grenadines | 116.3 (91.1 to 146.7) | 63.4 (52.8 to 74.4) | 1122 (939.6 to 1337.3) | 1059.2 (889.4 to 1254.9) | 62.8 (41.4 to 89.6) | 4.9 (3.9 to 6.1) | 2.6 (2.1 to 3.2) | 56.2 (45.1 to 69.1) | 53.9 (43.1 to 65.6) | 2.3 (1.5 to 3.2) | 2.4 (1.9 to 3) | 1.2 (1 to 1.5) | 38.5 (30.7 to 48) | 37.6 (30 to 46.7) | 0.9 (0.6 to 1.3) | 1.3 (0.9 to 1.9) | 0.3 (0.2 to 0.4) | 13.3 (10.6 to 16.9) | 12.7 (10.2 to 16.2) | 0.6 (0.3 to 1) |
| Samoa | 11.5 (5.1 to 18.8) | 7.6 (3.3 to 12.6) | 139.3 (59.5 to 232.6) | 132.9 (56.8 to 221.8) | 6.4 (2.7 to 11.5) | 3.2 (1.9 to 5.2) | 1.6 (1 to 2.6) | 36.8 (22.3 to 57.9) | 35.1 (21.1 to 55.5) | 1.7 (1 to 2.6) | 0.6 (0.3 to 1) | 0.3 (0.2 to 0.5) | 8.5 (5 to 14.5) | 8.3 (4.9 to 14) | 0.2 (0.1 to 0.5) | 1.7 (0.9 to 2.8) | 0.5 (0.3 to 0.7) | 22.5 (13.3 to 32.7) | 21.8 (12.8 to 31.5) | 0.7 (0.3 to 1.3) |
| San Marino | 60.9 (37.2 to 87.8) | 15.5 (10.1 to 21.9) | 257.8 (166.7 to 358.3) | 216.5 (142.9 to 304.3) | 41.4 (23 to 64.8) | 22.8 (14.2 to 34.7) | 8.5 (5.4 to 12.8) | 149.8 (95.5 to 222.2) | 138 (86.9 to 201.8) | 11.8 (7.1 to 18.9) | 6.9 (4.3 to 11.1) | 2.6 (1.6 to 4.1) | 58.3 (36.8 to 89.4) | 55.3 (35.2 to 84.9) | 3 (1.4 to 5.5) | 13.7 (7.7 to 21.5) | 0.3 (0.2 to 0.4) | 19.7 (11.8 to 31.7) | 13.5 (8.2 to 20.5) | 6.2 (3 to 11.6) |
| Sao Tome and Principe | 29.4 (19.1 to 41.4) | 22.4 (14.2 to 32.8) | 395.8 (261.6 to 558.8) | 382 (250.7 to 543.6) | 13.8 (8.3 to 20.6) | 15.4 (9.1 to 23.9) | 9.5 (5.4 to 14.6) | 199.9 (114.5 to 299.1) | 193.7 (110.8 to 289.9) | 6.1 (3.5 to 9.9) | 1.6 (0.9 to 2.4) | 0.9 (0.5 to 1.4) | 30.5 (18.5 to 45.7) | 30 (18.2 to 45) | 0.5 (0.3 to 0.9) | 0.5 (0.3 to 0.9) | 0.2 (0.1 to 0.2) | 7.5 (4.1 to 11.9) | 7.3 (4 to 11.8) | 0.2 (0.1 to 0.4) |
| Saudi Arabia | 24.9 (16.2 to 39.5) | 13.7 (9.6 to 19.7) | 208.7 (144.3 to 315.7) | 196.4 (136.2 to 295.7) | 12.3 (6.9 to 20.9) | 10.4 (7.3 to 15.4) | 5.5 (3.9 to 7.8) | 96.6 (67.4 to 143.7) | 92.2 (64.2 to 137.7) | 4.4 (2.9 to 6.6) | 3.2 (2.1 to 4.9) | 1.7 (1.1 to 2.6) | 40.6 (26.6 to 61.8) | 39.5 (26 to 59.9) | 1.1 (0.7 to 2) | 0.9 (0.5 to 1.6) | 0.1 (0.1 to 0.2) | 4.6 (2.5 to 6.9) | 4.1 (2.3 to 6.2) | 0.5 (0.2 to 0.9) |
| Senegal | 31.7 (18.4 to 47.9) | 26.2 (15.1 to 39) | 473.4 (278.5 to 720.4) | 458.6 (269 to 700) | 14.7 (8.2 to 24.6) | 4.2 (2.5 to 6.5) | 2.8 (1.7 to 4.2) | 60 (35.8 to 88.2) | 58.2 (34.5 to 85.2) | 1.8 (1 to 2.9) | 1.5 (0.9 to 2.4) | 1 (0.5 to 1.5) | 31.3 (18 to 48) | 30.8 (17.6 to 47.3) | 0.5 (0.3 to 0.9) | 0.4 (0.2 to 0.6) | 0.1 (0.1 to 0.2) | 6.7 (3.7 to 10.7) | 6.6 (3.6 to 10.6) | 0.1 (0 to 0.2) |
| Serbia | 41 (29.8 to 52.1) | 20.7 (15.7 to 26.1) | 349.4 (263.2 to 428.8) | 325.9 (246.7 to 402.4) | 23.5 (14.7 to 33.8) | 21.8 (17.6 to 27.4) | 10.3 (8.5 to 12.6) | 199.7 (163.1 to 246) | 189.3 (153.3 to 230.8) | 10.4 (7 to 14.5) | 9.2 (6.1 to 12.9) | 4.8 (3.2 to 6.4) | 118.3 (77.9 to 160.5) | 114.9 (75.9 to 155.1) | 3.5 (2.1 to 5.4) | 6.6 (3.9 to 10.4) | 0.8 (0.5 to 1.1) | 35.9 (23.7 to 50.3) | 32.6 (21.4 to 45.1) | 3.3 (1.7 to 6) |
| Seychelles | 90 (60.9 to 125.4) | 52.4 (35 to 72.7) | 909.5 (621.2 to 1245.8) | 855.8 (577.7 to 1179.7) | 53.7 (31.6 to 80.3) | 12.8 (9.1 to 18.4) | 6.2 (4.3 to 8.6) | 126.8 (89.8 to 174.4) | 120.2 (83.7 to 165.3) | 6.5 (4.1 to 10.3) | 2.2 (1.4 to 3.1) | 1 (0.6 to 1.4) | 27.4 (17.3 to 37.9) | 26.5 (16.8 to 36.6) | 0.9 (0.5 to 1.5) | 3.3 (1.7 to 5.7) | 0.6 (0.3 to 0.9) | 28.9 (16.5 to 44.7) | 27.4 (15.7 to 41.8) | 1.5 (0.7 to 2.9) |
| Singapore | 28.5 (21.8 to 35.8) | 8.2 (7.1 to 9.1) | 138.6 (122.4 to 156) | 120.1 (107 to 133.5) | 18.6 (12.5 to 26.1) | 8 (6.3 to 10.3) | 2.6 (2.2 to 3.1) | 45.9 (38.6 to 55) | 41.5 (34.6 to 49.5) | 4.3 (3 to 6.1) | 7 (5.3 to 8.6) | 2.9 (2.3 to 3.5) | 65.1 (53.4 to 77.9) | 62.2 (51.2 to 74.6) | 2.9 (1.9 to 4) | 3.2 (2.2 to 4.5) | 0.1 (0.1 to 0.1) | 6.4 (5.1 to 8.4) | 4.6 (3.8 to 5.7) | 1.8 (1 to 3) |
| Slovakia | 46.3 (33.4 to 58.8) | 22 (16 to 25.3) | 381.7 (284.4 to 439.7) | 354.5 (264.4 to 406.7) | 27.1 (18.2 to 39.1) | 16.5 (14.1 to 19.2) | 8.8 (7.3 to 10) | 166.9 (142.2 to 186.2) | 159.4 (135.4 to 177.2) | 7.5 (5.1 to 10.2) | 13.3 (8.5 to 17.2) | 6.8 (4.2 to 8.5) | 166.7 (104.9 to 210.3) | 161.7 (101.2 to 204.3) | 5 (3 to 7.6) | 10.2 (5.7 to 15.7) | 0.8 (0.5 to 1.2) | 43 (27 to 63.3) | 37.7 (23.8 to 54.7) | 5.3 (2.6 to 9.4) |
| Slovenia | 75.9 (63.2 to 91.2) | 26 (23.7 to 28.3) | 430.2 (393.3 to 464) | 385.6 (356.4 to 414.4) | 44.5 (32 to 62.1) | 15.5 (13.1 to 18) | 8.5 (7.4 to 9.5) | 152.9 (133.7 to 171.6) | 145.9 (127.8 to 163.3) | 7.1 (5 to 9.8) | 13.7 (11.3 to 16.4) | 5.4 (4.6 to 6.2) | 120.8 (103.2 to 137.6) | 115.2 (98.3 to 130.3) | 5.6 (3.7 to 7.9) | 9.1 (6.2 to 12.2) | 0.4 (0.3 to 0.4) | 20 (15.4 to 25.4) | 15.1 (12.3 to 18) | 4.8 (2.6 to 8.1) |
| Solomon Islands | 48.4 (31.1 to 72.8) | 38.7 (25 to 57) | 690.5 (449.3 to 1022.6) | 667.1 (432.3 to 988.6) | 23.3 (13.8 to 37.4) | 6.5 (3.8 to 10.3) | 3.5 (1.9 to 5.5) | 84.2 (46.6 to 133.2) | 81.2 (44.5 to 128.5) | 3.1 (1.6 to 5.3) | 1.5 (0.7 to 2.5) | 0.9 (0.4 to 1.6) | 24.9 (10.9 to 42.9) | 24.3 (10.7 to 42.1) | 0.5 (0.2 to 1) | 0.3 (0.1 to 0.4) | 0.2 (0.1 to 0.3) | 4.3 (2.2 to 7.6) | 4.3 (2.1 to 7.5) | 0.1 (0 to 0.1) |
| South Africa | 50 (37.5 to 63) | 37.1 (28 to 46) | 675.7 (522.7 to 832) | 649.7 (498.6 to 797) | 25.9 (17 to 35.8) | 6.4 (4.7 to 8.9) | 4 (2.9 to 5.4) | 83.3 (62.4 to 113.2) | 80.3 (59.9 to 109.8) | 3 (2 to 4.3) | 2.6 (1.7 to 3.6) | 1.7 (1.1 to 2.4) | 48.1 (31.5 to 66.2) | 47.1 (30.9 to 64.8) | 0.9 (0.6 to 1.4) | 0.9 (0.5 to 1.4) | 0.4 (0.3 to 0.6) | 13.4 (7.8 to 19.5) | 13.1 (7.6 to 19.2) | 0.3 (0.2 to 0.5) |
| Spain | 58.2 (44.9 to 73.1) | 14.4 (12.7 to 15.9) | 249.5 (219.3 to 275.9) | 209.6 (186.9 to 230.6) | 40 (26.6 to 55.5) | 27.1 (23.1 to 31.9) | 9.6 (8.7 to 10.6) | 174.4 (157 to 193.3) | 159.9 (146.1 to 177.2) | 14.5 (10.3 to 19.4) | 12 (10.2 to 13.9) | 4.8 (4.3 to 5.2) | 107.7 (97.6 to 117.9) | 102.7 (93.3 to 113) | 5 (3.5 to 6.9) | 6.4 (4.5 to 8.1) | 0.2 (0.1 to 0.2) | 10.8 (8.7 to 13.3) | 7.3 (6.3 to 8.6) | 3.5 (1.9 to 5.4) |
| Sri Lanka | 22.1 (14.2 to 30.5) | 9.1 (6 to 12.9) | 175.4 (119.7 to 245) | 160.5 (108.5 to 227) | 14.9 (8.8 to 22.9) | 6.8 (4.9 to 8.9) | 2.8 (2 to 3.6) | 60.4 (44.8 to 77.3) | 56.4 (41.1 to 71.4) | 4 (2.6 to 6) | 3.5 (2.5 to 5.4) | 1.4 (1 to 2) | 37.8 (26.5 to 55.2) | 36.1 (25.3 to 52.2) | 1.7 (1 to 2.9) | 1.9 (1 to 3.1) | 0.3 (0.2 to 0.4) | 11.9 (6.9 to 18) | 10.9 (6.2 to 16.4) | 0.9 (0.4 to 1.9) |
| Suriname | 58.7 (41.3 to 73.4) | 39.1 (27.8 to 47.1) | 650.8 (453.9 to 787.8) | 618.5 (432.6 to 751) | 32.3 (20.1 to 46.1) | 4.4 (2.9 to 6.5) | 2.7 (1.8 to 3.9) | 53.8 (36 to 77) | 51.7 (34.8 to 74.3) | 2.1 (1.3 to 3.3) | 3.4 (2.3 to 4.9) | 2 (1.3 to 2.8) | 59.2 (38.8 to 84.3) | 57.8 (37.7 to 82.5) | 1.4 (0.8 to 2.2) | 1.4 (0.8 to 2.1) | 0.4 (0.2 to 0.5) | 17.2 (10.4 to 26.5) | 16.6 (10.2 to 25.6) | 0.6 (0.2 to 1.2) |
| Sweden | 81 (68 to 95) | 24.2 (21.2 to 27) | 388.9 (339 to 432.3) | 335.6 (296.6 to 372.1) | 53.3 (37.7 to 72.5) | 12.7 (10.8 to 14.8) | 5 (4.6 to 5.5) | 85 (77.7 to 92.6) | 78.4 (71.7 to 85.8) | 6.6 (4.7 to 9.1) | 9.2 (7.8 to 10.6) | 4.4 (4 to 4.9) | 94.8 (85.1 to 105.5) | 91.3 (81.9 to 101.6) | 3.6 (2.4 to 4.8) | 5.9 (4.2 to 7.7) | 0.2 (0.1 to 0.2) | 9.8 (7.7 to 12.8) | 6.7 (5.5 to 8.5) | 3.1 (1.7 to 4.9) |
| Switzerland | 69 (53.5 to 87.7) | 18.3 (16 to 20.3) | 299.2 (260.2 to 336.6) | 252.8 (222.8 to 280.4) | 46.4 (30.4 to 66.7) | 14.1 (11.6 to 16.3) | 5.4 (4.8 to 6.1) | 93.3 (82.3 to 105.6) | 86 (75.8 to 97) | 7.3 (5.2 to 10.2) | 7.9 (6.3 to 9.7) | 3.2 (2.7 to 3.7) | 67 (56.4 to 77.2) | 63.7 (53.8 to 73.7) | 3.3 (2 to 4.4) | 6.2 (4.6 to 7.8) | 0.2 (0.1 to 0.2) | 10 (7.7 to 12.7) | 6.7 (5.5 to 8.1) | 3.3 (2 to 5.1) |
| Taiwan (Province of China) | 45.7 (38 to 54.1) | 12.3 (11.1 to 13.4) | 219.1 (197.3 to 238.9) | 190.4 (174.2 to 206.8) | 28.7 (19.8 to 40) | 17.6 (14.6 to 21.1) | 5.7 (5 to 6.4) | 114.3 (100.1 to 130.9) | 104.5 (92.2 to 119.2) | 9.9 (7.1 to 13.5) | 17.5 (14 to 22) | 4.7 (3.9 to 5.6) | 120.1 (99.9 to 143.9) | 112.3 (93.3 to 134.7) | 7.7 (5.1 to 11.3) | 3.6 (2.5 to 4.8) | 0.2 (0.2 to 0.2) | 10 (7.9 to 12.7) | 8.1 (6.5 to 10) | 1.9 (1 to 3.2) |
| Tajikistan | 9.3 (6.2 to 13.9) | 6.3 (4.3 to 9.2) | 122 (84.2 to 178.6) | 117 (80.6 to 172) | 5 (3 to 7.8) | 2.4 (1.6 to 3.7) | 1.6 (1 to 2.5) | 32.6 (20.6 to 50) | 31.5 (19.9 to 48.3) | 1.1 (0.7 to 1.8) | 4.6 (3 to 7.3) | 3.1 (1.9 to 4.8) | 80.3 (51 to 124.6) | 78.7 (50.1 to 122.4) | 1.6 (0.8 to 2.8) | 0 (0 to 0.1) | 0 (0 to 0) | 0.6 (0.3 to 0.9) | 0.6 (0.3 to 0.8) | 0 (0 to 0) |
| Thailand | 29.4 (18.9 to 49.3) | 12.1 (8.1 to 19.2) | 235.5 (161 to 355.9) | 217.2 (149 to 330) | 18.3 (9.9 to 29.8) | 9.2 (7.2 to 12.4) | 3.5 (2.7 to 4.5) | 79 (64.3 to 103.7) | 73.9 (59.9 to 96.7) | 5.2 (3.4 to 7.4) | 5.7 (4.1 to 9.1) | 2 (1.5 to 3.3) | 61.8 (45.2 to 93.9) | 59.3 (43.2 to 90.7) | 2.5 (1.6 to 4.5) | 2.5 (1.6 to 4.1) | 0.3 (0.2 to 0.4) | 15.5 (11.3 to 22.7) | 14.2 (10.5 to 21) | 1.3 (0.7 to 2.2) |
| Timor-Leste | 12.7 (7.7 to 20.1) | 9.7 (5.9 to 14.9) | 171.8 (105.1 to 258.8) | 165.2 (101.3 to 249.4) | 6.6 (3.7 to 10.3) | 2.6 (1.5 to 4.3) | 1.4 (0.8 to 2.3) | 31.2 (17.2 to 51.4) | 29.8 (16.4 to 49.6) | 1.3 (0.8 to 2.3) | 1.8 (1 to 2.8) | 1 (0.6 to 1.6) | 29.2 (16.9 to 45.9) | 28.5 (16.4 to 44.9) | 0.7 (0.4 to 1.2) | 0.5 (0.3 to 0.9) | 0.2 (0.1 to 0.3) | 8.5 (4.9 to 13.6) | 8.3 (4.8 to 13.4) | 0.2 (0.1 to 0.3) |
| Tonga | 46.5 (29.6 to 67.1) | 32.6 (20.4 to 46.3) | 534.2 (333.4 to 755.9) | 510.7 (316 to 724.5) | 23.5 (13.5 to 36.7) | 4.6 (2.7 to 7.2) | 2.3 (1.4 to 3.4) | 50.2 (30.3 to 76) | 47.9 (28.5 to 72.6) | 2.3 (1.3 to 3.8) | 1.6 (0.8 to 2.7) | 0.9 (0.4 to 1.4) | 22.5 (11 to 36.1) | 21.8 (10.7 to 35.2) | 0.6 (0.3 to 1.1) | 0.3 (0.2 to 0.5) | 0.1 (0.1 to 0.2) | 3.5 (2 to 5.6) | 3.4 (1.9 to 5.4) | 0.1 (0 to 0.2) |
| Trinidad and Tobago | 139.9 (112.2 to 173.9) | 81.8 (68.8 to 94.4) | 1262.1 (1064.1 to 1447.1) | 1187.4 (992.8 to 1366.2) | 74.7 (53.7 to 103.4) | 6.6 (5.2 to 8.1) | 3.7 (3.1 to 4.6) | 70.2 (57.6 to 87.2) | 67.2 (55.1 to 83.1) | 3 (2.1 to 4.4) | 4.8 (3.9 to 6) | 2.5 (2 to 3) | 70.3 (57.1 to 85.7) | 68.4 (55.4 to 83.6) | 1.9 (1.3 to 2.7) | 1.6 (1.1 to 2.2) | 0.3 (0.2 to 0.3) | 13 (10.3 to 16.1) | 12.2 (9.7 to 15) | 0.8 (0.4 to 1.3) |
| Tunisia | 50.2 (31 to 82.4) | 20.8 (13.6 to 32.3) | 372 (243.4 to 582.8) | 336.6 (219.7 to 530.2) | 35.5 (18.9 to 58.9) | 19.1 (12.7 to 26.3) | 8.6 (6 to 12) | 176.8 (123.3 to 243.7) | 165.7 (116.3 to 229.4) | 11.1 (6.7 to 16.8) | 5.2 (3.4 to 7.8) | 2.5 (1.7 to 3.6) | 63.8 (42.5 to 92.3) | 61.4 (41.1 to 88.9) | 2.4 (1.3 to 4) | 1.8 (0.9 to 3) | 0.2 (0.1 to 0.3) | 7.8 (4.7 to 12.2) | 6.9 (4.3 to 10.4) | 1 (0.4 to 2) |
| Türkiye | 37.5 (25.5 to 52.8) | 18 (12.6 to 23.8) | 290 (208.5 to 374.7) | 268.4 (191.9 to 353.8) | 21.6 (13.3 to 33.2) | 17.7 (12.4 to 23.2) | 8.3 (6.1 to 10.7) | 161.4 (117.5 to 208.7) | 152.9 (111.4 to 197.3) | 8.6 (5.3 to 12.7) | 6.3 (4.1 to 9) | 3 (2 to 4.3) | 77.5 (51.1 to 108.4) | 75 (49.4 to 105.7) | 2.5 (1.4 to 3.9) | 5.9 (4 to 8.4) | 0.5 (0.4 to 0.7) | 25.1 (19.3 to 31.7) | 22.1 (17.3 to 27.6) | 3 (1.7 to 4.9) |
| Turkmenistan | 7.8 (6.2 to 10) | 4.9 (4 to 5.8) | 103.2 (83.3 to 123.1) | 98.5 (79.7 to 116.3) | 4.7 (3.2 to 6.8) | 3.6 (2.8 to 4.4) | 2.1 (1.7 to 2.6) | 50.3 (39.3 to 61.2) | 48.5 (37.7 to 58.8) | 1.8 (1.1 to 2.6) | 7.3 (5.7 to 9.4) | 4.6 (3.6 to 5.7) | 133.5 (106.3 to 167.1) | 130.9 (104.4 to 163.9) | 2.6 (1.7 to 3.8) | 1.1 (0.8 to 1.6) | 0.4 (0.3 to 0.5) | 14.7 (11 to 19.2) | 14.3 (10.7 to 18.8) | 0.4 (0.2 to 0.7) |
| Tuvalu | 64 (40.5 to 91.4) | 54.5 (35.5 to 76.8) | 868.6 (567.5 to 1224.2) | 840.4 (548.9 to 1189) | 28.1 (16.2 to 41.7) | 8.2 (5 to 12.7) | 4.5 (2.7 to 7) | 100 (59.1 to 154.9) | 96.2 (56.8 to 148.4) | 3.8 (2.1 to 6.3) | 2.6 (1.4 to 4.4) | 1.5 (0.8 to 2.5) | 42.9 (22.1 to 69.7) | 41.9 (21.6 to 68.2) | 0.9 (0.5 to 1.6) | 0.5 (0.3 to 0.9) | 0.3 (0.2 to 0.5) | 8.8 (5.3 to 13.5) | 8.7 (5.2 to 13.3) | 0.1 (0.1 to 0.3) |
| Ukraine | 37.6 (29.9 to 45.7) | 14.4 (12.8 to 16.1) | 310.8 (281.3 to 347.2) | 286 (258.3 to 319.6) | 24.9 (17.5 to 34) | 12.4 (10.4 to 15) | 6.2 (5.3 to 7.3) | 140.3 (119.4 to 165.1) | 134.3 (114.4 to 158.3) | 6 (4.2 to 8.2) | 11.1 (9.6 to 13.2) | 5.8 (5 to 6.9) | 160.4 (139.3 to 191.1) | 156.3 (135.1 to 186.9) | 4.1 (2.9 to 5.7) | 2.8 (1.9 to 3.8) | 0.6 (0.5 to 0.7) | 28 (22.4 to 34.2) | 26.8 (21.6 to 32.8) | 1.2 (0.6 to 2) |
| United Arab Emirates | 16.3 (9.6 to 26.4) | 11.9 (7 to 18.9) | 163.5 (98.4 to 253.4) | 155.5 (93.5 to 241.5) | 8 (4.2 to 13.4) | 4.5 (2.7 to 6.8) | 2.8 (1.8 to 4.2) | 44.8 (27.9 to 66.5) | 42.8 (26.6 to 63.6) | 2 (1.2 to 3.1) | 1.2 (0.7 to 1.7) | 0.8 (0.4 to 1.1) | 15.8 (9.6 to 23.1) | 15.4 (9.3 to 22.5) | 0.4 (0.2 to 0.7) | 0.3 (0.2 to 0.5) | 0.1 (0 to 0.1) | 2 (1.2 to 3.1) | 1.8 (1.1 to 2.8) | 0.2 (0.1 to 0.5) |
| United Kingdom | 82 (70.4 to 94.6) | 24.2 (21.8 to 26.5) | 403 (365.4 to 438.1) | 348.2 (317.7 to 378.9) | 54.9 (38.6 to 74.2) | 18.2 (15.8 to 20.8) | 7.8 (7 to 8.5) | 132.6 (120.6 to 143.2) | 123.6 (113.1 to 134.5) | 9 (6.3 to 12.2) | 12 (10.4 to 13.8) | 5.2 (4.7 to 5.7) | 119.8 (109.9 to 131.1) | 114.9 (105.6 to 125.2) | 4.8 (3.4 to 6.6) | 7.5 (5.5 to 10.1) | 0.2 (0.2 to 0.3) | 13.8 (11.3 to 17.4) | 9.8 (8.3 to 11.9) | 4 (2.4 to 6.3) |
| United Republic of Tanzania | 45.1 (30.6 to 67.5) | 37.1 (24.2 to 55.6) | 683.6 (462.4 to 1014) | 662.8 (448.5 to 988.1) | 20.8 (12.5 to 31.9) | 3.1 (2 to 4.6) | 2.1 (1.3 to 3.2) | 45.2 (28.5 to 68) | 43.9 (27.6 to 65.8) | 1.3 (0.8 to 2.1) | 0.7 (0.4 to 1) | 0.5 (0.3 to 0.7) | 13.8 (7.9 to 20.6) | 13.6 (7.8 to 20.3) | 0.2 (0.1 to 0.4) | 0.4 (0.2 to 0.6) | 0.1 (0.1 to 0.2) | 7.1 (3.7 to 11.1) | 6.9 (3.6 to 11) | 0.1 (0 to 0.2) |
| United States of America | 101.5 (82.3 to 121.9) | 16.2 (13.9 to 18.4) | 324.4 (282.6 to 365.8) | 254 (221.2 to 286.8) | 70.4 (48.6 to 96.4) | 23.1 (20.1 to 25.9) | 5.7 (5 to 6.2) | 106.2 (97 to 116.4) | 93.6 (85.5 to 102.4) | 12.6 (9.3 to 16.6) | 14.9 (12.7 to 17.3) | 4.3 (3.9 to 4.8) | 104.2 (93.9 to 116.3) | 97.7 (88.5 to 108.4) | 6.5 (4.4 to 8.8) | 7.1 (4.9 to 9.5) | 0.3 (0.3 to 0.4) | 18.4 (14.7 to 23.2) | 14.5 (12.1 to 18.1) | 3.8 (2.1 to 6.2) |
| United States Virgin Islands | 104.1 (76.6 to 140) | 40 (31.7 to 49) | 690.2 (556.3 to 851.4) | 633.6 (509.1 to 776.8) | 56.7 (37.8 to 81.7) | 4.9 (3.7 to 6.4) | 2.3 (1.8 to 2.8) | 46.1 (37 to 57) | 43.6 (34.5 to 54.2) | 2.5 (1.6 to 3.5) | 10.1 (7.9 to 12.8) | 4 (3.3 to 4.8) | 125.4 (103.1 to 151.4) | 121.2 (99.7 to 145.8) | 4.2 (2.8 to 6) | 1.9 (1.2 to 2.8) | 0.2 (0.1 to 0.2) | 9.8 (7.7 to 12.6) | 8.8 (6.9 to 11.4) | 1 (0.5 to 1.7) |
| Uruguay | 69.5 (57.2 to 84.7) | 33.9 (30.8 to 37.1) | 580.1 (527.9 to 628.5) | 536.3 (490.9 to 582.7) | 43.8 (29.3 to 58.3) | 19.8 (16.9 to 23.1) | 9.7 (8.5 to 11.2) | 186.1 (163.4 to 209.8) | 176.9 (155.6 to 202.2) | 9.2 (6.3 to 12.8) | 19 (16.1 to 22) | 9.9 (8.9 to 11.1) | 256 (225.9 to 286.9) | 248.9 (219.2 to 277.5) | 7.1 (4.8 to 9.8) | 12.7 (8.2 to 17.9) | 1.1 (0.9 to 1.3) | 60.1 (47.8 to 72.9) | 53.7 (43.3 to 63.7) | 6.4 (3.5 to 9.9) |
| Uzbekistan | 8.3 (6.8 to 10.1) | 4.9 (4.2 to 5.7) | 96.3 (82.9 to 111.5) | 91.5 (78.8 to 105.9) | 4.8 (3.2 to 6.7) | 4 (3.2 to 4.9) | 2.4 (2 to 2.8) | 50.7 (42.5 to 60.1) | 48.8 (40.7 to 57.8) | 1.9 (1.3 to 2.6) | 3.5 (2.9 to 4.2) | 2.1 (1.8 to 2.4) | 58.5 (49.7 to 67.6) | 57.2 (48.6 to 66.1) | 1.3 (0.9 to 1.9) | 1.7 (1.1 to 2.3) | 0.5 (0.4 to 0.6) | 17.3 (13.9 to 21) | 16.6 (13.4 to 20.2) | 0.7 (0.4 to 1.1) |
| Vanuatu | 17.8 (11.2 to 26.5) | 14.7 (9.6 to 22) | 262.7 (170.3 to 397.4) | 253.7 (164.5 to 383.6) | 9 (5.3 to 13.9) | 2.9 (1.7 to 4.6) | 1.5 (0.9 to 2.3) | 38.5 (23.2 to 62.1) | 36.9 (22.1 to 59.5) | 1.6 (0.9 to 2.6) | 1.3 (0.6 to 2.2) | 0.8 (0.4 to 1.4) | 22.2 (10.3 to 37.8) | 21.7 (10.1 to 36.8) | 0.5 (0.2 to 0.8) | 0.1 (0.1 to 0.3) | 0.1 (0 to 0.1) | 2.7 (1.5 to 4.7) | 2.7 (1.5 to 4.6) | 0 (0 to 0.1) |
| Viet Nam | 6.1 (2.6 to 11.3) | 2.8 (1.1 to 5) | 53.4 (22.6 to 91.9) | 49.4 (20.5 to 85.5) | 3.9 (1.6 to 7.7) | 5.3 (3.2 to 8.2) | 2.2 (1.3 to 3.5) | 49 (29.5 to 75) | 46.2 (27.7 to 70.7) | 2.9 (1.6 to 4.7) | 1.7 (0.9 to 2.8) | 0.7 (0.4 to 1.1) | 19.9 (10.8 to 31.4) | 19.1 (10.4 to 30.3) | 0.7 (0.4 to 1.3) | 1.6 (0.8 to 2.7) | 0.2 (0.1 to 0.3) | 10.8 (6.4 to 16.1) | 10 (6 to 15.3) | 0.8 (0.3 to 1.4) |
| Zambia | 64.4 (42 to 92.5) | 54.2 (34.6 to 78.2) | 1004.2 (646.1 to 1452.8) | 975.5 (623.8 to 1412.6) | 28.6 (17.3 to 43.3) | 5.7 (3.6 to 8.3) | 3.7 (2.3 to 5.4) | 83.9 (51.9 to 122.6) | 81.7 (50.5 to 119.4) | 2.2 (1.3 to 3.5) | 1.2 (0.7 to 1.8) | 0.8 (0.4 to 1.2) | 25.6 (15.1 to 38.2) | 25.2 (14.8 to 37.6) | 0.3 (0.2 to 0.6) | 0.5 (0.3 to 0.8) | 0.2 (0.1 to 0.3) | 10.2 (5.6 to 15.8) | 10.1 (5.5 to 15.5) | 0.1 (0.1 to 0.3) |
| Zimbabwe | 30.3 (19.9 to 44.5) | 25.8 (16.3 to 39.4) | 493.6 (318.1 to 735.8) | 477.8 (307.2 to 711.6) | 15.8 (9.7 to 24.6) | 6.8 (4.1 to 10) | 4.6 (2.8 to 6.8) | 104 (61.9 to 155.3) | 101 (59.6 to 150.8) | 2.9 (1.7 to 4.7) | 1 (0.6 to 1.5) | 0.7 (0.5 to 1.1) | 20.4 (13 to 30.6) | 20.1 (12.8 to 30) | 0.3 (0.2 to 0.6) | 0.4 (0.3 to 0.7) | 0.3 (0.2 to 0.5) | 9.1 (5.3 to 14) | 9 (5.2 to 13.9) | 0.1 (0.1 to 0.2) |

## Table S6 Average annual percent change of age-standardized incidence, mortality, DALYs, YLLs, and YLDs rate from 1990 to 2023

| **Locations** | **Prostate cancer** | | | | | **Male bladder cancer** | | | | | **Male kidney cancer** | | | | | **Testicular cancer** | | | | |
| --- | --- | --- | --- | --- | --- | --- | --- | --- | --- | --- | --- | --- | --- | --- | --- | --- | --- | --- | --- | --- |
|  | ASIR AAPC and 95%CI | ASMR AAPC and 95%CI | ASDR AAPC and 95%CI | Age-Standardized YLL Rate AAPC and 95%CI | Age-Standardized YLD Rate AAPC and 95%CI | ASIR AAPC and 95%CI | ASMR AAPC and 95%CI | ASDR AAPC and 95%CI | Age-Standardized YLL Rate AAPC and 95%CI | Age-Standardized YLD Rate AAPC and 95%CI | ASIR AAPC and 95%CI | ASMR AAPC and 95%CI | ASDR AAPC and 95%CI | Age-Standardized YLL Rate AAPC and 95%CI | Age-Standardized YLD Rate AAPC and 95%CI | ASIR AAPC and 95%CI | ASMR AAPC and 95%CI | ASDR AAPC and 95%CI | Age-Standardized YLL Rate AAPC and 95%CI | Age-Standardized YLD Rate AAPC and 95%CI |
| World Bank High Income | 0.17 （0.13 to 0.21) | -1.19 (-1.22 to -1.16) | -1.10 (-1.13 to -1.06) | -1.25 (-1.30 to -1.21) | 0.18 (0.14 to 0.22) | -0.42 (-0.48 to -0.38) | -0.89 (-0.94 to -0.85) | -1.11 (-1.16 to -1.07) | -1.18 (-1.23 to -1.14) | -0.22 (-0.26 to -0.19) | 0.49 (0.45 to 0.52) | -0.27 (-0.32 to -0.23) | -0.60 (-0.65 to -0.54) | -0.64 (-0.70 to -0.58) | 0.72 (0.68 to 0.74) | 1.04 (0.98 to 1.11) | -1.30 (-1.38 to -1.21) | -0.97 (-1.04 to -0.90) | -1.33 (-1.40 to -1.28） | 1.14 (1.07 to 1.21) |
| World Bank Upper Middle Income | 1.17 (1.13 to 1.21) | -0.35 (-0.40 to -0.30) | -0.25 (-0.30 to -0.20) | -0.36 (-0.40 to -0.31) | 1.46 (1.40 to 1.51) | -0.09 (-0.18 to 0.01) | -0.79 (-0.90 to -0.64) | -0.98 (-1.08 to -0.84) | -1.03 (-1.14 to -0.89) | 0.32 (0.24 to 0.40) | 1.38 (1.34 to 1.42) | 0.28 (0.23 to 0.32) | 0.12 (0.08 to 0.16) | 0.07 (0.03 to 0.11) | 1.71 (1.66 to 1.76) | 3.42 (3.35 to 3.49) | 0.09 (0.02 to 0.17) | 0.70 (0.64 to 0.79) | 0.54 (0.47 to 0.62) | 4.57 (4.49 to 4.66) |
| World Bank Lower Middle Income | 1.26 (1.20 to 1.32) | 0.42 (0.35 to 0.48) | 0.40 (0.33 to 0.45) | 0.36 (0.29 to 0.41) | 1.61 (1.56 to 1.65) | 0.38 (0.32 to 0.45) | -0.08 (-0.15 to -0.02) | -0.24 (-0.31 to -0.17) | -0.27 (-0.34 to -0.19) | 0.52 (0.45 to 0.56) | 1.40 (1.34 to 1.45) | 0.86 (0.80 to 0.93) | 0.78 (0.72 to 0.84) | 0.77 (0.70 to 0.83) | 1.59 (1.52 to 1.64) | 2.34 (2.23 to 2.48) | 0.35 (0.20 to 0.51) | 0.32 (0.17 to 0.49) | 0.26 (0.09 to 0.44) | 3.45 (3.24 to 3.62) |
| Albania | 1.6 (1.6 to 1.7) | 0.1 (0.1 to 0.2) | 0.2 (0.1 to 0.3) | 0.1 (0.0 to 0.2) | 2.1 (2.0 to 2.2) | 1.1 (0.9 to 1.2) | 0.2 (0.1 to 0.4) | 0.3 (0.2 to 0.4) | 0.3 (0.2 to 0.4) | 1.0 (0.9 to 1.1) | 1.6 (1.5 to 1.8) | 0.8 (0.7 to 1.0) | 0.7 (0.5 to 0.8) | 0.6 (0.5 to 0.8) | 2.0 (1.8 to 2.2) | 2.4 (2.2 to 2.7) | -0.6 (-0.7 to -0.4) | -0.5 (-0.7 to -0.4) | -0.8 (-0.9 to -0.6) | 2.7 (2.4 to 3.1) |
| Algeria | 2.3 (2.1 to 2.5) | 0.8 (0.6 to 0.9) | 0.9 (0.7 to 1.1) | 0.8 (0.6 to 1.0) | 2.5 (2.4 to 2.7) | 0.7 (0.4 to 1.0) | 0.1 (-0.2 to 0.4) | 0.1 (-0.2 to 0.4) | 0.1 (-0.2 to 0.3) | 0.7 (0.5 to 0.9) | 1.8 (1.7 to 1.9) | 1.0 (0.9 to 1.1) | 0.8 (0.7 to 0.9) | 0.8 (0.7 to 0.8) | 2.0 (1.9 to 2.1) | 2.0 (1.7 to 2.3) | -0.8 (-1.1 to -0.6) | -0.8 (-1.1 to -0.5) | -1.0 (-1.2 to -0.7) | 2.6 (2.4 to 2.8) |
| American Samoa | 0.5 (0.2 to 0.7) | 0.4 (0.2 to 0.5) | 0.2 (-0.0 to 0.4) | 0.2 (-0.0 to 0.4) | 0.4 (0.2 to 0.7) | 0.8 (0.5 to 1.1) | 0.5 (0.2 to 0.8) | 0.5 (0.2 to 0.8) | 0.5 (0.2 to 0.7) | 0.8 (0.5 to 1.0) | 0.2 (0.1 to 0.4) | 0.3 (0.1 to 0.4) | 0.3 (0.1 to 0.4) | 0.3 (0.1 to 0.4) | 0.1 (-0.1 to 0.3) | 3.9 (3.3 to 4.4) | 3.4 (2.4 to 4.4) | 3.6 (3.0 to 4.2) | 3.6 (3.0 to 4.2) | 3.6 (3.0 to 4.3) |
| Andorra | 1.6 (1.4 to 1.8) | -0.4 (-0.4 to -0.3) | -0.1 (-0.2 to -0.1) | -0.4 (-0.4 to -0.3) | 1.5 (1.3 to 1.7) | -0.2 (-0.3 to -0.1) | -1.0 (-1.1 to -1.0) | -1.0 (-1.0 to -0.9) | -1.0 (-1.1 to -1.0) | -0.0 (-0.1 to 0.1) | -0.2 (-0.4 to 0.1) | -1.4 (-1.6 to -1.2) | -1.4 (-1.5 to -1.2) | -1.4 (-1.6 to -1.2) | 0.0 (-0.3 to 0.3) | 2.0 (1.8 to 2.1) | -0.5 (-0.7 to -0.2) | 0.1 (-0.1 to 0.3) | -0.4 (-0.7 to -0.2) | 1.6 (1.4 to 1.8) |
| Angola | 1.4 (1.3 to 1.5) | 0.8 (0.7 to 0.9) | 0.9 (0.8 to 1.1) | 0.9 (0.8 to 1.0) | 1.8 (1.7 to 1.9) | 1.4 (1.2 to 1.5) | 1.1 (0.9 to 1.2) | 1.1 (0.9 to 1.3) | 1.1 (0.9 to 1.3) | 1.4 (1.2 to 1.6) | 1.6 (1.4 to 1.7) | 1.2 (1.1 to 1.3) | 1.1 (1.0 to 1.2) | 1.1 (1.0 to 1.2) | 1.5 (1.4 to 1.7) | 1.8 (1.5 to 2.2) | 0.8 (0.5 to 1.0) | 0.8 (0.4 to 1.0) | 0.7 (0.4 to 1.0) | 2.2 (1.8 to 2.5) |
| Antigua and Barbuda | 0.0 (-0.3 to 0.3) | -0.6 (-0.9 to -0.3) | -0.7 (-0.9 to -0.4) | -0.8 (-1.0 to -0.5) | 0.2 (-0.1 to 0.4) | -0.2 (-0.4 to -0.0) | -0.5 (-0.7 to -0.3) | -0.6 (-0.8 to -0.5) | -0.6 (-0.8 to -0.5) | 0.1 (-0.0 to 0.2) | 0.3 (0.1 to 0.5) | -0.6 (-0.8 to -0.4) | -0.6 (-0.8 to -0.4) | -0.6 (-0.8 to -0.4) | 0.8 (0.5 to 1.0) | 6.2 (4.9 to 7.6) | 4.3 (3.1 to 5.6) | 4.5 (3.2 to 5.7) | 4.3 (3.1 to 5.6) | 6.8 (5.3 to 8.2) |
| Argentina | 0.2 (-0.3 to 0.6) | -0.9 (-1.3 to -0.5) | -0.9 (-1.3 to -0.5) | -1.0 (-1.3 to -0.6) | 0.5 (0.1 to 1.0) | -1.4 (-1.5 to -1.3) | -1.7 (-1.8 to -1.6) | -1.7 (-1.9 to -1.6) | -1.8 (-1.9 to -1.6) | -1.1 (-1.3 to -1.0) | 1.2 (1.0 to 1.4) | 0.7 (0.5 to 0.9) | 0.3 (0.2 to 0.5) | 0.3 (0.2 to 0.5) | 1.4 (1.3 to 1.6) | 2.6 (2.4 to 2.9) | 0.4 (0.3 to 0.6) | 0.7 (0.5 to 0.8) | 0.5 (0.3 to 0.7) | 3.1 (2.9 to 3.4) |
| Armenia | 2.0 (1.6 to 2.4) | 0.9 (0.4 to 1.3) | 0.7 (0.3 to 1.2) | 0.6 (0.2 to 1.1) | 2.2 (1.8 to 2.6) | -0.5 (-0.9 to -0.1) | -0.9 (-1.3 to -0.5) | -1.1 (-1.5 to -0.7) | -1.1 (-1.6 to -0.7) | -0.0 (-0.4 to 0.3) | 0.7 (0.3 to 1.1) | -0.1 (-0.5 to 0.2) | 0.0 (-0.4 to 0.4) | -0.0 (-0.4 to 0.4) | 1.2 (0.8 to 1.6) | 2.5 (2.1 to 2.8) | 0.6 (0.2 to 0.8) | 0.4 (0.1 to 0.8) | 0.3 (-0.0 to 0.6) | 3.2 (2.8 to 3.6) |
| Australia | 0.2 (-0.1 to 0.6) | -1.8 (-1.9 to -1.7) | -1.8 (-1.9 to -1.6) | -2.1 (-2.2 to -2.0) | 0.1 (-0.2 to 0.5) | -1.2 (-1.3 to -1.1) | -1.4 (-1.5 to -1.3) | -1.7 (-1.8 to -1.6) | -1.8 (-1.9 to -1.7) | -1.1 (-1.1 to -1.0) | 0.9 (0.7 to 1.1) | -0.5 (-0.6 to -0.5) | -0.8 (-0.8 to -0.7) | -0.8 (-0.9 to -0.8) | 1.3 (1.0 to 1.5) | 1.4 (1.1 to 1.8) | -1.6 (-2.1 to -1.0) | -0.9 (-1.4 to -0.4) | -1.6 (-2.1 to -1.1) | 1.5 (1.1 to 1.8) |
| Austria | 0.2 (-0.1 to 0.5) | -1.7 (-1.8 to -1.6) | -1.6 (-1.7 to -1.5) | -1.8 (-1.9 to -1.7) | 0.3 (0.1 to 0.6) | -0.5 (-0.6 to -0.4) | -1.2 (-1.3 to -1.1) | -1.4 (-1.5 to -1.3) | -1.5 (-1.6 to -1.4) | -0.2 (-0.3 to -0.1) | -0.1 (-0.3 to 0.0) | -1.1 (-1.2 to -0.9) | -1.4 (-1.5 to -1.3) | -1.5 (-1.6 to -1.4) | 0.3 (0.1 to 0.4) | 0.9 (0.7 to 1.2) | -1.7 (-2.0 to -1.5) | -1.4 (-1.6 to -1.1) | -2.0 (-2.3 to -1.8) | 1.0 (0.7 to 1.3) |
| Azerbaijan | 0.6 (0.5 to 0.8) | -0.2 (-0.4 to -0.0) | -0.3 (-0.5 to -0.2) | -0.4 (-0.5 to -0.2) | 0.9 (0.8 to 1.0) | -0.3 (-0.4 to -0.2) | -0.6 (-0.8 to -0.5) | -0.9 (-1.0 to -0.8) | -0.9 (-1.0 to -0.8) | -0.1 (-0.2 to 0.0) | 0.5 (0.3 to 0.6) | -0.1 (-0.2 to 0.1) | -0.3 (-0.4 to -0.1) | -0.3 (-0.4 to -0.2) | 0.7 (0.6 to 0.8) | 1.3 (1.0 to 1.5) | -0.5 (-0.6 to -0.3) | -0.4 (-0.6 to -0.3) | -0.5 (-0.6 to -0.4) | 2.4 (2.1 to 2.7) |
| Bahamas | 1.2 (0.9 to 1.4) | 0.7 (0.5 to 0.9) | 0.6 (0.3 to 0.8) | 0.5 (0.3 to 0.7) | 1.2 (1.0 to 1.5) | 0.9 (0.7 to 1.1) | 0.7 (0.5 to 0.9) | 0.5 (0.3 to 0.7) | 0.5 (0.3 to 0.7) | 1.0 (0.8 to 1.1) | 1.6 (1.3 to 1.8) | 0.8 (0.6 to 0.9) | 0.7 (0.5 to 0.9) | 0.7 (0.5 to 0.9) | 1.8 (1.6 to 2.0) | 7.2 (5.9 to 8.5) | 5.2 (4.0 to 6.4) | 5.3 (4.2 to 6.4) | 5.2 (4.1 to 6.3) | 7.9 (6.4 to 9.5) |
| Bahrain | 1.4 (1.2 to 1.6) | -0.1 (-0.3 to 0.1) | -0.3 (-0.5 to -0.1) | -0.4 (-0.6 to -0.2) | 1.8 (1.7 to 2.0) | -0.9 (-1.1 to -0.7) | -1.4 (-1.6 to -1.3) | -1.8 (-2.0 to -1.6) | -1.8 (-2.0 to -1.7) | -0.6 (-0.7 to -0.4) | 0.4 (0.3 to 0.6) | -0.6 (-0.8 to -0.4) | -0.9 (-1.1 to -0.8) | -1.0 (-1.1 to -0.8) | 0.8 (0.7 to 0.9) | 2.8 (2.2 to 3.4) | -1.0 (-1.5 to -0.5) | -0.8 (-1.3 to -0.2) | -1.1 (-1.6 to -0.6) | 3.9 (3.3 to 4.4) |
| Bangladesh | 1.5 (1.4 to 1.6) | 0.5 (0.4 to 0.7) | 0.5 (0.3 to 0.6) | 0.4 (0.3 to 0.5) | 1.8 (1.7 to 2.0) | 0.8 (0.6 to 0.9) | 0.2 (0.1 to 0.4) | 0.1 (-0.0 to 0.3) | 0.1 (-0.0 to 0.2) | 0.9 (0.7 to 1.1) | 1.5 (1.3 to 1.6) | 0.7 (0.6 to 0.9) | 0.6 (0.5 to 0.8) | 0.6 (0.5 to 0.8) | 1.6 (1.4 to 1.8) | 1.5 (1.0 to 2.0) | -0.9 (-1.2 to -0.5) | -1.0 (-1.4 to -0.5) | -1.0 (-1.4 to -0.6) | 3.2 (2.6 to 3.8) |
| Barbados | 1.2 (1.1 to 1.4) | 0.4 (0.3 to 0.5) | 0.4 (0.3 to 0.5) | 0.3 (0.2 to 0.4) | 1.3 (1.2 to 1.4) | 0.5 (0.3 to 0.6) | 0.1 (-0.1 to 0.2) | 0.0 (-0.1 to 0.2) | 0.0 (-0.1 to 0.2) | 0.6 (0.5 to 0.7) | 1.1 (1.0 to 1.3) | 0.3 (0.1 to 0.4) | 0.2 (0.1 to 0.3) | 0.2 (0.0 to 0.3) | 1.5 (1.3 to 1.6) | 5.1 (3.6 to 6.7) | 3.3 (1.9 to 4.8) | 3.4 (1.9 to 4.8) | 3.2 (1.8 to 4.6) | 5.5 (3.9 to 7.2) |
| Belarus | 3.7 (3.4 to 4.0) | 1.5 (1.2 to 1.9) | 1.7 (1.4 to 2.1) | 1.5 (1.1 to 1.9) | 3.8 (3.5 to 4.0) | 1.7 (0.2 to 3.3) | 0.8 (-0.8 to 2.3) | 0.8 (-0.8 to 2.3) | 0.8 (-0.8 to 2.3) | 1.9 (0.7 to 3.2) | 3.7 (2.4 to 5.0) | 2.7 (1.4 to 4.0) | 2.5 (1.1 to 3.8) | 2.4 (1.1 to 3.8) | 3.9 (2.7 to 5.2) | 2.2 (1.4 to 3.0) | -0.5 (-1.3 to 0.3) | -0.9 (-1.8 to 0.0) | -1.2 (-2.1 to -0.3) | 2.8 (1.9 to 3.6) |
| Belgium | -0.9 (-1.0 to -0.8) | -2.5 (-2.7 to -2.4) | -2.4 (-2.6 to -2.3) | -2.6 (-2.8 to -2.5) | -0.8 (-0.9 to -0.7) | -1.1 (-1.3 to -0.9) | -1.6 (-1.7 to -1.4) | -1.8 (-2.0 to -1.7) | -1.9 (-2.1 to -1.8) | -0.8 (-1.0 to -0.7) | 0.3 (0.1 to 0.5) | -0.5 (-0.6 to -0.3) | -0.7 (-0.9 to -0.6) | -0.8 (-0.9 to -0.6) | 0.6 (0.4 to 0.9) | 0.5 (0.3 to 0.7) | -1.6 (-1.8 to -1.5) | -1.4 (-1.5 to -1.3) | -1.9 (-2.0 to -1.8) | 0.6 (0.4 to 0.7) |
| Belize | 1.0 (0.5 to 1.6) | 0.7 (0.1 to 1.2) | 0.6 (-0.0 to 1.2) | 0.6 (-0.0 to 1.1) | 1.2 (0.7 to 1.7) | 0.9 (0.6 to 1.1) | 0.5 (0.3 to 0.8) | 0.6 (0.3 to 0.9) | 0.6 (0.3 to 0.9) | 1.1 (0.9 to 1.3) | 1.6 (1.3 to 1.9) | 0.9 (0.6 to 1.3) | 0.9 (0.6 to 1.2) | 0.9 (0.5 to 1.2) | 1.9 (1.6 to 2.2) | 7.6 (6.4 to 8.8) | 6.2 (4.8 to 7.5) | 6.3 (4.9 to 7.6) | 6.2 (4.8 to 7.6) | 8.3 (7.0 to 9.6) |
| Benin | 0.2 (0.1 to 0.2) | -0.3 (-0.4 to -0.2) | -0.2 (-0.3 to -0.2) | -0.3 (-0.3 to -0.2) | 0.6 (0.6 to 0.7) | -0.2 (-0.3 to -0.1) | -0.4 (-0.5 to -0.3) | -0.4 (-0.5 to -0.2) | -0.4 (-0.5 to -0.3) | 0.0 (-0.1 to 0.2) | 0.8 (0.7 to 1.0) | 0.4 (0.4 to 0.6) | 0.4 (0.3 to 0.6) | 0.4 (0.3 to 0.6) | 1.0 (0.9 to 1.1) | 1.0 (0.7 to 1.3) | 0.0 (-0.3 to 0.3) | 0.0 (-0.3 to 0.3) | -0.0 (-0.3 to 0.3) | 1.5 (1.2 to 1.8) |
| Bermuda | 0.5 (0.0 to 0.9) | -1.1 (-1.4 to -0.8) | -1.2 (-1.5 to -0.9) | -1.4 (-1.6 to -1.1) | 0.4 (-0.0 to 0.9) | -0.2 (-0.3 to -0.1) | -1.0 (-1.1 to -0.8) | -1.1 (-1.3 to -0.9) | -1.2 (-1.4 to -1.0) | 0.2 (0.0 to 0.3) | 0.5 (0.3 to 0.6) | -1.2 (-1.4 to -1.1) | -1.1 (-1.3 to -1.0) | -1.2 (-1.4 to -1.1) | 1.0 (0.8 to 1.2) | 9.1 (7.9 to 10.4) | 4.9 (3.9 to 5.9) | 5.8 (5.0 to 6.8) | 5.3 (4.4 to 6.2) | 9.2 (8.0 to 10.5) |
| Bhutan | 0.3 (0.2 to 0.4) | -0.6 (-0.7 to -0.4) | -0.5 (-0.6 to -0.4) | -0.6 (-0.7 to -0.4) | 1.0 (1.0 to 1.1) | -0.4 (-0.5 to -0.3) | -1.0 (-1.1 to -0.8) | -0.8 (-0.9 to -0.7) | -0.8 (-1.0 to -0.7) | 0.1 (0.0 to 0.2) | 1.6 (1.5 to 1.7) | 1.0 (1.0 to 1.1) | 0.9 (0.8 to 0.9) | 0.9 (0.8 to 0.9) | 1.8 (1.7 to 1.9) | 3.5 (3.5 to 3.6) | 1.2 (1.1 to 1.3) | 1.4 (1.4 to 1.5) | 1.4 (1.3 to 1.5) | 4.9 (4.7 to 5.0) |
| Bolivia (Plurinational State of) | 3.4 (3.2 to 3.7) | 1.7 (1.5 to 1.9) | 2.0 (1.8 to 2.2) | 1.9 (1.7 to 2.2) | 3.5 (3.4 to 3.7) | 2.7 (2.5 to 3.0) | 1.8 (1.5 to 2.0) | 1.9 (1.7 to 2.1) | 1.9 (1.6 to 2.1) | 2.4 (2.4 to 2.5) | 2.0 (1.8 to 2.2) | 1.3 (1.2 to 1.5) | 1.1 (0.9 to 1.3) | 1.1 (0.9 to 1.2) | 1.8 (1.6 to 1.9) | 4.8 (4.5 to 5.2) | 3.2 (2.8 to 3.5) | 2.9 (2.5 to 3.2) | 2.8 (2.5 to 3.2) | 6.0 (5.5 to 6.4) |
| Bosnia and Herzegovina | 2.2 (1.9 to 2.5) | 0.6 (0.3 to 0.8) | 0.7 (0.5 to 0.9) | 0.6 (0.4 to 0.8) | 2.5 (2.2 to 2.9) | 1.4 (1.2 to 1.6) | 0.7 (0.6 to 0.9) | 0.6 (0.5 to 0.7) | 0.6 (0.4 to 0.7) | 1.6 (1.4 to 1.8) | 2.2 (1.8 to 2.6) | 1.3 (1.0 to 1.7) | 1.2 (0.9 to 1.5) | 1.2 (0.8 to 1.5) | 2.4 (2.0 to 2.8) | 2.6 (2.3 to 3.0) | -0.3 (-0.4 to -0.3) | -0.3 (-0.3 to -0.2) | -0.5 (-0.6 to -0.4) | 3.2 (2.8 to 3.5) |
| Botswana | 0.6 (0.0 to 1.1) | -0.3 (-0.8 to 0.2) | -0.1 (-0.6 to 0.4) | -0.1 (-0.6 to 0.4) | 1.2 (0.7 to 1.7) | 0.3 (-0.3 to 0.8) | -0.1 (-0.6 to 0.4) | 0.0 (-0.5 to 0.6) | 0.0 (-0.5 to 0.6) | 0.6 (0.1 to 1.0) | 0.5 (0.2 to 0.9) | -0.1 (-0.5 to 0.3) | 0.1 (-0.3 to 0.5) | 0.1 (-0.3 to 0.5) | 0.8 (0.6 to 1.0) | 0.9 (0.2 to 1.7) | -0.4 (-1.1 to 0.3) | -0.6 (-1.2 to 0.1) | -0.6 (-1.3 to 0.0) | 2.0 (1.2 to 2.8) |
| Brazil | 0.9 (0.6 to 1.2) | -0.0 (-0.3 to 0.3) | -0.1 (-0.4 to 0.1) | -0.2 (-0.4 to 0.1) | 1.0 (0.7 to 1.3) | 0.3 (0.2 to 0.3) | -0.0 (-0.1 to 0.0) | -0.3 (-0.3 to -0.2) | -0.3 (-0.4 to -0.2) | 0.4 (0.3 to 0.5) | 2.1 (2.0 to 2.3) | 1.5 (1.4 to 1.6) | 1.1 (1.0 to 1.3) | 1.1 (1.0 to 1.3) | 2.3 (2.1 to 2.5) | 4.0 (3.8 to 4.2) | 1.6 (1.3 to 1.8) | 2.0 (1.7 to 2.2) | 1.9 (1.6 to 2.1) | 4.9 (4.7 to 5.0) |
| Brunei Darussalam | 0.4 (0.3 to 0.6) | -0.4 (-0.6 to -0.3) | -0.5 (-0.6 to -0.4) | -0.6 (-0.6 to -0.4) | 0.9 (0.7 to 1.1) | -0.5 (-0.6 to -0.4) | -0.9 (-1.0 to -0.8) | -1.0 (-1.1 to -0.9) | -1.0 (-1.1 to -0.9) | -0.3 (-0.4 to -0.2) | 0.4 (0.3 to 0.6) | -0.3 (-0.4 to -0.1) | -0.2 (-0.4 to -0.1) | -0.3 (-0.4 to -0.1) | 0.8 (0.7 to 1.0) | 2.0 (1.8 to 2.2) | -0.2 (-0.3 to -0.0) | 0.0 (-0.1 to 0.2) | -0.1 (-0.2 to 0.0) | 2.6 (2.4 to 2.9) |
| Bulgaria | 2.3 (2.1 to 2.5) | 1.1 (1.0 to 1.2) | 1.0 (0.8 to 1.2) | 0.9 (0.7 to 1.0) | 2.4 (2.2 to 2.6) | 1.2 (1.1 to 1.4) | 0.8 (0.7 to 0.9) | 0.6 (0.4 to 0.7) | 0.6 (0.4 to 0.7) | 1.3 (1.1 to 1.4) | 4.1 (3.5 to 4.7) | 3.6 (3.1 to 4.2) | 3.4 (2.9 to 4.0) | 3.4 (2.9 to 4.0) | 4.1 (3.5 to 4.6) | 1.9 (1.6 to 2.2) | -0.1 (-0.4 to 0.1) | -0.0 (-0.3 to 0.3) | -0.2 (-0.6 to 0.1) | 2.2 (1.9 to 2.4) |
| Cabo Verde | 2.0 (1.7 to 2.3) | 1.0 (0.7 to 1.2) | 1.2 (0.9 to 1.5) | 1.1 (0.8 to 1.4) | 2.7 (2.4 to 3.0) | 0.7 (0.5 to 1.0) | -0.0 (-0.2 to 0.2) | 0.3 (0.1 to 0.6) | 0.3 (0.1 to 0.6) | 0.5 (0.3 to 0.7) | 6.2 (5.1 to 7.2) | 5.8 (4.5 to 7.0) | 5.3 (4.2 to 6.4) | 5.3 (4.2 to 6.3) | 5.5 (4.3 to 6.6) | 2.5 (2.2 to 2.7) | 0.4 (0.2 to 0.6) | 0.9 (0.7 to 1.1) | 0.8 (0.6 to 1.0) | 2.8 (2.5 to 3.2) |
| Cambodia | 1.2 (1.2 to 1.3) | 0.5 (0.5 to 0.6) | 0.5 (0.5 to 0.6) | 0.5 (0.4 to 0.5) | 1.7 (1.6 to 1.7) | 0.7 (0.5 to 0.8) | 0.3 (0.1 to 0.4) | 0.0 (-0.1 to 0.2) | 0.0 (-0.2 to 0.2) | 0.8 (0.7 to 0.9) | 0.8 (0.7 to 0.9) | 0.6 (0.6 to 0.7) | 0.2 (0.2 to 0.3) | 0.2 (0.1 to 0.2) | 0.9 (0.8 to 1.0) | -0.8 (-1.2 to -0.5) | -1.7 (-2.0 to -1.4) | -2.4 (-2.8 to -2.1) | -2.5 (-2.8 to -2.1) | 0.0 (-0.4 to 0.4) |
| Cameroon | 1.6 (1.5 to 1.8) | 1.2 (1.1 to 1.3) | 1.2 (1.1 to 1.3) | 1.2 (1.1 to 1.3) | 1.8 (1.7 to 2.0) | -0.1 (-0.5 to 0.2) | -0.2 (-0.5 to 0.0) | -0.3 (-0.6 to -0.0) | -0.3 (-0.6 to -0.0) | -0.3 (-0.7 to -0.0) | 2.0 (1.9 to 2.1) | 1.6 (1.5 to 1.8) | 1.6 (1.4 to 1.6) | 1.5 (1.4 to 1.6) | 1.8 (1.7 to 1.9) | -0.8 (-1.3 to -0.3) | -1.8 (-2.1 to -1.4) | -1.9 (-2.2 to -1.5) | -1.9 (-2.2 to -1.5) | -0.5 (-1.0 to 0.0) |
| Canada | -1.5 (-1.7 to -1.3) | -2.1 (-2.3 to -2.0) | -2.3 (-2.5 to -2.1) | -2.4 (-2.6 to -2.3) | -1.5 (-1.7 to -1.2) | -0.3 (-0.5 to -0.2) | -0.6 (-0.7 to -0.5) | -0.9 (-1.0 to -0.8) | -0.9 (-1.0 to -0.8) | -0.2 (-0.4 to -0.0) | -0.0 (-0.2 to 0.1) | -0.5 (-0.6 to -0.4) | -0.8 (-0.9 to -0.7) | -0.8 (-0.9 to -0.8) | 0.0 (-0.1 to 0.2) | 1.5 (1.3 to 1.7) | -0.7 (-0.9 to -0.6) | -0.1 (-0.3 to 0.1) | -0.6 (-0.9 to -0.4) | 1.6 (1.4 to 1.8) |
| Chile | 1.2 (0.8 to 1.5) | -0.4 (-0.7 to -0.1) | -0.5 (-0.8 to -0.2) | -0.7 (-0.9 to -0.4) | 1.6 (1.2 to 1.9) | -0.1 (-0.2 to 0.1) | -0.7 (-0.9 to -0.5) | -0.8 (-1.0 to -0.7) | -0.9 (-1.1 to -0.7) | 0.2 (0.1 to 0.3) | 1.8 (1.6 to 1.9) | 0.7 (0.6 to 0.8) | 0.5 (0.3 to 0.6) | 0.4 (0.3 to 0.5) | 2.3 (2.1 to 2.4) | 3.3 (2.9 to 3.7) | -0.0 (-0.3 to 0.3) | 0.1 (-0.2 to 0.4) | -0.2 (-0.5 to 0.0) | 3.7 (3.3 to 4.2) |
| China | 1.0 (0.9 to 1.2) | -0.9 (-1.1 to -0.7) | -1.0 (-1.2 to -0.8) | -1.2 (-1.4 to -1.0) | 1.5 (1.4 to 1.7) | -0.6 (-0.8 to -0.3) | -1.7 (-2.0 to -1.5) | -2.0 (-2.2 to -1.7) | -2.0 (-2.3 to -1.8) | 0.0 (-0.2 to 0.2) | 1.8 (1.7 to 2.0) | 0.3 (0.1 to 0.5) | 0.0 (-0.2 to 0.2) | -0.1 (-0.3 to 0.1) | 2.3 (2.2 to 2.5) | 0.0 (-0.3 to 0.4) | -3.4 (-3.8 to -3.0) | -3.6 (-4.0 to -3.2) | -3.8 (-4.2 to -3.4) | 2.0 (1.7 to 2.3) |
| Colombia | 0.9 (0.6 to 1.2) | -1.0 (-1.2 to -0.8) | -1.0 (-1.2 to -0.8) | -1.2 (-1.3 to -1.0) | 1.1 (0.9 to 1.4) | -0.1 (-0.2 to 0.1) | -0.8 (-1.0 to -0.6) | -1.0 (-1.1 to -0.8) | -1.0 (-1.2 to -0.8) | 0.3 (0.2 to 0.5) | 2.2 (2.1 to 2.4) | 1.1 (1.0 to 1.2) | 0.8 (0.7 to 0.9) | 0.8 (0.6 to 0.8) | 2.7 (2.6 to 2.9) | 4.7 (4.5 to 4.9) | 1.2 (1.0 to 1.3) | 1.5 (1.3 to 1.7) | 1.2 (1.1 to 1.4) | 5.6 (5.3 to 5.9) |
| Comoros | 0.8 (0.5 to 1.0) | 0.2 (-0.0 to 0.5) | 0.2 (0.0 to 0.5) | 0.2 (-0.0 to 0.5) | 1.0 (0.8 to 1.2) | 0.1 (-0.1 to 0.4) | -0.0 (-0.3 to 0.2) | -0.1 (-0.3 to 0.2) | -0.1 (-0.4 to 0.2) | 0.2 (-0.0 to 0.4) | 1.1 (1.0 to 1.3) | 0.8 (0.6 to 0.9) | 0.7 (0.5 to 0.8) | 0.7 (0.5 to 0.8) | 1.1 (1.0 to 1.3) | -0.4 (-0.7 to -0.2) | -1.4 (-1.7 to -1.1) | -1.6 (-1.9 to -1.3) | -1.6 (-1.9 to -1.3) | 0.3 (-0.0 to 0.5) |
| Congo | 2.2 (2.0 to 2.4) | 1.5 (1.3 to 1.6) | 1.7 (1.5 to 1.8) | 1.6 (1.5 to 1.8) | 2.3 (2.1 to 2.5) | 2.0 (1.8 to 2.2) | 1.8 (1.6 to 1.9) | 1.8 (1.6 to 1.9) | 1.8 (1.6 to 1.9) | 1.6 (1.4 to 1.8) | 1.2 (1.1 to 1.4) | 0.8 (0.7 to 1.0) | 0.8 (0.6 to 0.9) | 0.8 (0.6 to 0.9) | 1.0 (0.8 to 1.1) | 0.5 (0.3 to 0.8) | -0.2 (-0.4 to 0.0) | -0.6 (-0.9 to -0.4) | -0.6 (-0.9 to -0.4) | 0.6 (0.3 to 0.9) |
| Costa Rica | 1.0 (0.7 to 1.4) | -0.1 (-0.5 to 0.2) | -0.1 (-0.4 to 0.1) | -0.3 (-0.6 to 0.0) | 1.1 (0.8 to 1.5) | 0.5 (0.4 to 0.7) | 0.1 (-0.0 to 0.2) | -0.0 (-0.1 to 0.1) | -0.1 (-0.2 to 0.1) | 0.7 (0.6 to 0.8) | 2.2 (2.0 to 2.4) | 1.2 (1.0 to 1.3) | 1.1 (0.9 to 1.2) | 1.0 (0.9 to 1.2) | 2.5 (2.3 to 2.7) | 5.1 (4.9 to 5.4) | 2.5 (2.3 to 2.8) | 3.0 (2.8 to 3.2) | 2.8 (2.5 to 3.0) | 5.4 (5.1 to 5.7) |
| C么te d'Ivoire | 1.2 (0.9 to 1.6) | 0.6 (0.3 to 0.9) | 0.9 (0.6 to 1.2) | 0.9 (0.6 to 1.2) | 1.7 (1.5 to 2.0) | 0.2 (-0.2 to 0.6) | -0.1 (-0.4 to 0.3) | 0.1 (-0.3 to 0.5) | 0.1 (-0.5 to 0.6) | -1.0 (-1.3 to -0.7) | 1.5 (1.3 to 1.6) | 1.0 (0.9 to 1.1) | 1.1 (0.9 to 1.2) | 1.1 (0.9 to 1.2) | 1.5 (1.4 to 1.7) | 0.0 (-0.6 to 0.7) | -0.8 (-1.5 to -0.2) | -0.9 (-1.6 to -0.3) | -0.9 (-1.6 to -0.3) | 0.4 (-0.4 to 1.2) |
| Croatia | 1.5 (1.2 to 1.8) | 0.0 (-0.2 to 0.2) | -0.0 (-0.2 to 0.1) | -0.2 (-0.4 to 0.0) | 1.6 (1.4 to 1.9) | 0.8 (0.6 to 0.9) | 0.2 (0.1 to 0.4) | 0.0 (-0.1 to 0.2) | 0.0 (-0.1 to 0.1) | 1.0 (0.9 to 1.2) | 1.4 (1.1 to 1.8) | 0.7 (0.4 to 1.1) | 0.4 (0.1 to 0.7) | 0.4 (0.0 to 0.7) | 1.7 (1.3 to 2.1) | 1.9 (1.7 to 2.2) | -0.8 (-0.9 to -0.6) | -0.7 (-0.8 to -0.5) | -1.0 (-1.2 to -0.9) | 2.1 (1.8 to 2.4) |
| Cuba | 2.0 (1.8 to 2.2) | 1.0 (0.9 to 1.2) | 1.0 (0.9 to 1.2) | 1.0 (0.8 to 1.1) | 2.0 (1.8 to 2.2) | 1.2 (1.0 to 1.4) | 0.7 (0.5 to 0.9) | 0.7 (0.5 to 0.9) | 0.7 (0.5 to 0.9) | 1.3 (1.1 to 1.5) | 1.8 (1.6 to 2.0) | 1.1 (0.8 to 1.3) | 0.7 (0.5 to 0.9) | 0.7 (0.5 to 0.9) | 1.9 (1.8 to 2.1) | 5.8 (4.5 to 7.1) | 4.3 (3.2 to 5.4) | 4.0 (2.9 to 5.1) | 3.8 (2.7 to 5.0) | 5.9 (4.6 to 7.3) |
| Cyprus | 2.5 (2.2 to 2.7) | -0.0 (-0.1 to 0.1) | 0.0 (-0.1 to 0.1) | -0.3 (-0.3 to -0.2) | 2.9 (2.6 to 3.2) | 1.3 (1.0 to 1.5) | 0.1 (-0.1 to 0.2) | -0.1 (-0.2 to 0.1) | -0.2 (-0.3 to -0.0) | 1.8 (1.5 to 2.0) | 1.5 (1.3 to 1.6) | -0.2 (-0.3 to -0.1) | -0.2 (-0.3 to -0.2) | -0.3 (-0.4 to -0.2) | 2.2 (2.0 to 2.4) | 3.7 (3.3 to 4.1) | -1.0 (-1.2 to -0.8) | -0.5 (-0.7 to -0.3) | -1.4 (-1.6 to -1.1) | 3.7 (3.3 to 4.2) |
| Czechia | 0.8 (0.4 to 1.3) | -1.0 (-1.3 to -0.7) | -1.0 (-1.3 to -0.7) | -1.2 (-1.4 to -0.9) | 1.0 (0.5 to 1.5) | -0.4 (-0.5 to -0.2) | -1.0 (-1.2 to -0.9) | -1.3 (-1.5 to -1.2) | -1.4 (-1.6 to -1.3) | -0.1 (-0.3 to 0.1) | -0.3 (-0.8 to 0.2) | -1.2 (-1.6 to -0.8) | -1.5 (-1.9 to -1.1) | -1.6 (-2.0 to -1.2) | 0.1 (-0.5 to 0.6) | 1.5 (1.2 to 1.7) | -1.5 (-1.7 to -1.2) | -1.4 (-1.6 to -1.1) | -1.8 (-2.0 to -1.5) | 1.8 (1.5 to 2.0) |
| Denmark | 0.9 (0.5 to 1.3) | -1.3 (-1.5 to -1.0) | -1.5 (-1.8 to -1.3) | -1.8 (-2.0 to -1.5) | 1.2 (0.8 to 1.6) | 0.4 (-0.7 to 1.5) | -0.2 (-1.3 to 0.9) | -0.6 (-1.7 to 0.4) | -0.7 (-1.8 to 0.4) | 0.7 (-0.3 to 1.7) | 1.6 (0.8 to 2.4) | 0.5 (-0.3 to 1.3) | 0.1 (-0.7 to 0.9) | 0.0 (-0.8 to 0.8) | 2.0 (1.3 to 2.8) | 1.1 (0.8 to 1.4) | -2.2 (-2.4 to -2.1) | -1.8 (-1.9 to -1.6) | -2.6 (-2.8 to -2.4) | 1.2 (0.8 to 1.5) |
| Djibouti | 0.7 (0.5 to 0.9) | 0.2 (0.1 to 0.4) | 0.3 (0.1 to 0.4) | 0.2 (0.1 to 0.4) | 0.9 (0.7 to 1.2) | 0.1 (-0.2 to 0.4) | -0.0 (-0.2 to 0.2) | -0.1 (-0.4 to 0.1) | -0.1 (-0.4 to 0.1) | -0.0 (-0.3 to 0.3) | 1.3 (1.2 to 1.5) | 1.0 (0.9 to 1.1) | 0.9 (0.8 to 1.1) | 0.9 (0.8 to 1.1) | 1.3 (1.1 to 1.5) | 0.1 (-0.5 to 0.7) | -0.8 (-1.3 to -0.3) | -0.9 (-1.4 to -0.4) | -0.9 (-1.4 to -0.4) | 0.5 (-0.1 to 1.1) |
| Dominica | 0.1 (-0.1 to 0.3) | -0.1 (-0.2 to 0.0) | -0.1 (-0.2 to 0.0) | -0.1 (-0.3 to -0.0) | 0.2 (-0.0 to 0.4) | 0.7 (0.6 to 0.8) | 0.6 (0.5 to 0.7) | 0.6 (0.5 to 0.6) | 0.6 (0.5 to 0.6) | 0.9 (0.8 to 1.0) | 1.1 (1.0 to 1.1) | 0.6 (0.5 to 0.6) | 0.6 (0.5 to 0.6) | 0.5 (0.5 to 0.6) | 1.3 (1.2 to 1.4) | 5.4 (4.4 to 6.3) | 4.4 (3.5 to 5.2) | 4.6 (3.7 to 5.4) | 4.5 (3.7 to 5.3) | 6.0 (5.0 to 7.1) |
| Dominican Republic | 1.8 (1.6 to 2.1) | 1.6 (1.4 to 1.9) | 1.4 (1.2 to 1.6) | 1.4 (1.2 to 1.6) | 1.7 (1.5 to 1.9) | 1.5 (1.2 to 1.7) | 1.3 (0.9 to 1.6) | 1.2 (0.9 to 1.5) | 1.2 (0.9 to 1.5) | 1.3 (1.2 to 1.5) | 0.8 (0.6 to 1.0) | 0.4 (0.2 to 0.6) | 0.1 (-0.1 to 0.3) | 0.1 (-0.1 to 0.3) | 0.9 (0.7 to 1.0) | 7.3 (6.1 to 8.6) | 6.6 (5.5 to 7.7) | 6.2 (5.1 to 7.3) | 6.1 (5.0 to 7.2) | 8.0 (6.7 to 9.3) |
| Ecuador | 0.7 (0.2 to 1.2) | -1.1 (-1.5 to -0.6) | -1.0 (-1.5 to -0.5) | -1.1 (-1.6 to -0.7) | 1.1 (0.6 to 1.6) | 0.4 (-0.0 to 0.8) | -0.5 (-0.8 to -0.1) | -0.5 (-0.9 to -0.2) | -0.6 (-1.0 to -0.2) | 0.7 (0.3 to 1.1) | 1.6 (1.2 to 2.0) | 0.6 (0.2 to 0.9) | 0.4 (0.0 to 0.8) | 0.4 (-0.0 to 0.8) | 1.9 (1.5 to 2.3) | 8.0 (5.8 to 10.1) | 5.3 (3.2 to 7.4) | 5.5 (3.4 to 7.6) | 5.3 (3.3 to 7.4) | 9.2 (6.8 to 11.5) |
| Egypt | 3.6 (3.3 to 3.9) | 2.4 (2.1 to 2.8) | 2.3 (2.0 to 2.6) | 2.2 (2.0 to 2.5) | 4.0 (3.7 to 4.2) | -2.3 (-2.7 to -1.9) | -2.3 (-2.6 to -1.9) | -3.1 (-3.6 to -2.8) | -3.2 (-3.6 to -2.8) | -2.3 (-2.7 to -2.0) | 2.0 (1.7 to 2.3) | 1.4 (1.1 to 1.7) | 0.9 (0.6 to 1.2) | 0.9 (0.6 to 1.2) | 2.2 (1.9 to 2.6) | 3.9 (3.7 to 4.1) | 1.5 (1.2 to 1.7) | 1.2 (1.0 to 1.3) | 1.0 (0.9 to 1.2) | 5.6 (5.4 to 5.8) |
| El Salvador | 2.7 (2.5 to 3.0) | 1.6 (1.4 to 1.7) | 1.4 (1.3 to 1.6) | 1.3 (1.2 to 1.5) | 2.9 (2.7 to 3.2) | 2.0 (1.8 to 2.2) | 1.6 (1.3 to 1.8) | 1.3 (1.1 to 1.6) | 1.3 (1.0 to 1.6) | 2.0 (1.9 to 2.2) | 2.4 (2.3 to 2.6) | 1.5 (1.4 to 1.6) | 1.3 (1.2 to 1.4) | 1.3 (1.1 to 1.4) | 2.8 (2.6 to 3.0) | 4.0 (3.5 to 4.5) | 1.8 (1.5 to 2.0) | 1.7 (1.4 to 2.0) | 1.5 (1.2 to 1.8) | 5.2 (4.5 to 5.8) |
| Equatorial Guinea | 2.6 (2.5 to 2.8) | 1.4 (1.3 to 1.5) | 1.6 (1.4 to 1.7) | 1.5 (1.4 to 1.7) | 3.1 (2.9 to 3.3) | 2.9 (2.7 to 3.2) | 2.2 (2.0 to 2.4) | 2.3 (2.1 to 2.5) | 2.3 (2.1 to 2.5) | 2.9 (2.6 to 3.2) | 2.4 (2.3 to 2.5) | 1.7 (1.6 to 1.8) | 1.5 (1.4 to 1.6) | 1.5 (1.4 to 1.6) | 2.1 (2.0 to 2.3) | 3.1 (2.8 to 3.5) | 1.0 (0.7 to 1.3) | 0.7 (0.3 to 1.0) | 0.6 (0.3 to 0.9) | 4.2 (3.8 to 4.6) |
| Estonia | 3.3 (2.8 to 3.7) | 1.0 (0.7 to 1.3) | 0.9 (0.6 to 1.2) | 0.6 (0.3 to 0.9) | 3.3 (2.8 to 3.8) | 0.1 (-0.2 to 0.3) | -0.7 (-1.0 to -0.5) | -1.1 (-1.4 to -0.8) | -1.2 (-1.5 to -0.9) | 0.6 (0.3 to 0.9) | 1.7 (1.2 to 2.2) | 0.6 (0.1 to 1.1) | 0.1 (-0.4 to 0.6) | -0.0 (-0.5 to 0.5) | 2.1 (1.6 to 2.6) | 1.0 (0.6 to 1.5) | -2.6 (-2.9 to -2.3) | -2.8 (-3.2 to -2.5) | -3.2 (-3.6 to -2.9) | 1.7 (1.2 to 2.3) |
| Eswatini | -1.6 (-1.9 to -1.2) | -1.6 (-1.9 to -1.3) | -1.9 (-2.2 to -1.6) | -2.0 (-2.3 to -1.6) | -1.1 (-1.4 to -0.7) | -2.4 (-2.7 to -2.0) | -2.0 (-2.3 to -1.7) | -2.5 (-2.8 to -2.2) | -2.5 (-2.8 to -2.2) | -2.1 (-2.5 to -1.8) | 0.1 (-0.2 to 0.3) | -0.1 (-0.4 to 0.2) | -0.2 (-0.5 to 0.1) | -0.2 (-0.5 to 0.1) | 0.1 (-0.4 to 0.6) | -1.5 (-1.9 to -1.0) | -2.0 (-2.5 to -1.5) | -1.8 (-2.4 to -1.3) | -1.8 (-2.4 to -1.3) | -1.1 (-1.7 to -0.4) |
| Fiji | 1.1 (1.0 to 1.3) | 1.0 (0.8 to 1.1) | 0.8 (0.7 to 0.9) | 0.8 (0.6 to 0.9) | 1.1 (1.0 to 1.3) | 1.8 (1.6 to 2.0) | 1.7 (1.4 to 1.9) | 1.5 (1.2 to 1.7) | 1.5 (1.2 to 1.7) | 1.6 (1.4 to 1.8) | -0.6 (-0.8 to -0.4) | -0.6 (-0.8 to -0.4) | -0.6 (-0.8 to -0.4) | -0.6 (-0.8 to -0.4) | -0.6 (-0.9 to -0.3) | 1.5 (1.2 to 1.8) | 1.4 (1.1 to 1.7) | 1.1 (0.8 to 1.4) | 1.1 (0.8 to 1.4) | 1.7 (1.4 to 2.0) |
| Finland | 0.6 (0.2 to 1.1) | -1.6 (-1.8 to -1.5) | -1.6 (-1.7 to -1.4) | -1.9 (-2.0 to -1.8) | 0.7 (0.3 to 1.1) | -0.4 (-0.6 to -0.3) | -1.2 (-1.3 to -1.1) | -1.4 (-1.6 to -1.3) | -1.5 (-1.6 to -1.4) | -0.1 (-0.2 to 0.1) | -0.2 (-0.3 to 0.0) | -1.1 (-1.2 to -1.0) | -1.4 (-1.6 to -1.3) | -1.5 (-1.6 to -1.4) | 0.2 (0.0 to 0.4) | 1.8 (1.4 to 2.1) | -1.8 (-2.0 to -1.6) | -1.1 (-1.4 to -0.9) | -1.8 (-2.0 to -1.6) | 1.9 (1.5 to 2.2) |
| France | 0.7 (0.5 to 0.9) | -2.4 (-2.5 to -2.2) | -2.1 (-2.2 to -1.9) | -2.5 (-2.7 to -2.4) | 0.8 (0.7 to 1.0) | -0.1 (-0.3 to -0.0) | -1.0 (-1.0 to -0.9) | -1.2 (-1.3 to -1.1) | -1.3 (-1.4 to -1.2) | 0.2 (0.0 to 0.3) | 1.8 (1.6 to 2.0) | -0.4 (-0.5 to -0.3) | -0.6 (-0.6 to -0.4) | -0.7 (-0.8 to -0.6) | 2.3 (2.1 to 2.6) | 1.8 (1.5 to 2.0) | -1.9 (-2.1 to -1.7) | -1.1 (-1.3 to -0.9) | -2.0 (-2.2 to -1.8) | 1.8 (1.5 to 2.1) |
| Gabon | 1.7 (1.6 to 1.9) | 0.9 (0.8 to 1.0) | 1.1 (1.0 to 1.2) | 1.1 (0.9 to 1.2) | 2.1 (1.9 to 2.3) | 1.0 (0.9 to 1.2) | 0.7 (0.5 to 0.8) | 0.8 (0.7 to 0.9) | 0.8 (0.7 to 0.9) | 1.0 (0.9 to 1.2) | 1.7 (1.6 to 1.8) | 1.2 (1.0 to 1.3) | 1.2 (1.1 to 1.3) | 1.2 (1.1 to 1.3) | 1.6 (1.5 to 1.8) | 1.9 (1.7 to 2.2) | 0.7 (0.6 to 0.8) | 0.7 (0.5 to 0.8) | 0.6 (0.5 to 0.8) | 2.8 (2.4 to 3.1) |
| Georgia | 4.5 (3.8 to 5.2) | 3.9 (3.1 to 4.6) | 3.8 (3.1 to 4.4) | 3.7 (3.1 to 4.4) | 4.4 (3.8 to 5.0) | 1.3 (0.8 to 1.7) | 1.1 (0.6 to 1.6) | 1.0 (0.5 to 1.4) | 0.9 (0.5 to 1.4) | 1.1 (0.8 to 1.4) | 5.4 (4.7 to 6.2) | 5.2 (4.5 to 5.9) | 4.9 (4.2 to 5.6) | 4.9 (4.2 to 5.6) | 5.3 (4.6 to 6.0) | 0.8 (0.3 to 1.2) | 0.1 (-0.5 to 0.7) | -0.2 (-0.8 to 0.3) | -0.3 (-0.8 to 0.3) | 1.0 (0.6 to 1.4) |
| Germany | 0.5 (0.2 to 0.8) | -1.6 (-1.7 to -1.4) | -1.3 (-1.4 to -1.2) | -1.6 (-1.7 to -1.4) | 0.6 (0.3 to 1.0) | -1.0 (-1.1 to -0.9) | -1.8 (-2.0 to -1.6) | -1.8 (-2.0 to -1.7) | -1.9 (-2.1 to -1.7) | -0.6 (-0.8 to -0.5) | -0.5 (-0.6 to -0.3) | -1.1 (-1.2 to -0.9) | -1.5 (-1.6 to -1.4) | -1.6 (-1.7 to -1.4) | -0.3 (-0.5 to -0.1) | 0.6 (0.4 to 0.8) | -1.9 (-2.3 to -1.5) | -1.5 (-1.9 to -1.2) | -2.1 (-2.5 to -1.7) | 0.6 (0.3 to 0.8) |
| Ghana | -0.1 (-0.5 to 0.3) | -0.6 (-1.0 to -0.2) | -0.5 (-0.9 to -0.1) | -0.5 (-0.9 to -0.1) | 0.4 (0.0 to 0.8) | -0.3 (-0.7 to 0.2) | -0.6 (-0.9 to -0.2) | -0.4 (-0.8 to 0.0) | -0.4 (-0.8 to 0.0) | -0.1 (-0.5 to 0.2) | 2.3 (2.2 to 2.4) | 2.2 (2.1 to 2.3) | 1.8 (1.7 to 1.9) | 1.8 (1.7 to 1.9) | 2.2 (2.1 to 2.3) | 0.4 (-0.0 to 0.9) | -0.5 (-0.9 to -0.1) | -0.5 (-0.9 to -0.1) | -0.5 (-0.9 to -0.1) | 1.0 (0.6 to 1.5) |
| Greece | -0.6 (-1.0 to -0.3) | -1.3 (-1.6 to -1.0) | -1.4 (-1.7 to -1.2) | -1.5 (-1.8 to -1.3) | -0.6 (-0.9 to -0.2) | -0.6 (-0.7 to -0.4) | -1.0 (-1.2 to -0.8) | -1.0 (-1.1 to -0.8) | -1.0 (-1.2 to -0.8) | -0.3 (-0.5 to -0.2) | 0.6 (0.3 to 0.8) | -0.1 (-0.2 to 0.1) | -0.1 (-0.2 to 0.0) | -0.1 (-0.3 to 0.0) | 0.8 (0.6 to 1.1) | 0.2 (-0.3 to 0.7) | -0.9 (-1.1 to -0.8) | -0.8 (-1.0 to -0.5) | -1.0 (-1.2 to -0.9) | 0.2 (-0.3 to 0.7) |
| Greenland | 0.2 (0.0 to 0.4) | -0.6 (-0.8 to -0.5) | -0.7 (-0.9 to -0.6) | -0.8 (-0.9 to -0.6) | 0.4 (0.2 to 0.6) | -0.8 (-0.9 to -0.6) | -1.2 (-1.3 to -1.0) | -1.3 (-1.5 to -1.2) | -1.4 (-1.5 to -1.2) | -0.6 (-0.7 to -0.4) | 0.5 (0.3 to 0.7) | -0.3 (-0.5 to -0.2) | -0.6 (-0.8 to -0.4) | -0.6 (-0.8 to -0.5) | 0.9 (0.7 to 1.1) | -0.5 (-1.0 to 0.0) | -3.0 (-3.5 to -2.5) | -3.0 (-3.5 to -2.4) | -3.1 (-3.7 to -2.5) | 0.1 (-0.3 to 0.6) |
| Grenada | 0.5 (0.2 to 0.9) | -0.1 (-0.5 to 0.3) | -0.0 (-0.4 to 0.3) | -0.1 (-0.4 to 0.3) | 1.0 (0.6 to 1.3) | -0.1 (-0.3 to 0.0) | -0.5 (-0.7 to -0.4) | -0.4 (-0.6 to -0.3) | -0.4 (-0.6 to -0.3) | 0.4 (0.2 to 0.6) | 0.7 (0.5 to 0.9) | 0.1 (-0.1 to 0.2) | 0.0 (-0.1 to 0.2) | 0.0 (-0.1 to 0.2) | 1.2 (0.9 to 1.4) | 6.4 (5.1 to 7.6) | 4.8 (3.6 to 5.9) | 5.0 (3.9 to 6.1) | 4.9 (3.8 to 6.0) | 7.1 (5.8 to 8.3) |
| Guam | -1.0 (-1.7 to -0.2) | -1.8 (-2.5 to -1.0) | -1.6 (-2.3 to -0.8) | -1.6 (-2.3 to -0.9) | -0.6 (-1.3 to 0.0) | 1.2 (0.2 to 2.2) | 0.3 (-0.7 to 1.3) | 0.8 (-0.1 to 1.8) | 0.8 (-0.2 to 1.7) | 1.6 (0.8 to 2.5) | 0.1 (-1.7 to 1.8) | -0.4 (-2.1 to 1.4) | 0.1 (-1.6 to 1.9) | 0.1 (-1.6 to 1.9) | 0.3 (-1.4 to 2.0) | 5.1 (2.7 to 7.5) | 3.3 (0.9 to 5.7) | 4.3 (1.8 to 6.7) | 4.2 (1.8 to 6.6) | 5.4 (3.0 to 7.8) |
| Guatemala | 2.0 (1.3 to 2.7) | 1.2 (0.6 to 1.8) | 1.0 (0.4 to 1.6) | 0.9 (0.3 to 1.5) | 2.4 (1.8 to 3.0) | -0.6 (-0.8 to -0.4) | -1.1 (-1.3 to -0.8) | -1.1 (-1.4 to -0.9) | -1.2 (-1.4 to -0.9) | 0.0 (-0.1 to 0.2) | 1.7 (1.4 to 1.9) | 1.0 (0.8 to 1.3) | 0.7 (0.5 to 1.0) | 0.7 (0.5 to 0.9) | 2.2 (2.0 to 2.4) | 4.0 (3.4 to 4.5) | 1.8 (1.5 to 2.1) | 1.9 (1.6 to 2.3) | 1.8 (1.5 to 2.2) | 5.4 (4.7 to 6.2) |
| Guinea | 0.3 (0.2 to 0.5) | 0.0 (-0.1 to 0.2) | 0.0 (-0.1 to 0.2) | 0.0 (-0.1 to 0.1) | 0.8 (0.6 to 0.9) | -1.1 (-1.5 to -0.8) | -1.2 (-1.5 to -0.9) | -1.2 (-1.6 to -0.9) | -1.2 (-1.6 to -0.9) | -0.9 (-1.2 to -0.6) | 1.0 (0.9 to 1.1) | 0.9 (0.8 to 0.9) | 0.6 (0.5 to 0.7) | 0.6 (0.5 to 0.7) | 1.0 (0.9 to 1.2) | 0.5 (0.3 to 0.6) | -0.2 (-0.4 to -0.1) | -0.3 (-0.4 to -0.1) | -0.3 (-0.4 to -0.2) | 1.0 (0.7 to 1.2) |
| Guyana | 0.7 (0.4 to 1.0) | 0.5 (0.3 to 0.8) | 0.5 (0.2 to 0.8) | 0.5 (0.2 to 0.7) | 0.8 (0.4 to 1.0) | 0.6 (0.3 to 0.8) | 0.4 (0.1 to 0.6) | 0.4 (0.2 to 0.6) | 0.4 (0.1 to 0.6) | 0.7 (0.4 to 0.9) | 0.8 (0.5 to 1.2) | 0.3 (-0.1 to 0.7) | 0.4 (0.1 to 0.8) | 0.4 (0.1 to 0.8) | 1.1 (0.7 to 1.4) | 6.3 (5.0 to 7.7) | 5.3 (4.1 to 6.6) | 5.5 (4.2 to 6.8) | 5.5 (4.2 to 6.8) | 6.8 (5.4 to 8.2) |
| Haiti | 1.2 (1.1 to 1.3) | 0.9 (0.8 to 1.0) | 0.9 (0.8 to 1.0) | 0.9 (0.8 to 1.0) | 1.3 (1.1 to 1.4) | 0.8 (0.8 to 0.9) | 0.6 (0.5 to 0.7) | 0.6 (0.6 to 0.8) | 0.6 (0.6 to 0.7) | 0.9 (0.8 to 1.0) | 0.8 (0.7 to 0.9) | 0.4 (0.3 to 0.6) | 0.3 (0.2 to 0.4) | 0.3 (0.1 to 0.4) | 0.8 (0.7 to 0.9) | 3.1 (3.0 to 3.2) | 2.4 (2.2 to 2.5) | 2.2 (2.1 to 2.4) | 2.2 (2.1 to 2.4) | 3.3 (3.1 to 3.5) |
| Honduras | 0.7 (0.6 to 0.9) | 0.7 (0.5 to 0.8) | 0.3 (0.2 to 0.4) | 0.3 (0.2 to 0.4) | 0.6 (0.5 to 0.8) | 0.3 (0.2 to 0.4) | 0.4 (0.2 to 0.5) | -0.0 (-0.1 to 0.1) | -0.0 (-0.1 to 0.1) | 0.0 (-0.1 to 0.1) | 1.0 (0.8 to 1.2) | 0.7 (0.5 to 1.0) | 0.2 (0.0 to 0.5) | 0.2 (0.0 to 0.4) | 1.0 (0.8 to 1.2) | 1.8 (1.7 to 2.0) | 0.8 (0.6 to 0.9) | 0.7 (0.5 to 0.8) | 0.7 (0.5 to 0.8) | 1.9 (1.9 to 2.0) |
| Hungary | 0.8 (0.5 to 1.0) | -1.0 (-1.2 to -0.7) | -0.8 (-1.0 to -0.6) | -0.9 (-1.1 to -0.7) | 1.2 (0.9 to 1.5) | 0.3 (0.1 to 0.4) | -0.4 (-0.5 to -0.3) | -0.4 (-0.5 to -0.3) | -0.4 (-0.5 to -0.3) | 0.8 (0.6 to 0.9) | 0.4 (0.1 to 0.8) | -0.3 (-0.5 to -0.1) | -0.5 (-0.7 to -0.2) | -0.5 (-0.8 to -0.3) | 0.8 (0.4 to 1.2) | 0.9 (0.6 to 1.2) | -1.6 (-1.8 to -1.3) | -1.6 (-1.8 to -1.4) | -1.9 (-2.1 to -1.6) | 1.2 (0.9 to 1.6) |
| Iceland | -0.2 (-0.4 to 0.0) | -1.2 (-1.3 to -1.0) | -1.4 (-1.5 to -1.2) | -1.5 (-1.7 to -1.4) | -0.3 (-0.5 to -0.0) | -0.4 (-0.6 to -0.2) | -1.0 (-1.1 to -0.9) | -1.2 (-1.4 to -1.1) | -1.3 (-1.4 to -1.2) | -0.2 (-0.4 to -0.0) | 0.7 (0.5 to 0.9) | -0.4 (-0.6 to -0.2) | -0.5 (-0.6 to -0.4) | -0.6 (-0.7 to -0.4) | 1.1 (0.8 to 1.3) | 1.4 (1.2 to 1.6) | -1.0 (-1.2 to -0.8) | -0.4 (-0.6 to -0.3) | -1.1 (-1.4 to -0.9) | 1.4 (1.2 to 1.6) |
| India | 0.9 (0.7 to 1.1) | 0.0 (-0.1 to 0.1) | -0.1 (-0.2 to 0.1) | -0.1 (-0.3 to 0.0) | 1.4 (1.1 to 1.6) | 0.2 (0.0 to 0.4) | -0.2 (-0.3 to -0.1) | -0.3 (-0.5 to -0.2) | -0.4 (-0.5 to -0.2) | 0.4 (0.2 to 0.6) | 1.6 (1.5 to 1.7) | 1.1 (1.0 to 1.2) | 0.9 (0.8 to 1.0) | 0.9 (0.7 to 1.0) | 1.8 (1.7 to 1.9) | 2.1 (1.9 to 2.3) | -0.1 (-0.3 to 0.1) | -0.0 (-0.2 to 0.2) | -0.1 (-0.3 to 0.1) | 3.4 (3.2 to 3.6) |
| Indonesia | 1.2 (1.1 to 1.4) | 0.4 (0.3 to 0.5) | 0.6 (0.5 to 0.7) | 0.6 (0.4 to 0.7) | 1.8 (1.6 to 1.9) | 0.9 (0.8 to 1.0) | -0.0 (-0.1 to 0.1) | 0.3 (0.2 to 0.4) | 0.3 (0.2 to 0.4) | 1.2 (1.1 to 1.3) | 1.6 (1.6 to 1.7) | 1.4 (1.3 to 1.5) | 1.3 (1.2 to 1.4) | 1.3 (1.2 to 1.4) | 1.7 (1.6 to 1.8) | 3.3 (3.1 to 3.5) | 1.8 (1.6 to 1.9) | 2.2 (1.9 to 2.4) | 2.1 (1.9 to 2.4) | 4.0 (3.9 to 4.2) |
| Iran (Islamic Republic of) | 1.7 (1.6 to 1.9) | 0.2 (0.1 to 0.3) | 0.0 (-0.1 to 0.2) | -0.1 (-0.2 to 0.0) | 2.2 (2.0 to 2.3) | 0.5 (0.4 to 0.6) | -0.1 (-0.2 to 0.0) | -0.2 (-0.3 to -0.1) | -0.3 (-0.4 to -0.2) | 0.8 (0.7 to 0.8) | 1.2 (1.1 to 1.3) | 0.2 (0.1 to 0.4) | -0.0 (-0.1 to 0.1) | -0.1 (-0.2 to 0.0) | 1.6 (1.5 to 1.8) | 3.7 (3.5 to 4.0) | 0.1 (0.0 to 0.3) | 0.6 (0.4 to 0.8) | 0.3 (0.1 to 0.4) | 4.3 (4.1 to 4.5) |
| Iraq | 3.1 (2.9 to 3.3) | 1.6 (1.4 to 1.7) | 1.5 (1.4 to 1.7) | 1.4 (1.3 to 1.6) | 3.5 (3.4 to 3.7) | 1.9 (1.8 to 2.1) | 1.4 (1.2 to 1.5) | 1.1 (1.0 to 1.2) | 1.1 (1.0 to 1.2) | 1.9 (1.8 to 2.1) | 2.1 (2.0 to 2.2) | 1.1 (1.0 to 1.3) | 0.9 (0.8 to 1.0) | 0.9 (0.8 to 0.9) | 2.3 (2.2 to 2.4) | 4.9 (4.7 to 5.1) | 1.3 (1.2 to 1.4) | 1.7 (1.6 to 1.8) | 1.5 (1.4 to 1.6) | 5.8 (5.6 to 5.9) |
| Ireland | 0.3 (0.0 to 0.5) | -1.8 (-2.1 to -1.6) | -2.0 (-2.2 to -1.7) | -2.2 (-2.5 to -2.0) | 0.3 (0.1 to 0.6) | -0.3 (-0.4 to -0.1) | -1.2 (-1.4 to -1.1) | -1.5 (-1.6 to -1.4) | -1.6 (-1.7 to -1.4) | 0.2 (0.1 to 0.3) | 1.0 (0.8 to 1.3) | -0.4 (-0.7 to -0.2) | -0.6 (-0.9 to -0.4) | -0.7 (-1.0 to -0.5) | 1.6 (1.3 to 1.9) | 1.8 (1.4 to 2.3) | -2.1 (-2.3 to -2.0) | -1.4 (-1.6 to -1.1) | -2.3 (-2.5 to -2.1) | 2.0 (1.5 to 2.4) |
| Israel | -0.0 (-0.3 to 0.2) | -2.0 (-2.3 to -1.7) | -1.9 (-2.2 to -1.7) | -2.2 (-2.4 to -1.9) | 0.2 (-0.1 to 0.4) | 0.1 (-0.0 to 0.3) | -0.7 (-0.8 to -0.5) | -0.8 (-1.0 to -0.7) | -0.9 (-1.1 to -0.8) | 0.5 (0.3 to 0.6) | 0.9 (0.7 to 1.1) | -0.2 (-0.4 to -0.0) | -0.4 (-0.6 to -0.2) | -0.4 (-0.6 to -0.3) | 1.4 (1.2 to 1.6) | 1.7 (1.4 to 2.0) | -1.6 (-1.7 to -1.4) | -1.1 (-1.3 to -0.9) | -1.7 (-1.9 to -1.5) | 1.8 (1.5 to 2.1) |
| Italy | -0.2 (-0.6 to 0.3) | -1.4 (-1.6 to -1.1) | -1.5 (-1.7 to -1.3) | -1.7 (-1.9 to -1.5) | -0.2 (-0.6 to 0.2) | -1.2 (-1.4 to -1.0) | -1.5 (-1.5 to -1.4) | -1.9 (-2.0 to -1.8) | -2.0 (-2.1 to -1.9) | -1.1 (-1.3 to -0.8) | 0.3 (0.0 to 0.6) | -0.4 (-0.5 to -0.3) | -0.8 (-0.9 to -0.7) | -0.9 (-1.0 to -0.8) | 0.5 (0.2 to 0.8) | 2.3 (1.9 to 2.7) | -0.0 (-0.3 to 0.3) | 0.4 (0.1 to 0.6) | -0.2 (-0.4 to 0.1) | 2.3 (1.9 to 2.7) |
| Jamaica | 2.0 (1.5 to 2.6) | 1.6 (1.1 to 2.0) | 1.6 (1.1 to 2.1) | 1.5 (1.0 to 2.0) | 2.0 (1.5 to 2.6) | 1.1 (1.0 to 1.3) | 0.6 (0.5 to 0.8) | 0.9 (0.7 to 1.1) | 0.9 (0.7 to 1.0) | 1.2 (1.1 to 1.4) | 1.0 (0.8 to 1.3) | 0.5 (0.2 to 0.7) | 0.4 (0.0 to 0.7) | 0.3 (0.0 to 0.7) | 1.0 (0.7 to 1.2) | 8.6 (7.2 to 9.9) | 6.5 (5.3 to 7.6) | 7.2 (5.9 to 8.4) | 7.1 (5.9 to 8.4) | 7.8 (6.4 to 9.2) |
| Japan | 2.2 (1.7 to 2.7) | 0.0 (-0.2 to 0.3) | 0.0 (-0.2 to 0.3) | -0.3 (-0.5 to -0.0) | 2.2 (1.8 to 2.7) | 0.3 (0.2 to 0.4) | -0.1 (-0.2 to -0.0) | -0.3 (-0.4 to -0.3) | -0.4 (-0.5 to -0.3) | 0.4 (0.4 to 0.5) | 1.2 (0.9 to 1.4) | 0.4 (0.2 to 0.5) | -0.1 (-0.3 to 0.0) | -0.2 (-0.3 to -0.1) | 1.4 (1.1 to 1.7) | 0.2 (-0.3 to 0.7) | -2.2 (-2.5 to -1.9) | -2.0 (-2.3 to -1.6) | -2.6 (-2.9 to -2.3) | 0.2 (-0.3 to 0.7) |
| Jordan | 1.8 (1.7 to 1.9) | -0.2 (-0.3 to -0.2) | -0.2 (-0.3 to -0.2) | -0.4 (-0.4 to -0.3) | 2.1 (2.0 to 2.3) | 0.4 (0.3 to 0.5) | -0.4 (-0.4 to -0.3) | -0.5 (-0.6 to -0.5) | -0.6 (-0.6 to -0.5) | 0.5 (0.3 to 0.6) | 0.6 (0.3 to 0.8) | -0.6 (-0.8 to -0.4) | -0.8 (-1.1 to -0.6) | -0.9 (-1.1 to -0.6) | 1.0 (0.6 to 1.4) | 2.2 (1.8 to 2.6) | -1.2 (-1.4 to -1.1) | -1.3 (-1.6 to -1.1) | -1.6 (-1.8 to -1.4) | 2.8 (2.4 to 3.2) |
| Kazakhstan | 2.0 (1.6 to 2.4) | 0.9 (0.5 to 1.2) | 0.6 (0.3 to 0.9) | 0.5 (0.2 to 0.8) | 2.3 (1.9 to 2.7) | -1.2 (-1.4 to -0.9) | -1.6 (-1.9 to -1.3) | -2.0 (-2.3 to -1.6) | -2.0 (-2.4 to -1.7) | -0.8 (-1.0 to -0.6) | -0.4 (-0.7 to 0.0) | -1.0 (-1.5 to -0.6) | -1.4 (-1.9 to -0.9) | -1.4 (-1.9 to -1.0) | 0.1 (-0.3 to 0.4) | 2.3 (1.9 to 2.7) | -0.1 (-0.5 to 0.2) | -0.3 (-0.6 to 0.1) | -0.4 (-0.8 to -0.1) | 3.8 (3.1 to 4.3) |
| Kenya | 0.7 (0.4 to 0.9) | 0.5 (0.3 to 0.8) | 0.3 (0.1 to 0.5) | 0.3 (0.1 to 0.5) | 0.6 (0.4 to 0.9) | -0.5 (-0.8 to -0.2) | -0.4 (-0.7 to -0.1) | -0.5 (-0.8 to -0.3) | -0.5 (-0.8 to -0.3) | -0.8 (-1.0 to -0.5) | 1.5 (1.4 to 1.6) | 1.3 (1.1 to 1.4) | 1.2 (1.1 to 1.4) | 1.2 (1.1 to 1.4) | 1.5 (1.4 to 1.6) | 1.5 (1.3 to 1.6) | 1.2 (1.1 to 1.4) | 1.2 (1.0 to 1.4) | 1.2 (1.0 to 1.4) | 1.4 (1.1 to 1.6) |
| Kiribati | 0.3 (0.2 to 0.4) | 0.1 (0.0 to 0.2) | 0.1 (0.0 to 0.2) | 0.1 (0.0 to 0.2) | 0.4 (0.3 to 0.5) | 0.3 (0.1 to 0.4) | -0.1 (-0.3 to 0.1) | 0.0 (-0.2 to 0.2) | -0.0 (-0.2 to 0.2) | 0.2 (0.1 to 0.4) | 0.5 (0.3 to 0.6) | 0.5 (0.3 to 0.7) | 0.4 (0.3 to 0.6) | 0.4 (0.3 to 0.6) | 0.3 (0.2 to 0.5) | 1.3 (1.2 to 1.4) | 1.1 (0.9 to 1.2) | 1.0 (1.0 to 1.1) | 1.0 (1.0 to 1.1) | 1.6 (1.3 to 1.8) |
| Kuwait | 1.1 (0.6 to 1.5) | -0.4 (-0.8 to -0.0) | -0.6 (-1.1 to -0.2) | -0.8 (-1.2 to -0.4) | 1.2 (0.8 to 1.7) | -1.8 (-1.9 to -1.6) | -2.1 (-2.3 to -1.9) | -2.4 (-2.6 to -2.2) | -2.5 (-2.7 to -2.3) | -1.5 (-1.6 to -1.3) | 0.6 (0.2 to 1.0) | -0.6 (-1.0 to -0.2) | -0.8 (-1.1 to -0.3) | -0.8 (-1.2 to -0.4) | 1.0 (0.6 to 1.4) | 0.3 (-1.9 to 2.4) | -3.0 (-5.1 to -1.0) | -2.6 (-4.7 to -0.4) | -3.1 (-5.2 to -1.0) | 0.4 (-1.7 to 2.6) |
| Kyrgyzstan | 0.3 (-0.0 to 0.6) | -0.6 (-0.9 to -0.2) | -0.8 (-1.0 to -0.5) | -0.8 (-1.1 to -0.6) | 0.6 (0.4 to 0.9) | -1.2 (-1.7 to -0.7) | -1.6 (-2.1 to -1.1) | -1.8 (-2.4 to -1.2) | -1.8 (-2.4 to -1.3) | -0.8 (-1.2 to -0.3) | 1.7 (0.8 to 2.5) | 1.2 (0.4 to 2.0) | 0.8 (0.0 to 1.7) | 0.8 (-0.0 to 1.7) | 1.9 (1.1 to 2.8) | 0.4 (0.1 to 0.8) | -1.1 (-1.6 to -0.7) | -1.5 (-2.0 to -1.0) | -1.6 (-2.1 to -1.1) | 1.6 (1.2 to 1.9) |
| Lao People's Democratic Republic | 1.8 (1.7 to 1.8) | 1.0 (1.0 to 1.1) | 1.1 (1.0 to 1.1) | 1.0 (1.0 to 1.1) | 2.2 (2.0 to 2.3) | 1.4 (1.2 to 1.7) | 0.7 (0.4 to 0.9) | 0.8 (0.6 to 1.0) | 0.8 (0.5 to 1.0) | 1.7 (1.5 to 2.0) | 1.3 (1.2 to 1.4) | 1.0 (0.9 to 1.0) | 0.7 (0.7 to 0.8) | 0.7 (0.6 to 0.8) | 1.3 (1.2 to 1.5) | 2.2 (1.9 to 2.5) | 0.9 (0.7 to 1.0) | 0.7 (0.5 to 0.9) | 0.7 (0.4 to 0.9) | 2.5 (2.1 to 2.9) |
| Latvia | 2.6 (2.2 to 2.9) | 1.2 (0.9 to 1.5) | 1.1 (0.8 to 1.4) | 1.0 (0.7 to 1.3) | 2.6 (2.3 to 3.0) | 0.6 (0.4 to 0.8) | 0.1 (-0.1 to 0.3) | -0.1 (-0.4 to 0.0) | -0.2 (-0.4 to 0.0) | 0.9 (0.8 to 1.1) | 1.3 (0.9 to 1.6) | 0.6 (0.2 to 1.0) | 0.2 (-0.2 to 0.6) | 0.2 (-0.2 to 0.6) | 1.5 (1.1 to 1.9) | 0.6 (0.4 to 0.8) | -1.8 (-2.0 to -1.5) | -2.0 (-2.3 to -1.7) | -2.2 (-2.5 to -1.9) | 1.2 (1.0 to 1.4) |
| Lebanon | 5.0 (4.6 to 5.4) | 2.6 (2.3 to 3.0) | 2.7 (2.4 to 3.1) | 2.5 (2.2 to 2.9) | 5.5 (5.2 to 5.9) | 3.4 (2.9 to 3.8) | 2.2 (1.8 to 2.6) | 2.4 (1.9 to 2.8) | 2.3 (1.8 to 2.7) | 3.7 (3.3 to 4.1) | 3.2 (2.9 to 3.5) | 1.7 (1.4 to 2.0) | 1.7 (1.4 to 2.1) | 1.7 (1.4 to 2.0) | 3.9 (3.6 to 4.2) | 5.9 (5.6 to 6.2) | 1.5 (1.2 to 1.9) | 1.8 (1.6 to 2.1) | 1.5 (1.2 to 1.8) | 6.6 (6.3 to 6.9) |
| Lesotho | -0.9 (-1.5 to -0.4) | -1.0 (-1.5 to -0.6) | -0.9 (-1.5 to -0.4) | -0.9 (-1.5 to -0.4) | -0.7 (-1.1 to -0.3) | -0.9 (-1.4 to -0.4) | -0.8 (-1.2 to -0.3) | -0.7 (-1.2 to -0.1) | -0.7 (-1.2 to -0.1) | -0.9 (-1.3 to -0.6) | 0.8 (0.6 to 1.2) | 0.8 (0.5 to 1.1) | 0.8 (0.5 to 1.2) | 0.8 (0.5 to 1.2) | 0.9 (0.7 to 1.1) | -0.7 (-1.2 to -0.2) | -0.6 (-1.1 to -0.2) | -0.6 (-1.1 to -0.1) | -0.6 (-1.1 to -0.1) | -0.5 (-0.8 to -0.2) |
| Libya | 0.8 (0.5 to 1.0) | -0.1 (-0.2 to 0.0) | -0.1 (-0.3 to -0.0) | -0.2 (-0.3 to -0.1) | 0.9 (0.6 to 1.3) | 0.1 (-0.1 to 0.2) | -0.1 (-0.2 to -0.1) | -0.2 (-0.3 to -0.1) | -0.2 (-0.3 to -0.1) | 0.1 (-0.1 to 0.3) | 1.0 (0.8 to 1.3) | 0.3 (0.1 to 0.5) | 0.3 (0.1 to 0.5) | 0.3 (0.1 to 0.5) | 1.3 (1.0 to 1.6) | 2.1 (1.8 to 2.4) | 0.3 (0.3 to 0.4) | 0.4 (0.3 to 0.4) | 0.2 (0.2 to 0.3) | 2.5 (2.2 to 2.9) |
| Lithuania | 2.0 (1.7 to 2.3) | 0.8 (0.6 to 1.1) | 0.8 (0.5 to 1.0) | 0.6 (0.3 to 0.9) | 2.0 (1.7 to 2.3) | 0.1 (-0.2 to 0.4) | -0.2 (-0.5 to 0.1) | -0.5 (-0.8 to -0.2) | -0.6 (-0.9 to -0.2) | 0.3 (-0.0 to 0.6) | 2.2 (1.5 to 2.8) | 1.8 (1.1 to 2.4) | 1.3 (0.6 to 1.9) | 1.2 (0.6 to 1.9) | 2.3 (1.6 to 2.9) | 0.3 (0.0 to 0.7) | -1.3 (-1.6 to -1.0) | -1.6 (-1.9 to -1.2) | -1.7 (-2.0 to -1.4) | 0.8 (0.4 to 1.2) |
| Luxembourg | -0.4 (-0.7 to -0.1) | -2.3 (-2.5 to -2.1) | -2.4 (-2.5 to -2.2) | -2.6 (-2.8 to -2.5) | -0.3 (-0.6 to 0.1) | -1.0 (-1.3 to -0.8) | -1.8 (-2.0 to -1.6) | -2.1 (-2.2 to -1.9) | -2.1 (-2.3 to -2.0) | -0.7 (-0.9 to -0.4) | 0.3 (-0.0 to 0.6) | -0.8 (-1.0 to -0.6) | -1.2 (-1.4 to -1.0) | -1.2 (-1.4 to -1.0) | 0.7 (0.4 to 1.1) | -0.8 (-1.1 to -0.5) | -3.4 (-3.5 to -3.3) | -3.4 (-3.5 to -3.3) | -4.1 (-4.3 to -4.0) | -0.8 (-1.1 to -0.4) |
| Malaysia | 1.5 (1.4 to 1.6) | 0.3 (0.2 to 0.5) | 0.4 (0.3 to 0.5) | 0.3 (0.2 to 0.4) | 2.0 (1.9 to 2.1) | 0.7 (0.6 to 0.8) | -0.2 (-0.3 to -0.2) | -0.1 (-0.1 to 0.0) | -0.1 (-0.2 to -0.0) | 1.2 (1.1 to 1.4) | 1.0 (0.9 to 1.1) | 0.4 (0.3 to 0.5) | 0.4 (0.3 to 0.5) | 0.4 (0.3 to 0.5) | 1.3 (1.1 to 1.4) | 3.5 (3.4 to 3.7) | 1.6 (1.4 to 1.7) | 1.6 (1.4 to 1.8) | 1.5 (1.3 to 1.7) | 4.6 (4.5 to 4.8) |
| Maldives | 4.4 (4.2 to 4.5) | 2.6 (2.5 to 2.7) | 2.3 (2.2 to 2.4) | 2.1 (2.0 to 2.2) | 4.7 (4.5 to 4.9) | 2.6 (2.5 to 2.7) | 1.8 (1.6 to 1.8) | 1.2 (1.1 to 1.3) | 1.1 (1.0 to 1.2) | 2.7 (2.5 to 2.8) | 1.6 (1.5 to 1.8) | 0.6 (0.5 to 0.7) | 0.0 (-0.0 to 0.1) | -0.0 (-0.1 to 0.1) | 2.0 (1.9 to 2.2) | 3.5 (3.3 to 3.7) | 0.8 (0.7 to 0.9) | -0.2 (-0.3 to -0.1) | -0.4 (-0.5 to -0.3) | 5.3 (5.0 to 5.6) |
| Malta | -0.4 (-0.7 to -0.1) | -2.2 (-2.5 to -2.0) | -2.2 (-2.4 to -2.0) | -2.5 (-2.7 to -2.2) | -0.3 (-0.6 to -0.0) | -1.1 (-1.3 to -1.0) | -1.9 (-2.0 to -1.7) | -2.0 (-2.1 to -1.8) | -2.0 (-2.2 to -1.9) | -0.7 (-0.9 to -0.6) | 0.8 (0.6 to 1.0) | -0.4 (-0.5 to -0.2) | -0.3 (-0.5 to -0.2) | -0.4 (-0.5 to -0.3) | 1.4 (1.2 to 1.6) | 1.0 (0.5 to 1.5) | -1.8 (-2.0 to -1.5) | -1.4 (-1.7 to -1.1) | -2.0 (-2.3 to -1.7) | 1.1 (0.6 to 1.6) |
| Marshall Islands | 0.5 (0.4 to 0.6) | 0.2 (0.1 to 0.2) | 0.2 (0.1 to 0.3) | 0.2 (0.1 to 0.3) | 0.5 (0.4 to 0.6) | 0.6 (0.4 to 0.7) | 0.2 (0.1 to 0.3) | 0.3 (0.2 to 0.4) | 0.3 (0.2 to 0.4) | 0.5 (0.4 to 0.6) | 0.4 (0.2 to 0.5) | 0.3 (0.2 to 0.4) | 0.3 (0.2 to 0.4) | 0.3 (0.2 to 0.4) | 0.2 (0.0 to 0.3) | 1.1 (1.0 to 1.3) | 0.8 (0.7 to 0.9) | 0.7 (0.6 to 0.8) | 0.7 (0.6 to 0.8) | 1.3 (1.0 to 1.6) |
| Mauritania | 2.6 (2.4 to 2.8) | 1.7 (1.5 to 1.9) | 1.8 (1.6 to 2.0) | 1.8 (1.6 to 2.0) | 2.9 (2.8 to 3.1) | 0.6 (0.4 to 0.7) | 0.1 (-0.0 to 0.3) | 0.2 (0.0 to 0.4) | 0.2 (0.0 to 0.3) | 0.6 (0.4 to 0.7) | 1.7 (1.6 to 1.8) | 1.2 (1.1 to 1.3) | 1.0 (1.0 to 1.1) | 1.0 (0.9 to 1.1) | 1.6 (1.5 to 1.7) | 0.6 (0.3 to 0.9) | -1.0 (-1.3 to -0.7) | -1.1 (-1.4 to -0.9) | -1.2 (-1.5 to -0.9) | 1.5 (1.1 to 2.0) |
| Mauritius | 1.5 (1.2 to 1.8) | 0.5 (0.3 to 0.8) | 0.6 (0.3 to 0.8) | 0.5 (0.3 to 0.8) | 1.7 (1.4 to 2.0) | -0.6 (-1.2 to 0.0) | -1.2 (-1.9 to -0.6) | -1.2 (-1.8 to -0.6) | -1.3 (-1.9 to -0.6) | -0.2 (-0.7 to 0.3) | 1.0 (0.3 to 1.7) | 0.7 (-0.0 to 1.5) | 0.7 (-0.0 to 1.4) | 0.7 (-0.1 to 1.4) | 1.2 (0.4 to 1.9) | 5.0 (4.2 to 5.8) | 3.2 (2.3 to 4.1) | 3.6 (2.7 to 4.5) | 3.5 (2.6 to 4.4) | 5.7 (4.9 to 6.5) |
| Mexico | 0.3 (0.0 to 0.6) | -0.7 (-0.8 to -0.5) | -0.6 (-0.8 to -0.5) | -0.7 (-0.9 to -0.6) | 0.6 (0.3 to 0.9) | -0.1 (-0.2 to 0.0) | -0.6 (-0.8 to -0.4) | -0.5 (-0.7 to -0.4) | -0.6 (-0.7 to -0.4) | 0.2 (0.1 to 0.3) | 2.0 (1.9 to 2.1) | 1.2 (1.1 to 1.3) | 1.2 (1.1 to 1.3) | 1.1 (1.0 to 1.2) | 2.4 (2.2 to 2.5) | 4.2 (4.0 to 4.3) | 1.8 (1.5 to 2.0) | 2.4 (2.1 to 2.6) | 2.2 (2.0 to 2.5) | 5.0 (4.9 to 5.2) |
| Micronesia (Federated States of) | -1.1 (-1.2 to -1.0) | -1.5 (-1.6 to -1.4) | -1.6 (-1.7 to -1.5) | -1.6 (-1.7 to -1.5) | -0.8 (-0.9 to -0.7) | -1.1 (-1.2 to -1.0) | -1.6 (-1.7 to -1.4) | -1.6 (-1.7 to -1.4) | -1.6 (-1.7 to -1.5) | -0.6 (-0.7 to -0.5) | 0.6 (0.5 to 0.7) | 0.6 (0.5 to 0.7) | 0.4 (0.3 to 0.5) | 0.4 (0.3 to 0.5) | 0.6 (0.5 to 0.8) | -0.2 (-0.3 to -0.1) | -0.9 (-1.0 to -0.8) | -0.8 (-0.9 to -0.7) | -0.8 (-0.9 to -0.7) | 0.4 (0.2 to 0.6) |
| Monaco | 1.2 (1.1 to 1.4) | 0.1 (0.0 to 0.3) | 0.0 (-0.1 to 0.1) | -0.1 (-0.2 to -0.0) | 0.9 (0.7 to 1.1) | 0.3 (0.2 to 0.4) | 0.0 (-0.1 to 0.2) | -0.3 (-0.4 to -0.2) | -0.3 (-0.4 to -0.2) | 0.0 (-0.0 to 0.1) | 2.0 (1.8 to 2.1) | 1.1 (1.0 to 1.2) | 1.0 (0.9 to 1.1) | 1.0 (0.9 to 1.1) | 1.9 (1.7 to 2.1) | 0.9 (0.8 to 1.0) | -1.1 (-1.3 to -0.9) | -0.7 (-0.8 to -0.6) | -1.2 (-1.3 to -1.0) | 0.8 (0.7 to 0.9) |
| Mongolia | 1.7 (1.6 to 1.8) | 0.8 (0.7 to 0.9) | 0.8 (0.6 to 0.9) | 0.7 (0.6 to 0.9) | 1.9 (1.8 to 2.1) | -2.0 (-2.4 to -1.7) | -2.6 (-3.0 to -2.3) | -2.5 (-2.9 to -2.2) | -2.6 (-2.9 to -2.2) | -1.4 (-1.7 to -1.1) | 0.7 (0.4 to 0.9) | -0.1 (-0.3 to 0.2) | 0.1 (-0.2 to 0.4) | 0.1 (-0.2 to 0.4) | 1.0 (0.7 to 1.2) | 1.3 (1.1 to 1.5) | -0.6 (-0.8 to -0.4) | -0.4 (-0.6 to -0.2) | -0.5 (-0.7 to -0.2) | 2.6 (2.4 to 2.7) |
| Montenegro | 1.0 (0.8 to 1.1) | -0.1 (-0.1 to -0.0) | -0.1 (-0.1 to -0.0) | -0.1 (-0.2 to -0.1) | 1.2 (1.1 to 1.4) | 0.3 (0.2 to 0.4) | -0.1 (-0.2 to -0.0) | -0.2 (-0.2 to -0.1) | -0.2 (-0.3 to -0.1) | 0.4 (0.3 to 0.5) | 0.7 (0.6 to 0.8) | 0.3 (0.2 to 0.3) | 0.0 (-0.1 to 0.1) | -0.0 (-0.1 to 0.1) | 0.8 (0.7 to 0.9) | 1.1 (0.9 to 1.4) | -0.6 (-0.6 to -0.5) | -0.5 (-0.6 to -0.4) | -0.7 (-0.8 to -0.6) | 1.2 (0.9 to 1.4) |
| Morocco | 1.8 (1.6 to 2.0) | 0.8 (0.6 to 1.0) | 0.8 (0.7 to 1.0) | 0.8 (0.6 to 1.0) | 1.9 (1.8 to 2.1) | 1.0 (0.7 to 1.2) | 0.5 (0.3 to 0.7) | 0.5 (0.3 to 0.7) | 0.5 (0.3 to 0.7) | 0.8 (0.6 to 0.9) | 1.3 (1.2 to 1.4) | 0.7 (0.6 to 0.8) | 0.5 (0.3 to 0.6) | 0.5 (0.3 to 0.6) | 1.1 (0.9 to 1.3) | 3.7 (3.3 to 4.0) | 1.0 (0.7 to 1.2) | 1.4 (1.1 to 1.6) | 1.3 (1.1 to 1.5) | 4.7 (4.4 to 5.0) |
| Myanmar | 1.7 (1.6 to 1.9) | 1.0 (0.9 to 1.1) | 1.0 (0.8 to 1.1) | 0.9 (0.8 to 1.1) | 2.0 (1.9 to 2.2) | 0.8 (0.5 to 1.1) | 0.1 (-0.1 to 0.4) | 0.1 (-0.2 to 0.3) | 0.0 (-0.2 to 0.3) | 1.0 (0.8 to 1.2) | 0.7 (0.6 to 0.8) | 0.1 (0.1 to 0.2) | 0.1 (0.1 to 0.2) | 0.1 (0.0 to 0.2) | 0.8 (0.7 to 0.9) | 1.5 (1.3 to 1.8) | 0.2 (0.1 to 0.4) | 0.0 (-0.1 to 0.2) | 0.0 (-0.1 to 0.1) | 2.0 (1.7 to 2.4) |
| Namibia | 1.6 (1.3 to 1.9) | 1.0 (0.8 to 1.3) | 1.0 (0.7 to 1.3) | 1.0 (0.7 to 1.2) | 1.9 (1.7 to 2.2) | -0.2 (-0.5 to 0.0) | -0.4 (-0.6 to -0.2) | -0.5 (-0.8 to -0.2) | -0.5 (-0.8 to -0.3) | -0.2 (-0.3 to -0.0) | 0.9 (0.8 to 1.0) | 0.4 (0.3 to 0.6) | 0.5 (0.4 to 0.6) | 0.5 (0.4 to 0.6) | 0.9 (0.7 to 1.1) | 0.1 (-0.3 to 0.4) | -1.1 (-1.3 to -0.8) | -1.1 (-1.4 to -0.9) | -1.2 (-1.4 to -1.0) | 1.2 (1.0 to 1.4) |
| Nauru | 0.6 (0.4 to 0.7) | 0.2 (0.1 to 0.3) | 0.3 (0.2 to 0.4) | 0.2 (0.2 to 0.3) | 0.8 (0.7 to 1.0) | 0.6 (0.4 to 0.8) | 0.1 (-0.0 to 0.3) | 0.3 (0.1 to 0.5) | 0.3 (0.1 to 0.5) | 0.7 (0.6 to 0.9) | -0.1 (-0.2 to 0.0) | -0.1 (-0.2 to -0.1) | -0.1 (-0.2 to -0.0) | -0.1 (-0.2 to -0.0) | -0.1 (-0.3 to 0.0) | 0.9 (0.5 to 1.3) | 0.4 (0.2 to 0.6) | 0.4 (0.2 to 0.7) | 0.4 (0.2 to 0.7) | 1.2 (0.7 to 1.7) |
| Nepal | -0.1 (-0.4 to 0.2) | -0.8 (-1.0 to -0.5) | -0.8 (-1.1 to -0.5) | -0.9 (-1.2 to -0.6) | 0.5 (0.2 to 0.8) | -0.7 (-1.0 to -0.4) | -1.1 (-1.4 to -0.9) | -1.1 (-1.4 to -0.8) | -1.1 (-1.4 to -0.8) | -0.2 (-0.5 to 0.0) | 1.1 (1.0 to 1.2) | 0.6 (0.5 to 0.7) | 0.5 (0.4 to 0.6) | 0.5 (0.4 to 0.6) | 1.4 (1.2 to 1.5) | 3.5 (3.3 to 3.7) | 1.4 (1.2 to 1.6) | 1.7 (1.5 to 1.9) | 1.7 (1.4 to 1.9) | 4.2 (4.0 to 4.5) |
| Netherlands | 0.1 (-0.1 to 0.3) | -1.7 (-1.8 to -1.5) | -1.6 (-1.7 to -1.4) | -1.8 (-1.9 to -1.6) | 0.2 (-0.1 to 0.4) | -0.8 (-1.0 to -0.7) | -1.3 (-1.3 to -1.2) | -1.6 (-1.7 to -1.5) | -1.6 (-1.7 to -1.6) | -0.6 (-0.7 to -0.4) | 0.3 (0.1 to 0.5) | -0.6 (-0.7 to -0.4) | -0.9 (-1.0 to -0.8) | -0.9 (-1.1 to -0.8) | 0.7 (0.5 to 1.0) | 1.7 (1.5 to 2.0) | -1.6 (-1.8 to -1.5) | -1.0 (-1.2 to -0.9) | -1.8 (-1.9 to -1.6) | 1.8 (1.6 to 2.1) |
| New Zealand | 0.4 (0.1 to 0.8) | -1.8 (-2.0 to -1.5) | -1.7 (-2.0 to -1.5) | -2.1 (-2.3 to -1.9) | 0.5 (0.1 to 0.9) | -1.0 (-1.2 to -0.7) | -1.0 (-1.2 to -0.8) | -1.2 (-1.5 to -1.0) | -1.3 (-1.5 to -1.0) | -0.9 (-1.1 to -0.6) | 0.9 (0.7 to 1.2) | -0.3 (-0.5 to -0.1) | -0.4 (-0.6 to -0.2) | -0.5 (-0.7 to -0.3) | 1.2 (0.9 to 1.4) | 0.3 (-0.5 to 1.2) | -2.6 (-3.4 to -1.8) | -2.1 (-2.9 to -1.3) | -2.8 (-3.6 to -2.0) | 0.4 (-0.4 to 1.2) |
| Nicaragua | 1.9 (1.7 to 2.1) | 0.6 (0.4 to 0.8) | 0.7 (0.5 to 0.9) | 0.6 (0.4 to 0.8) | 2.3 (2.1 to 2.5) | 1.4 (1.2 to 1.7) | 0.8 (0.6 to 1.1) | 0.9 (0.7 to 1.1) | 0.8 (0.6 to 1.1) | 1.6 (1.4 to 1.8) | 2.3 (2.1 to 2.4) | 1.4 (1.3 to 1.6) | 1.2 (1.1 to 1.4) | 1.2 (1.1 to 1.3) | 2.6 (2.5 to 2.8) | 3.7 (3.5 to 3.8) | 1.8 (1.6 to 1.9) | 1.6 (1.4 to 1.8) | 1.5 (1.3 to 1.7) | 4.4 (4.2 to 4.5) |
| Nigeria | 1.5 (1.4 to 1.5) | 0.8 (0.8 to 0.9) | 0.8 (0.8 to 0.9) | 0.8 (0.8 to 0.9) | 1.6 (1.5 to 1.6) | 0.7 (0.6 to 0.8) | 0.5 (0.5 to 0.6) | 0.4 (0.3 to 0.5) | 0.4 (0.3 to 0.5) | 0.3 (0.2 to 0.5) | 2.4 (2.3 to 2.6) | 2.1 (1.9 to 2.2) | 1.9 (1.7 to 2.0) | 1.9 (1.7 to 2.0) | 2.3 (2.1 to 2.5) | -0.8 (-1.2 to -0.5) | -2.1 (-2.5 to -1.7) | -2.1 (-2.5 to -1.8) | -2.2 (-2.5 to -1.9) | -0.1 (-0.5 to 0.3) |
| North Macedonia | 1.4 (1.3 to 1.5) | 0.6 (0.6 to 0.7) | 0.7 (0.6 to 0.8) | 0.6 (0.6 to 0.7) | 1.7 (1.6 to 1.9) | 0.8 (0.7 to 0.9) | 0.1 (0.1 to 0.2) | 0.1 (0.0 to 0.2) | 0.1 (-0.0 to 0.2) | 1.1 (1.0 to 1.2) | 1.8 (1.6 to 2.0) | 1.0 (0.9 to 1.2) | 0.9 (0.8 to 1.1) | 0.9 (0.7 to 1.1) | 2.1 (1.9 to 2.3) | 2.7 (2.5 to 2.8) | -0.1 (-0.2 to 0.0) | 0.1 (-0.0 to 0.2) | -0.1 (-0.3 to -0.0) | 3.2 (3.1 to 3.4) |
| Northern Mariana Islands | -0.1 (-0.5 to 0.2) | -0.4 (-0.7 to -0.1) | -0.5 (-0.8 to -0.2) | -0.5 (-0.8 to -0.2) | -0.1 (-0.5 to 0.3) | 1.8 (1.3 to 2.3) | 1.5 (1.0 to 2.0) | 1.5 (1.0 to 2.0) | 1.5 (1.0 to 2.0) | 1.8 (1.4 to 2.2) | 0.0 (-0.1 to 0.1) | 0.1 (0.1 to 0.2) | 0.1 (0.0 to 0.1) | 0.1 (0.0 to 0.1) | -0.1 (-0.2 to -0.0) | 4.5 (4.1 to 4.9) | 4.0 (3.4 to 4.5) | 4.1 (3.6 to 4.6) | 4.1 (3.6 to 4.6) | 4.5 (4.1 to 5.0) |
| Norway | 0.1 (-0.4 to 0.5) | -1.7 (-1.9 to -1.5) | -1.9 (-2.2 to -1.6) | -2.1 (-2.4 to -1.9) | 0.1 (-0.3 to 0.6) | -1.0 (-1.3 to -0.6) | -1.6 (-1.8 to -1.4) | -1.9 (-2.1 to -1.7) | -2.0 (-2.2 to -1.8) | -0.6 (-1.0 to -0.3) | -0.1 (-0.5 to 0.2) | -1.3 (-1.5 to -1.1) | -1.6 (-1.8 to -1.3) | -1.6 (-1.9 to -1.4) | 0.3 (-0.0 to 0.7) | -0.6 (-1.9 to 0.7) | -3.0 (-4.0 to -1.9) | -2.5 (-3.6 to -1.4) | -3.2 (-4.2 to -2.1) | -0.6 (-2.0 to 0.7) |
| Oman | 1.8 (1.6 to 1.9) | 0.9 (0.7 to 1.1) | 0.4 (0.3 to 0.6) | 0.3 (0.2 to 0.5) | 2.1 (2.0 to 2.3) | 0.4 (0.3 to 0.6) | 0.3 (0.1 to 0.5) | -0.3 (-0.5 to -0.1) | -0.3 (-0.5 to -0.2) | 0.6 (0.4 to 0.7) | 1.6 (1.4 to 1.7) | 1.0 (0.8 to 1.1) | 0.4 (0.2 to 0.5) | 0.3 (0.2 to 0.5) | 2.0 (1.8 to 2.2) | 2.2 (1.8 to 2.5) | -0.0 (-0.0 to 0.0) | -0.5 (-0.7 to -0.4) | -0.7 (-0.9 to -0.6) | 3.2 (2.6 to 3.9) |
| Pakistan | 1.1 (1.0 to 1.3) | 0.5 (0.4 to 0.6) | 0.6 (0.5 to 0.7) | 0.6 (0.4 to 0.7) | 1.5 (1.4 to 1.6) | 0.0 (-0.2 to 0.2) | -0.3 (-0.4 to -0.1) | -0.2 (-0.3 to -0.1) | -0.2 (-0.3 to -0.1) | 0.1 (-0.0 to 0.2) | 1.4 (1.2 to 1.5) | 0.9 (0.8 to 1.0) | 0.9 (0.8 to 1.1) | 0.9 (0.8 to 1.1) | 1.5 (1.4 to 1.6) | 1.7 (1.5 to 1.8) | 0.4 (0.4 to 0.5) | 0.3 (0.3 to 0.4) | 0.3 (0.3 to 0.4) | 2.4 (2.2 to 2.6) |
| Palau | -0.1 (-0.4 to 0.1) | -0.4 (-0.7 to -0.2) | -0.5 (-0.8 to -0.3) | -0.5 (-0.8 to -0.3) | -0.0 (-0.3 to 0.2) | -0.1 (-0.2 to 0.1) | -0.5 (-0.6 to -0.3) | -0.4 (-0.6 to -0.3) | -0.4 (-0.6 to -0.3) | 0.0 (-0.1 to 0.2) | 0.0 (-0.2 to 0.3) | 0.1 (-0.2 to 0.3) | -0.1 (-0.3 to 0.1) | -0.1 (-0.3 to 0.1) | -0.1 (-0.2 to 0.1) | 0.7 (0.6 to 0.8) | 0.2 (-0.0 to 0.3) | 0.2 (0.1 to 0.2) | 0.2 (0.1 to 0.2) | 1.3 (1.1 to 1.5) |
| Palestine | 1.9 (1.8 to 2.0) | 0.4 (0.3 to 0.4) | 0.4 (0.3 to 0.4) | 0.3 (0.2 to 0.3) | 2.5 (2.4 to 2.6) | 0.5 (0.4 to 0.7) | 0.0 (-0.1 to 0.1) | -0.1 (-0.2 to -0.0) | -0.2 (-0.3 to -0.1) | 0.8 (0.7 to 1.0) | 1.0 (0.9 to 1.1) | 0.0 (-0.1 to 0.2) | -0.1 (-0.2 to 0.0) | -0.1 (-0.3 to -0.0) | 1.6 (1.5 to 1.6) | -0.2 (-0.4 to 0.1) | -2.3 (-2.5 to -2.1) | -3.1 (-3.3 to -3.0) | -3.3 (-3.5 to -3.2) | 0.8 (0.6 to 1.0) |
| Panama | 1.0 (0.6 to 1.4) | -0.4 (-0.7 to -0.0) | -0.3 (-0.6 to 0.0) | -0.4 (-0.7 to -0.1) | 1.1 (0.8 to 1.5) | 0.4 (0.1 to 0.7) | -0.2 (-0.5 to 0.1) | -0.2 (-0.4 to 0.1) | -0.2 (-0.5 to 0.1) | 0.7 (0.4 to 0.9) | 2.6 (2.5 to 2.8) | 1.5 (1.4 to 1.7) | 1.6 (1.4 to 1.7) | 1.5 (1.4 to 1.7) | 3.0 (2.8 to 3.2) | 4.0 (3.8 to 4.2) | 1.5 (1.3 to 1.8) | 1.8 (1.6 to 2.1) | 1.6 (1.4 to 1.9) | 4.5 (4.3 to 4.7) |
| Papua New Guinea | -0.0 (-0.2 to 0.1) | -0.3 (-0.4 to -0.2) | -0.3 (-0.5 to -0.2) | -0.4 (-0.5 to -0.3) | 0.1 (-0.0 to 0.3) | -0.1 (-0.2 to 0.0) | -0.5 (-0.6 to -0.4) | -0.4 (-0.5 to -0.3) | -0.4 (-0.5 to -0.3) | 0.1 (0.0 to 0.2) | 0.2 (0.0 to 0.3) | 0.2 (0.1 to 0.2) | 0.1 (0.0 to 0.2) | 0.1 (0.0 to 0.2) | 0.1 (-0.0 to 0.3) | 1.4 (1.2 to 1.6) | 0.5 (0.4 to 0.6) | 1.0 (0.8 to 1.2) | 1.0 (0.8 to 1.2) | 1.9 (1.6 to 2.2) |
| Paraguay | 1.9 (1.6 to 2.1) | 1.4 (1.2 to 1.7) | 1.3 (1.1 to 1.6) | 1.3 (1.0 to 1.6) | 2.0 (1.7 to 2.3) | 1.7 (1.5 to 1.8) | 1.5 (1.3 to 1.7) | 1.4 (1.3 to 1.6) | 1.4 (1.3 to 1.6) | 1.6 (1.5 to 1.8) | 2.4 (2.4 to 2.5) | 2.0 (1.9 to 2.0) | 1.8 (1.8 to 1.9) | 1.8 (1.8 to 1.9) | 2.6 (2.5 to 2.7) | 4.4 (4.2 to 4.5) | 3.1 (2.9 to 3.3) | 3.3 (3.0 to 3.5) | 3.2 (3.0 to 3.4) | 5.1 (4.9 to 5.2) |
| Peru | 2.6 (2.2 to 3.0) | 0.6 (0.2 to 0.9) | 0.6 (0.2 to 0.9) | 0.4 (0.0 to 0.8) | 2.7 (2.3 to 3.1) | 1.3 (1.0 to 1.6) | 0.2 (-0.1 to 0.5) | 0.2 (-0.1 to 0.5) | 0.1 (-0.2 to 0.4) | 1.3 (1.0 to 1.6) | 2.1 (1.9 to 2.3) | 0.9 (0.7 to 1.1) | 0.7 (0.4 to 0.9) | 0.6 (0.4 to 0.9) | 2.1 (2.0 to 2.3) | 3.9 (3.5 to 4.3) | 0.7 (0.3 to 1.1) | 0.8 (0.4 to 1.2) | 0.6 (0.2 to 1.0) | 4.8 (4.3 to 5.3) |
| Philippines | 1.0 (0.8 to 1.1) | 0.6 (0.5 to 0.8) | 0.7 (0.5 to 0.8) | 0.7 (0.5 to 0.8) | 1.2 (1.0 to 1.4) | 0.3 (0.2 to 0.4) | 0.2 (0.1 to 0.4) | 0.1 (-0.1 to 0.2) | 0.1 (-0.1 to 0.2) | 0.2 (0.1 to 0.4) | 0.6 (0.5 to 0.7) | 0.8 (0.8 to 0.9) | 0.6 (0.5 to 0.6) | 0.6 (0.5 to 0.6) | 0.5 (0.3 to 0.6) | 0.7 (0.3 to 1.0) | 0.4 (0.3 to 0.6) | 0.3 (0.1 to 0.5) | 0.3 (0.1 to 0.5) | 0.9 (0.5 to 1.4) |
| Poland | 2.1 (1.7 to 2.5) | 0.8 (0.5 to 1.2) | 0.6 (0.3 to 1.0) | 0.6 (0.2 to 0.9) | 2.4 (2.0 to 2.8) | 0.7 (0.4 to 0.9) | 0.3 (0.1 to 0.5) | -0.1 (-0.2 to 0.1) | -0.1 (-0.3 to 0.1) | 0.9 (0.6 to 1.1) | -0.4 (-0.7 to -0.2) | -0.8 (-1.0 to -0.7) | -1.3 (-1.5 to -1.1) | -1.3 (-1.5 to -1.1) | -0.2 (-0.5 to 0.1) | 2.0 (1.8 to 2.2) | -0.7 (-0.9 to -0.4) | -0.4 (-0.7 to -0.2) | -0.6 (-0.9 to -0.4) | 2.6 (2.4 to 2.8) |
| Portugal | 0.8 (0.5 to 1.2) | -1.8 (-2.0 to -1.5) | -1.6 (-1.8 to -1.3) | -1.9 (-2.1 to -1.7) | 1.1 (0.7 to 1.4) | 0.6 (0.4 to 0.9) | -0.7 (-0.8 to -0.5) | -0.6 (-0.8 to -0.4) | -0.8 (-0.9 to -0.6) | 1.0 (0.8 to 1.3) | 1.8 (1.5 to 2.2) | 0.3 (0.0 to 0.5) | 0.1 (-0.1 to 0.3) | -0.0 (-0.2 to 0.2) | 2.2 (1.9 to 2.6) | 1.2 (0.9 to 1.5) | -2.5 (-2.7 to -2.3) | -2.2 (-2.5 to -2.0) | -2.9 (-3.1 to -2.6) | 1.4 (1.1 to 1.7) |
| Puerto Rico | -0.3 (-0.8 to 0.1) | -2.1 (-2.5 to -1.7) | -2.0 (-2.4 to -1.7) | -2.2 (-2.5 to -1.8) | -0.2 (-0.6 to 0.2) | 0.3 (-0.0 to 0.6) | -0.4 (-0.7 to -0.1) | -0.6 (-0.8 to -0.3) | -0.6 (-0.9 to -0.4) | 0.6 (0.3 to 0.9) | 1.1 (0.9 to 1.3) | -0.3 (-0.5 to -0.1) | -0.5 (-0.7 to -0.4) | -0.6 (-0.8 to -0.4) | 1.5 (1.3 to 1.7) | 7.3 (6.2 to 8.4) | 3.8 (2.8 to 4.7) | 4.2 (3.2 to 5.1) | 3.8 (2.8 to 4.7) | 7.6 (6.5 to 8.7) |
| Qatar | 1.0 (0.8 to 1.2) | -1.1 (-1.3 to -0.9) | -1.2 (-1.4 to -1.1) | -1.4 (-1.6 to -1.3) | 1.5 (1.4 to 1.7) | -2.4 (-2.7 to -2.0) | -3.0 (-3.3 to -2.7) | -3.4 (-3.7 to -3.1) | -3.5 (-3.8 to -3.2) | -1.7 (-2.0 to -1.5) | -0.3 (-0.5 to -0.1) | -2.0 (-2.2 to -1.7) | -1.9 (-2.2 to -1.7) | -2.0 (-2.3 to -1.8) | 0.5 (0.3 to 0.6) | 0.9 (0.2 to 1.5) | -3.8 (-4.5 to -3.2) | -3.5 (-4.3 to -2.8) | -4.1 (-4.8 to -3.3) | 1.7 (1.1 to 2.3) |
| Republic of Korea | 4.1 (3.7 to 4.6) | 0.9 (0.6 to 1.2) | 0.8 (0.6 to 1.1) | 0.5 (0.2 to 0.8) | 4.7 (4.2 to 5.2) | -0.0 (-0.2 to 0.1) | -1.3 (-1.4 to -1.1) | -1.7 (-1.9 to -1.6) | -1.9 (-2.0 to -1.8) | 0.5 (0.3 to 0.6) | 1.7 (1.5 to 2.0) | -0.2 (-0.4 to -0.0) | -0.5 (-0.7 to -0.4) | -0.6 (-0.8 to -0.5) | 2.5 (2.2 to 2.9) | 2.7 (2.5 to 3.0) | -3.8 (-4.1 to -3.5) | -2.8 (-3.1 to -2.6) | -3.8 (-4.1 to -3.5) | 3.0 (2.7 to 3.3) |
| Republic of Moldova | 3.6 (3.1 to 4.1) | 1.8 (1.4 to 2.3) | 2.3 (1.8 to 2.7) | 2.1 (1.7 to 2.6) | 3.8 (3.3 to 4.3) | 0.0 (-0.1 to 0.2) | -0.8 (-1.0 to -0.6) | -0.6 (-0.7 to -0.4) | -0.6 (-0.8 to -0.4) | 0.6 (0.4 to 0.7) | 1.6 (1.3 to 2.0) | 0.9 (0.5 to 1.2) | 0.8 (0.5 to 1.2) | 0.8 (0.5 to 1.2) | 1.9 (1.6 to 2.2) | 0.8 (0.6 to 1.0) | -1.3 (-1.5 to -1.1) | -1.3 (-1.5 to -1.1) | -1.4 (-1.7 to -1.2) | 1.7 (1.5 to 2.0) |
| Romania | 2.8 (2.5 to 3.0) | 1.3 (1.2 to 1.5) | 1.3 (1.1 to 1.4) | 1.2 (1.0 to 1.3) | 3.1 (2.9 to 3.3) | 1.5 (1.3 to 1.7) | 1.1 (0.9 to 1.2) | 0.7 (0.5 to 0.8) | 0.6 (0.5 to 0.8) | 1.7 (1.5 to 1.9) | 2.8 (2.5 to 3.1) | 2.2 (1.9 to 2.4) | 1.8 (1.5 to 2.0) | 1.7 (1.5 to 2.0) | 2.9 (2.6 to 3.2) | 2.2 (1.9 to 2.5) | -0.6 (-0.7 to -0.4) | -0.6 (-0.7 to -0.4) | -0.8 (-0.9 to -0.6) | 2.9 (2.5 to 3.3) |
| Russian Federation | 4.1 (3.8 to 4.3) | 2.2 (2.0 to 2.5) | 2.1 (1.8 to 2.3) | 1.9 (1.6 to 2.1) | 4.1 (3.8 to 4.4) | -0.6 (-0.8 to -0.3) | -1.4 (-1.7 to -1.1) | -1.6 (-1.9 to -1.3) | -1.7 (-2.0 to -1.4) | -0.1 (-0.3 to 0.1) | 0.7 (0.6 to 0.9) | -0.1 (-0.3 to 0.0) | -0.5 (-0.8 to -0.3) | -0.6 (-0.8 to -0.4) | 1.1 (0.9 to 1.3) | 2.4 (2.2 to 2.6) | -1.4 (-1.7 to -1.2) | -1.0 (-1.4 to -0.7) | -1.4 (-1.7 to -1.0) | 3.1 (2.9 to 3.4) |
| Saint Kitts and Nevis | 0.8 (0.5 to 1.1) | -0.0 (-0.3 to 0.3) | -0.0 (-0.3 to 0.2) | -0.1 (-0.4 to 0.1) | 1.5 (1.1 to 1.8) | 0.0 (-0.2 to 0.2) | -0.4 (-0.6 to -0.3) | -0.5 (-0.7 to -0.4) | -0.6 (-0.7 to -0.4) | 0.6 (0.5 to 0.8) | 0.5 (0.4 to 0.7) | -0.3 (-0.4 to -0.1) | -0.3 (-0.5 to -0.2) | -0.4 (-0.5 to -0.2) | 1.4 (1.2 to 1.6) | 7.5 (6.2 to 8.9) | 5.0 (3.9 to 6.1) | 5.5 (4.4 to 6.6) | 5.4 (4.2 to 6.5) | 9.4 (7.8 to 11.0) |
| Saint Lucia | 0.1 (-0.1 to 0.4) | -0.8 (-1.0 to -0.6) | -0.7 (-0.9 to -0.5) | -0.8 (-1.0 to -0.6) | 0.4 (0.2 to 0.7) | -0.2 (-0.4 to 0.0) | -0.8 (-1.1 to -0.6) | -0.6 (-0.9 to -0.4) | -0.7 (-0.9 to -0.5) | 0.3 (0.1 to 0.5) | 0.5 (0.3 to 0.7) | -0.5 (-0.7 to -0.4) | -0.4 (-0.5 to -0.2) | -0.4 (-0.5 to -0.2) | 1.0 (0.8 to 1.2) | 7.1 (5.9 to 8.4) | 4.7 (3.6 to 5.8) | 5.2 (4.0 to 6.3) | 5.0 (3.9 to 6.1) | 8.1 (6.7 to 9.5) |
| Saint Vincent and the Grenadines | 0.3 (-0.0 to 0.6) | -0.3 (-0.6 to 0.1) | -0.2 (-0.5 to 0.2) | -0.2 (-0.5 to 0.1) | 0.4 (0.1 to 0.7) | -0.2 (-0.3 to 0.0) | -0.7 (-0.9 to -0.5) | -0.3 (-0.5 to -0.2) | -0.4 (-0.5 to -0.2) | 0.2 (0.1 to 0.4) | 0.7 (0.5 to 0.9) | -0.0 (-0.2 to 0.1) | 0.2 (-0.0 to 0.4) | 0.2 (-0.0 to 0.4) | 1.0 (0.8 to 1.3) | 5.9 (4.7 to 7.2) | 4.6 (3.4 to 5.8) | 4.8 (3.6 to 6.0) | 4.7 (3.5 to 5.9) | 6.6 (5.3 to 7.9) |
| Samoa | 0.7 (0.5 to 0.9) | 0.2 (0.0 to 0.4) | 0.2 (0.1 to 0.4) | 0.2 (0.0 to 0.4) | 0.7 (0.5 to 1.0) | 0.5 (0.3 to 0.8) | 0.2 (0.0 to 0.4) | 0.2 (0.0 to 0.4) | 0.2 (-0.0 to 0.4) | 0.5 (0.3 to 0.7) | -0.0 (-0.1 to 0.0) | -0.1 (-0.2 to 0.0) | -0.1 (-0.1 to -0.0) | -0.1 (-0.1 to -0.0) | -0.1 (-0.1 to -0.0) | 0.5 (0.2 to 0.7) | -0.1 (-0.2 to 0.1) | -0.2 (-0.3 to -0.0) | -0.2 (-0.4 to -0.1) | 1.1 (0.9 to 1.4) |
| San Marino | -1.7 (-2.0 to -1.3) | -2.5 (-2.7 to -2.4) | -2.7 (-2.9 to -2.4) | -2.9 (-3.1 to -2.6) | -1.5 (-1.9 to -1.1) | -2.5 (-2.8 to -2.2) | -2.8 (-2.9 to -2.6) | -3.0 (-3.2 to -2.8) | -3.1 (-3.3 to -2.9) | -2.2 (-2.5 to -1.9) | -0.8 (-1.0 to -0.6) | -1.6 (-1.7 to -1.4) | -1.7 (-1.9 to -1.6) | -1.8 (-1.9 to -1.6) | -0.5 (-0.7 to -0.2) | 1.5 (1.0 to 2.0) | -0.7 (-1.1 to -0.2) | 0.1 (-0.3 to 0.6) | -0.2 (-0.8 to 0.3) | 1.1 (0.8 to 1.4) |
| Sao Tome and Principe | 1.9 (1.8 to 2.0) | 1.2 (1.0 to 1.3) | 1.3 (1.2 to 1.4) | 1.3 (1.1 to 1.4) | 2.2 (2.2 to 2.3) | 1.7 (1.6 to 1.8) | 1.2 (1.1 to 1.4) | 1.4 (1.3 to 1.5) | 1.4 (1.3 to 1.5) | 1.6 (1.4 to 1.7) | 1.2 (1.0 to 1.5) | 1.1 (0.9 to 1.3) | 0.5 (0.3 to 0.7) | 0.5 (0.3 to 0.7) | 0.7 (0.5 to 0.9) | 1.7 (1.2 to 2.1) | 0.1 (-0.2 to 0.4) | 0.1 (-0.2 to 0.5) | 0.1 (-0.2 to 0.4) | 2.5 (2.0 to 3.0) |
| Saudi Arabia | 1.8 (1.5 to 2.0) | 0.1 (-0.1 to 0.3) | 0.1 (-0.1 to 0.3) | -0.0 (-0.2 to 0.2) | 2.1 (1.9 to 2.4) | -0.2 (-0.4 to -0.0) | -0.9 (-1.1 to -0.7) | -1.1 (-1.3 to -0.9) | -1.1 (-1.3 to -1.0) | -0.0 (-0.2 to 0.1) | 3.0 (2.7 to 3.2) | 2.1 (1.8 to 2.3) | 1.5 (1.3 to 1.8) | 1.5 (1.3 to 1.8) | 3.1 (2.8 to 3.3) | 3.0 (2.5 to 3.5) | -0.2 (-0.6 to 0.2) | -0.6 (-1.0 to -0.1) | -0.8 (-1.3 to -0.3) | 4.2 (3.7 to 4.7) |
| Senegal | 2.0 (1.8 to 2.3) | 1.6 (1.4 to 1.8) | 1.6 (1.4 to 1.8) | 1.6 (1.4 to 1.8) | 2.2 (2.0 to 2.4) | -0.2 (-0.5 to 0.0) | -0.2 (-0.5 to 0.0) | -0.4 (-0.7 to -0.2) | -0.4 (-0.7 to -0.2) | -0.3 (-0.5 to -0.1) | 1.4 (1.3 to 1.6) | 1.4 (1.2 to 1.6) | 0.8 (0.7 to 1.0) | 0.8 (0.7 to 1.0) | 1.2 (1.0 to 1.4) | -1.4 (-1.9 to -0.9) | -2.1 (-2.5 to -1.7) | -2.4 (-2.9 to -2.0) | -2.5 (-2.9 to -2.0) | -0.8 (-1.3 to -0.3) |
| Serbia | 1.7 (1.5 to 1.9) | 0.5 (0.3 to 0.6) | 0.3 (0.2 to 0.5) | 0.2 (0.1 to 0.4) | 2.0 (1.8 to 2.3) | 1.1 (1.0 to 1.2) | 0.4 (0.3 to 0.5) | 0.2 (0.1 to 0.2) | 0.1 (0.1 to 0.2) | 1.4 (1.3 to 1.5) | 0.9 (0.8 to 1.1) | 0.1 (0.0 to 0.2) | -0.0 (-0.1 to 0.1) | -0.0 (-0.1 to 0.0) | 1.3 (1.2 to 1.5) | 1.3 (1.0 to 1.6) | -0.8 (-1.0 to -0.7) | -1.0 (-1.1 to -0.8) | -1.2 (-1.3 to -1.0) | 1.7 (1.4 to 2.1) |
| Seychelles | 1.3 (0.9 to 1.7) | 0.5 (0.1 to 0.8) | 0.4 (0.0 to 0.7) | 0.3 (-0.0 to 0.7) | 1.7 (1.3 to 2.1) | -0.2 (-0.4 to 0.0) | -0.7 (-0.9 to -0.5) | -0.8 (-1.1 to -0.6) | -0.9 (-1.1 to -0.7) | 0.2 (0.0 to 0.4) | 0.3 (0.0 to 0.5) | -0.1 (-0.4 to 0.1) | -0.2 (-0.4 to 0.1) | -0.2 (-0.5 to 0.1) | 0.5 (0.3 to 0.7) | 1.0 (0.8 to 1.1) | -0.9 (-1.1 to -0.8) | -0.7 (-0.8 to -0.6) | -0.8 (-0.9 to -0.6) | 2.0 (1.8 to 2.2) |
| Singapore | 2.0 (1.7 to 2.3) | -0.7 (-1.0 to -0.5) | -0.7 (-1.0 to -0.5) | -1.0 (-1.3 to -0.8) | 2.3 (2.0 to 2.6) | -0.9 (-1.1 to -0.7) | -1.9 (-2.1 to -1.7) | -2.2 (-2.4 to -2.0) | -2.3 (-2.5 to -2.1) | -0.5 (-0.6 to -0.3) | 0.7 (0.4 to 0.9) | -0.9 (-1.1 to -0.7) | -1.1 (-1.4 to -1.0) | -1.2 (-1.4 to -1.0) | 1.4 (1.1 to 1.6) | 1.9 (1.5 to 2.4) | -3.2 (-3.6 to -2.8) | -2.3 (-2.8 to -1.8) | -3.1 (-3.6 to -2.6) | 2.2 (1.8 to 2.6) |
| Slovakia | 1.7 (1.5 to 1.8) | 0.4 (0.3 to 0.5) | 0.3 (0.2 to 0.4) | 0.2 (0.1 to 0.3) | 1.9 (1.8 to 2.1) | 0.6 (0.5 to 0.7) | 0.2 (0.1 to 0.3) | -0.1 (-0.2 to -0.0) | -0.1 (-0.2 to -0.0) | 0.8 (0.7 to 0.8) | 1.0 (0.8 to 1.3) | 0.3 (0.1 to 0.6) | 0.1 (-0.1 to 0.3) | 0.0 (-0.2 to 0.3) | 1.3 (1.1 to 1.6) | 2.2 (2.1 to 2.3) | -0.6 (-0.7 to -0.5) | -0.4 (-0.5 to -0.2) | -0.6 (-0.8 to -0.5) | 2.5 (2.5 to 2.6) |
| Slovenia | 2.0 (1.7 to 2.4) | -0.1 (-0.4 to 0.2) | -0.2 (-0.6 to 0.1) | -0.5 (-0.8 to -0.1) | 2.3 (1.9 to 2.6) | 0.1 (0.0 to 0.3) | -0.4 (-0.5 to -0.3) | -0.8 (-0.9 to -0.7) | -0.8 (-1.0 to -0.7) | 0.5 (0.3 to 0.6) | 1.3 (0.8 to 1.9) | 0.3 (-0.2 to 0.7) | -0.1 (-0.6 to 0.3) | -0.2 (-0.7 to 0.3) | 1.7 (1.1 to 2.2) | 2.1 (1.9 to 2.4) | -1.9 (-2.1 to -1.8) | -1.6 (-1.8 to -1.4) | -2.3 (-2.5 to -2.1) | 2.3 (2.0 to 2.6) |
| Solomon Islands | 0.7 (0.6 to 0.9) | 0.4 (0.2 to 0.5) | 0.4 (0.2 to 0.6) | 0.4 (0.2 to 0.6) | 0.7 (0.6 to 0.9) | 0.8 (0.6 to 1.0) | 0.4 (0.2 to 0.6) | 0.5 (0.3 to 0.7) | 0.5 (0.3 to 0.7) | 0.8 (0.7 to 1.0) | 0.5 (0.3 to 0.6) | 0.5 (0.3 to 0.6) | 0.5 (0.4 to 0.6) | 0.5 (0.4 to 0.6) | 0.4 (0.2 to 0.5) | 1.4 (1.2 to 1.6) | 1.0 (0.8 to 1.2) | 1.1 (0.9 to 1.2) | 1.1 (0.9 to 1.2) | 1.5 (1.2 to 1.8) |
| South Africa | 1.0 (0.8 to 1.3) | 0.5 (0.2 to 0.8) | 0.6 (0.3 to 0.8) | 0.6 (0.3 to 0.8) | 1.5 (1.2 to 1.8) | 0.1 (-0.2 to 0.4) | -0.1 (-0.3 to 0.2) | -0.0 (-0.3 to 0.3) | -0.0 (-0.3 to 0.2) | 0.3 (-0.1 to 0.6) | 1.3 (1.0 to 1.5) | 0.9 (0.6 to 1.2) | 0.9 (0.6 to 1.1) | 0.9 (0.6 to 1.1) | 1.5 (1.2 to 1.8) | 0.9 (0.6 to 1.3) | 0.1 (-0.1 to 0.4) | 0.3 (0.0 to 0.5) | 0.2 (-0.0 to 0.5) | 1.8 (1.4 to 2.3) |
| Spain | 0.1 (-0.1 to 0.4) | -2.2 (-2.4 to -2.1) | -2.1 (-2.2 to -2.0) | -2.4 (-2.5 to -2.3) | 0.3 (0.1 to 0.6) | -0.4 (-0.7 to -0.1) | -1.3 (-1.4 to -1.1) | -1.6 (-1.8 to -1.4) | -1.7 (-1.9 to -1.5) | -0.0 (-0.4 to 0.2) | 1.6 (1.5 to 1.8) | 0.5 (0.5 to 0.6) | 0.2 (0.1 to 0.3) | 0.1 (0.0 to 0.2) | 2.0 (1.8 to 2.2) | 0.4 (0.2 to 0.6) | -2.5 (-2.7 to -2.2) | -2.1 (-2.4 to -1.8) | -2.9 (-3.2 to -2.6) | 0.5 (0.2 to 0.7) |
| Sri Lanka | 2.9 (2.8 to 3.1) | 1.2 (1.1 to 1.3) | 1.2 (1.1 to 1.3) | 1.1 (0.9 to 1.2) | 3.5 (3.3 to 3.7) | 2.4 (2.2 to 2.6) | 1.5 (1.3 to 1.8) | 1.3 (1.1 to 1.6) | 1.3 (1.0 to 1.5) | 2.6 (2.4 to 2.8) | -0.3 (-0.6 to -0.0) | -1.2 (-1.4 to -0.9) | -1.3 (-1.5 to -1.0) | -1.3 (-1.6 to -1.0) | 0.1 (-0.1 to 0.4) | 2.6 (2.4 to 3.0) | 0.0 (-0.2 to 0.3) | -0.1 (-0.4 to 0.2) | -0.3 (-0.6 to 0.0) | 3.8 (3.5 to 4.0) |
| Suriname | 1.0 (0.8 to 1.2) | 0.6 (0.4 to 0.8) | 0.6 (0.4 to 0.8) | 0.6 (0.4 to 0.8) | 1.4 (1.2 to 1.5) | 0.9 (0.7 to 1.1) | 0.6 (0.5 to 0.8) | 0.6 (0.4 to 0.8) | 0.6 (0.4 to 0.8) | 1.2 (1.0 to 1.3) | 0.9 (0.7 to 1.0) | 0.3 (0.1 to 0.4) | 0.3 (0.1 to 0.4) | 0.2 (0.1 to 0.4) | 1.3 (1.2 to 1.4) | 5.3 (4.5 to 6.1) | 3.9 (3.1 to 4.6) | 4.1 (3.4 to 4.9) | 4.1 (3.4 to 4.8) | 6.3 (5.5 to 7.1) |
| Sweden | -0.2 (-0.6 to 0.2) | -1.3 (-1.6 to -1.1) | -1.6 (-1.9 to -1.4) | -1.8 (-2.0 to -1.5) | -0.3 (-0.7 to 0.1) | -0.4 (-0.7 to -0.1) | -0.9 (-1.0 to -0.7) | -1.2 (-1.4 to -1.0) | -1.3 (-1.5 to -1.1) | -0.2 (-0.5 to 0.1) | -0.4 (-0.6 to -0.2) | -1.3 (-1.4 to -1.2) | -1.6 (-1.8 to -1.5) | -1.7 (-1.8 to -1.6) | 0.0 (-0.1 to 0.2) | 1.4 (1.1 to 1.6) | -1.3 (-1.5 to -1.1) | -0.7 (-0.9 to -0.5) | -1.4 (-1.6 to -1.1) | 1.4 (1.1 to 1.7) |
| Switzerland | -1.8 (-2.0 to -1.6) | -3.3 (-3.6 to -2.9) | -3.4 (-3.7 to -3.0) | -3.6 (-3.9 to -3.2) | -1.7 (-1.9 to -1.6) | 1.2 (0.4 to 2.0) | 0.8 (0.1 to 1.5) | 0.3 (-0.3 to 1.0) | 0.3 (-0.4 to 1.0) | 1.2 (0.5 to 1.9) | 1.1 (0.3 to 1.8) | 0.4 (-0.4 to 1.2) | -0.2 (-0.9 to 0.5) | -0.3 (-1.0 to 0.4) | 1.2 (0.5 to 2.0) | -0.8 (-1.0 to -0.6) | -2.6 (-2.8 to -2.4) | -2.5 (-2.7 to -2.4) | -3.2 (-3.4 to -3.0) | -0.8 (-1.0 to -0.5) |
| Taiwan (Province of China) | 2.8 (2.3 to 3.3) | 0.8 (0.5 to 1.1) | 0.8 (0.5 to 1.2) | 0.6 (0.3 to 0.9) | 2.9 (2.4 to 3.4) | -0.2 (-0.4 to 0.0) | -1.1 (-1.2 to -0.9) | -1.3 (-1.5 to -1.1) | -1.4 (-1.6 to -1.3) | 0.2 (-0.1 to 0.4) | 3.3 (2.4 to 4.2) | 2.6 (1.7 to 3.6) | 2.1 (1.2 to 3.0) | 2.1 (1.1 to 3.0) | 3.2 (2.4 to 4.1) | 1.9 (1.5 to 2.3) | -1.3 (-1.7 to -1.0) | -0.9 (-1.3 to -0.6) | -1.4 (-1.7 to -1.0) | 2.2 (1.7 to 2.6) |
| Tajikistan | 0.3 (-0.0 to 0.6) | -0.1 (-0.4 to 0.2) | -0.3 (-0.6 to -0.0) | -0.4 (-0.7 to -0.1) | 0.3 (0.1 to 0.6) | -0.4 (-0.8 to -0.0) | -0.5 (-0.8 to -0.1) | -0.8 (-1.1 to -0.4) | -0.8 (-1.2 to -0.4) | -0.4 (-0.8 to -0.1) | 0.3 (-0.0 to 0.6) | -0.0 (-0.3 to 0.3) | -0.2 (-0.5 to 0.1) | -0.2 (-0.5 to 0.1) | 0.5 (0.2 to 0.9) | -0.9 (-1.4 to -0.4) | -0.3 (-0.7 to 0.1) | -1.8 (-2.1 to -1.4) | -1.8 (-2.2 to -1.5) | 0.1 (-0.6 to 0.9) |
| Thailand | 0.8 (0.5 to 1.1) | -0.9 (-1.3 to -0.6) | -0.7 (-1.0 to -0.4) | -0.8 (-1.1 to -0.5) | 1.3 (1.0 to 1.6) | -0.1 (-0.3 to 0.2) | -1.2 (-1.5 to -1.0) | -1.0 (-1.2 to -0.8) | -1.1 (-1.3 to -0.8) | 0.6 (0.4 to 0.8) | 0.7 (0.5 to 0.9) | -0.1 (-0.3 to 0.1) | -0.0 (-0.2 to 0.2) | -0.0 (-0.3 to 0.2) | 1.0 (0.8 to 1.2) | 2.3 (1.8 to 2.8) | -0.4 (-0.8 to -0.0) | -0.1 (-0.6 to 0.4) | -0.3 (-0.8 to 0.2) | 3.2 (2.8 to 3.7) |
| Timor-Leste | 0.1 (0.0 to 0.2) | -0.4 (-0.5 to -0.4) | -0.4 (-0.4 to -0.3) | -0.4 (-0.5 to -0.3) | 0.7 (0.6 to 0.8) | -0.3 (-0.5 to -0.1) | -1.0 (-1.2 to -0.8) | -0.8 (-0.9 to -0.6) | -0.8 (-1.0 to -0.6) | 0.3 (0.2 to 0.4) | 1.0 (0.9 to 1.1) | 0.9 (0.8 to 1.0) | 0.6 (0.5 to 0.8) | 0.6 (0.5 to 0.8) | 1.1 (0.9 to 1.2) | 1.3 (1.0 to 1.7) | 0.2 (-0.1 to 0.4) | 0.3 (-0.0 to 0.7) | 0.3 (-0.1 to 0.6) | 2.1 (1.8 to 2.5) |
| Tonga | 0.4 (0.3 to 0.6) | -0.0 (-0.2 to 0.1) | -0.0 (-0.1 to 0.1) | -0.0 (-0.1 to 0.1) | 0.6 (0.5 to 0.7) | 0.4 (0.2 to 0.5) | -0.0 (-0.1 to 0.1) | 0.0 (-0.1 to 0.1) | -0.0 (-0.1 to 0.1) | 0.4 (0.3 to 0.5) | 0.4 (0.3 to 0.5) | 0.3 (0.2 to 0.4) | 0.3 (0.2 to 0.5) | 0.3 (0.2 to 0.5) | 0.3 (0.2 to 0.4) | 1.2 (1.1 to 1.3) | 0.6 (0.5 to 0.8) | 0.6 (0.5 to 0.6) | 0.5 (0.4 to 0.6) | 1.6 (1.5 to 1.8) |
| Trinidad and Tobago | 0.7 (0.5 to 1.0) | 0.1 (-0.2 to 0.3) | -0.1 (-0.4 to 0.2) | -0.2 (-0.4 to 0.1) | 1.0 (0.8 to 1.2) | 0.8 (0.6 to 1.0) | 0.2 (-0.0 to 0.4) | 0.3 (0.1 to 0.5) | 0.2 (0.0 to 0.4) | 1.2 (1.1 to 1.4) | 0.9 (0.8 to 1.1) | -0.0 (-0.2 to 0.1) | -0.0 (-0.2 to 0.1) | -0.0 (-0.2 to 0.1) | 1.4 (1.3 to 1.6) | 7.1 (5.7 to 8.4) | 4.4 (3.1 to 5.7) | 4.9 (3.6 to 6.2) | 4.7 (3.4 to 6.0) | 8.2 (6.8 to 9.7) |
| Tunisia | 1.3 (1.1 to 1.4) | -0.7 (-0.9 to -0.6) | -0.4 (-0.5 to -0.2) | -0.5 (-0.6 to -0.4) | 1.8 (1.6 to 2.1) | 0.3 (0.2 to 0.4) | -0.6 (-0.7 to -0.5) | -0.3 (-0.4 to -0.1) | -0.3 (-0.4 to -0.2) | 0.8 (0.5 to 1.0) | -0.3 (-0.8 to 0.2) | -1.5 (-2.0 to -0.9) | -1.3 (-1.8 to -0.8) | -1.4 (-1.9 to -0.9) | 0.3 (-0.1 to 0.7) | 2.6 (2.5 to 2.7) | -0.5 (-0.6 to -0.4) | -0.2 (-0.3 to -0.1) | -0.4 (-0.5 to -0.3) | 3.2 (3.0 to 3.3) |
| Türkiye | 2.7 (2.5 to 2.9) | 0.8 (0.6 to 1.0) | 0.6 (0.5 to 0.8) | 0.5 (0.3 to 0.6) | 3.4 (3.2 to 3.6) | 1.0 (0.9 to 1.2) | 0.3 (0.1 to 0.6) | -0.1 (-0.3 to 0.1) | -0.1 (-0.3 to 0.1) | 1.4 (1.2 to 1.5) | 1.0 (0.9 to 1.1) | -0.3 (-0.5 to -0.2) | -0.5 (-0.7 to -0.4) | -0.6 (-0.7 to -0.4) | 1.6 (1.5 to 1.7) | 2.8 (2.4 to 3.1) | -1.5 (-1.9 to -1.1) | -1.4 (-1.8 to -0.9) | -1.7 (-2.1 to -1.3) | 4.1 (3.8 to 4.4) |
| Turkmenistan | 1.5 (1.1 to 1.8) | 0.7 (0.4 to 1.0) | 0.9 (0.6 to 1.1) | 0.8 (0.6 to 1.1) | 1.7 (1.3 to 2.0) | -0.8 (-1.1 to -0.4) | -1.2 (-1.5 to -0.8) | -1.0 (-1.4 to -0.7) | -1.0 (-1.4 to -0.7) | -0.4 (-0.7 to -0.1) | 7.2 (5.9 to 8.5) | 7.2 (6.0 to 8.4) | 6.7 (5.4 to 8.0) | 6.7 (5.4 to 8.0) | 7.0 (5.7 to 8.3) | 0.2 (0.0 to 0.3) | -0.6 (-0.6 to -0.5) | -0.8 (-0.9 to -0.6) | -0.8 (-1.0 to -0.6) | 0.9 (0.7 to 1.1) |
| Tuvalu | 1.1 (0.9 to 1.2) | 0.6 (0.4 to 0.7) | 0.6 (0.5 to 0.7) | 0.6 (0.5 to 0.7) | 1.4 (1.3 to 1.5) | 1.1 (0.9 to 1.2) | 0.5 (0.4 to 0.6) | 0.6 (0.5 to 0.7) | 0.6 (0.5 to 0.7) | 1.4 (1.3 to 1.4) | 1.5 (1.4 to 1.6) | 1.1 (1.0 to 1.2) | 1.3 (1.2 to 1.4) | 1.3 (1.2 to 1.4) | 1.7 (1.6 to 1.8) | 1.8 (1.6 to 2.0) | 1.0 (0.8 to 1.1) | 1.0 (0.9 to 1.2) | 1.0 (0.8 to 1.1) | 2.4 (2.3 to 2.5) |
| Ukraine | 2.0 (1.9 to 2.2) | 0.6 (0.4 to 0.8) | 0.9 (0.7 to 1.1) | 0.8 (0.6 to 1.0) | 2.2 (2.0 to 2.4) | 0.5 (0.2 to 0.8) | -0.1 (-0.3 to 0.2) | -0.0 (-0.3 to 0.3) | -0.0 (-0.3 to 0.2) | 0.8 (0.6 to 1.1) | 1.2 (0.9 to 1.6) | 0.8 (0.5 to 1.1) | 0.6 (0.3 to 0.9) | 0.6 (0.2 to 0.9) | 1.4 (1.0 to 1.7) | 2.0 (1.7 to 2.3) | 0.5 (0.2 to 0.8) | 0.5 (0.2 to 0.8) | 0.4 (0.1 to 0.7) | 2.8 (2.5 to 3.1) |
| United Arab Emirates | -1.5 (-1.8 to -1.1) | -1.6 (-1.9 to -1.4) | -2.3 (-2.6 to -2.0) | -2.3 (-2.6 to -2.0) | -1.6 (-2.0 to -1.2) | -3.2 (-3.6 to -2.9) | -2.9 (-3.2 to -2.6) | -3.8 (-4.2 to -3.4) | -3.8 (-4.2 to -3.5) | -3.1 (-3.6 to -2.8) | -2.9 (-3.4 to -2.4) | -3.1 (-3.6 to -2.6) | -3.9 (-4.4 to -3.3) | -3.9 (-4.4 to -3.4) | -2.5 (-3.0 to -2.0) | -0.7 (-1.1 to -0.3) | -3.1 (-3.4 to -2.8) | -2.8 (-3.1 to -2.5) | -3.1 (-3.4 to -2.8) | 1.6 (1.2 to 2.0) |
| United Kingdom | 0.7 (0.5 to 0.9) | -1.1 (-1.2 to -1.0) | -1.2 (-1.3 to -1.1) | -1.4 (-1.5 to -1.3) | 0.8 (0.6 to 1.0) | -1.4 (-1.7 to -1.2) | -1.5 (-1.7 to -1.3) | -1.9 (-2.1 to -1.7) | -1.9 (-2.1 to -1.7) | -1.4 (-1.6 to -1.2) | 1.0 (0.9 to 1.2) | 0.2 (0.1 to 0.3) | -0.1 (-0.2 to -0.0) | -0.2 (-0.3 to -0.1) | 1.4 (1.2 to 1.6) | -0.4 (-0.8 to -0.1) | -3.1 (-3.5 to -2.6) | -2.5 (-2.9 to -2.2) | -3.1 (-3.5 to -2.8) | -0.4 (-0.7 to -0.1) |
| United Republic of Tanzania | 0.6 (0.5 to 0.7) | 0.2 (0.1 to 0.3) | 0.2 (0.1 to 0.3) | 0.2 (0.1 to 0.3) | 0.8 (0.8 to 0.9) | 0.1 (0.0 to 0.2) | 0.1 (-0.0 to 0.2) | -0.0 (-0.1 to 0.1) | -0.0 (-0.1 to 0.1) | 0.1 (-0.0 to 0.2) | 0.7 (0.6 to 0.8) | 0.5 (0.4 to 0.6) | 0.3 (0.2 to 0.4) | 0.3 (0.2 to 0.4) | 0.6 (0.5 to 0.8) | 0.6 (0.4 to 0.7) | -0.1 (-0.3 to 0.1) | -0.3 (-0.5 to -0.1) | -0.3 (-0.5 to -0.1) | 0.9 (0.6 to 1.2) |
| United States of America | -1.3 (-1.4 to -1.2) | -2.2 (-2.4 to -2.0) | -2.1 (-2.2 to -1.9) | -2.2 (-2.4 to -2.1) | -1.4 (-1.5 to -1.3) | -0.2 (-0.4 to -0.0) | -0.2 (-0.3 to -0.1) | -0.5 (-0.6 to -0.3) | -0.5 (-0.6 to -0.4) | -0.2 (-0.4 to -0.0) | -0.2 (-0.4 to 0.0) | -0.6 (-0.7 to -0.5) | -0.9 (-1.0 to -0.8) | -0.9 (-1.0 to -0.8) | -0.2 (-0.4 to 0.1) | 0.9 (0.7 to 1.0) | -0.1 (-0.3 to 0.0) | 0.0 (-0.2 to 0.2) | -0.2 (-0.4 to 0.0) | 0.9 (0.7 to 1.0) |
| United States Virgin Islands | 0.1 (-0.1 to 0.3) | -1.9 (-2.0 to -1.7) | -1.2 (-1.4 to -1.0) | -1.3 (-1.5 to -1.2) | 0.6 (0.4 to 0.8) | -0.4 (-0.6 to -0.2) | -1.4 (-1.6 to -1.2) | -0.9 (-1.1 to -0.8) | -1.0 (-1.2 to -0.8) | 0.3 (0.1 to 0.5) | 1.3 (1.1 to 1.5) | -0.3 (-0.5 to -0.1) | 0.3 (0.1 to 0.4) | 0.2 (0.0 to 0.4) | 2.0 (1.8 to 2.1) | 8.1 (7.1 to 9.1) | 4.7 (3.8 to 5.6) | 5.6 (4.6 to 6.6) | 5.4 (4.4 to 6.4) | 8.6 (7.6 to 9.7) |
| Uruguay | 0.7 (0.4 to 1.1) | -0.3 (-0.6 to -0.1) | -0.4 (-0.7 to -0.1) | -0.5 (-0.7 to -0.2) | 1.0 (0.6 to 1.4) | -0.4 (-0.5 to -0.2) | -0.5 (-0.6 to -0.4) | -0.8 (-0.9 to -0.7) | -0.8 (-0.9 to -0.7) | -0.3 (-0.5 to -0.2) | 1.6 (1.5 to 1.8) | 1.0 (0.8 to 1.1) | 0.7 (0.6 to 0.9) | 0.7 (0.6 to 0.8) | 1.9 (1.7 to 2.1) | 3.1 (2.9 to 3.3) | 0.7 (0.6 to 0.9) | 1.0 (0.8 to 1.2) | 0.8 (0.6 to 1.0) | 3.4 (3.1 to 3.6) |
| Uzbekistan | 1.1 (0.3 to 1.9) | 0.3 (-0.5 to 1.0) | 0.3 (-0.4 to 1.1) | 0.3 (-0.5 to 1.1) | 1.3 (0.5 to 2.0) | 0.4 (0.3 to 0.6) | 0.2 (-0.0 to 0.3) | 0.0 (-0.1 to 0.2) | 0.0 (-0.2 to 0.2) | 0.5 (0.4 to 0.7) | 2.4 (2.1 to 2.6) | 2.0 (1.7 to 2.2) | 1.8 (1.6 to 2.0) | 1.8 (1.5 to 2.0) | 2.5 (2.2 to 2.8) | 1.6 (1.3 to 1.9) | 0.5 (0.2 to 0.8) | 0.3 (0.0 to 0.7) | 0.3 (-0.0 to 0.6) | 2.4 (2.0 to 2.7) |
| Vanuatu | 0.1 (-0.1 to 0.2) | -0.2 (-0.3 to -0.0) | -0.2 (-0.3 to -0.0) | -0.2 (-0.4 to -0.1) | 0.2 (0.1 to 0.4) | 0.0 (-0.1 to 0.2) | -0.3 (-0.5 to -0.1) | -0.2 (-0.4 to -0.1) | -0.3 (-0.4 to -0.1) | 0.2 (0.1 to 0.3) | 0.1 (0.0 to 0.2) | 0.2 (0.1 to 0.2) | 0.1 (0.1 to 0.2) | 0.2 (0.1 to 0.2) | 0.1 (-0.0 to 0.2) | 0.8 (0.7 to 1.0) | 0.4 (0.3 to 0.6) | 0.5 (0.3 to 0.6) | 0.4 (0.3 to 0.6) | 1.2 (0.9 to 1.5) |
| Viet Nam | 2.4 (2.3 to 2.5) | 0.9 (0.8 to 1.0) | 1.0 (0.9 to 1.1) | 0.9 (0.8 to 1.0) | 2.8 (2.7 to 2.8) | 1.8 (1.6 to 1.9) | 0.7 (0.5 to 0.9) | 0.9 (0.7 to 1.0) | 0.8 (0.6 to 1.0) | 2.1 (1.9 to 2.2) | 2.8 (2.6 to 2.9) | 2.1 (1.9 to 2.3) | 2.0 (1.8 to 2.2) | 2.0 (1.8 to 2.2) | 2.9 (2.8 to 3.1) | 3.2 (3.0 to 3.4) | 0.7 (0.6 to 0.8) | 0.8 (0.7 to 0.9) | 0.7 (0.6 to 0.8) | 4.2 (4.0 to 4.4) |
| Zambia | 0.6 (0.4 to 0.9) | 0.1 (-0.2 to 0.3) | 0.2 (0.0 to 0.4) | 0.2 (-0.0 to 0.4) | 0.6 (0.4 to 0.8) | 0.8 (0.6 to 1.0) | 0.4 (0.2 to 0.6) | 0.7 (0.5 to 0.8) | 0.7 (0.5 to 0.9) | 0.4 (0.2 to 0.6) | 1.4 (1.4 to 1.5) | 1.0 (0.9 to 1.1) | 1.0 (1.0 to 1.1) | 1.1 (1.0 to 1.1) | 0.7 (0.5 to 0.8) | 1.6 (1.1 to 2.0) | 0.7 (0.3 to 1.0) | 0.5 (0.1 to 0.9) | 0.5 (0.1 to 0.9) | 1.2 (0.6 to 1.7) |
| Zimbabwe | -0.7 (-1.1 to -0.2) | -0.7 (-1.1 to -0.3) | -0.5 (-0.9 to -0.1) | -0.5 (-0.9 to -0.1) | -1.0 (-1.5 to -0.4) | -2.2 (-2.8 to -1.5) | -1.9 (-2.5 to -1.3) | -1.8 (-2.4 to -1.2) | -1.8 (-2.4 to -1.1) | -2.6 (-3.3 to -2.0) | 0.3 (-0.2 to 0.8) | 0.2 (-0.3 to 0.7) | 0.5 (-0.0 to 1.0) | 0.5 (-0.0 to 1.0) | -0.3 (-0.8 to 0.1) | -1.1 (-1.8 to -0.4) | -1.1 (-1.8 to -0.5) | -0.7 (-1.4 to 0.1) | -0.6 (-1.4 to 0.1) | -2.0 (-2.8 to -1.3) |

## Table S7 Age-standardized DALYs rates for prostate, male bladder, and male kidney cancers attributable to risk factors in 2023.

| **Locations** | Prostate cancer | | Male bladder cancer | | Male kidney cancer | | |
| --- | --- | --- | --- | --- | --- | --- | --- |
|  | Alcohol use | Smoking | Smoking | High fasting plasma glucose | High body-mass index | Occupational exposure to trichloroethylene | Smoking |
| World Bank High Income | 6.9 (0.3 to 16.6) | 12.4 (5.6 to 20.8) | 36.0 (29.9 to 43.0) | 13.6 (8.3 to 19.9) | 26.4 (11.5 to 39.4) | 0.03 (0.01 to 0.06) | 14.9 (8.7 to 21.7) |
| World Bank Upper Middle Income | 2.0 (0.1 to 4.6) | 6.2 (2.8 to 10.9) | 29.0 (24.6 to 34.3) | 7.1 (4.4 to 10.6) | 9.4 (4.2 to 14.6) | 0.05 (0.01 to 0.10) | 7.0 (4.3 to 10.0) |
| World Bank Lower Middle Income | 1.2 (0.0 to 2.9) | 4.2 (1.9 to 8.3) | 14.0 (8.8 to 20.0) | 4.7 (2.5 to 7.9) | 2.9 (1.1 to 5.4) | 0.03 (0.01 to 0.05) | 2.0 (1.1 to 3.1) |
| Albania | 1.14 (0.04 to 2.88) | 7.68 (2.93 to 13.77) | 4.8 (2.8 to 7.1) | 1.2 (0.6 to 2.1) | 15.9 (5.9 to 30.4) | 0 (0 to 0) | 16.9 (9.2 to 28.8) |
| Algeria | 0.02 (0 to 0.06) | 1.13 (0.37 to 2.26) | 22.1 (11.4 to 36.5) | 7.4 (3.3 to 13.5) | 3.4 (1.2 to 7.1) | 0 (0 to 0) | 2.2 (0.9 to 3.9) |
| American Samoa | 1.8 (0.03 to 5.36) | 9.06 (3.4 to 16.35) | 14 (7.8 to 22.8) | 10.4 (5.6 to 17.5) | 11.4 (4.9 to 20.2) | 0 (0 to 0) | 2.3 (1.1 to 3.9) |
| Andorra | 3.09 (0 to 8.84) | 5.34 (2.09 to 10.01) | 41.4 (24.7 to 63.5) | 13.1 (6.2 to 24.2) | 17.6 (7.3 to 31.9) | 0 (0 to 0) | 9.4 (4.8 to 17.9) |
| Angola | 7.25 (0.24 to 16.89) | 6.66 (2.44 to 13.31) | 15.7 (8.8 to 24.8) | 8.1 (4.1 to 14.4) | 3.1 (1.1 to 6.2) | 0 (0 to 0) | 1.6 (0.8 to 3) |
| Antigua and Barbuda | 4.42 (0.15 to 14.91) | 15.43 (5.7 to 33.83) | 12.1 (8.1 to 16.5) | 4.5 (2.5 to 7.9) | 8.8 (3.5 to 14.3) | 0 (0 to 0.1) | 3.7 (1.9 to 6.7) |
| Argentina | 3.89 (0.13 to 9.39) | 4.1 (1.71 to 7.29) | 27.1 (20.1 to 36.2) | 9.7 (5.6 to 15.3) | 57 (27.2 to 86.8) | 0.2 (0 to 0.5) | 20.2 (11.7 to 30.9) |
| Armenia | 0.29 (0 to 1.29) | 5.07 (2.36 to 8.95) | 66.6 (54.3 to 81.5) | 9.5 (5.7 to 15.5) | 18.5 (7.7 to 30.3) | 0.1 (0 to 0.1) | 18.5 (11.6 to 25.9) |
| Australia | 4.45 (0.15 to 10.59) | 4.81 (2.08 to 8.48) | 15.1 (12 to 18.7) | 7.7 (4.4 to 12.3) | 25.2 (11.4 to 38.3) | 0 (0 to 0) | 9.6 (5.2 to 15.2) |
| Austria | 4.05 (0.14 to 10.06) | 6.19 (2.64 to 11.08) | 33.5 (26.4 to 42) | 9.3 (5.3 to 14.8) | 23.6 (10.5 to 35.5) | 0 (0 to 0) | 13.7 (8 to 20.6) |
| Azerbaijan | 0.9 (0.03 to 2.61) | 4.36 (1.83 to 7.95) | 34.2 (21.5 to 49.8) | 6.9 (3.4 to 12) | 23.8 (10.3 to 42) | 0.1 (0 to 0.2) | 18.4 (10.1 to 30.9) |
| Bahrain | 0.31 (0.01 to 0.87) | 4.91 (1.86 to 8.87) | 8.5 (5.7 to 12.5) | 5.8 (3.3 to 9.3) | 21 (9 to 34.5) | 0.1 (0 to 0.1) | 3.3 (1.7 to 6.1) |
| Bangladesh | 0.01 (0 to 0.02) | 3.32 (1.31 to 7.19) | 45.2 (28.1 to 69) | 18.5 (9.6 to 31.5) | 11.7 (4.7 to 21) | 0 (0 to 0.1) | 5.4 (2.6 to 9.6) |
| Barbados | 2.79 (0.09 to 10.53) | 9.32 (3.46 to 19.61) | 20.1 (10.6 to 35.9) | 4.7 (2.1 to 9.2) | 2.2 (0.8 to 4.6) | 0 (0 to 0.1) | 2.9 (1.3 to 5.8) |
| Belarus | 4.4 (0.13 to 12.57) | 11.22 (4.7 to 20.08) | 11.8 (7.7 to 17.4) | 8.9 (5 to 14.2) | 22.2 (10.1 to 36.5) | 0.1 (0 to 0.2) | 5.3 (2.9 to 9.5) |
| Belgium | 3.33 (0.11 to 8.52) | 5.68 (2.33 to 10.02) | 56.1 (46.2 to 67.7) | 8 (4.8 to 12.6) | 40.5 (18.9 to 63) | 0 (0 to 0) | 31.1 (18.9 to 44) |
| Belize | 4.24 (0.15 to 11.38) | 8.59 (3.33 to 15.67) | 35.4 (27 to 44.9) | 13.4 (6.8 to 21.9) | 24.4 (10.9 to 38.2) | 0 (0 to 0) | 12.9 (7.2 to 19.9) |
| Benin | 2.2 (0.08 to 5.42) | 3.01 (1.1 to 6.12) | 11.3 (7.7 to 15.9) | 3.6 (2 to 6) | 10.1 (4 to 16.8) | 0.1 (0 to 0.1) | 3.9 (2 to 6.5) |
| Bermuda | 6.64 (0.25 to 17.72) | 6.97 (2.61 to 14.45) | 3.2 (1.7 to 5.7) | 2.2 (1.1 to 4) | 6.4 (2.2 to 14.2) | 0 (0 to 0.1) | 0.9 (0.4 to 1.7) |
| Bhutan | 0.24 (0.01 to 0.75) | 1.03 (0.3 to 2.52) | 31.1 (21 to 42.4) | 12.2 (7 to 20.1) | 15.3 (5.8 to 26.1) | 0.1 (0 to 0.1) | 5.6 (3 to 9.7) |
| Bosnia and Herzegovina | 2.59 (0.09 to 6.48) | 6.93 (2.9 to 11.86) | 4.7 (2.1 to 8.5) | 2 (0.8 to 3.8) | 3.7 (1.3 to 7.4) | 0 (0 to 0) | 1.4 (0.5 to 3) |
| Botswana | 1.21 (0.04 to 2.88) | 3.07 (1.28 to 5.98) | 10.4 (5.5 to 18.7) | 6.7 (3 to 12.5) | 17.6 (6.8 to 32.3) | 0.1 (0 to 0.2) | 2.7 (1.3 to 4.6) |
| Brazil | 2.17 (0.08 to 5.2) | 5.22 (2.26 to 9.38) | 67.2 (49.3 to 92.8) | 20 (10.4 to 32.5) | 22.9 (9.1 to 40.3) | 0 (0 to 0) | 20.6 (10.4 to 33) |
| Brunei Darussalam | 0.09 (0 to 0.24) | 3.3 (1.17 to 6.37) | 4.3 (2.5 to 6.8) | 0.9 (0.5 to 1.6) | 2.4 (0.9 to 4.9) | 0 (0 to 0) | 1.1 (0.5 to 1.9) |
| Bulgaria | 3.69 (0.12 to 9.66) | 7.69 (3.44 to 12.96) | 16.9 (13.5 to 20.2) | 8.2 (4.4 to 13.2) | 17.1 (7.2 to 25.8) | 0.1 (0 to 0.1) | 6.4 (3.7 to 10) |
| Cambodia | 0.87 (0.03 to 1.93) | 2.51 (0.93 to 4.33) | 17 (10.5 to 26.2) | 6.3 (3.2 to 10.8) | 17.9 (7.1 to 31.2) | 0 (0 to 0) | 10.9 (5.6 to 19) |
| Cameroon | 3.35 (0.12 to 9.56) | 3.5 (1.26 to 7.24) | 62.4 (51.9 to 74.8) | 18.7 (10.2 to 28.8) | 25.5 (11 to 38) | 0 (0 to 0) | 21.9 (13.7 to 31.9) |
| Canada | 1.99 (0.07 to 5.71) | 5.54 (2.17 to 10.69) | 8.3 (4.1 to 14.2) | 5.6 (2.6 to 10.8) | 4.8 (0.8 to 11.8) | 0 (0 to 0) | 1 (0.2 to 2.2) |
| Chile | 4.15 (0.15 to 9.37) | 3.29 (1.37 to 6.01) | 14.4 (7.8 to 24) | 0.9 (0.4 to 1.7) | 1.7 (0.6 to 3.5) | 0 (0 to 0.1) | 3.7 (1.8 to 6.3) |
| China | 0.51 (0.02 to 1.1) | 1.83 (0.79 to 3.4) | 10.5 (5.3 to 19.5) | 9.8 (4.9 to 17.5) | 6.1 (2.1 to 11.9) | 0 (0 to 0) | 0.9 (0.4 to 1.8) |
| Colombia | 0.86 (0.03 to 2.56) | 1.74 (0.62 to 3.4) | 25.6 (20 to 32.4) | 11.6 (6.8 to 17.4) | 27.4 (11.9 to 42.5) | 0 (0 to 0) | 12.9 (6.7 to 20.5) |
| Commonwealth of the Bahamas | 7.5 (0.3 to 21.56) | 9.87 (3.62 to 19.56) | 13.6 (10.6 to 17.3) | 7.2 (3.7 to 12) | 47.1 (22.5 to 69.7) | 0.2 (0 to 0.4) | 13.4 (7.7 to 21.5) |
| Comoros | 0.06 (0 to 0.16) | 1.13 (0.44 to 2.37) | 31.1 (24.6 to 37.5) | 6.8 (4.2 to 10.3) | 4.8 (1.9 to 8.1) | 0 (0 to 0.1) | 6.6 (4 to 10) |
| Congo | 7.68 (0.23 to 18.33) | 8.1 (3.22 to 16.16) | 4.7 (3.5 to 6.3) | 2.8 (1.7 to 4.2) | 11.2 (4.8 to 16.9) | 0.1 (0 to 0.1) | 2.2 (1.2 to 3.7) |
| Costa Rica | 2.62 (0.1 to 7.48) | 4.29 (1.66 to 7.96) | 5 (2.8 to 8) | 1.1 (0.5 to 2) | 2 (0.6 to 4) | 0 (0 to 0) | 0.7 (0.3 to 1.4) |
| Cote d Ivoire | 3.52 (0.11 to 8.82) | 5.5 (1.89 to 10.79) | 16.3 (8.7 to 28.6) | 10.1 (4.7 to 17.7) | 2.8 (1 to 5.8) | 0 (0 to 0) | 1.2 (0.6 to 2.1) |
| Croatia | 3.52 (0.13 to 9.4) | 8.95 (3.98 to 15.82) | 11.1 (8.1 to 14.4) | 7.2 (3.9 to 11.4) | 17.1 (7.7 to 25.7) | 0.1 (0 to 0.2) | 4.5 (2.2 to 7.6) |
| Cuba | 4.93 (0.21 to 12.4) | 14.52 (6.14 to 25.28) | 8.2 (4.2 to 14.2) | 5.3 (2.5 to 9.9) | 2.1 (0.8 to 4) | 0 (0 to 0) | 0.6 (0.3 to 1.1) |
| Cyprus | 3.58 (0.14 to 9.74) | 6.84 (2.66 to 13.79) | 69.7 (56.7 to 85.2) | 24.7 (14.2 to 36.7) | 37.4 (17.2 to 58.4) | 0 (0 to 0) | 28.5 (17.9 to 40.6) |
| Czech Republic | 4.74 (0.17 to 10.71) | 7.12 (3.08 to 11.89) | 44.1 (33.7 to 58.3) | 8.7 (4.8 to 13.6) | 18.8 (7.8 to 29.3) | 0.1 (0 to 0.2) | 11.3 (6.5 to 17.4) |
| Denmark | 6.63 (0.25 to 14.9) | 9.57 (3.92 to 17.4) | 51.4 (32.7 to 76.8) | 16.7 (8 to 31.1) | 19.3 (7.9 to 35.6) | 0 (0 to 0) | 11.6 (5.9 to 19.7) |
| Djibouti | 0.45 (0.01 to 1.12) | 4.52 (1.7 to 8.88) | 48.4 (39.4 to 59.1) | 20.6 (12.1 to 30.3) | 48.2 (22 to 73.6) | 0 (0 to 0.1) | 28.1 (16.6 to 40.3) |
| Dominica | 9.89 (0.39 to 26.16) | 20.76 (7.77 to 40.41) | 35.4 (27.8 to 43.5) | 11.7 (6.1 to 20.3) | 24.6 (10.5 to 36.9) | 0 (0 to 0) | 13.4 (7.2 to 20.5) |
| Dominican Republic | 4.39 (0.17 to 12.75) | 7.54 (2.66 to 15.07) | 14.5 (7.8 to 25.8) | 2.3 (1 to 4.1) | 1.4 (0.4 to 3.2) | 0 (0 to 0) | 1.3 (0.6 to 2.3) |
| Ecuador | 1.21 (0.05 to 3.31) | 3.22 (1.22 to 5.66) | 14.3 (9 to 21.6) | 7.9 (3.9 to 14.8) | 17.8 (7.1 to 33.7) | 0.1 (0 to 0.1) | 5.3 (2.3 to 9.9) |
| Egypt | 0.01 (0 to 0.04) | 4.42 (1.82 to 8.22) | 8.9 (5.5 to 14.4) | 3.7 (1.8 to 6.4) | 5.3 (1.9 to 10) | 0 (0 to 0.1) | 1.7 (0.8 to 3.2) |
| El Salvador | 1.8 (0.06 to 5.3) | 2.36 (0.82 to 5.17) | 5.1 (3.6 to 6.8) | 3.5 (2 to 5.2) | 12.7 (5.8 to 19.5) | 0.1 (0 to 0.1) | 2.4 (1.3 to 4) |
| Equatorial Guinea | 9.47 (0.24 to 22.2) | 6.09 (2.59 to 11.92) | 69.2 (42.7 to 104.5) | 15.9 (7.9 to 26.3) | 6 (2.6 to 10.4) | 0 (0 to 0) | 3.4 (1.8 to 5.4) |
| Estonia | 4.21 (0.12 to 13.47) | 10.01 (4.36 to 17.21) | 4.3 (2.6 to 6.5) | 4.4 (2.4 to 7.4) | 10.8 (4.3 to 19) | 0.1 (0 to 0.1) | 1.6 (0.7 to 3.2) |
| Federated States of Micronesia | 0.03 (0 to 0.19) | 4.3 (1.8 to 8.2) | 20.1 (10.9 to 35.3) | 18.9 (8.6 to 34.5) | 6.8 (2.7 to 12.9) | 0 (0 to 0) | 0.8 (0.4 to 1.5) |
| Fiji | 1.08 (0.05 to 2.72) | 5.95 (2.3 to 11.43) | 45.5 (35.4 to 57.7) | 14.7 (7.8 to 23.5) | 47.2 (19.5 to 74.4) | 0 (0 to 0) | 28 (15.5 to 41.9) |
| Finland | 4.43 (0.15 to 10.67) | 5.58 (2.44 to 9.96) | 2.7 (1.4 to 4.8) | 2.3 (1.2 to 4.2) | 7.4 (2.5 to 14.5) | 0 (0 to 0) | 0.6 (0.2 to 1.1) |
| France | 4.63 (0.16 to 11.32) | 5.41 (2.39 to 10.05) | 16.8 (9.6 to 27.9) | 9.3 (4.4 to 15.8) | 2.7 (1.1 to 5) | 0 (0 to 0) | 0.8 (0.4 to 1.5) |
| Gabon | 8.91 (0.29 to 19.66) | 7.03 (2.43 to 13.84) | 17 (12.9 to 21.7) | 10 (5.8 to 14.8) | 26.8 (11.8 to 41.1) | 0 (0 to 0) | 11 (6.2 to 17.2) |
| Georgia | 3.31 (0.08 to 9.42) | 13.95 (6.46 to 22.74) | 44.3 (34.4 to 54.8) | 16.2 (9.1 to 25.4) | 32.6 (14.8 to 51.9) | 0 (0 to 0) | 14.3 (8.6 to 22.2) |
| Germany | 6.07 (0.21 to 13.72) | 6.73 (2.88 to 11.17) | 11.6 (5.7 to 20.2) | 10.8 (4.9 to 19.6) | 6.6 (2.5 to 12.4) | 0 (0 to 0) | 1.1 (0.5 to 2) |
| Ghana | 3.84 (0.14 to 10.06) | 2.49 (0.93 to 5.3) | 102.3 (80.8 to 126) | 15.9 (9 to 25.8) | 35.7 (15.1 to 58.6) | 0.1 (0 to 0.2) | 30 (19.3 to 44.6) |
| Greece | 2.92 (0.11 to 6.71) | 7.21 (3.29 to 12.25) | 35.9 (28.1 to 45.3) | 13.2 (7.8 to 20) | 30.1 (12.8 to 46.6) | 0 (0 to 0.1) | 16.8 (9.2 to 26) |
| Greenland | 1.92 (0.06 to 6.33) | 5.5 (2.09 to 11.37) | 7.8 (4 to 13.9) | 12.4 (5.7 to 23.1) | 3.6 (1.1 to 7.1) | 0 (0 to 0) | 0.4 (0.2 to 0.8) |
| Grenada | 6.56 (0.26 to 18.02) | 10.01 (3.94 to 19.51) | 85.2 (69.7 to 100.7) | 22 (11.9 to 34.8) | 28.9 (12.9 to 43.6) | 0 (0 to 0) | 19.8 (11.3 to 28.9) |
| Guam | 0.31 (0.01 to 0.9) | 1.8 (0.7 to 3.39) | 46.1 (28.5 to 68.4) | 14.3 (7.2 to 24.1) | 45.9 (18.6 to 86.2) | 0 (0 to 0.1) | 23.8 (11 to 41.8) |
| Guatemala | 0.81 (0.03 to 2.46) | 2.7 (1.07 to 5.03) | 8.9 (5.6 to 12.6) | 4.1 (2.2 to 7.3) | 10.1 (4 to 17.5) | 0.1 (0 to 0.1) | 3 (1.7 to 5.6) |
| Guinea | 0.21 (0.01 to 0.51) | 5.47 (1.94 to 10.71) | 18.2 (13.4 to 23.6) | 9.7 (5.7 to 15.3) | 23.5 (10.1 to 36.8) | 0.1 (0 to 0.1) | 5.6 (3.2 to 9) |
| Guyana | 4.07 (0.13 to 14.3) | 15.38 (5.74 to 27.41) | 2.7 (1.8 to 4) | 2.6 (1.5 to 3.9) | 10.8 (4.7 to 18) | 0.1 (0 to 0.1) | 1.9 (1 to 3.4) |
| Haiti | 6.48 (0.21 to 16.2) | 4.86 (1.76 to 10.25) | 10.8 (5.7 to 17.9) | 3.1 (1.5 to 5.7) | 2 (0.7 to 4) | 0 (0 to 0) | 1 (0.4 to 1.8) |
| Honduras | 0.62 (0.02 to 1.61) | 1.33 (0.5 to 2.64) | 13.3 (9.6 to 19) | 4.8 (2.8 to 7.5) | 10.3 (4.2 to 18.1) | 0.1 (0 to 0.1) | 3.9 (2 to 6.8) |
| Hungary | 4.4 (0.16 to 10.78) | 5.86 (2.6 to 10.04) | 6.7 (3.1 to 11.2) | 3.1 (1.4 to 5.7) | 4.8 (1.6 to 10.7) | 0 (0 to 0.1) | 1 (0.4 to 2) |
| Iceland | 3.74 (0.13 to 9.4) | 8.12 (2.96 to 15.63) | 2.1 (1.1 to 3.7) | 1.5 (0.7 to 2.7) | 3.4 (1.3 to 6.7) | 0 (0 to 0) | 1 (0.5 to 1.9) |
| India | 0.19 (0.01 to 0.55) | 1.01 (0.44 to 2.02) | 68.2 (55.1 to 82.7) | 24.1 (13.8 to 35.7) | 40.9 (18.1 to 61.1) | 0 (0 to 0.1) | 23.9 (13.9 to 35) |
| Indonesia | 0.02 (0 to 0.08) | 3.79 (1.6 to 7.02) | 24.3 (17.6 to 32) | 11.2 (5.8 to 18.4) | 36.4 (16.1 to 57.1) | 0 (0 to 0.1) | 17.1 (8.7 to 28.2) |
| Iraq | 0.03 (0 to 0.08) | 7.11 (2.85 to 13.99) | 9.3 (5.8 to 14.5) | 3.9 (2 to 6.9) | 1.9 (0.7 to 3.7) | 0 (0 to 0) | 1.3 (0.7 to 2.2) |
| Ireland | 3.77 (0.13 to 9.18) | 4.17 (1.7 to 7.44) | 15.6 (8.4 to 28) | 2.6 (1.2 to 5) | 2.9 (1.1 to 5.6) | 0 (0 to 0.1) | 4.6 (2.4 to 7.5) |
| Islamic Republic of Iran | 0.02 (0 to 0.08) | 3.19 (1.28 to 6.43) | 20.4 (12.6 to 32) | 4.5 (2.3 to 7.7) | 10.4 (4.3 to 18) | 0 (0 to 0.1) | 4.2 (2.3 to 7.3) |
| Israel | 0.63 (0.01 to 1.81) | 2.87 (1.2 to 5.14) | 129.1 (86.8 to 180.8) | 39.4 (20.8 to 64.4) | 20.9 (8.7 to 35.4) | 0.1 (0 to 0.1) | 13.2 (7.2 to 23.5) |
| Italy | 3.01 (0.1 to 7.03) | 3.85 (1.65 to 6.43) | 19.9 (15.5 to 25.1) | 9.5 (5.2 to 16) | 27.3 (11.8 to 40.1) | 0 (0 to 0) | 8.9 (4.5 to 14.8) |
| Jamaica | 2.99 (0.09 to 9.89) | 11.36 (4.43 to 22.22) | 31.7 (24.8 to 40.8) | 13.3 (7 to 21.3) | 23.3 (10.2 to 34.9) | 0 (0 to 0) | 10.7 (5.4 to 17.8) |
| Japan | 1.05 (0.04 to 2.4) | 2.45 (0.98 to 4.13) | 44.4 (35.5 to 55) | 17.7 (10.8 to 26.3) | 25.1 (10.8 to 37.5) | 0 (0 to 0) | 12.5 (7.2 to 18.9) |
| Jordan | 0.04 (0 to 0.14) | 7.38 (3.11 to 13.78) | 14.6 (10.1 to 19.8) | 5.9 (3.6 to 9.8) | 7.3 (2.6 to 12.7) | 0.1 (0 to 0.1) | 3.2 (1.7 to 5.7) |
| Kazakhstan | 0.86 (0.02 to 2.5) | 2.76 (1.25 to 4.65) | 22.6 (18.7 to 27.3) | 9.5 (5.7 to 13.8) | 9 (3.7 to 15) | 0 (0 to 0) | 10.1 (5.9 to 15.2) |
| Kenya | 2.11 (0.06 to 5.01) | 2.2 (0.83 to 4.38) | 64.1 (42.1 to 92.6) | 22.9 (12.8 to 35.5) | 12.1 (5 to 21.3) | 0 (0 to 0.1) | 7.9 (4.2 to 13.2) |
| Kingdom of Eswatini | 2.6 (0.07 to 5.92) | 1.92 (0.71 to 3.8) | 29.2 (23.1 to 35.5) | 5.6 (3.4 to 8.9) | 17.4 (7.3 to 28.9) | 0.1 (0 to 0.1) | 10.4 (6.2 to 15.4) |
| Kiribati | 0.06 (0 to 0.22) | 1.6 (0.62 to 3.19) | 4.5 (2.5 to 7.3) | 1 (0.5 to 1.8) | 1.7 (0.6 to 3.6) | 0 (0 to 0) | 0.7 (0.4 to 1.3) |
| Kuwait | 0 (0 to 0.01) | 2.58 (1.01 to 4.64) | 3.4 (1.9 to 5.5) | 1.2 (0.6 to 2.1) | 8.1 (3.1 to 16.7) | 0 (0 to 0) | 3.2 (1.3 to 6.7) |
| Kyrgyzstan | 0.37 (0.01 to 1.09) | 2.07 (0.89 to 3.55) | 23 (16.4 to 31.3) | 14 (8.2 to 19.9) | 11.4 (5.4 to 17.9) | 0 (0 to 0.1) | 4.2 (2.4 to 6.8) |
| Lao People's Democratic Republic | 1.2 (0.05 to 2.89) | 3.6 (1.46 to 6.72) | 18.2 (13.9 to 23.2) | 3.4 (2.1 to 5.5) | 11.4 (4.7 to 18.6) | 0 (0 to 0.1) | 7.4 (4.4 to 10.8) |
| Latvia | 5.92 (0.19 to 16.38) | 9.58 (4.04 to 16.32) | 18.7 (10.4 to 29.1) | 2.1 (1 to 3.7) | 2 (0.7 to 4.2) | 0 (0 to 0) | 3.4 (1.6 to 5.7) |
| Lebanon | 0.31 (0.01 to 1.02) | 5.91 (2.41 to 11.03) | 81.2 (62 to 99.8) | 16.1 (8.9 to 29.9) | 52.2 (23.5 to 83.3) | 0 (0 to 0) | 33 (20.2 to 48) |
| Lesotho | 4.13 (0.1 to 9.87) | 2.7 (1.02 to 5.61) | 86.1 (57.9 to 120.3) | 26.1 (14.2 to 41) | 19 (8.4 to 32.6) | 0.1 (0 to 0.1) | 12.3 (6.6 to 19.8) |
| Libya | 0 (0 to 0.01) | 4.54 (1.63 to 8.98) | 10.7 (6.3 to 16.8) | 2 (0.9 to 3.7) | 5 (1.6 to 10.7) | 0 (0 to 0) | 1.8 (0.7 to 3.4) |
| Lithuania | 4.97 (0.15 to 13.61) | 9.37 (3.97 to 15.4) | 78.4 (45 to 127.4) | 20.9 (10 to 37.3) | 16.7 (7.1 to 32) | 0 (0 to 0.1) | 9.4 (4.3 to 16.1) |
| Luxembourg | 4.55 (0.18 to 10.42) | 5.2 (2.05 to 9.3) | 57.5 (46.6 to 68.9) | 13.5 (6.7 to 23.4) | 52.3 (24.1 to 81.3) | 0 (0 to 0) | 30.1 (18.4 to 44.1) |
| Malaysia | 0.13 (0.01 to 0.37) | 2.06 (0.85 to 3.86) | 31.7 (23.2 to 41.1) | 12.4 (6.7 to 19.1) | 14.2 (6.3 to 21.8) | 0 (0 to 0) | 7.8 (4.3 to 12.6) |
| Maldives | 0.07 (0 to 0.25) | 4.52 (1.85 to 8.48) | 15.5 (10.3 to 23) | 5.3 (2.9 to 8.8) | 5.7 (2.2 to 10.6) | 0 (0 to 0.1) | 3.6 (1.7 to 6) |
| Malta | 1.71 (0.06 to 4.18) | 2.84 (1.12 to 5.19) | 29.6 (19.6 to 43.5) | 5 (2.5 to 9) | 2.6 (1 to 4.8) | 0 (0 to 0) | 3.7 (2 to 5.8) |
| Marshall Islands | 1.04 (0.04 to 2.59) | 8 (3.06 to 14.73) | 31.8 (23.2 to 42.7) | 12.5 (7 to 20.1) | 26.2 (11.3 to 42.6) | 0 (0 to 0) | 12.8 (7.1 to 20.4) |
| Mauritania | 0.01 (0 to 0.04) | 3.3 (1.14 to 6.69) | 22.3 (11.7 to 36.2) | 15 (7.7 to 24.6) | 8.6 (3.3 to 16.5) | 0 (0 to 0) | 2.5 (1.1 to 4.4) |
| Mauritius | 1.94 (0.09 to 4.62) | 4.68 (2.02 to 8.23) | 11.3 (5.9 to 18.8) | 10.8 (5.2 to 19.8) | 5.4 (1.8 to 10.9) | 0 (0 to 0) | 1.1 (0.5 to 2.1) |
| Mexico | 1.41 (0.06 to 3.56) | 2.64 (1.02 to 4.62) | 23.4 (17.4 to 30.2) | 11.4 (7 to 16.3) | 10.4 (4 to 17.5) | 0.1 (0 to 0.1) | 5.5 (3.2 to 8.4) |
| Mongolia | 0.68 (0.02 to 1.87) | 1.91 (0.8 to 3.43) | 6.6 (5 to 8.7) | 4.6 (2.8 to 6.3) | 29.4 (13.4 to 43.8) | 0.2 (0 to 0.3) | 4.7 (2.6 to 7.4) |
| Montenegro | 4.5 (0.14 to 11.07) | 11.81 (4.72 to 22.13) | 18.3 (10.7 to 28.1) | 8.4 (4.3 to 13.6) | 2.3 (0.9 to 4.4) | 0 (0 to 0) | 0.7 (0.3 to 1.2) |
| Morocco | 0.01 (0 to 0.03) | 1.09 (0.34 to 2.39) | 51.3 (28.4 to 91.9) | 21.4 (9.6 to 38.1) | 46.4 (17.9 to 84.6) | 0 (0 to 0.1) | 22.4 (10.4 to 39.3) |
| Myanmar | 0.37 (0.01 to 1.1) | 2.24 (0.88 to 4.53) | 16.6 (10.3 to 24.6) | 3.8 (2 to 6.4) | 16.7 (6.8 to 29.6) | 0.1 (0 to 0.1) | 8.8 (4.8 to 14.4) |
| Namibia | 4.58 (0.17 to 9.57) | 2.37 (0.94 to 4.5) | 65.9 (44.3 to 97.7) | 22.7 (11.3 to 36.4) | 25.6 (10.5 to 42.9) | 0 (0 to 0) | 19.9 (10.9 to 31.7) |
| Nepal | 0.17 (0 to 0.55) | 1.24 (0.49 to 2.58) | 12.6 (6.7 to 21.3) | 6.9 (3 to 12.6) | 1.1 (0.4 to 2.1) | 0 (0 to 0) | 0.4 (0.2 to 0.8) |
| Netherlands | 4.74 (0.17 to 11.19) | 7.03 (3.18 to 11.68) | 12.2 (6.7 to 19.5) | 3.3 (1.5 to 6.1) | 1.6 (0.6 to 3.4) | 0 (0 to 0) | 1.3 (0.6 to 2.2) |
| New Zealand | 4.31 (0.14 to 11.28) | 3.85 (1.67 to 6.69) | 4.2 (2.3 to 7.2) | 2.5 (1.2 to 4.6) | 5.4 (1.8 to 10.8) | 0 (0 to 0) | 0.7 (0.3 to 1.3) |
| Nicaragua | 1 (0.03 to 3.02) | 2.41 (0.87 to 4.5) | 27.4 (14.4 to 46.6) | 11.6 (5.4 to 21) | 13.7 (5 to 26) | 0 (0 to 0.1) | 2.5 (1 to 4.5) |
| Nigeria | 2.43 (0.08 to 6.85) | 3.1 (1.05 to 6.44) | 7.8 (4.2 to 13) | 2.9 (1.4 to 5.1) | 1.8 (0.7 to 3.9) | 0 (0 to 0) | 1.1 (0.5 to 2.3) |
| Northern Mariana Islands | 0.37 (0.01 to 1.24) | 4.62 (1.59 to 10.35) | 35.3 (27.9 to 43.5) | 12.6 (7.7 to 18.9) | 28.5 (12.5 to 43) | 0 (0 to 0.1) | 15.8 (8.8 to 24) |
| Norway | 3.65 (0.13 to 9.33) | 3.25 (1.22 to 5.84) | 19.6 (14.7 to 24.8) | 10.7 (5.8 to 17) | 30.5 (12.9 to 44.7) | 0 (0 to 0.1) | 8.6 (4.4 to 13.5) |
| Oman | 0.05 (0 to 0.16) | 1.58 (0.63 to 3.28) | 3.7 (2 to 6) | 1.9 (1 to 3.4) | 10.6 (3.9 to 20) | 0.1 (0 to 0.1) | 2.6 (1.2 to 4.9) |
| Pakistan | 0.03 (0 to 0.07) | 3.01 (1.21 to 5.72) | 2.3 (1.1 to 4.1) | 3.3 (1.4 to 6.6) | 4.2 (1.5 to 8.8) | 0 (0 to 0) | 0.4 (0.2 to 0.8) |
| Palestine | 0.07 (0 to 0.22) | 6.35 (2.55 to 11.67) | 66.6 (48.6 to 89.3) | 21.8 (10.9 to 35.1) | 15.7 (6.6 to 27) | 0 (0 to 0) | 12.2 (6.6 to 19.2) |
| Panama | 2.2 (0.09 to 5.72) | 2.05 (0.82 to 3.84) | 20.2 (11.9 to 31.7) | 10.6 (5.6 to 17) | 18.2 (7.4 to 32.2) | 0 (0 to 0.1) | 4.5 (2.2 to 7.8) |
| Papua New Guinea | 0.25 (0.01 to 0.78) | 2.88 (1.13 to 5.87) | 14.9 (11 to 19.8) | 11.8 (6.5 to 17.8) | 18.6 (8.1 to 28.1) | 0 (0 to 0) | 6 (3.1 to 9.6) |
| Paraguay | 3.73 (0.14 to 9.22) | 7.77 (2.83 to 14.88) | 18.2 (10.6 to 32) | 11.2 (5.7 to 19.6) | 5.3 (2.2 to 10) | 0 (0 to 0) | 1.6 (0.7 to 2.9) |
| Peru | 2.13 (0.07 to 5.42) | 2.66 (0.94 to 5.6) | 19.6 (10.6 to 30.6) | 6.5 (3.1 to 11.8) | 4.8 (1.7 to 9.7) | 0 (0 to 0.1) | 3 (1.4 to 5.5) |
| Philippines | 1.9 (0.07 to 4.16) | 4.29 (1.73 to 8.11) | 6.7 (3.7 to 10.5) | 3.1 (1.5 to 5.4) | 3.7 (1.5 to 6.4) | 0 (0 to 0) | 1.5 (0.7 to 2.7) |
| Plurinational State of Bolivia | 3.25 (0.11 to 8.74) | 4.81 (1.79 to 9.4) | 54.1 (36.7 to 84.3) | 17.4 (8.7 to 29.4) | 11.8 (5 to 20.4) | 0 (0 to 0.1) | 6.7 (3.3 to 11.1) |
| Poland | 4.16 (0.15 to 10.43) | 7.73 (3.44 to 13.07) | 3.2 (2.1 to 4.7) | 3.5 (2.1 to 5.1) | 17.5 (7.4 to 27.8) | 0.1 (0 to 0.2) | 2.8 (1.5 to 4.9) |
| Portugal | 4.96 (0.17 to 11.71) | 4.49 (1.92 to 7.87) | 6.7 (3.1 to 11.9) | 3 (1.2 to 5.9) | 2.7 (0.8 to 5.9) | 0 (0 to 0) | 0.7 (0.2 to 1.3) |
| Principality of Monaco | 5.09 (0 to 14.83) | 10.11 (3.99 to 20.45) | 18.9 (13.1 to 27.1) | 7.5 (3.5 to 12.6) | 18 (5.9 to 32.1) | 0.1 (0 to 0.2) | 8.5 (3.8 to 14.6) |
| Puerto Rico | 1.9 (0.06 to 4.7) | 4.07 (1.64 to 7.24) | 4.5 (3 to 6.4) | 2.7 (1.6 to 4.7) | 20.3 (8.6 to 32.8) | 0.1 (0 to 0.2) | 2.2 (1 to 4.2) |
| Qatar | 0.11 (0 to 0.39) | 2.43 (0.88 to 5.36) | 11.2 (7.6 to 15.7) | 2.1 (1.1 to 3.5) | 4.4 (1.6 to 8.3) | 0 (0 to 0.1) | 5.7 (3.2 to 9.2) |
| Republic of Cabo Verde | 5.31 (0.17 to 12.31) | 2.8 (1.11 to 5.5) | 81 (63.9 to 98.2) | 29.2 (17.2 to 44) | 39.6 (17.4 to 59.2) | 0 (0 to 0) | 22.9 (14 to 33.4) |
| Republic of Korea | 1.02 (0.03 to 2.52) | 2.1 (0.88 to 4.12) | 33.9 (26.4 to 42.2) | 17.4 (9.7 to 25.7) | 21 (9.7 to 32.2) | 0 (0 to 0) | 9.5 (5.6 to 14.3) |
| Republic of Moldova | 2.23 (0.06 to 6.97) | 7.47 (2.99 to 13.09) | 14.3 (10.9 to 17.9) | 8.2 (5.2 to 11.8) | 19.2 (8.4 to 29) | 0 (0 to 0) | 5.1 (2.7 to 8.3) |
| Republic of Nauru | 1.42 (0.05 to 3.48) | 6.49 (2.42 to 12.04) | 10.2 (5.7 to 16.6) | 6.4 (3.1 to 10.3) | 6.1 (2.8 to 10.5) | 0 (0 to 0) | 1.8 (0.9 to 3.2) |
| Republic of Palau | 0.87 (0.03 to 2.74) | 7.28 (2.86 to 14.72) | 20.5 (16 to 28.5) | 7.1 (4.3 to 10.5) | 7.1 (3 to 12) | 0 (0 to 0) | 8.2 (4.6 to 12.8) |
| Republic of San Marino | 3.37 (0.09 to 8.59) | 4.9 (1.85 to 10.2) | 57.5 (44.4 to 71.5) | 12.2 (6.8 to 20.5) | 26.1 (11 to 42.8) | 0 (0 to 0) | 20.1 (12.4 to 30.1) |
| Romania | 3.72 (0.13 to 9.36) | 4.35 (1.93 to 7.57) | 65.4 (52.5 to 78.4) | 26.7 (15.1 to 40.1) | 26.6 (11 to 40.8) | 0 (0 to 0) | 17.8 (10.5 to 25.8) |
| Russian Federation | 2.63 (0.07 to 8.11) | 8.57 (3.91 to 14.35) | 52.1 (41.8 to 62.3) | 9.1 (5 to 15.4) | 35 (15.2 to 56.6) | 0 (0 to 0) | 29.4 (18.7 to 40.6) |
| Saint Kitts and Nevis | 4.36 (0.13 to 13.55) | 9.5 (3.33 to 20.56) | 9.5 (6.3 to 14.1) | 6 (3.4 to 10.1) | 11.9 (4.5 to 20.3) | 0.1 (0 to 0.1) | 2.9 (1.4 to 5.4) |
| Saint Lucia | 7.01 (0.25 to 21.18) | 13.69 (5.1 to 27.75) | 15.8 (10.8 to 22.7) | 6.1 (3.4 to 10.2) | 8.8 (3.7 to 14.3) | 0 (0 to 0.1) | 3.4 (1.8 to 5.9) |
| Saint Vincent and the Grenadines | 9.31 (0.34 to 25.14) | 12.28 (4.8 to 23.54) | 10 (6.5 to 14.4) | 4.1 (2.2 to 7.1) | 5.6 (2.1 to 10) | 0 (0 to 0.1) | 1.9 (0.9 to 3.3) |
| Samoa | 0.25 (0.01 to 0.78) | 4 (1.16 to 8.66) | 14.4 (7.8 to 23.2) | 5 (2.4 to 9) | 2.2 (0.9 to 4.4) | 0 (0 to 0) | 1 (0.5 to 2.1) |
| Sao Tome and Principe | 4 (0.13 to 10.27) | 1.71 (0.62 to 3.45) | 40.9 (23.2 to 66) | 17.4 (8.8 to 30.9) | 13.9 (5.4 to 26.4) | 0 (0 to 0) | 7.5 (3.3 to 14.4) |
| Saudi Arabia | 0.01 (0 to 0.04) | 1.93 (0.68 to 4.36) | 17.3 (8.6 to 30.2) | 13.3 (6 to 24.6) | 3 (1 to 6) | 0 (0 to 0) | 0.5 (0.2 to 1) |
| Senegal | 0.35 (0.01 to 0.87) | 2.6 (0.88 to 5.52) | 17.1 (10.6 to 27.8) | 15.6 (8.4 to 24.4) | 11.1 (4.7 to 20) | 0 (0 to 0.1) | 3 (1.5 to 5) |
| Serbia | 3.03 (0.11 to 7.5) | 6.68 (2.58 to 12.59) | 6.6 (3.5 to 10.4) | 5.8 (3.1 to 10.5) | 2.4 (0.8 to 4.9) | 0 (0 to 0) | 0.8 (0.3 to 1.6) |
| Seychelles | 11.16 (0.38 to 25.32) | 15.7 (5.75 to 29.46) | 76 (55.9 to 99.2) | 25.1 (13.1 to 45) | 29.7 (12.7 to 50.6) | 0 (0 to 0) | 20 (9.7 to 33) |
| Singapore | 0.56 (0.02 to 1.34) | 1.17 (0.46 to 2.21) | 46.2 (29.3 to 70.1) | 15.5 (8.2 to 26.1) | 4.2 (1.6 to 8) | 0 (0 to 0.1) | 3.3 (1.6 to 5.4) |
| Slovakia | 4.59 (0.15 to 10.74) | 6.86 (3.14 to 12.49) | 8.4 (5.9 to 11.2) | 5.2 (2.9 to 8.3) | 10.1 (4.4 to 15.6) | 0 (0 to 0) | 4.7 (2.5 to 7.5) |
| Slovenia | 2.31 (0.08 to 7.09) | 9.16 (3.82 to 17.54) | 54.9 (42.7 to 67) | 23.1 (13.4 to 33.7) | 41.4 (18.3 to 66.2) | 0 (0 to 0.1) | 24.2 (13.2 to 38.5) |
| Socialist Republic of Viet Nam | 0.44 (0.01 to 1.07) | 1.35 (0.43 to 2.73) | 42 (32.8 to 53.2) | 22.4 (13.4 to 32.8) | 32 (13.1 to 48.9) | 0 (0 to 0) | 17.4 (10 to 26.9) |
| Solomon Islands | 0.21 (0 to 1.05) | 14.43 (5.74 to 27.11) | 31.4 (15.8 to 51.8) | 5.3 (2.2 to 9.9) | 5.3 (1.6 to 11.8) | 0 (0 to 0) | 2.8 (1.1 to 5.2) |
| South Africa | 5.48 (0.22 to 11.65) | 5.6 (2.31 to 10.25) | 19.8 (13.1 to 29.2) | 8.6 (5 to 13.8) | 8 (3.1 to 14.4) | 0 (0 to 0) | 2.7 (1.4 to 4.5) |
| Spain | 2.53 (0.08 to 6.32) | 5.29 (2.27 to 9.26) | 60.3 (48.4 to 74) | 18.1 (11.1 to 26.3) | 28.8 (12.5 to 43.8) | 0 (0 to 0) | 17.3 (10.2 to 26) |
| Sri Lanka | 1.06 (0.05 to 2.36) | 1.26 (0.48 to 2.34) | 13.8 (9.8 to 19) | 5 (2.8 to 8) | 3.5 (1.3 to 6.5) | 0 (0 to 0.1) | 1.8 (1 to 3) |
| Suriname | 2.89 (0.11 to 8.22) | 9.3 (3.67 to 18.49) | 15.4 (9.3 to 23.2) | 5.8 (2.9 to 9.9) | 8.5 (3.2 to 15.8) | 0 (0 to 0.1) | 4.9 (2.3 to 8.7) |
| Sweden | 4.04 (0.14 to 10.58) | 9.1 (3.81 to 17.14) | 19.4 (15 to 24.8) | 9.2 (5.5 to 14.1) | 21.7 (9.6 to 33.7) | 0 (0 to 0) | 10.9 (5.8 to 17.7) |
| Switzerland | 3.88 (0.14 to 8.83) | 5.38 (2.29 to 9.77) | 27.7 (22.1 to 34.5) | 9.5 (5.9 to 13.9) | 15.9 (6.9 to 23.4) | 0 (0 to 0) | 9.1 (5.2 to 14.2) |
| Taiwan (Province of China) | 0.98 (0.04 to 2.28) | 2.92 (1.23 to 5.15) | 38.9 (29.4 to 50.2) | 14.5 (8.1 to 21.5) | 19.5 (8.2 to 32.3) | 0.1 (0 to 0.3) | 14 (7.8 to 21.4) |
| Tajikistan | 0.04 (0 to 0.17) | 1.6 (0.53 to 3.69) | 6.2 (3.3 to 10.5) | 3 (1.4 to 5.4) | 17.2 (7.6 to 31.5) | 0 (0 to 0.1) | 5.9 (2.7 to 11.6) |
| Thailand | 2.26 (0.08 to 5.15) | 3.74 (1.48 to 7.38) | 26.2 (20.1 to 35.7) | 5.8 (3.4 to 9.2) | 7.3 (2.7 to 12.6) | 0.1 (0 to 0.1) | 5.4 (2.8 to 10.4) |
| The former Yugoslav Republic of Macedonia | 2.31 (0.07 to 5.48) | 6.64 (2.87 to 11.79) | 10.2 (5.3 to 17.7) | 1.1 (0.4 to 2.2) | 1.2 (0.4 to 2.6) | 0 (0 to 0) | 2.1 (0.9 to 3.8) |
| Timor-Leste | 0.29 (0.01 to 0.92) | 2.69 (1 to 5.46) | 16.5 (9.3 to 26.2) | 6.2 (2.9 to 10.9) | 6.8 (2.6 to 13.8) | 0 (0 to 0) | 2.2 (0.9 to 4) |
| Tonga | 0.75 (0.03 to 1.86) | 7.63 (2.95 to 15.06) | 17.4 (12.7 to 24.4) | 7.5 (4 to 12.1) | 14.7 (6.6 to 23.4) | 0.1 (0 to 0.1) | 5.3 (2.9 to 8.5) |
| Trinidad and Tobago | 5.65 (0.22 to 16.22) | 17.19 (6.98 to 32.82) | 80.8 (52.7 to 114) | 22.1 (11.7 to 37.1) | 14.4 (5.9 to 24.7) | 0 (0 to 0.1) | 11 (6 to 18.1) |
| Tunisia | 0.25 (0.01 to 0.81) | 9.64 (3.58 to 20.24) | 60.9 (43.9 to 81.6) | 18.5 (9.9 to 29.4) | 21.2 (7.7 to 34.2) | 0.1 (0 to 0.2) | 12.4 (6.6 to 19.8) |
| Turkey | 0.27 (0.01 to 0.87) | 5.3 (2.21 to 9.83) | 13.4 (9.5 to 18.4) | 4.1 (2.2 to 6.9) | 27.6 (11.5 to 46.1) | 0.1 (0 to 0.2) | 13 (7.2 to 21.9) |
| Turkmenistan | 0.19 (0 to 0.63) | 1.63 (0.64 to 3.21) | 26.3 (14 to 42.4) | 7.4 (3.7 to 12.9) | 13 (5.1 to 25) | 0 (0 to 0.1) | 3 (1.3 to 5.5) |
| Tuvalu | 1.13 (0.04 to 2.78) | 7.92 (3.01 to 15.1) | 54.5 (39.4 to 69.3) | 10 (5.2 to 17.4) | 37.6 (16.9 to 61.5) | 0 (0 to 0) | 26.2 (16.4 to 38.4) |
| Ukraine | 2.34 (0.06 to 7.87) | 6.52 (2.51 to 11.43) | 6.1 (3.2 to 9.9) | 7.5 (3.8 to 12.5) | 4 (1.8 to 7.3) | 0 (0 to 0) | 0.7 (0.3 to 1.2) |
| United Arab Emirates | 0.22 (0.01 to 0.7) | 1.29 (0.46 to 2.68) | 34.1 (27 to 40.8) | 18 (11.3 to 24.9) | 31.6 (14.4 to 48.7) | 0 (0 to 0) | 14.1 (8.4 to 21.3) |
| United Kingdom of Great Britain and Northern Ireland | 5.45 (0.21 to 12.74) | 6.14 (2.77 to 11.2) | 6.7 (3.9 to 11.5) | 1.3 (0.6 to 2.4) | 1.2 (0.4 to 2.4) | 0 (0 to 0) | 0.4 (0.2 to 0.7) |
| United Republic of Tanzania | 2.78 (0.09 to 7.04) | 6.97 (2.82 to 13.92) | 28.2 (22.9 to 33.5) | 12.9 (7.7 to 18.9) | 29.9 (12.9 to 44.5) | 0 (0 to 0) | 12.6 (7.2 to 19.3) |
| United States of America | 2.74 (0.09 to 7.55) | 6.28 (2.71 to 11.28) | 6.3 (4.4 to 8.4) | 4.2 (2 to 7.7) | 30.4 (12.9 to 48.5) | 0.1 (0 to 0.2) | 5.2 (2.8 to 8.5) |
| United States Virgin Islands | 2.71 (0 to 11.37) | 5.89 (2.16 to 10.65) | 57.8 (44.9 to 74.4) | 16.7 (9.7 to 26.7) | 67.8 (30.2 to 102.4) | 0.3 (0.1 to 0.6) | 32.4 (18.1 to 47.5) |
| Uruguay | 5.33 (0.18 to 13.06) | 8.66 (3.65 to 15.26) | 13.2 (9.4 to 18) | 4.4 (2.6 to 7.2) | 12.8 (5.5 to 21.2) | 0 (0 to 0.1) | 5.1 (3 to 8) |
| Uzbekistan | 0.27 (0.01 to 0.8) | 1.07 (0.48 to 1.96) | 7.6 (4 to 11.8) | 4.5 (1.9 to 7.8) | 4.6 (1.5 to 9.4) | 0 (0 to 0) | 0.9 (0.4 to 1.8) |
| Vanuatu | 0.19 (0 to 0.63) | 2.86 (1.13 to 5.49) | 20.9 (12.5 to 33.3) | 2.1 (1 to 4) | 1.2 (0.4 to 2.5) | 0 (0 to 0) | 2.9 (1.3 to 5.3) |
| Zambia | 5.8 (0.12 to 14.39) | 4.35 (1.77 to 8.09) | 8.9 (4.9 to 14.4) | 5.5 (2.9 to 9.7) | 2.1 (0.7 to 4.5) | 0 (0 to 0) | 0.3 (0.1 to 0.5) |
| Zimbabwe | 4.55 (0.13 to 9.87) | 3.95 (1.52 to 7.63) | 27.5 (16.1 to 43.3) | 7.5 (3.5 to 14.3) | 1.8 (0.6 to 3.5) | 0 (0 to 0) | 1.1 (0.5 to 1.9) |

## Table S8 Age-standardized DALYs rate of average annual percent change attributable to risk factors for male genitourinary cancers from 1990 to 2023

| **Locations** | Prostate cancer, AAPC for ASDR, 95% CI | | Male bladder cancer, AAPC for ASDR, 95% CI | | Male kidney cancer, AAPC for ASDR, 95% CI | | |
| --- | --- | --- | --- | --- | --- | --- | --- |
|  | Alcohol use | Smoking | Smoking | High fasting plasma glucose | High body-mass index | Occupational exposure to trichloroethylene | Smoking |
| World Bank High Income | -2.3 (-3.1 to -1.5) | -0.7 (-1.9 to 0.5) | -2.1 (-2.6 to -1.7) | -0.7 (-1.6 to 0.1) | 0 (-0.7 to 0.6) | 0 (-17.9 to 21.9) | -1.6 (-2.3 to -0.8) |
| World Bank Upper Middle Income | -1 (-2.2 to 0.2) | -0.7 (-2.9 to 1.6) | -1.7 (-2.2 to -1.1) | -1.1 (-2.3 to 0.2) | 3.3 (0.8 to 5.9) | 1.2 (-19.9 to 27.8) | -0.1 (-2.4 to 2.2) |
| World Bank Lower Middle Income | -0.8 (-2.4 to 0.8) | 0.2 (-3 to 3.5) | -1.4 (-2.2 to -0.5) | 0.3 (-1.4 to 2.1) | 1.5 (0.3 to 2.8) | 0.9 (-13.9 to 18.2) | -0.1 (-1.3 to 1.1) |
| Albania | -1 (-1.8 to -0.3) | 3.3 (0.7 to 6) | 0.6 (0 to 1.2) | 0.7 (-2.5 to 4) | 1.8 (0.8 to 2.7) | 0 (0 to 0) | 0.8 (0 to 1.7) |
| Algeria | -0.3 (-2.6 to 2.1) | 2.8 (-16.5 to 26.5) | -0.6 (-1.3 to 0.1) | 0.9 (-0.6 to 2.3) | 6.4 (6 to 6.8) | 0 (0 to 0) | 0.2 (-2.1 to 2.7) |
| American Samoa | -0.5 (-1.2 to 0.2) | 2.3 (0 to 4.7) | -0.4 (-1.2 to 0.5) | 7.8 (5 to 10.6) | 0.6 (-0.5 to 1.6) | -0.1 (-18.4 to 22.4) | -0.8 (-2.8 to 1.2) |
| Andorra | -0.8 (-1.8 to 0.2) | 0.6 (-0.8 to 2.1) | -1.7 (-2.1 to -1.2) | -0.5 (-1.4 to 0.4) | -0.4 (-1.1 to 0.3) | -1.5 (-20.5 to 22.1) | -2.2 (-3 to -1.3) |
| Angola | -0.4 (-1.3 to 0.4) | 3 (2 to 4) | 0.4 (-0.5 to 1.3) | 1.5 (0.1 to 2.9) | 7.7 (7.1 to 8.3) | 1.1 (-22.5 to 31.9) | 0.5 (-2.1 to 3.2) |
| Antigua and Barbuda | -0.7 (-1.2 to -0.1) | 14.5 (11.3 to 17.8) | -0.6 (-1.5 to 0.4) | 0 (-1.6 to 1.6) | 0.7 (-0.5 to 1.9) | -0.6 (-14.2 to 15.2) | -0.5 (-2.1 to 1.1) |
| Argentina | -1.5 (-2.4 to -0.6) | -1.1 (-2.1 to -0.2) | -2.3 (-2.8 to -1.8) | -1.6 (-2.5 to -0.6) | 1.4 (0.9 to 1.9) | 0.6 (-6.4 to 8.1) | -0.1 (-0.8 to 0.7) |
| Armenia | 0.3 (-0.5 to 1.1) | -0.3 (-3.9 to 3.3) | -1.2 (-1.6 to -0.8) | -0.8 (-1.6 to 0.1) | 0.5 (-0.2 to 1.2) | 0.1 (0.1 to 0.1) | -0.1 (-0.8 to 0.6) |
| Australia | -3 (-3.8 to -2.2) | -0.6 (-1.6 to 0.5) | -3.2 (-3.9 to -2.6) | -0.9 (-2 to 0.3) | 0.1 (-0.6 to 0.8) | -1.6 (-20.5 to 21.8) | -28.6 (-30.1 to -27.2) |
| Austria | -1.8 (-2.6 to -1.1) | -1.2 (-2.2 to -0.2) | -90.5 (-97.7 to -83.4) | -0.3 (-1.4 to 0.8) | -1.2 (-1.8 to -0.5) | -1.2 (-19.2 to 20.7) | -1.9 (-2.7 to -1.1) |
| Azerbaijan | -0.4 (-1.4 to 0.5) | 2.1 (-0.5 to 4.7) | -0.8 (-1.3 to -0.3) | -0.3 (-1.6 to 0.9) | 0.3 (-0.5 to 1) | -1.5 (-12.1 to 10.4) | -0.2 (-1 to 0.5) |
| Bahrain | -0.3 (-5.7 to 5) | 0.1 (-0.7 to 0.9) | 0.3 (-0.9 to 1.4) | 1.3 (-0.3 to 2.9) | 1.6 (0.8 to 2.5) | 0.1 (0.1 to 0.1) | 0.5 (-1.3 to 2.4) |
| Bangladesh | -1.1 (-2.1 to 0) | -1.3 (-5.4 to 3.1) | -2.3 (-2.7 to -1.8) | -1 (-1.8 to -0.2) | 0.3 (-0.8 to 1.3) | -1 (-15.7 to 16.3) | -1.4 (-2.7 to -0.1) |
| Barbados | 2.9 (1.9 to 4) | -2.9 (-27.2 to 29.3) | -0.1 (-0.9 to 0.7) | 1.9 (0 to 3.9) | 3.4 (0.5 to 6.4) | 0.1 (0.1 to 0.1) | 0.1 (-1.8 to 2.1) |
| Belarus | -0.6 (-1.3 to 0.1) | -1.1 (-2.6 to 0.3) | -1 (-1.9 to 0) | 0.6 (-0.6 to 1.9) | 1.4 (0.6 to 2.2) | 0.9 (-10.4 to 13.6) | -0.6 (-2 to 0.9) |
| Belgium | 2 (1.4 to 2.7) | 1.7 (0.5 to 2.8) | 0.2 (-1.1 to 1.5) | 1 (-0.3 to 2.3) | 3.6 (2.3 to 4.9) | 2.1 (-18.4 to 27.8) | 2.3 (1 to 3.7) |
| Belize | -4 (-4.7 to -3.3) | -1.8 (-2.9 to -0.8) | -3.2 (-3.6 to -2.7) | -1 (-1.9 to -0.2) | -0.2 (-0.8 to 0.5) | 0 (0 to 0) | -2 (-2.8 to -1.3) |
| Benin | 0.4 (-0.4 to 1.2) | 18.2 (14.5 to 21.9) | 0.2 (-0.8 to 1.2) | 4.4 (3.7 to 5.1) | 2.5 (1.2 to 3.8) | 2 (-12.6 to 19) | 0.6 (-1.2 to 2.4) |
| Bermuda | -1.2 (-2.5 to 0) | -1.4 (-2.8 to 0) | -6.2 (-6.8 to -5.7) | 0.2 (-2.2 to 2.7) | 2.9 (1 to 4.7) | 1.3 (-15.9 to 21.9) | -0.4 (-3.9 to 3.3) |
| Bhutan | -67.2 (-76.9 to -57.4) | -0.8 (-1.7 to 0) | -2.2 (-2.7 to -1.7) | -0.1 (-1.1 to 1) | -0.2 (-1.1 to 0.7) | -1.4 (-11.8 to 10.2) | -22.4 (-23.9 to -20.9) |
| Bosnia and Herzegovina | -1.2 (-3.4 to 1) | -1.6 (-6 to 2.9) | -1.7 (-3.1 to -0.3) | -1.1 (-3.3 to 1.1) | 7.2 (7 to 7.4) | 0 (0 to 0) | 0.4 (-2.5 to 3.3) |
| Botswana | 17.9 (14.5 to 21.4) | 2 (0.5 to 3.5) | 2.5 (1.2 to 3.8) | 2.8 (1.1 to 4.4) | 2.7 (1.7 to 3.7) | 2.4 (-9.2 to 15.5) | 1.6 (-0.7 to 4) |
| Brazil | 0.8 (0 to 1.6) | 1.5 (0 to 3) | 0.6 (0.2 to 1) | 23.2 (21 to 25.3) | 1.8 (1 to 2.6) | 0 (0 to 0) | 28.2 (17.9 to 38.5) |
| Brunei Darussalam | 0.7 (-0.8 to 2.2) | 0 (-0.9 to 0.9) | 0.4 (-1.5 to 2.3) | 0.9 (-3.4 to 5.3) | 3.4 (0.5 to 6.2) | 0 (0 to 0) | 0.1 (-3 to 3.4) |
| Bulgaria | -1.5 (-2.3 to -0.6) | 0.2 (-1.3 to 1.7) | -2.1 (-2.8 to -1.4) | -0.4 (-1.6 to 0.8) | 3.1 (2.1 to 4.2) | 0.1 (0.1 to 0.1) | -0.6 (-1.9 to 0.8) |
| Cambodia | -1.8 (-2.9 to -0.6) | -1.1 (-7.9 to 6.2) | -46.3 (-52.7 to -40) | -0.9 (-2.1 to 0.4) | 1.7 (0.7 to 2.6) | 0 (0 to 0) | -1.5 (-2.5 to -0.5) |
| Cameroon | 0.5 (-0.3 to 1.3) | 1.2 (0 to 2.4) | 0.5 (0.1 to 0.9) | 0.8 (0 to 1.6) | 3.8 (2.9 to 4.6) | 2.9 (-22.3 to 36.2) | 3.5 (2.6 to 4.4) |
| Canada | 0.5 (-0.9 to 1.9) | 1.2 (0.2 to 2.2) | -0.2 (-1.5 to 1) | 0.2 (-1.3 to 1.7) | 6.4 (3.9 to 9) | 3.2 (-33.5 to 60.2) | 4 (-0.4 to 8.6) |
| Chile | -0.8 (-2.1 to 0.5) | 7.6 (3 to 12.3) | -0.4 (-1.4 to 0.5) | -0.3 (-4 to 3.5) | 3 (-0.4 to 6.4) | 1.8 (-18.5 to 27.2) | -0.4 (-2.1 to 1.3) |
| China | 0.3 (-1 to 1.6) | 1.5 (-0.1 to 3) | -1.1 (-2.1 to 0) | 1.2 (-0.1 to 2.5) | 3.2 (1.5 to 5.1) | 0 (0 to 0.1) | 1.1 (-2.5 to 4.8) |
| Colombia | -70.6 (-77.4 to -63.7) | -1.5 (-3 to 0) | -2.1 (-2.7 to -1.6) | 0.1 (-0.9 to 1.1) | -0.3 (-0.9 to 0.4) | -1.6 (-20.5 to 21.8) | -2 (-2.8 to -1.2) |
| Commonwealth of the Bahamas | -1.4 (-2.4 to -0.3) | 0.7 (-0.4 to 1.8) | -1.8 (-2.5 to -1) | 0.2 (-1.1 to 1.5) | 1.1 (0.5 to 1.7) | 0.7 (-7.7 to 9.9) | -0.4 (-1.4 to 0.5) |
| Comoros | -0.7 (-2.4 to 0.9) | -1.3 (-4.4 to 1.8) | -75.1 (-84.8 to -65.3) | -1.8 (-3 to -0.6) | 2 (0.2 to 3.8) | 1.2 (-15.2 to 20.7) | 0.6 (-0.8 to 1.9) |
| Congo | -3.3 (-4.6 to -2) | -1.2 (-3.4 to 0.9) | -2.8 (-4 to -1.6) | -0.3 (-2.2 to 1.7) | 2.6 (1.3 to 3.9) | 2.1 (-12.4 to 19.1) | -2.6 (-3 to -2.3) |
| Costa Rica | -3.9 (-4.6 to -3.3) | 3.3 (-7.2 to 15) | -1 (-2.5 to 0.4) | 1 (-2.7 to 4.8) | 4.9 (4.5 to 5.3) | 0.4 (-28.4 to 40.8) | -0.3 (-0.4 to -0.2) |
| Cote d Ivoire | 26.5 (23.9 to 29.2) | 4.2 (2.9 to 5.4) | 28.7 (25.7 to 31.7) | 1.9 (0.5 to 3.2) | 3.3 (0.6 to 6) | -1.7 (-27.5 to 33.1) | 1.6 (-1.8 to 5.1) |
| Croatia | -1.8 (-2.7 to -0.9) | -3.4 (-5.1 to -1.7) | -1.9 (-2.8 to -1.1) | 0.7 (-0.7 to 2.1) | 2 (1 to 2.9) | 1.2 (-10.7 to 14.6) | -1.1 (-2.4 to 0.2) |
| Cuba | -0.5 (-1.2 to 0.2) | -6.8 (-9.7 to -4) | -0.2 (-0.6 to 0.2) | 0.8 (0.1 to 1.5) | 1 (0.4 to 1.6) | 1.6 (-17.9 to 25.8) | 0.3 (-0.4 to 0.9) |
| Cyprus | 21.3 (16.5 to 26) | 3 (1.7 to 4.3) | 0.4 (-0.1 to 1) | 1.3 (0.1 to 2.6) | 2 (1 to 3) | 1.5 (-11.8 to 16.8) | 0.9 (-0.3 to 2.1) |
| Czech Republic | -28 (-32.8 to -23.2) | 2.3 (0.9 to 3.8) | -41.5 (-56 to -27.1) | 13.8 (11.5 to 16) | 0.8 (0 to 1.6) | -2.1 (-25.1 to 28) | -0.7 (-1.6 to 0.3) |
| Denmark | -2.1 (-2.7 to -1.4) | -7.3 (-11.4 to -3.3) | -171 (-182.4 to -159.5) | -1.1 (-1.8 to -0.4) | -71.8 (-97.5 to -46.1) | -1.5 (-14.5 to 13.6) | -2.4 (-2.9 to -1.9) |
| Djibouti | 8.4 (6.3 to 10.5) | -0.2 (-1.6 to 1.1) | -0.7 (-3 to 1.6) | -0.8 (-3.8 to 2.2) | 3.2 (0.2 to 6.4) | 0 (0 to 0) | 1.2 (-3.4 to 5.9) |
| Dominica | -131.8 (-143.1 to -120.5) | -1.6 (-2.4 to -0.8) | -2.9 (-3.9 to -1.8) | -0.3 (-1.2 to 0.7) | 0.2 (-0.4 to 0.9) | 0.2 (-18.4 to 23) | -1.6 (-2.5 to -0.8) |
| Dominican Republic | 1.5 (0.1 to 3) | -0.8 (-6.8 to 5.6) | 6.5 (3 to 10) | 0.3 (-2.2 to 2.8) | 3.3 (-0.6 to 7.3) | 0 (0 to 0) | 1.5 (-1.9 to 5.1) |
| Ecuador | 0.7 (0.1 to 1.2) | -0.4 (-1.2 to 0.3) | 0.6 (-0.4 to 1.5) | 1.6 (0.2 to 3.1) | 1.7 (0.8 to 2.7) | 1.7 (-12.6 to 18.3) | 0.6 (-1 to 2.2) |
| Egypt | -1.9 (-2.6 to -1.1) | 1.8 (0.4 to 3.2) | -0.9 (-2 to 0.3) | 4.7 (3.8 to 5.6) | 2.4 (0.6 to 4.3) | 1.2 (-16.2 to 22.2) | -1.9 (-4 to 0.3) |
| El Salvador | -2.4 (-3.4 to -1.4) | 0.5 (-1.2 to 2.3) | -2.3 (-3.4 to -1.1) | 0.4 (-1.4 to 2.3) | 10.7 (6 to 15.3) | 0.1 (0.1 to 0.1) | -1.3 (-3.1 to 0.6) |
| Equatorial Guinea | 13 (10.9 to 15.1) | -0.9 (-36 to 53.5) | -2.8 (-3.1 to -2.4) | -1.6 (-2.5 to -0.7) | 11.1 (9 to 13.2) | 0.7 (-21.4 to 29.1) | 1.5 (-0.3 to 3.3) |
| Estonia | -0.9 (-2.2 to 0.4) | 2.2 (0.2 to 4.3) | 0.7 (-1.1 to 2.4) | 2.2 (0.1 to 4.3) | 2.7 (1.4 to 3.9) | 3 (-11.7 to 20.2) | 0.7 (-1.8 to 3.3) |
| Federated States of Micronesia | 1.2 (0.3 to 2) | 1.7 (0.9 to 2.5) | 2.8 (1.8 to 3.8) | 2.7 (1.7 to 3.7) | 4.3 (2.5 to 6.2) | 2 (-21.6 to 32.7) | 1.4 (1.3 to 1.5) |
| Fiji | 0.5 (-0.2 to 1.1) | 1.6 (0.6 to 2.7) | -1.6 (-2 to -1.2) | -5.5 (-9.4 to -1.6) | 0.8 (0.3 to 1.3) | -1.4 (-18.5 to 19.2) | -0.1 (-0.7 to 0.5) |
| Finland | -3.6 (-4.9 to -2.3) | -2.7 (-3.9 to -1.6) | -3.5 (-5.3 to -1.8) | -1.2 (-3.4 to 1) | 0.6 (-0.7 to 1.8) | 0 (0 to 0) | -1.3 (-5.2 to 2.8) |
| France | -0.3 (-1.2 to 0.7) | 3.3 (0.4 to 6.3) | 0.3 (-0.6 to 1.2) | 2.1 (0.7 to 3.5) | 0.5 (-1.6 to 2.6) | 0 (0 to 0) | -2.6 (-2.9 to -2.3) |
| Gabon | -1.7 (-2.5 to -0.9) | -0.9 (-1.9 to 0.1) | -2.5 (-3.2 to -1.9) | -0.4 (-1.4 to 0.7) | -0.9 (-1.5 to -0.3) | -1 (-19.4 to 21.6) | -2.4 (-3.2 to -1.5) |
| Georgia | -3.2 (-4 to -2.4) | -1.3 (-2.3 to -0.4) | -2.2 (-2.6 to -1.8) | -0.8 (-1.7 to 0) | 0.1 (-0.5 to 0.7) | 0 (0 to 0) | -28.3 (-30.3 to -26.4) |
| Germany | 4.3 (3 to 5.5) | 6.8 (4.8 to 8.9) | 2.4 (1 to 3.7) | 1.1 (-0.1 to 2.3) | 12.8 (12.2 to 13.4) | 0 (-21.5 to 27.4) | 2.4 (-1.6 to 6.6) |
| Ghana | 4.8 (4.1 to 5.6) | 3.7 (2 to 5.3) | 1.6 (1.2 to 2) | 1.3 (0.4 to 2.3) | 5.4 (4.5 to 6.4) | 3.8 (-8.8 to 18.2) | 5.4 (4.4 to 6.3) |
| Greece | -2.9 (-3.6 to -2.1) | -0.5 (-1.4 to 0.4) | -2.7 (-3.2 to -2.2) | -1.7 (-2.6 to -0.9) | -1.3 (-1.8 to -0.7) | -1.5 (-18.3 to 18.7) | -64.4 (-68.7 to -60.1) |
| Greenland | -2.1 (-3.4 to -0.7) | -0.1 (-2.9 to 2.8) | -9.3 (-12.3 to -6.3) | 0 (-1 to 1.1) | 5.2 (2.5 to 7.9) | 0 (0 to 0.1) | 1.9 (-4.1 to 8.3) |
| Grenada | -2.4 (-3.1 to -1.7) | -1.2 (-2.5 to 0) | -1.3 (-1.7 to -1) | -0.2 (-1 to 0.5) | 0.9 (0.2 to 1.5) | 0 (-21.5 to 27.4) | -0.5 (-1.2 to 0.2) |
| Guam | -1.4 (-2.3 to -0.5) | 0.1 (-1.6 to 1.8) | -1.7 (-2.1 to -1.3) | -0.9 (-1.8 to -0.1) | -16.3 (-25.8 to -6.7) | -1 (-17.2 to 18.4) | -1.1 (-1.7 to -0.5) |
| Guatemala | -0.6 (-1.3 to 0.1) | 1.2 (0.2 to 2.2) | -0.8 (-1.8 to 0.1) | 0.1 (-1.5 to 1.8) | 2 (0.8 to 3.3) | 1.1 (-12.6 to 16.9) | -0.5 (-2.3 to 1.2) |
| Guinea | -1.1 (-2.3 to 0.2) | -1.7 (-2.5 to -0.9) | 0.6 (-0.3 to 1.5) | 1.2 (-0.1 to 2.4) | 0.5 (-0.7 to 1.8) | 0.5 (-13.3 to 16.6) | -1.1 (-2.4 to 0.3) |
| Guyana | -10.2 (-15.1 to -5.3) | 1.8 (1.4 to 2.1) | -2.8 (-4.4 to -1.1) | 0 (-2.1 to 2.1) | 2.6 (1.2 to 4) | 1.7 (-13 to 19) | -0.9 (-3.2 to 1.5) |
| Haiti | -0.1 (-1.2 to 1) | -0.7 (-6.6 to 5.7) | -1.8 (-2.8 to -0.8) | -1.1 (-3.1 to 0.9) | 3.4 (0.2 to 6.7) | 0 (0 to 0) | 0.8 (-2.6 to 4.4) |
| Honduras | 0.8 (0.1 to 1.5) | -2.6 (-3.8 to -1.4) | 0.4 (-0.6 to 1.4) | 0.7 (-1.1 to 2.5) | 1.5 (0.3 to 2.7) | 0.5 (-13.3 to 16.5) | 0.6 (-1.1 to 2.3) |
| Hungary | 0.5 (-0.7 to 1.6) | 2 (0.9 to 3.1) | -0.4 (-1.8 to 1) | 2.6 (2.4 to 2.9) | 9.5 (8.8 to 10.2) | 0.1 (0.1 to 0.1) | -0.7 (-3.9 to 2.6) |
| Iceland | -3.2 (-4.8 to -1.6) | 0.2 (-3 to 3.5) | -1.9 (-3.9 to 0.2) | 0.5 (-2.5 to 3.7) | 1.4 (-0.6 to 3.5) | 1.5 (-19.7 to 28.2) | -1.2 (-4 to 1.6) |
| India | -2.3 (-3 to -1.5) | -0.4 (-1.4 to 0.6) | -0.9 (-1.2 to -0.5) | 0.1 (-0.6 to 0.8) | 8.3 (-4 to 20.6) | 0.6 (-15.8 to 20.3) | -1 (-1.6 to -0.5) |
| Indonesia | -2.6 (-3.3 to -1.9) | 0.6 (-0.6 to 1.9) | -2.6 (-3.1 to -2) | -0.7 (-1.6 to 0.3) | -0.2 (-0.7 to 0.4) | 0 (0 to 0) | -39.8 (-42.7 to -36.9) |
| Iraq | -1.4 (-3.4 to 0.6) | -1.6 (-6.5 to 3.5) | -1.5 (-2.5 to -0.5) | 0.4 (-1.4 to 2.3) | 5.5 (1.8 to 9.3) | 0.7 (-21.1 to 28.6) | -0.4 (-3.2 to 2.4) |
| Ireland | 1.1 (-0.2 to 2.4) | -0.2 (-14.9 to 17) | 0.3 (-0.6 to 1.2) | 0.3 (-1.9 to 2.5) | 4.2 (1.5 to 7) | 2.3 (-15.4 to 23.5) | 6.2 (5.8 to 6.6) |
| Islamic Republic of Iran | -0.2 (-1.5 to 1.1) | -4 (-13.5 to 6.6) | -0.2 (-0.9 to 0.5) | 1.1 (-0.6 to 2.9) | 2.2 (0.9 to 3.5) | 0 (0 to 0) | 0.3 (-1.4 to 2) |
| Israel | 1.6 (0.6 to 2.6) | 0.4 (-13.7 to 16.8) | 1 (0.6 to 1.3) | 2.6 (1.8 to 3.3) | 2.3 (1.4 to 3.2) | 0.1 (0.1 to 0.1) | 1 (0 to 2) |
| Italy | -4.8 (-5.6 to -4) | -1.5 (-2.6 to -0.5) | -3.5 (-4.1 to -2.9) | -0.8 (-1.9 to 0.3) | 0.3 (-0.3 to 1) | 0 (0 to 0) | -2.6 (-3.5 to -1.8) |
| Jamaica | -3.1 (-4.1 to -2) | 1.7 (-1.7 to 5.2) | -1.8 (-2.3 to -1.3) | -0.4 (-1.3 to 0.5) | 0.1 (-0.6 to 0.9) | 0 (-21.5 to 27.4) | -1.3 (-2.3 to -0.4) |
| Japan | -3.4 (-4.2 to -2.5) | -1.5 (-2.7 to -0.4) | -3.3 (-3.7 to -2.9) | -1.4 (-2.1 to -0.7) | -0.1 (-0.7 to 0.6) | -1.6 (-20.5 to 21.8) | -2.3 (-3.1 to -1.5) |
| Jordan | -0.6 (-1.2 to 0.1) | 20.1 (14.3 to 25.9) | -0.7 (-1.5 to 0.2) | 0.4 (-1 to 1.9) | 16.1 (14.6 to 17.5) | 1.3 (-13.2 to 18.2) | -1.1 (-2.7 to 0.6) |
| Kazakhstan | -1.4 (-2.7 to -0.1) | -0.9 (-2.9 to 1.2) | -1.5 (-2.1 to -0.8) | -0.2 (-1.3 to 0.9) | 0.9 (-0.3 to 2.2) | 0 (-21.5 to 27.4) | -19.7 (-21.1 to -18.3) |
| Kenya | -1.2 (-2 to -0.3) | -0.3 (-11.7 to 12.6) | -0.7 (-1.1 to -0.3) | 0.3 (-0.4 to 1.1) | -0.1 (-1 to 0.8) | 0 (0 to 0) | -0.9 (-1.9 to 0.1) |
| Kingdom of Eswatini | -0.2 (-1.3 to 1) | -0.3 (-2.5 to 1.9) | -110.7 (-132.2 to -89.1) | -1.6 (-2.7 to -0.4) | -0.7 (-1.3 to 0) | -1.3 (-10.7 to 9.2) | -29.9 (-40.7 to -19.1) |
| Kiribati | -0.3 (-1.8 to 1.3) | -0.5 (-2.1 to 1.2) | -6.6 (-8.1 to -5) | 0.2 (-3.5 to 4) | 4.5 (0.8 to 8.3) | 0.1 (0 to 0.1) | 0.7 (-3.3 to 4.9) |
| Kuwait | -0.3 (-1.9 to 1.4) | -0.4 (-8.8 to 8.8) | -0.1 (-1.9 to 1.8) | 0.2 (-3.1 to 3.5) | 1 (-0.2 to 2.3) | 3.1 (-22.9 to 37.8) | 0.3 (-1.5 to 2.1) |
| Kyrgyzstan | -1.5 (-2.6 to -0.3) | 0 (-36.8 to 58.2) | -3.1 (-3.6 to -2.5) | -1.6 (-2.4 to -0.8) | 3.5 (-1.9 to 9) | -0.1 (-16.1 to 19) | -1.3 (-2.6 to 0) |
| Lao People's Democratic Republic | -0.9 (-2.2 to 0.4) | -1.1 (-4.2 to 2) | -1.6 (-2.3 to -1) | -1.3 (-2.8 to 0.3) | 1.8 (0.8 to 2.7) | 1.3 (-12.3 to 17) | 0.8 (-0.3 to 1.9) |
| Latvia | 0.1 (-1.2 to 1.4) | 1.8 (-1.1 to 4.7) | 0.5 (-0.3 to 1.4) | 0.3 (-2.2 to 2.9) | 4.7 (4.5 to 4.9) | 1.9 (-20.8 to 31.1) | 0.4 (-1.4 to 2.3) |
| Lebanon | -0.2 (-0.8 to 0.5) | 1.5 (0.6 to 2.6) | -0.6 (-0.9 to -0.2) | 0.4 (-0.5 to 1.3) | 0.8 (0.3 to 1.3) | 0 (0 to 0) | -0.2 (-0.7 to 0.4) |
| Lesotho | 33.4 (28.3 to 38.5) | 2 (-2.6 to 6.8) | 3.1 (2.5 to 3.6) | 3.1 (2.3 to 4) | 2.7 (1.8 to 3.7) | 2.5 (-12.8 to 20.5) | 2.7 (1.5 to 3.9) |
| Libya | -2.6 (-3.7 to -1.4) | -0.8 (-1.9 to 0.2) | -1.5 (-2.5 to -0.5) | -0.2 (-2.8 to 2.4) | 3.3 (1.2 to 5.4) | 0 (0 to 0) | -1 (-3.2 to 1.4) |
| Lithuania | 0.7 (-0.5 to 2) | NA (NA to NA) | 0.3 (-0.1 to 0.7) | 1.2 (0.3 to 2) | 1.6 (0.7 to 2.5) | 0 (0 to 0) | 0.9 (-0.2 to 2.1) |
| Luxembourg | 0.2 (-0.5 to 0.9) | 2.2 (1.2 to 3.2) | -1 (-1.4 to -0.6) | 0.3 (-0.7 to 1.2) | 1.9 (1.2 to 2.5) | 0.8 (-17.4 to 23.1) | 1.1 (0.4 to 1.7) |
| Malaysia | -2.9 (-3.7 to -2.1) | -25.9 (-27.7 to -24) | -2.9 (-3.3 to -2.4) | -1.4 (-2.2 to -0.5) | -0.4 (-1.3 to 0.4) | -0.5 (-29 to 39.6) | -1.8 (-2.8 to -0.8) |
| Maldives | -1.6 (-3 to -0.1) | 0.9 (-5.6 to 8) | -1 (-1.8 to -0.2) | -4.5 (-5 to -4) | 2.2 (0.6 to 3.8) | 0.9 (-14.1 to 18.6) | -4.1 (-5.1 to -3.1) |
| Malta | 0.7 (-0.6 to 2.1) | 3.5 (-14.4 to 25.1) | 1.3 (0.6 to 2.1) | 5.6 (5.2 to 6) | 3.1 (0.5 to 5.7) | 0 (-21.5 to 27.4) | 0.3 (-1.5 to 2.2) |
| Marshall Islands | -3.8 (-4.7 to -2.8) | 1.4 (-0.5 to 3.3) | -3.1 (-3.6 to -2.6) | -1.5 (-2.3 to -0.6) | 16.5 (11.7 to 21.2) | 0 (0 to 0) | -1.4 (-2.2 to -0.5) |
| Mauritania | -0.1 (-1 to 0.8) | 0.5 (-2 to 3.1) | 0.3 (-0.4 to 1.1) | 0.7 (-0.3 to 1.7) | 1 (-0.2 to 2.2) | 0.5 (-21.4 to 28.5) | 0.3 (0 to 0.6) |
| Mauritius | 1.2 (-0.3 to 2.8) | 2.8 (-22.2 to 35.7) | -0.4 (-1.5 to 0.6) | 0.7 (-0.4 to 1.9) | 2.9 (1.1 to 4.8) | 3.1 (-23.1 to 38.3) | 0.7 (-2.6 to 4.1) |
| Mexico | -0.4 (-1.4 to 0.7) | 0.5 (-1.2 to 2.2) | -2.1 (-2.9 to -1.4) | -0.9 (-1.9 to 0) | 2 (0.7 to 3.4) | 1.1 (-13.5 to 18) | -1.9 (-5.7 to 1.9) |
| Mongolia | -24.3 (-26.8 to -21.8) | -1 (-2.7 to 0.8) | -2.4 (-3.4 to -1.4) | -0.6 (-2.1 to 0.9) | 43.2 (40.5 to 45.9) | 1.5 (-8.3 to 12.2) | -1.1 (-2.5 to 0.4) |
| Montenegro | -2.2 (-3 to -1.3) | -2 (-12.3 to 9.5) | -1.7 (-2.4 to -1) | -1.4 (-2.4 to -0.3) | 0.9 (-1.4 to 3.2) | 0 (0 to 0) | 0.1 (-3.7 to 4.1) |
| Morocco | -20.1 (-21.7 to -18.5) | 0.4 (-0.6 to 1.5) | -1.2 (-1.6 to -0.8) | 0.3 (-0.5 to 1.1) | 1.5 (1 to 2.1) | 1.3 (-15.7 to 21.6) | 0.3 (-0.4 to 1) |
| Myanmar | 5.4 (4.7 to 6.1) | 4.7 (0.9 to 8.5) | -2.3 (-3.1 to -1.6) | -1.2 (-2.9 to 0.6) | 1.1 (0.3 to 2) | 1.2 (-12.3 to 16.7) | 0.5 (-0.6 to 1.6) |
| Namibia | -0.5 (-1.1 to 0.2) | 1 (-0.1 to 2.2) | -0.4 (-0.9 to 0) | 4.9 (3.9 to 5.9) | 0.5 (-0.2 to 1.2) | 2.9 (-24.4 to 40.1) | -0.2 (-0.9 to 0.5) |
| Nepal | -0.4 (-2.8 to 2.1) | 0.5 (-21.9 to 29.3) | -0.5 (-1.5 to 0.5) | 1.2 (-0.3 to 2.8) | 2 (-1.7 to 5.9) | NA (NA to NA) | -0.4 (-5 to 4.4) |
| Netherlands | -2.2 (-3.5 to -0.8) | -0.1 (-3.8 to 3.8) | -1.8 (-2.7 to -0.9) | 0.5 (-0.2 to 1.2) | 2.5 (-0.7 to 5.9) | 0 (0 to 0.1) | -2.9 (-5.2 to -0.5) |
| New Zealand | -0.2 (-1.6 to 1.3) | 4.9 (3.5 to 6.3) | -5.2 (-6.2 to -4.1) | 0.5 (-1.9 to 2.9) | 3.3 (1.4 to 5.3) | -1.9 (-24.9 to 28.2) | -0.3 (-4.1 to 3.7) |
| Nicaragua | -2.3 (-3.9 to -0.6) | 0.2 (-1.8 to 2.2) | -0.1 (-0.8 to 0.6) | 10.1 (8.1 to 12.1) | 0.3 (-0.6 to 1.3) | 0 (-17.9 to 21.7) | -0.7 (-2.7 to 1.2) |
| Nigeria | -1.8 (-3.8 to 0.1) | -2.5 (-7.3 to 2.6) | -2.6 (-3.6 to -1.5) | -0.4 (-2.5 to 1.7) | 3.8 (0.4 to 7.3) | 1.1 (-24.8 to 35.9) | -1.4 (-4.2 to 1.5) |
| Northern Mariana Islands | -2.5 (-3.2 to -1.8) | -13.8 (-16.1 to -11.5) | -3 (-3.4 to -2.5) | -1.1 (-2 to -0.2) | -0.1 (-0.7 to 0.5) | 0.1 (-17.5 to 21.4) | -2.3 (-3 to -1.6) |
| Norway | -3.8 (-4.7 to -2.9) | -1.2 (-2.2 to -0.1) | -2.6 (-3.3 to -2) | -0.4 (-1.4 to 0.7) | 0.3 (-0.3 to 0.9) | 0.3 (-17.8 to 22.5) | -1.9 (-2.9 to -1) |
| Oman | -0.3 (-1.5 to 1) | 0.9 (-1.5 to 3.4) | 0 (-1.7 to 1.6) | 2.1 (-0.6 to 5) | 2.8 (1.5 to 4.1) | 3.4 (-12.1 to 21.5) | 0.2 (-1.8 to 2.2) |
| Pakistan | 0.7 (-0.7 to 2.1) | 1.8 (0.2 to 3.6) | -0.7 (-3 to 1.5) | 1.3 (-0.9 to 3.5) | 4.1 (1.9 to 6.4) | 1.5 (-26.7 to 40.7) | 1.9 (-4 to 8.2) |
| Palestine | 0.4 (-0.5 to 1.3) | 1 (-0.7 to 2.7) | -0.1 (-0.5 to 0.3) | 0.8 (0 to 1.6) | 1.6 (0.7 to 2.6) | 0 (0 to 0) | 1 (0 to 2) |
| Panama | -1 (-2 to -0.1) | -1.1 (-4.3 to 2.3) | 0.6 (-0.2 to 1.3) | 14.9 (9.5 to 20.2) | 0.6 (-0.2 to 1.4) | 0.5 (-13.6 to 17) | -1.2 (-2.6 to 0.3) |
| Papua New Guinea | -4.3 (-5.2 to -3.4) | -5.5 (-10.3 to -0.7) | -4.3 (-4.9 to -3.7) | -19.3 (-22.4 to -16.1) | -1.3 (-1.9 to -0.6) | 0 (0 to 0) | -3.9 (-4.8 to -2.9) |
| Paraguay | -1.6 (-3.2 to 0.1) | -1.6 (-9.8 to 7.4) | -1.4 (-2.1 to -0.7) | 0.5 (-0.5 to 1.4) | 2.2 (0.4 to 3.9) | 0 (0 to 0) | -0.5 (-3.1 to 2.2) |
| Peru | -1 (-2.3 to 0.3) | -0.6 (-14.7 to 16) | -1.1 (-1.8 to -0.4) | 0.3 (-1.1 to 1.8) | 3.2 (1.2 to 5.1) | 1.9 (-15.7 to 23.1) | -0.2 (-1.9 to 1.7) |
| Philippines | -1 (-1.8 to -0.2) | -1 (-3.3 to 1.3) | -0.9 (-2.1 to 0.3) | -0.2 (-2 to 1.7) | 0.2 (-1.6 to 2) | 0 (0 to 0) | -0.6 (-3.3 to 2.2) |
| Plurinational State of Bolivia | 0.1 (-0.9 to 1) | -0.1 (-0.2 to -0.1) | -0.2 (-0.7 to 0.2) | 1.2 (0.3 to 2.2) | 0.8 (-0.3 to 1.9) | 0 (0 to 0) | -0.2 (-1.5 to 1.1) |
| Poland | -25.5 (-27.9 to -23) | -0.3 (-1.7 to 1.2) | -2.8 (-4.2 to -1.4) | 0.6 (-1.1 to 2.4) | 3.1 (2 to 4.1) | 2.9 (-9.7 to 17.2) | -1.5 (-3.2 to 0.3) |
| Portugal | -1.2 (-2.6 to 0.3) | 0.5 (-4.3 to 5.7) | -1.1 (-2.3 to 0.2) | -0.1 (-2.1 to 2) | 3.1 (2.9 to 3.3) | 0 (0 to 0) | -0.8 (-4.7 to 3.4) |
| Principality of Monaco | -0.1 (-0.8 to 0.7) | 1.1 (-0.2 to 2.4) | 0 (-0.8 to 0.8) | 1.9 (0.4 to 3.5) | 3.5 (2.4 to 4.6) | 3.3 (-10.1 to 18.7) | 0.3 (-1 to 1.5) |
| Puerto Rico | -1.1 (-2.4 to 0.2) | 0.7 (-1 to 2.4) | -1.3 (-2.8 to 0.2) | 1.2 (-1.3 to 3.7) | 1.9 (0.9 to 3) | 1.7 (-10.4 to 15.4) | -0.7 (-3.1 to 1.7) |
| Qatar | -16.9 (-19.1 to -14.7) | 0.1 (-1.5 to 1.8) | -0.8 (-1.7 to 0.1) | 1.4 (1.2 to 1.6) | 5.5 (5.2 to 5.8) | 1.2 (-14.5 to 19.7) | -0.5 (-1.8 to 0.8) |
| Republic of Cabo Verde | -1.8 (-2.4 to -1.1) | 2.2 (1 to 3.4) | -1.1 (-1.4 to -0.8) | 0.2 (-0.4 to 0.9) | -0.2 (-0.7 to 0.3) | -0.9 (-17 to 18.4) | -2.5 (-3 to -1.9) |
| Republic of Korea | -3.1 (-4 to -2.3) | -1.1 (-2 to -0.1) | -1.3 (-1.8 to -0.8) | 0 (-0.8 to 0.8) | 1.2 (0.4 to 2.1) | 0.1 (-21.6 to 27.8) | -0.4 (-1.4 to 0.7) |
| Republic of Moldova | -2.3 (-3.1 to -1.4) | -1.3 (-2.7 to 0.1) | -1.1 (-1.9 to -0.3) | -0.6 (-1.7 to 0.5) | 1.4 (-1.6 to 4.3) | 0 (0 to 0) | -7.2 (-8.4 to -6) |
| Republic of Nauru | -2 (-3.4 to -0.5) | -3.2 (-9.2 to 3.2) | -3.8 (-4.6 to -3) | -2.4 (-3.5 to -1.2) | -0.8 (-2 to 0.5) | 0 (-0.1 to 0) | -2.2 (-4.1 to -0.2) |
| Republic of Palau | -0.8 (-2.2 to 0.7) | 0.4 (-1.7 to 2.6) | -106.1 (-111.2 to -100.9) | -1.8 (-2.9 to -0.6) | 1.3 (-0.2 to 2.7) | -1.4 (-23.6 to 27.3) | -1.7 (-2.7 to -0.7) |
| Republic of San Marino | 2.7 (1.8 to 3.7) | 2.5 (0.7 to 4.4) | -0.2 (-0.6 to 0.3) | 0.3 (-0.7 to 1.3) | 2 (1.3 to 2.8) | 0 (0 to 0) | 26.7 (19.4 to 34.1) |
| Romania | 0 (-1.1 to 1.1) | 2 (0.7 to 3.3) | 0.1 (-0.3 to 0.5) | 1.4 (0.7 to 2.1) | 2.4 (1.7 to 3.2) | 1.5 (-20.6 to 29.8) | 1.6 (0.7 to 2.5) |
| Russian Federation | 40.5 (31.3 to 49.8) | 1.7 (0.3 to 3.2) | -1.7 (-2.1 to -1.3) | -11.7 (-15.3 to -8.2) | 0.2 (-0.4 to 0.8) | -1.3 (-19.3 to 20.8) | -0.4 (-1 to 0.1) |
| Saint Kitts and Nevis | -1.5 (-2.2 to -0.8) | 0.7 (-0.4 to 1.9) | -20 (-21.8 to -18.2) | 0.2 (-1.3 to 1.6) | 1.1 (0 to 2.2) | -0.4 (-13.3 to 14.4) | -1.8 (-3.4 to -0.1) |
| Saint Lucia | -1.5 (-2.1 to -1) | -1.2 (-2 to -0.3) | -1 (-1.8 to -0.3) | 0.1 (-1.4 to 1.5) | 1.9 (0.6 to 3.2) | 0.1 (-14.4 to 17) | -1 (-2.5 to 0.6) |
| Saint Vincent and the Grenadines | -0.3 (-0.9 to 0.4) | 14.4 (6.5 to 22.2) | -1 (-2 to 0) | 0 (-1.7 to 1.7) | 2.2 (0.6 to 3.9) | 0 (0 to 0.1) | -0.9 (-3.1 to 1.3) |
| Samoa | -0.1 (-1.3 to 1.2) | -0.5 (-5.4 to 4.7) | -0.2 (-1.1 to 0.8) | 0.8 (-0.9 to 2.5) | 1.6 (1.5 to 1.8) | NA (NA to NA) | -0.6 (-3.7 to 2.7) |
| Sao Tome and Principe | -3.4 (-4.3 to -2.6) | -2 (-3.1 to -0.9) | -279.1 (-294.6 to -263.6) | -2.3 (-3 to -1.6) | -0.8 (-1.6 to 0.1) | 0 (0 to 0) | -2.6 (-3.7 to -1.6) |
| Saudi Arabia | 1.5 (-0.4 to 3.5) | 0.7 (-0.5 to 2) | 2.2 (1.2 to 3.2) | 2.2 (1.1 to 3.4) | 3.5 (0.9 to 6.1) | 0 (0 to 0) | 2.1 (-3.1 to 7.7) |
| Senegal | 0.2 (-1.5 to 2) | -2.9 (-25.8 to 27.1) | -0.8 (-1.5 to -0.1) | -2.6 (-5.8 to 0.7) | 3.2 (1.9 to 4.4) | 1.6 (-17.9 to 25.8) | 5.8 (4.7 to 6.8) |
| Serbia | 0.3 (-1.3 to 1.9) | -0.9 (-4.6 to 3) | -2 (-3.2 to -0.7) | -0.2 (-1.7 to 1.3) | 3.3 (0.4 to 6.3) | 0 (0 to 0) | 0.1 (-3.5 to 3.9) |
| Seychelles | -19.1 (-27.2 to -11) | 0.6 (-0.7 to 2) | -0.2 (-0.6 to 0.2) | 1 (0.3 to 1.8) | 0.7 (0.1 to 1.4) | 0 (-21.5 to 27.4) | -0.2 (-0.9 to 0.5) |
| Singapore | -0.6 (-1.1 to 0) | 5.5 (4.4 to 6.6) | -1.3 (-1.7 to -0.8) | -0.6 (-1.4 to 0.3) | 1 (-0.8 to 2.8) | 0.8 (-17.8 to 23.5) | -0.8 (-2.6 to 0.9) |
| Slovakia | -4 (-4.6 to -3.5) | -1.4 (-4.2 to 1.5) | -2.8 (-3.8 to -1.8) | -2.3 (-3.6 to -1) | 0.7 (-0.5 to 1.9) | -0.9 (-22.9 to 27.3) | -1.6 (-2.9 to -0.1) |
| Slovenia | 0.2 (-0.6 to 1) | 0.3 (-0.7 to 1.3) | -0.4 (-0.8 to 0.1) | 0.3 (-0.5 to 1) | 0.4 (-0.1 to 0.9) | 0 (0 to 0) | 0 (-0.7 to 0.7) |
| Socialist Republic of Viet Nam | -0.3 (-1 to 0.4) | -3 (-4.3 to -1.8) | -1 (-1.5 to -0.5) | -0.3 (-1 to 0.4) | 12.7 (-4.9 to 30.3) | 0.4 (-17.9 to 22.7) | 0 (-0.7 to 0.7) |
| Solomon Islands | 0.6 (-0.1 to 1.3) | -0.1 (-5.6 to 5.7) | 0.6 (-0.1 to 1.3) | 0.1 (-1.4 to 1.7) | 1.3 (-0.3 to 2.9) | 0 (0 to 0.1) | 1.2 (0.6 to 1.7) |
| South Africa | -24.8 (-32.1 to -17.5) | -0.1 (-1 to 0.8) | -1.5 (-2.2 to -0.8) | 1.4 (0.1 to 2.8) | 1.2 (0 to 2.5) | 0 (0 to 0) | -0.9 (-2.7 to 1) |
| Spain | -3.5 (-4.3 to -2.7) | -1.8 (-3.1 to -0.6) | -2.3 (-2.7 to -2) | -1.5 (-2.2 to -0.8) | 1 (0.3 to 1.7) | 0 (-21.5 to 27.4) | -0.6 (-1.3 to 0.2) |
| Sri Lanka | -1.4 (-3.3 to 0.5) | 2.2 (-0.5 to 5) | 0.2 (-0.8 to 1.1) | 2.7 (0.8 to 4.6) | 1.2 (-0.9 to 3.3) | 0.5 (-16.6 to 21.3) | -2.8 (-4.8 to -0.8) |
| Suriname | -0.6 (-1.2 to 0.1) | 7.2 (5.1 to 9.3) | -0.2 (-1 to 0.6) | 1 (-0.4 to 2.5) | 1.7 (0.4 to 3) | 0.8 (-13.9 to 18) | -0.8 (-2.1 to 0.6) |
| Sweden | -1.7 (-2.4 to -1.1) | -0.6 (-1.7 to 0.4) | -2.3 (-2.9 to -1.6) | -3.5 (-6.5 to -0.5) | -1.4 (-2.1 to -0.8) | -1.7 (-20.6 to 21.5) | -2.5 (-3.3 to -1.6) |
| Switzerland | -5.3 (-6 to -4.6) | -51.8 (-58.6 to -45) | -0.7 (-1.3 to 0) | 0.4 (-0.7 to 1.5) | -0.3 (-1 to 0.5) | -0.9 (-21.1 to 24.5) | -1.2 (-2.1 to -0.2) |
| Taiwan (Province of China) | -1 (-2.2 to 0.2) | 0 (-2.3 to 2.4) | -2.1 (-2.6 to -1.7) | -0.3 (-1.2 to 0.6) | 3.1 (2.1 to 4.1) | 2 (-7.9 to 13) | 0.9 (-0.1 to 1.8) |
| Tajikistan | -2.3 (-4.1 to -0.5) | -6.2 (-15.1 to 3.7) | -2.7 (-4 to -1.5) | -0.1 (-2.3 to 2.1) | 0.4 (-0.5 to 1.4) | -0.4 (-17.4 to 20.1) | -2.2 (-3.5 to -0.9) |
| Thailand | -1.9 (-2.9 to -0.9) | -5.4 (-7.5 to -3.3) | -80.3 (-87.7 to -72.8) | -0.7 (-2 to 0.7) | 3.1 (1.4 to 4.8) | 1.2 (-12.8 to 17.4) | -1.9 (-3.2 to -0.6) |
| The former Yugoslav Republic of Macedonia | -1.4 (-2.7 to 0) | -1.5 (-5.5 to 2.6) | -15.4 (-17.6 to -13.2) | -1.6 (-4.6 to 1.6) | 5.3 (0.6 to 10.2) | 0 (0 to 0) | 0.4 (-2 to 2.9) |
| Timor-Leste | -0.9 (-1.7 to -0.1) | 0.1 (-2.9 to 3.1) | -0.8 (-1.6 to 0) | 0.5 (-1 to 2) | 1 (-0.4 to 2.3) | 0 (0 to 0) | -0.8 (-2.8 to 1.3) |
| Tonga | -0.4 (-1 to 0.2) | 0.2 (-0.9 to 1.3) | -0.1 (-0.9 to 0.7) | 0.7 (-0.6 to 2.1) | 1 (0 to 2) | 0.8 (-12 to 15.4) | -0.6 (-2 to 0.9) |
| Trinidad and Tobago | -0.6 (-1.3 to 0.2) | 1.5 (-4.2 to 7.6) | -0.3 (-0.7 to 0) | 0.6 (-0.2 to 1.4) | 0.2 (-0.6 to 1.1) | -1 (-13.2 to 13) | -1.4 (-2.1 to -0.6) |
| Tunisia | -0.8 (-2.5 to 0.9) | 2.1 (-4.3 to 9) | -40.1 (-46.8 to -33.5) | -0.7 (-2.4 to 0.9) | 6.8 (5.5 to 8.1) | 0.4 (0.3 to 0.4) | 4.9 (3.5 to 6.2) |
| Turkey | 0.1 (-0.7 to 0.9) | 0.7 (-1.9 to 3.2) | 0.2 (-0.4 to 0.9) | 1.2 (-0.3 to 2.6) | 2.4 (1.2 to 3.5) | 0 (0 to 0.1) | 0.6 (-1.5 to 2.8) |
| Turkmenistan | -1.3 (-2.2 to -0.4) | -0.5 (-4.7 to 3.8) | -62.7 (-74.1 to -51.3) | 1.3 (0.4 to 2.2) | 0.7 (-0.1 to 1.6) | -0.4 (-12.8 to 13.9) | -1.3 (-2.3 to -0.4) |
| Tuvalu | 0.4 (-0.4 to 1.2) | 1.6 (0.1 to 3) | -0.7 (-1.1 to -0.2) | 0.5 (-0.6 to 1.6) | 1.5 (0.9 to 2.1) | 0 (0 to 0) | -0.1 (-0.7 to 0.5) |
| Ukraine | -4.7 (-6.5 to -2.9) | -3.2 (-7.9 to 1.9) | -5 (-5.8 to -4.1) | -2.6 (-3.6 to -1.6) | -2.6 (-4 to -1.3) | -4.3 (-22.9 to 18.8) | -5.1 (-7.4 to -2.7) |
| United Arab Emirates | -3.1 (-3.9 to -2.4) | -0.6 (-1.6 to 0.4) | -194.4 (-213.2 to -175.6) | -39.5 (-44.5 to -34.6) | 0.5 (-0.1 to 1.2) | 0 (0 to 0) | -1.6 (-2.3 to -0.8) |
| United Kingdom of Great Britain and Northern Ireland | -5.9 (-9.2 to -2.7) | 9.2 (7.7 to 10.7) | -1.3 (-2.5 to -0.1) | 0.9 (-2.3 to 4.1) | 3 (-1 to 7.1) | 0 (0 to 0) | -0.7 (-5.6 to 4.6) |
| United Republic of Tanzania | -1.6 (-2.3 to -0.8) | 1.3 (-0.2 to 2.8) | -2 (-3.1 to -0.9) | -0.3 (-2 to 1.4) | 0.9 (0.1 to 1.6) | 1 (-9.7 to 13) | -1.6 (-2.9 to -0.3) |
| United States of America | -2.7 (-3.4 to -1.9) | -0.7 (-2 to 0.6) | -1.2 (-1.7 to -0.6) | 0.2 (-0.7 to 1.2) | 0 (-0.6 to 0.6) | -1.2 (-18 to 19) | -32.2 (-34.7 to -29.7) |
| United States Virgin Islands | -0.9 (-1.6 to -0.3) | -7.6 (-12.3 to -3) | -1.5 (-1.9 to -1.1) | -0.2 (-1 to 0.6) | 1.2 (0.8 to 1.7) | 0.3 (0.2 to 0.3) | 0 (-0.6 to 0.6) |
| Uruguay | -0.7 (-3 to 1.6) | 2.4 (-3.1 to 8.1) | -0.3 (-1.1 to 0.6) | -0.1 (-1.7 to 1.5) | 2.5 (1.4 to 3.7) | 0.1 (0.1 to 0.1) | 1.3 (-0.2 to 2.9) |
| Uzbekistan | -10.1 (-12.2 to -8.1) | 2.7 (-5.1 to 11) | -1.6 (-2.7 to -0.5) | 0.3 (-1.4 to 2) | 1 (-0.7 to 2.7) | 2.5 (-22.1 to 34.8) | -1.5 (-4.5 to 1.6) |
| Vanuatu | 0.7 (-1.3 to 2.8) | 7.4 (1.5 to 13.7) | 6.9 (3 to 10.9) | 2 (-0.8 to 5) | 3.4 (3.2 to 3.7) | 3.1 (-23.1 to 38.3) | 1.7 (-0.5 to 4) |
| Zambia | -1.4 (-2.5 to -0.3) | 0.1 (-0.9 to 1.1) | -0.3 (-1.4 to 0.9) | 1.4 (-0.3 to 3.1) | 3.6 (0.5 to 6.9) | 2.7 (-21.6 to 34.6) | -0.3 (-6 to 5.8) |
| Zimbabwe | -2.6 (-3.5 to -1.7) | -0.6 (-1.6 to 0.3) | -2.5 (-3.2 to -1.9) | -1.1 (-2.2 to 0.1) | 1.9 (-0.8 to 4.6) | -0.8 (-27.4 to 35.6) | -1.2 (-3.8 to 1.5) |
